# Base-Free C–N Cross-Electrophile Coupling

**DOI:** 10.1021/jacs.6c05089

**Published:** 2026-07-31

**Authors:** Samya Samanta, Lauv Patel, Diyasha Manna, Melissa Ojeda, David C. Powers

**Affiliations:** Department of Chemistry, 14736 Texas A&M University, College Station, Texas 77843, United States

## Abstract

Metal-catalyzed carbon–nitrogen (C–N) cross-coupling—a philicity-matched reaction of carbon electrophiles with nitrogen nucleophiles under basic conditions—is a foundational tool in modern medicinal chemistry. Here, we report a philicity-mismatched nickel-catalyzed C–N cross-electrophile coupling (XEC) that combines electrophilic carbon and nitrogen precursors. Because the method relies on electrophilic nitrogen precursors, basic additives are not required to activate the nitrogen coupling partner. This mild protocol exhibits broad functional group tolerance, including base-sensitive substrates, and is compatible with late-stage applications. The electrophilic nitrogen precursors can be accessed from diverse starting materials, including C–H bonds and olefins. We demonstrate a two-step, one-pot *N*-phenothiazination, C–N XEC sequence that provides the platform for application of C–N XEC to the functionalization of amine-containing bioactive small molecules. Strategic implementation of C–N XEC enables synthesis of designer secondary and tertiary amines as well as unsymmetrical diamines. Mechanistic investigations indicate the C–N XEC proceeds via a Ni­(II)–aryl-amido intermediate and enforces cross-selectivity by avoiding Ni­(II)–aryl-halide intermediates associated with homocoupling. These findings establish a paradigm for base-free C–N bond construction and introduce a mechanistic rationale for controlling C–X XEC selectivity.

## Graphical abstract

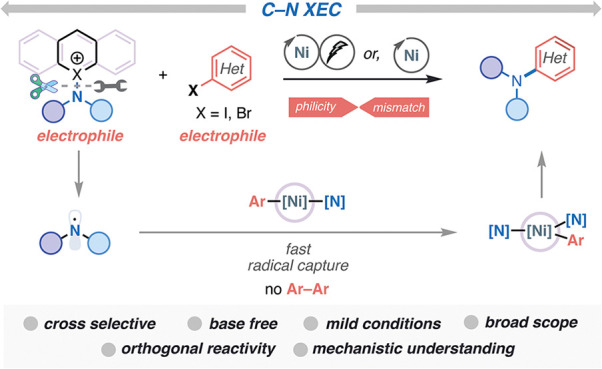

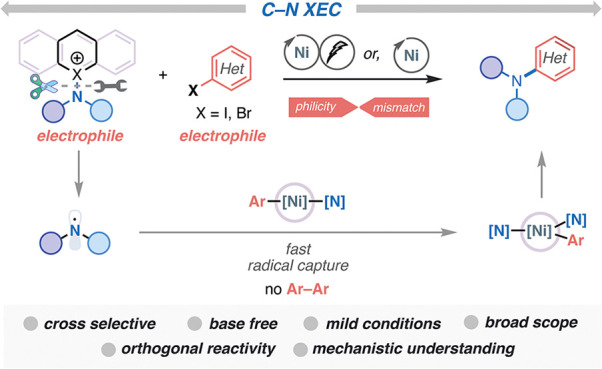

## Introduction

C–N coupling reactions are among the most broadly deployed synthetic tools in medicinal chemistry due to the ubiquity of bioactive amines in pharmacologically active natural products and small-molecule therapeutics.^1–3^ Broadly, the synthetic logic of C–N coupling can be conceptualized according to the philicity of the coupling partners. Philicity-matched strategies include (i) C–N cross-coupling methods, such as the Cu-catalyzed Ullmann reaction and Pd-catalyzed Buchwald-Hartwig chemistry, which utilize nucleophilic nitrogen sources and electrophilic carbon sources,^4–6^ and (ii) umpolung C–N coupling methods, which combine electrophilic nitrogen synthons with nucleophilic carbon precursors.^7^ Philicity-mismatched strategies include (iii) oxidative C–N coupling methods, such as the Cu-catalyzed Chan-Lam reaction, which employ nucleophilic nitrogen and carbon sources in the presence of an oxidant,^8^ and (iv) reductive C–N cross-electrophile coupling (XEC) reactions, which combine electrophilic nitrogen and carbon sources in the presence of a reductant (Figure 1a). Enormous progress has established cross-coupling, umpolung-based methods, and oxidative coupling as robust synthetic platforms for C–N bond construction in academic and industrial settings.^2–6,8,9^ In contrast, C–N XEC remains underdeveloped and applicable only to the synthesis of biaryl amines and aliphatic imines.^10–12^

**Figure 1:**
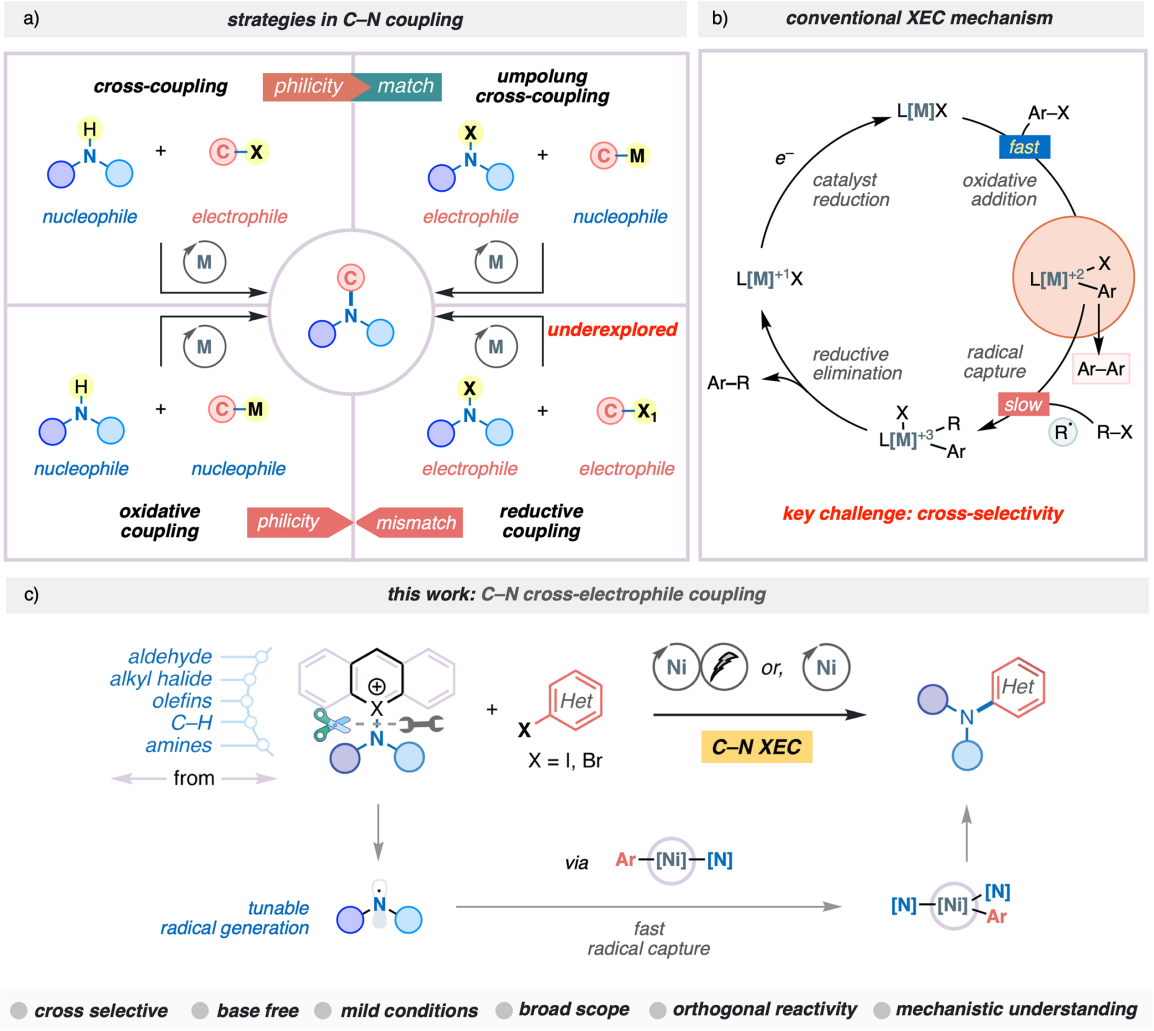
Overview of C–N coupling chemistry and summary of the C–N XEC developed here. (a) C–N coupling methods can be categorized according to the philicity of the coupling partners. (b) C–C XEC represents a complementary approach to C–C bond construction as compared to C–C cross-coupling. Metal–aryl-halide (M­(X)–Ar) intermediates in C–C XEC catalysis can give rise to homocoupling byproducts. (c) C–N cross-electrophile coupling (XEC) combines *N*-centered radical precursors and aryl halides. *N*-centered radical precursors can be accessed from diverse functional groups. C–N XEC proceeds under base-free conditions, is compatible with late-stage application and native amine functionalization, and avoids C–C homocoupling by accessing a nitrogen-first XEC mechanism.

Carbon–carbon (C–C) XEC has garnered intense recent interest as a complement to cross-coupling methods due to the (1) broad availability of organohalides and pseudohalides, (2) demonstrated functional group tolerance of XEC methods, (3) opportunity to avoid stoichiometric quantities of reactive organometallics, and (4) availability of strategies to achieve high cross-selectivity.^13–19^ Notably, the discovery and optimization of Ni-catalyzed methods has enabled selective C–C XEC.^19^ From a mechanistic perspective, homocoupling typically arises from poorly matched rates of oxidative addition and radical capture (Figure 1b): Slower radical capture enables Ni–aryl-halide intermediates to undergo undesired transmetalation reactions to generate diarylated species that give rise to C–C homocoupling products.^19,20^ Faster radical generation—an attractive strategy to balance the relative rates of substrate activation—has enabled high cross-selectivity in C–C XEC methods.^19^ Similar approaches have not yet been deployed in the context of C–N coupling.

We have been attracted to C–N XEC as a distinct—and potentially complementary—platform for C–N bond construction.^21^ In addition to providing a heretofore unknown bond disconnection, we reasoned that because XEC does not rely on the nucleophilicity of amine coupling partners, it may be inherently compatible with weak-base or base-free conditions, which remain a forefront challenge for method development in C–N cross-coupling.^22–27^ Further, we envisioned that the development of C–N XEC would provide strategic opportunities to directly utilize *N*-centered radicals from electrophilic nitrogen sources in coupling reactions. Given the significant recent progress in accessing electrophilic *N*-centered radical precursors such as *N*-aminopyridinium salts from activated C–H bonds^28,29^ and olefins,^30,31^ as well as sulfilimines from amines,^32,33^ the development of direct coupling methods with *N*-centered radicals would unlock an orthogonal chemical space in C–N coupling, both synthetically and mechanistically.

Here, we describe C–N XEC under mild, nonbasic conditions using either chemical or electrochemical protocols (Figure 1c). Using *N*-centered radical precursors, we demonstrate a cross-selective C–N XEC that displays broad scope and is compatible with late-stage application. The requisite *N*-centered radical precursors can be generated from an array of starting materials, including aldehydes, alkyl halides, olefins, C–H bonds, and amines, highlighting the generality and modularity of this method. Moreover, achieving C–N XEC under electrochemical conditions enables substrate activation at precise potentials, thus engendering selective coupling under mild conditions. C–N XEC enables modular build-and-couple synthesis of functional amines and aziridines from activated C–H bonds and olefins, respectively. Chemoselective C–N coupling in the presence of nucleophilic amines provides an orthogonal C–N coupling opportunity for 1,2-diamine synthesis. Mechanistic studies imply rapid capture of *N*-centered radicals by a Ni­(II)–aryl-amido resting state. This mechanism avoids Ni­(II)–aryl-halide intermediates that are often associated with homocoupling in C–C XEC. Realization of C–N XEC adds an orthogonal strategy in the C–N coupling toolbox and provides streamlined access to complex nitrogen-containing small molecules.

## Results and Discussion

Based on the reductive lability of *N*-aminopyridinium salts,^29,34,35^ we envisioned that reductive activation of the N–N bond in *N*-aminopyridinium salts would generate *N*-centered radicals that could be engaged in metal-catalyzed C–N XEC with aryl halides. While, ultimately, we sought an electrochemical method (vide infra), we initiated our studies by examining the XEC of *N*-aminopyridinium salt **1a** (*E*_onset_ = −1.62 V vs. Fc^+^/Fc) and *p*-tolyl iodide (**2a**) in the presence of chemical reductants. After systematic evaluation of the reaction parameters (see Tables S1–S13 for optimization details), we found that a combination of **1a** and **2a** (1.5 equiv) in the presence of NiBr_2_(dme) (10 mol %) as a catalyst, 4,4′-di-*tert*-butyl-2,2′-bipyridine (dtbbpy, 13 mol %) as a ligand, NaI as an additive, and Zn as a terminal reductant in DMA at 23 °C afforded XEC product **3a** in 76% yield (Table 1, entry 1). No homocoupling was observed under these conditions. Catalyst loading has a significant impact on the observed XEC selectivity: While 10 mol % NiBr_2_(dme) provided exclusive cross-selectivity, increasing the loading to 20 and 50 mol % provided 3.5:1 and 0.8:1 selectivity, respectively (Table 1, entries 2 and 3). Electron-rich ligands, which have been employed in Ni-catalyzed C–C coupling for enhanced cross-selectivity,^19^ also provide exclusive cross-selectivity but with diminished yields (Table 1, entries 4 and 5).

**Table 1:**
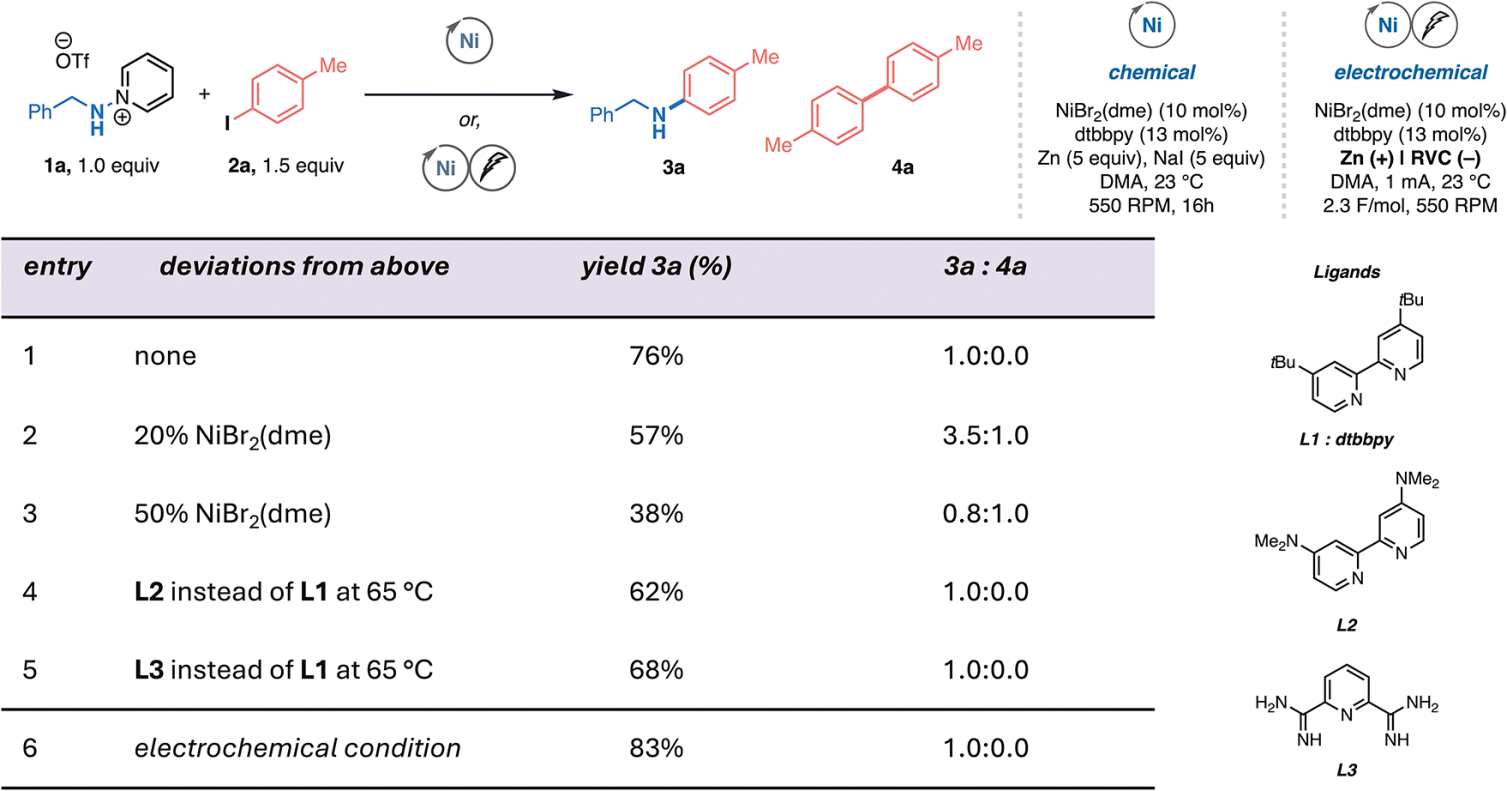
Optimization of C–N XEC^a^

a Chemical conditions: **1a** (0.050 mmol), **2a** (0.075 mmol), NiBr_2_(dme) (5.00 μmol), dtbbpy (6.50 μmol), zinc powder (0.250 mmol), NaI (0.250 mmol) in DMA (0.3 mL) for 16 h at 23 °C. Electrochemical conditions: **1a** (0.060 mmol), aryl halide (0.066 mmol), NiBr_2_(dme) (6.00 μmol), dtbbpy (7.80 μmol) in DMA (3 mL) in undivided cell, Zn­(+)|RVC(−), 1 mA until 2.30 F/mol was passed at 23 °C. Yields were determined by ^1^H NMR against 1,3,5-trimethoxybenzene.

We translated the developed chemical conditions to an analogous electrochemical cross-electrophile C–N coupling (see Tables S17–S24 for optimization details), which is generally more efficient than the described chemical conditions while maintaining exclusive cross-selectivity even at 1.1 equiv of **2**. In our optimized electrochemical conditions, electrolysis of a DMA solution of *N*-aminopyridinium salt **1a**, aryl halide **2a** (1.1 equiv), NiBr_2_(dme) (10 mol %), and dtbbpy (13 mol %) with an RVC cathode and zinc anode at 23 °C afforded C–N coupled product **3a** in 83% (2.3 F/mol at 1 mA constant current electrolysis) with no homocoupling observed (Table 1, entry 6). Of significance, this reaction operates without the need for an exogenous supporting electrolyte; *N*-aminopyridinium salts are sufficiently conductive to enable efficient electrocatalysis.

### Substrate Scope

#### Aryl Halide Scope

To evaluate the impact of aryl halide structure on C–N XEC efficiency, we examined the XEC of *N*-aminopyridinium salt **1a** with various aryl halides (Figure 2). *p*-Tolyl iodide generates C–N coupled product **3a** in 77% yield. Iodobenzene participates in coupling to afford **3b** in 66% yield. Electron-deficient (*p*-COCH_3_, *p*-CO_2_Et, *p*-CF_3_, *p*-F, *p*-Cl, *p*-Br, and *p*-I (**3c**–**3i**)) and electron-rich (*p*-OMe (**3j**) and *o*-OMe (**3n**)) aryl halides, which are challenging coupling partners in Ni-catalyzed C–N cross-coupling chemistry due to slow oxidative addition,^36,37^ are excellent XEC substrates. Of note, boronic esters are compatible with the developed reductive coupling (i.e., **3k**) and provide the opportunity for subsequent orthogonal coupling chemistry. Aldehydes, which are not often compatible in C–N coupling protocols due to competing imine condensation^38^ and facile decomposition,^27^ undergo efficient coupling (i.e., **3m**). The base-free C–N XEC conditions are compatible with unprotected alcohols (**3o**) and *N*-heterocyclic aryl halides. These functional groups are synthetically important but are often incompatible with base-promoted C–N coupling due to catalyst poisoning.^21,27^ Indole and pyridine, which are among the top 10 most abundant nitrogen heterocycles in FDA-approved drugs,^3,39^ are well tolerated (**3p**–**3s**). Benzoxazole- and thiophene-containing heteroaryl iodides perform with modest yields in this C–N coupling sequence (**3t** and **3u**).

**Figure 2:**
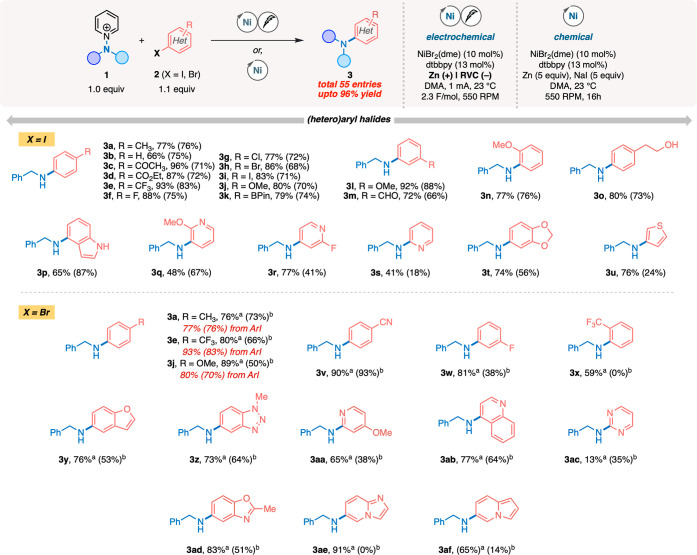
C–N XEC Scope. Electrochemical conditions: **1** (0.120 mmol), **2** (0.132 mmol), NiBr_2_(dme) (12.0 μmol), dtbbpy (15.6 μmol) in DMA (5 mL) in an undivided cell, Zn­(+)|RVC(−), 1 mA until 2.30 F/mol was passed at 23 °C at 550 rpm. ^a^LiBr (0.180 mmol) as an additive, 0.5 mA. Chemical conditions: **1** (0.100 mmol), aryl halide (0.110 mmol), NiBr_2_(dme) (10.0 μmol), dtbbpy (13.0 μmol), zinc powder (0.500 mmol), NaI (0.500 mmol) in DMA (0.5 mL) for 16 h at 23 °C at 550 rpm. ^b^Reaction run at 65 °C for 30 min ^c^bpy (13.0 μmol) as a ligand, Ni­(bpp)_2_ (5 mol %) as a mediator. Reaction yields: Isolated yield from electrochemical conditions (^1^H NMR yield from chemical conditions determined against 1,3,5-trimethoxybenzene).

Aryl bromides are also efficient substrates for C–N XEC (see Tables S21–S24 for optimization details). Under the optimized electrochemical conditions, aryl bromides with diverse electronic demand engage in C–N XEC as efficiently as the corresponding iodides: **1a** couples with *p*-tolyl halides (76% from *p*-tolyl bromide and 77% from *p*-tolyl iodide); with *p*-trifluoromethyl aryl halides (80% (Br) and 93% (I)); and with *p*-methoxy aryl halides (89% (Br) and 80% (I)). Electron-deficient 4-bromobenzonitrile provides C–N coupling product **3v** in 90% yield. *Meta*- and *ortho*-substituted aryl bromides engage in C–N XEC to afford **3w** or **3x** in 81 and 59% yields, respectively. Heteroaryl bromides, which are more readily available than the corresponding iodides, are also efficient under the optimized conditions. Benzofuran-, benzotriazole-, and pyridine-containing heteroaryl bromides are well-tolerated during the C–N coupling (**3y–3aa**). 4-Aminoquinoline derivatives, which have prominent pharmaceutical relevance,^40–42^ can be prepared using the C–N XEC condition with good yield (**3ab**, 77%). Pyrimidine-based heteroaryl bromide **3ac** is an inefficient partner. Base-sensitive heteroaryl bromides which have been problematic in C–N coupling with NaOTMS,^23^ undergo coupling in excellent to moderate yields (**3ad**–**3af**) under the C–N XEC condition.

Aryl chlorides and aryl triflates are ineffective substrates in the developed C–N XEC. Activation of these substrates is typically proposed to proceed at Ni(0) intermediates, which we speculate are not accessed under the described reaction conditions (vide infra).^43^

To canvass the base sensitivity of C–N XEC in greater detail, we carried out an additive screening study following the protocol developed by Glorius et al. (Figure 3).^44^ Additive screening was carried by examining the addition of a set of base-sensitive heterocycles and functional groups not contained within Figure 2 to the coupling of *N*-aminopyridinium salt **1a** with *p*-tolyl iodide (**2a**) (Figure S3). Base-sensitive five-membered heterocycles, such as furan (**A1**), pyrrole (**A2**), imidazole (**A3**), pyrazole (**A4**), and triazole (**A5**), are well-tolerated in the C–N XEC conditions; good coupling yield and excellent additive recovery were observed. Additives with low p*K*_a_s (**A6**, **A7**), a chromone-containing a γ-pyrone core (**A8**), and an alkyne (**A9**) are also well-tolerated under this additive-screening protocol (for additional details of additive screening, see the Supporting Information, Section C.2).

**Figure 3:**
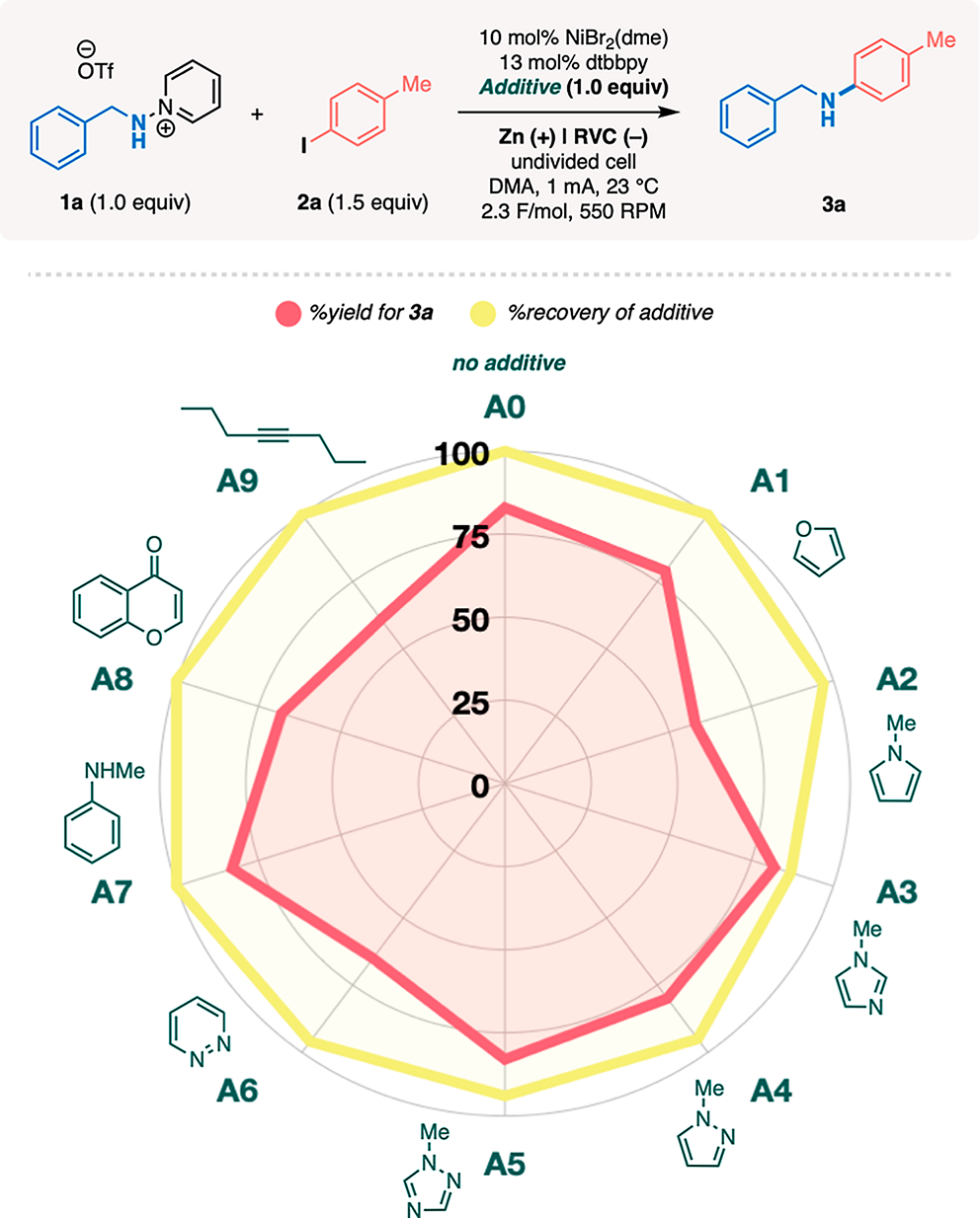
Additive screening for C–N XEC between **1a** and **2a**. Reactions were performed on a 0.06 mmol scale. Yields were determined by ^1^H NMR analysis of crude reaction mixtures using 1,3,5-trimethoxybenzene as the internal standard.

#### *N*-Pyridinium Amine Scope

To evaluate the impact of *N*-aminopyridinium structure on C–N XEC efficiency, we examined the XEC of *p*-tolyl iodide with various *N*-aminopyridinium salts (Figure 4). Primary pyridinium amine salts are excellent XEC partners. Significant variation of the steric demand at the α-position is tolerated; for example, adamantyl amine **1aj** engages in efficient coupling to afford **3aj** (92% yield). Benzylic and allylic pyridinium amine salts (**3ak**–**3an**) are similarly competent in C–N XEC. Biologically and pharmacologically active β-phenethylamine derivative **1ap** engaged in C–N XEC in 80% yield, which is a substantial improvement over C–N coupling with aryl boronic acids (**3ap**).^45^

**Figure 4:**
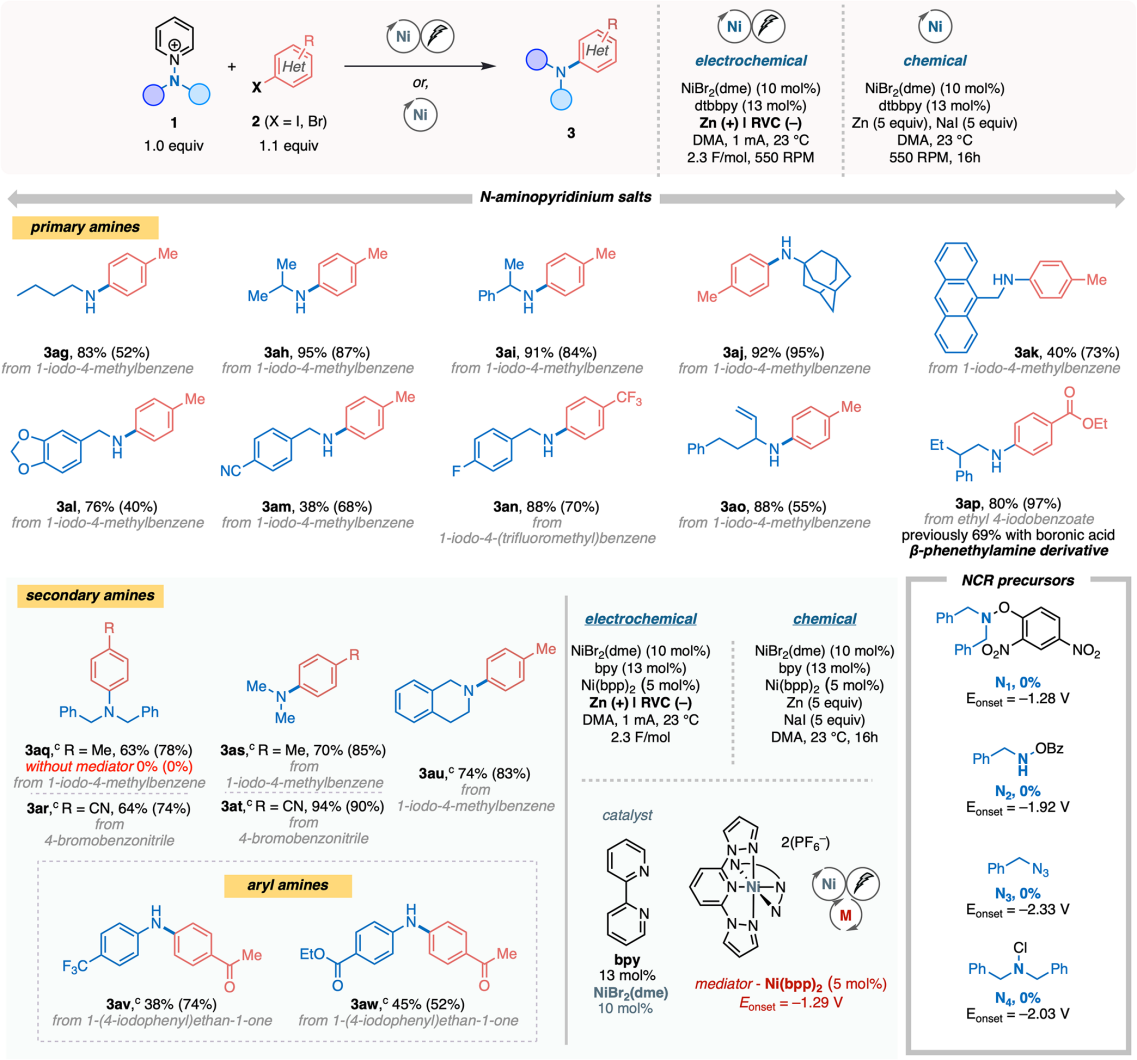
C–N XEC Scope. Electrochemical conditions: **1** (0.120 mmol), **2** (0.132 mmol), NiBr_2_(dme) (12.0 μmol), dtbbpy (15.6 μmol) in DMA (5 mL) in an undivided cell, Zn­(+)|RVC(−), 1 mA until 2.30 F/mol was passed at 23 °C at 550 rpm. ^a^LiBr (0.180 mmol) as an additive, 0.5 mA. Chemical conditions: **1** (0.100 mmol), aryl halide (0.110 mmol), NiBr_2_(dme) (10.0 μmol), dtbbpy (13.0 μmol), zinc powder (0.500 mmol), NaI (0.500 mmol) in DMA (0.5 mL) for 16 h at 23 °C at 550 rpm. ^b^Reaction run at 65 °C for 30 min ^c^bpy (13.0 μmol) as a ligand, Ni­(bpp)_2_ (5 mol %) as a mediator. Reaction yields: Isolated yield from electrochemical conditions (^1^H NMR yield from chemical conditions determined against 1,3,5-trimethoxybenzene).

Secondary *N*-aminopyridinium salts do not participate in efficient C–N XEC using our initially optimized conditions (see Tables S14 and S15 for examples). We speculated that the challenge with secondary substrates may be due to the more facile reduction as compared to primary *N*-centered radical precursors—*E*_onset_ of **1an** = −1.36 V vs. Fc^+^/Fc; *E*_onset_ of **1a** = −1.62 V vs. Fc^+^/Fc—leading to mismatched rates of *N*-centered radical generation of XEC (vide infra). Based on this hypothesis, we reoptimized our reaction conditions by replacing dtbbpy with 2,2′-bipyridine (bpy) and introducing [Ni­(bpp)_2_]­(PF_6_)_2_ (*E*_onset_ = −1.29 V) as an electron transfer mediator to convey interfacial electron transfer to the bulk and to modulate the rate of *N*-centered radical generation (bpp = 2,6-bis­(pyrazol-1-yl)­pyridine). Of note, this is the first example of mediator-based reductive *N*-centered radical generation.

Under these conditions, *N*,*N*-dibenzyl and *N*,*N*-dimethylpyridinium amines **1aq** and **1as** engage in efficient C–N coupling to afford **3aq** and **3as** in 63 and 70% yield, respectively. Aryl bromides can also efficiently couple with secondary *N*-aminopyridinium salts providing **3ar** and **3at** in 64 and 94% yield, respectively. *N*-Pyridinium tetrahydroisoquinoline, which is comprised of an important structural motif in medicinal chemistry,^46^ undergoes C–N XEC to afford *N*-aryltetrahydroisoquinoline **3au** in 74% yield. We observe moderate to low yields for *N*-aryl aminopyridinium salts—under electrochemical XEC conditions, biaryl amines **3av** and **3aw** were accessed in 38 and 45% yield, respectively—which we speculate may be due to rapid depyridylation at the RVC surface due to π–π stacking.^47^ In these cases, the developed chemical conditions are superior (**3av**, 74% and **3aw**, 52%). Other reported *N*-centered radical precursors, **N**_**1**_–**N**_**4**_,^48–50^ did not participate in efficient coupling.

#### Late-Stage Functionalization

The developed reductive coupling is compatible with late-stage functionalization of pharmaceutically derived aryl halides and *N*-aminopyridinium salts (Figure 5). Dehydroepiandrosterone-derived aryl bromide **2ax** engages in electrochemically promoted XEC with **1ax** in 90% yield. TBS-protected 1-(4-bromophenyl)­ribose derivative **2ay** participates in C–N coupling to afford 1-arylribose (**3ay**, 44% yield), which is part of a class of molecules important for artificial base-pairs to expand the genetic alphabet.^51^ Aryl halides derived from bromantane, a central nervous system stimulant, and PD153035, a tyrosine kinase inhibitor,^52^ engage in XEC to afford **3az** and **3aaa** in 51 and 15% yield, respectively. Aziridines, which are potentially reductively labile, engage in mediated XEC to furnish **3aab** without detectable ring opening.^53,54^ *N*,*N*-Diallyl aminopyridinium salt participates in C–N XEC to afford Androgen receptor antagonist **3aac** with moderate yield.

**Figure 5:**
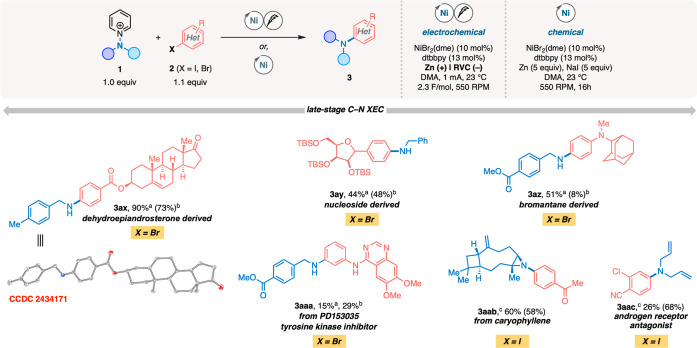
Late-stage application of C–N XEC. Electrochemical conditions: **1** (0.120 mmol), **2** (0.132 mmol), NiBr_2_(dme) (12.0 μmol), dtbbpy (15.6 μmol) in DMA (5 mL) in an undivided cell, Zn­(+)|RVC(−), 1 mA until 2.30 F/mol was passed at 23 °C at 550 rpm. ^a^LiBr (0.180 mmol) as an additive, 0.5 mA. Chemical conditions: **1** (0.100 mmol), aryl halide (0.110 mmol), NiBr_2_(dme) (10.0 μmol), dtbbpy (13.0 μmol), zinc powder (0.500 mmol), NaI (0.500 mmol) in DMA (0.5 mL) for 16 h at 23 °C at 550 rpm. ^b^Reaction run at 65 °C for 30 min ^c^bpy (13.0 μmol) as a ligand, Ni­(bpp)_2_ (5 mol %) as a mediator. Reaction yields: Isolated yield from electrochemical conditions (^1^H NMR yield from chemical conditions determined against 1,3,5-trimethoxybenzene).

### Strategic Application of C–N XEC

#### Build-and-Couple Amine Synthesis

The broad functional group tolerance and exclusive cross-selectivity enabled application of C–N XEC in build-and-couple synthesis of designer amines. Figure 6a illustrates the build-and-couple logic that enables rapid assembly of bespoke amines from readily available olefins and C–H bonds. In this paradigm, the build phase comprises olefin and C–H *N*-aminopyridylation; subsequent C–N XEC delivers targeted amines in the couple phase. Build-and-couple synthesis has been applied to synthesize norbornene-derived *N*-aryl aziridine **6a**, which is not efficient when synthesized via Ni-catalyzed photochemical^55^ and electrochemical^56^ C–N coupling from N–H aziridines. Isatin-derived C–H aminated product **6b** further demonstrates the utility of this build-and-couple paradigm where the corresponding amine is unknown.

**Figure 6:**
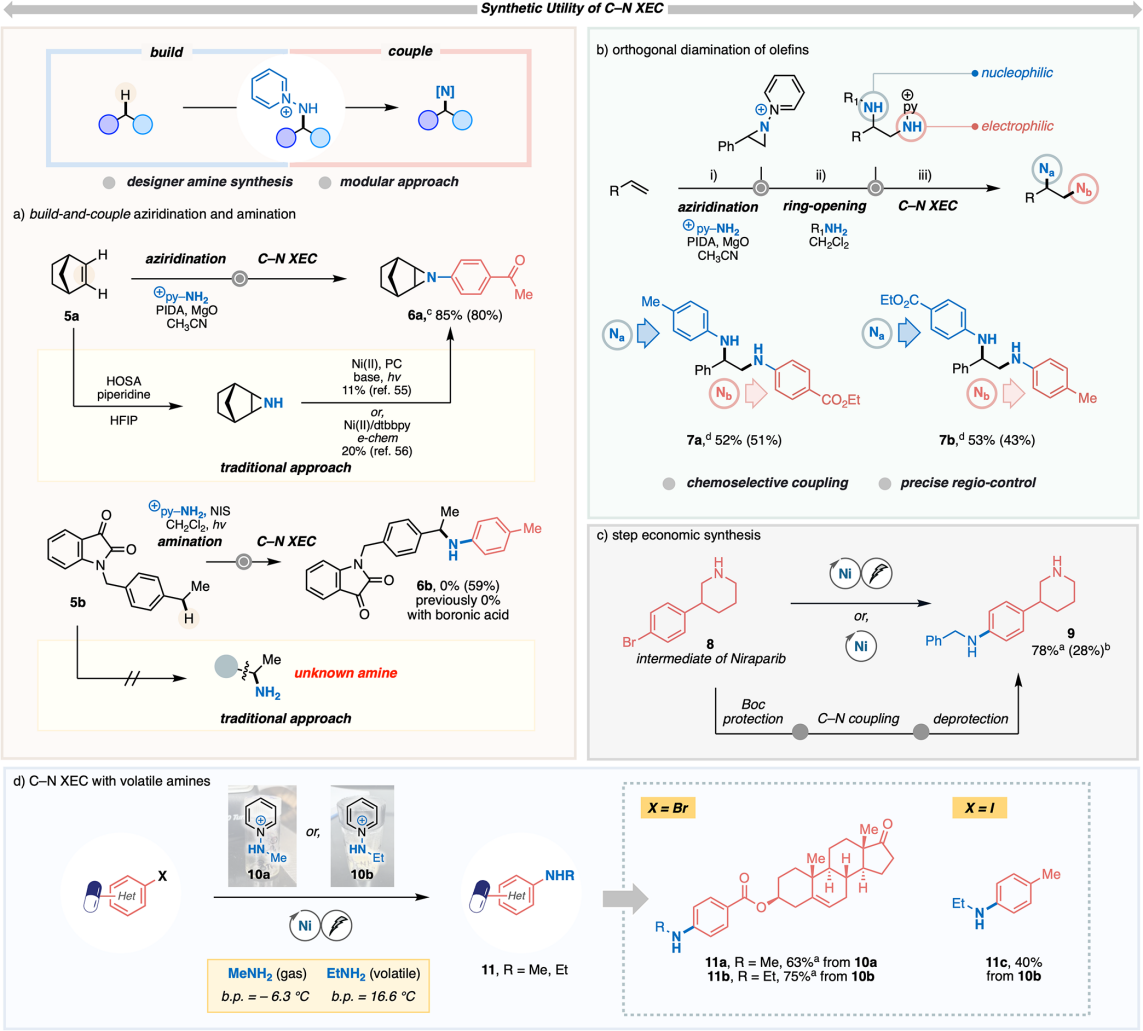
Strategic application of C–N XEC. (a) Build-and-couple synthetic logic enables rapid access of designer amines. (b) Modular unsymmetrical 1,2-diamine synthesis is enabled by sequential aziridine opening, C–N XEC. (c) Step-economic synthesis using C–N XEC without protecting the free amine center. (d) Use of volatile amines in C–N XEC. Electrochemical conditions: *N*-aminopyridinium salts (0.120 mmol), aryl halide (0.132 mmol), NiBr_2_(dme) (12.0 μmol), dtbbpy (15.6 μmol) in DMA (5 mL) in undivided cell, Zn­(+)|RVC(−), 1 mA until 2.30 F/mol was passed at 23 °C at 550 RPM. ^a^LiBr (0.180 mmol) as additive, 0.5 mA. Chemical conditions: *N*-aminopyridinium salts (0.100 mmol), aryl halide (0.110 mmol), NiBr_2_(dme) (10.0 μmol), dtbbpy (13.0 μmol), zinc powder (0.500 mmol), NaI (0.500 mmol) in DMA (0.5 mL) for 16 h at 23 °C at 550 RPM. ^b^Reaction run at 65 °C for 30 min. ^c^bpy (13.0 μmol) as ligand, Ni­(bpp)_2_ (5 mol%) as mediator. ^d^Ni­(bpp)_2_ (5 mol%) as mediator. Reaction yields: Isolated yield from electrochemical conditions (^1^H NMR yield from chemical conditions determined against 1,3,5-trimethoxybenzene).

#### Diamination of Olefins

Figure 6b illustrates the extension of build-and-couple synthetic logic to the assembly of unsymmetrical diamines, which are challenging to synthesize^57,58^ but important structural motifs in bioactive molecules and transition-metal catalysis.^59–61^ Nucleophilic ring opening of *N*-pyridinium aziridine with amines followed by regioselective Ni-catalyzed reductive coupling at the electrophilic pyridinium linchpin provides access to unsymmetrical 1,2-diamines. The modularity of this strategy enables precise control over the installation of structurally similar nitrogen-containing fragments, as highlighted in the synthesis of **7a** and **7b**.

#### Step Economic Synthesis

C–N XEC is tolerant of free N–H groups, which enabled application in the synthesis of **9**—an intermediate for anticancer medicine Niraparib—from **8** without N–H protection (Figure 6c). While we subject aryl bromide (**8**) for traditional redox-neutral Ni-catalyzed C–N coupling with nucleophilic amines,^55,56^ we observe inefficient coupling (16%) or a total lack of reactivity (0%) for the formation of **9**. This highlights the applicability of C–N XEC.

#### C–N XEC with Volatile Amines

Short-chain primary amines, such as methyl- and ethylamine, can pose challenging substrates in C–N coupling due to (1) achieving monoarylation without competing diarylation and (2) practical handling of these volatile reagents.^62^ *N*-Methyl- and *N*-ethyl aminopyridinium salts (**10a** and **10b**)—electrophilic surrogates of methyl- and ethylamine—are bench-stable oils and solids, respectively (Figure 6d). The orthogonality of reductive activation in XEC enables coupling under base-free conditions^62–64^ and prevents these reagents from engaging in diarylation. Both methyl- and ethylamine can be transferred to dehydroepiandrosterone-derived aryl bromide **2ax** in moderate yields (**11a** and **11b**). *N*-Ethyl aminopyridinium salt **10b** can also be transferred to *p*-tolyl iodide (**2a**) in 40% yield (**11c**).

### Reaction Mechanism of C–N XEC

We carried out a series of experiments designed to evaluate the origin of the observed cross-selectivity. Radical trapping experiments provide compelling evidence for the intermediacy of *N*-centered radicals during C–N XEC: Addition of 2,2,6,6-tetramethyl-1-piperidinyloxy (TEMPO, 1.0 equiv) to the reductive coupling (under either chemical or electrochemical conditions) of **1aq** and *p*-tolyl iodide completely inhibited C–N coupling (Figure 7a); addition of *N*-*tert*-butyl-α-phenylnitrone (PBN, 1.0 equiv) suppressed XEC coupling but also provided the opportunity to trap and characterize the reductively generated *N*-centered radicals (Figure 7b). The obtained EPR spectrum was consistent with trapping of the aminyl radical derived from **1aq** (a_N(PBN)_ = 14.4 G and a_H_ = 3.53 G),^65^ and the formation of **3aad** was confirmed by HR-MS analysis (calculated for [M + H]^+^ = 375.2431, observed [M + H]^+^ = 375.2423; Figure 7c). No carbon-based radicals were detected during either TEMPO- or PBN-trapping experiment.

**Figure 7:**
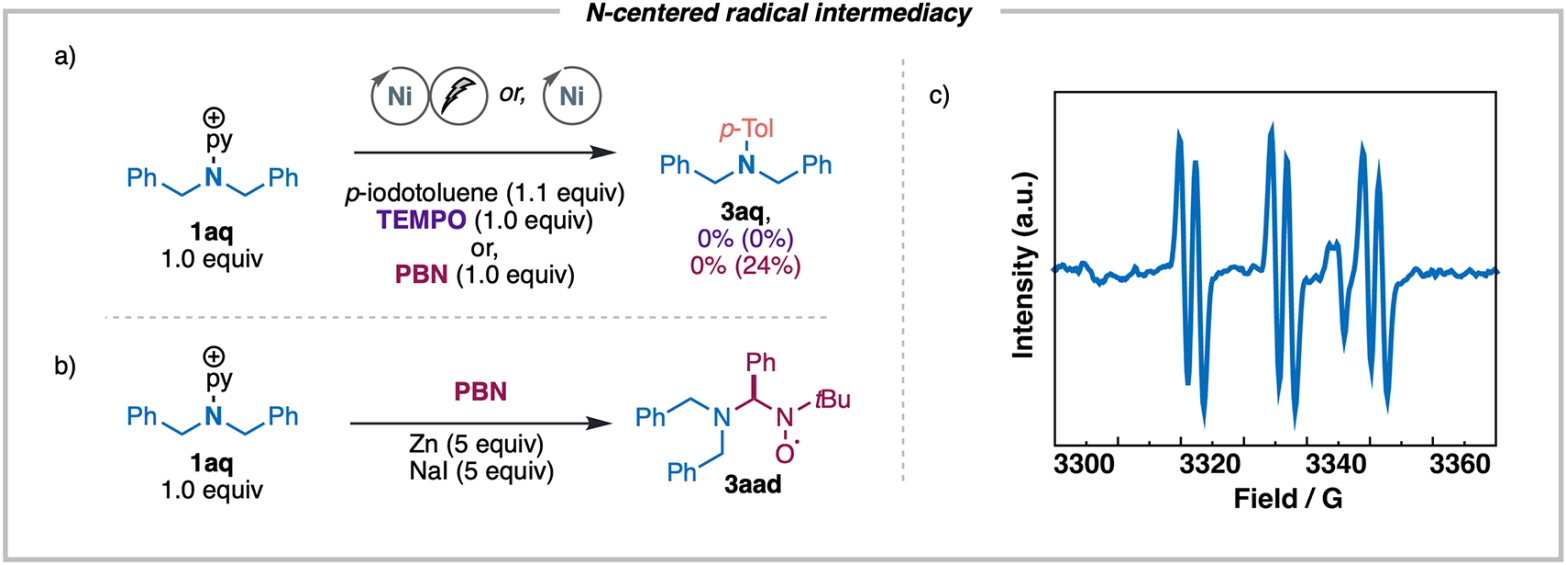
*N*-centered radical intermediacy. (a) Addition of TEMPO or PBN inhibits C–N XEC. (b) PBN trapping enables characterization of the adduct with reductively generated *N*-centered radicals. (c) EPR spectrum of PBN-trapped aminyl radical **3aad**.

Under optimized chemical or electrochemical conditions, efficient biaryl formation is observed when *N*-aminopyridinium salt **1a** is removed from the C–N XEC reaction mixture. In the presence of **1a**, we do not observe any homocoupling, even when **2a** is used in excess (see Figures S5 and S6). These observations indicate that (1) *N*-centered radical capture is kinetically competitive with oxidative addition of aryl halides and (2) *N*-centered radical intermediates divert an otherwise competent C–C coupling cycle toward C–N coupling.

The rates of consumption of **1a** and **2a** are precisely matched during C–N XEC (Figure 8), which enables the observed high chemical yield and exclusive cross-selectivity. A series of observations indicate that slower generation of *N*-centered radicals leads to undesired biaryl formation (and attenuated cross-selectivity), while faster radical generation diminishes reaction yield (without impacting cross-selectivity). First, reactions run at 5 mA and 10 mA produce 56 and 21% yield, respectively, indicating that faster generation of *N*-centered radicals results in less efficient coupling. Second, to match the slower rate of oxidative addition of *p*-tolyl bromide as compared to *p*-tolyl iodide, lowering the current from 1 mA to 0.5 mA increased the reaction efficiency from 44 to 61%. Third, Ar–Ar homocoupling is observed for electron-deficient aryl iodides due to their faster rate for oxidative addition with low-valent nickel complexes (e.g., 16:1 selectivity instead of exclusive C–N coupling in the case of **3e**). Fourth, catalyst loading has a notable impact on **2a** activation. Higher catalyst loading (>10 mol %) results in poor cross-selectivity due to enhanced activation of **2a** via bimolecular oxidative addition of aryl halide with low-valent nickel (see the Supporting Information, Section E.6).^66^

**Figure 8:**
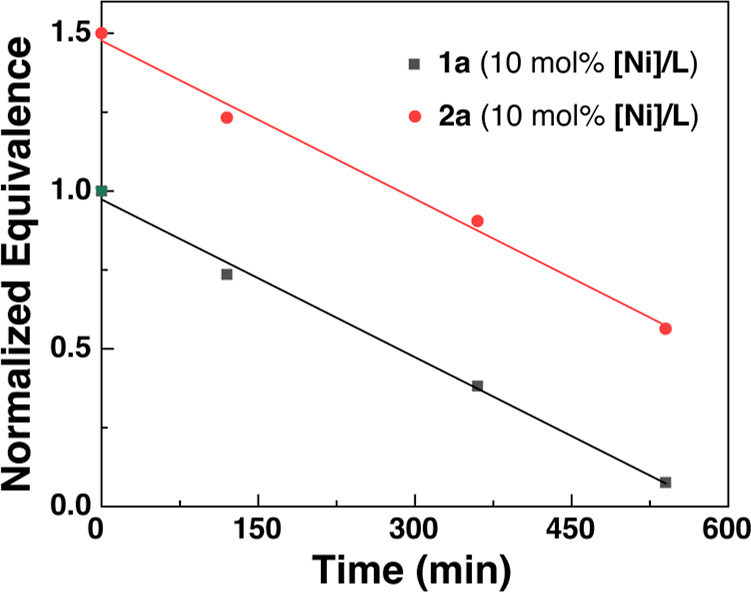
Relative rates of consumption of *N*-aminopyridinium salts and aryl iodides at 10 mol % catalyst loading.

To gain insight into the catalyst resting state, we monitored the coupling of **1a** and 1-bromo-4-(trifluoromethyl)­benzene (**2e′**) by ^19^F NMR (Figure 9). During early reaction times (between 0.10 and 0.15 F passed), we observe the formation of a Ni­(II)­ArBr (δ = −61.66 ppm) (see the Supporting Information, Section E.7. for more details). This species is consumed with concurrent formation of a new compound (0.30 F passed) which we assign as a Ni­(II)–aryl-amido complex^67^ (δ = −62.03 ppm). This new species accounts for ∼80% of the Ni loading and persists during catalysis. Addition of I_2_/LiI quenches the ^19^F signal at −62.03 and results in the formation of 1-iodo-4-(trifluoromethyl)­benzene (δ = −63.13 ppm), which is consistent with formulation of the observed intermediate as a Ni­(II)–monoaryl species.^68–72^ Similar observations are made when presynthesized (dtbbpy)­NiArBr is used as a catalyst (Ar = 4-CF_3_–C_6_H_4_−): concurrent disappearance of Ni­(II)­ArBr peak (−61.66 ppm) and appearance of the putative Ni­(II)–aryl-amido intermediate (δ = −62.03 ppm) and **3e** (δ = −60.67 ppm), without any homocoupling (see Figure S11). Formation of **3e** at very early reaction times (from 0.05 F passed) when (dtbbpy)­NiArBr is used as a catalyst further validates that the initiation stage proceeds through the Ni­(II)­ArBr intermediate (see Figure S13).

**Figure 9:**
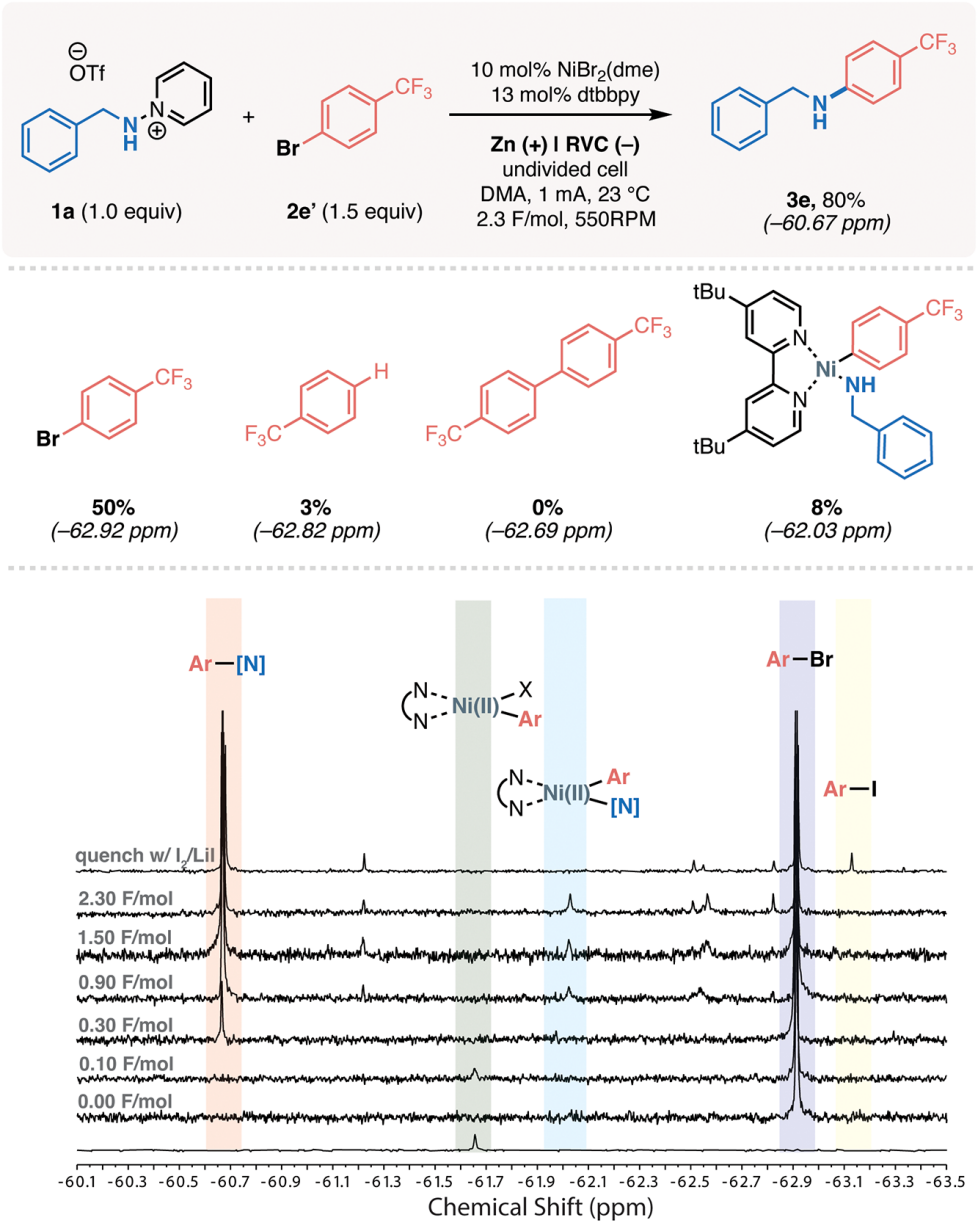
A time-course study of C–N XEC by ^19^F NMR to postulate the Ni­(II)–aryl-amido complex as a catalytic intermediate.

We carried out additional experiments to evaluate the catalyst oxidation states accessed during C–N XEC (Figure 10). CV measurements made during titration of Ni­(dtbbpy)­Br_2_ with *p*-iodotoluene (**2a**) indicate the disappearance of the peak for the Ni­(I)/Ni­(II) wave, suggesting rapid oxidative addition to Ni­(I) (Figure 10a). This observation is analogous to those made by Baran and attributed to a Ni­(I)/Ni­(III) cycle.^73^ Finally, replacing NiBr_2_(dme) with Ni­(COD)_2_ results in inefficient coupling (COD = 1,4-cyclooctadiene, Figure 10b).

**Figure 10:**
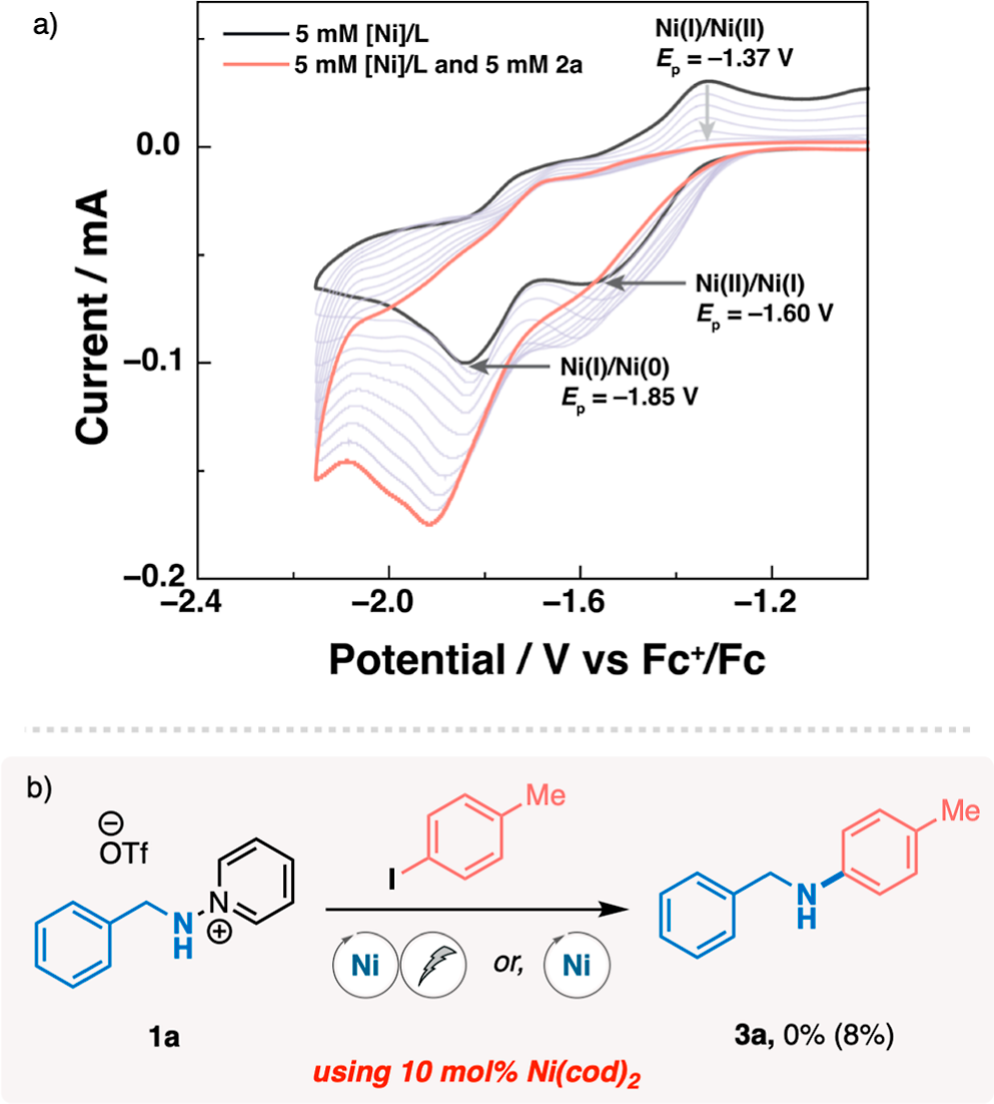
Evidence for a plausible Ni­(I)/Ni­(III) catalytic system for C–N XEC. (a) Quenching of the N­(II)/Ni­(II) redox couple by aryl iodide indicates oxidative addition of the Ni­(I) intermediate. (b) Unproductive outcomes using the Ni(0) precatalyst suggests the nonoperational Ni(0)/Ni­(II) pathway.

Together, the assembled mechanistic data support the proposed catalytic C–N XEC mechanism illustrated in Figure 11. Reduction of the Ni­(II) precatalyst generates Ni­(I) intermediate **A**, which engages in rapid oxidative addition to form Ni­(III)–aryl intermediate **B**. One-electron reduction then generates Ni­(II)­ArBr complex **C**, which we observe at early times during our ^19^F NMR time-course study. Instead of transmetalation from **C** leading to homocoupling,^68,74,75^ **C** rapidly traps *N*-centered radicals generated via concomitant reduction of *N*-pyridinium salts to afford Ni­(III)–aryl-amido intermediate **D**. **D** is rapidly reduced under reductive condition to generate catalyst resting state **E**.^67^ Resting state **E** subsequently traps an *N*-centered radical to generate Ni­(III) intermediate **F**, which engages in rapid C–N reductive elimination to afford the observed XEC products and Ni­(I)–amido **G**. Oxidative addition of **G** with aryl halide regenerates Ni­(III)–aryl-amido intermediate **D** to close the catalytic cycle. The active catalytic cycle avoids Ni­(II)­ArBr intermediates, which are often the origin of C–C homocoupling, and accounts for the excellent Faradaic efficiency of the developed C–N XEC (87%).

**Figure 11:**
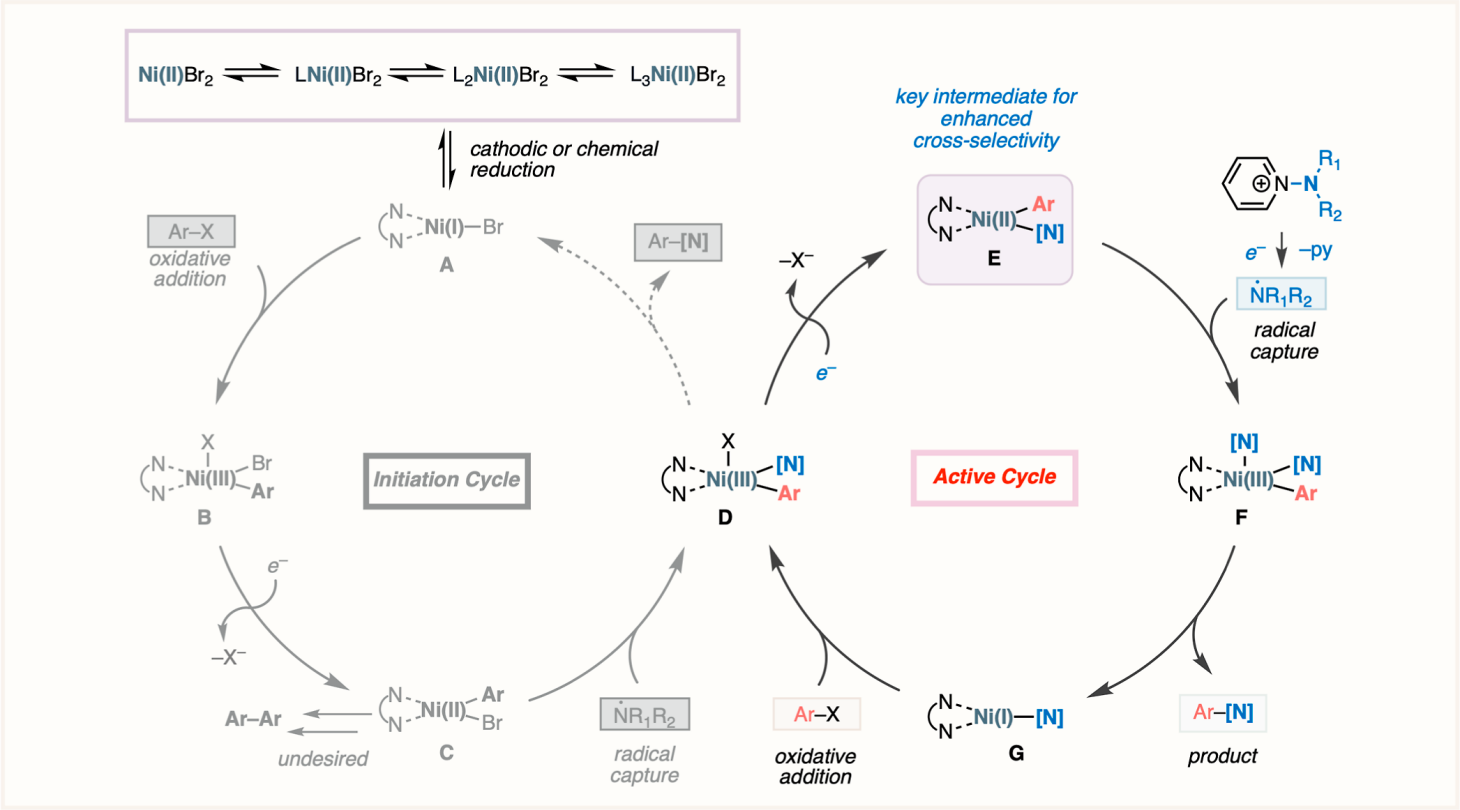
Proposed C–N XEC mechanism that accounts for exclusive cross-selectivity.

### Native Amine Functionalization

The synthetic applications described above leverage the ability to install *N*-aminopyridinium handles in organic small molecules via C–H activation or olefin aziridination. Motivated by the potential to apply C–N XEC chemistry to the elaboration of amines, we sought to identify strategies to engage native amine functional groups directly.

Guided by the critical importance of *N*-centered radicals in the developed coupling, we envisioned using *N*-phenothiazinium intermediates, which could be prepared by oxidative coupling of amines and phenothiazine, to prepare alternate radical precursors. To this end, treatment of amines with *N*-bromosuccinimide (NBS) in the presence of *N*-methylphenothiazine provides access to *N*-phenothiazinium intermediates,^76^ which engage in subsequent C–N XEC under slightly modified conditions to those developed for XEC with *N*-aminopyridinium salts (Figure 12). Piperidine and adamantyl amine-derived *N*-phenothiazinium salts showcase good yields in this format (**14a**, 89% and **14b**, 68%). Coupling with aryl bromide is also efficient to provide **14b** in 57% yield. Late-stage amine arylation can be accomplished from desloratadine-derived *N*-phenothiazinium to produce **14c** in 53% yield. Installation of phenothiazine to piperidine followed by C–N coupling in a one-pot condition delivers 54% product formation, which highlights the practicality of this approach.

**Figure 12:**
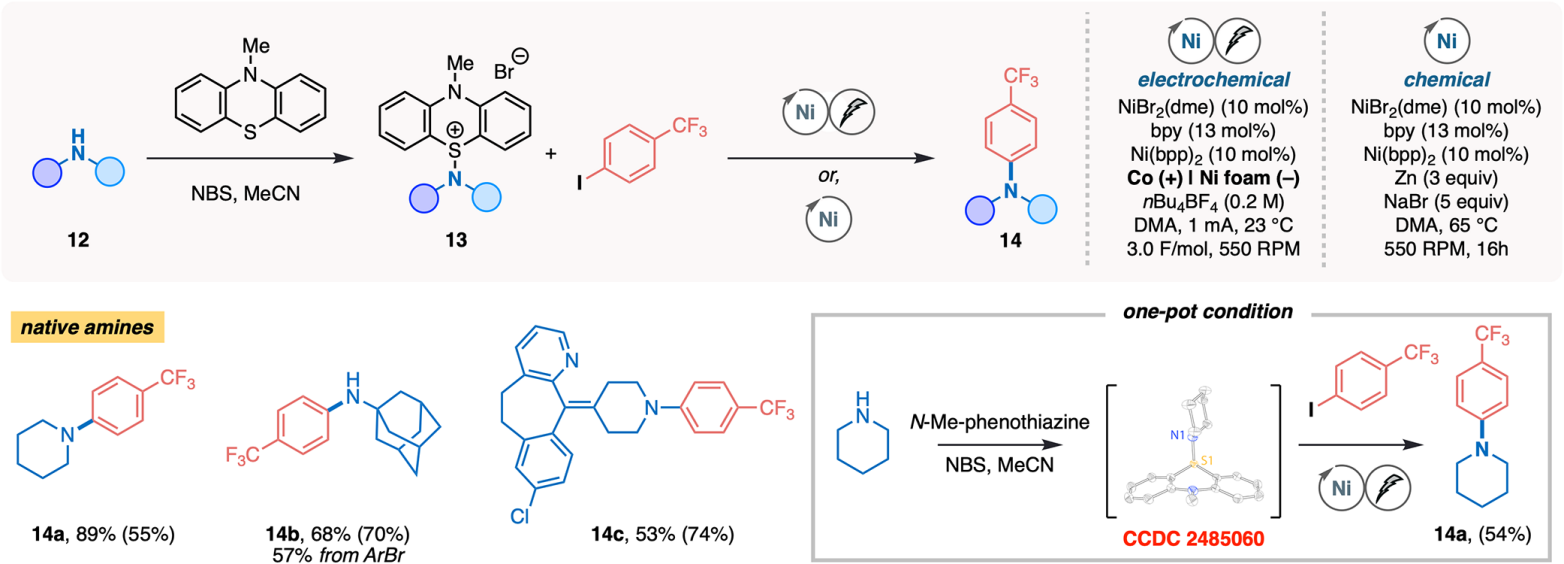
Application of C–N XEC in native amine functionalization. Native amine functionality engages in C–N XEC via a two-step procedure comprised of *N*-phenothiazination followed by C–N XEC. This protocol can be accomplished in one pot and takes advantage of traceless installation of an *N*-phenothiazinium linchpin to activate amines as *N*-centered radical precursors. Reaction yields: Isolated yield from electrochemical conditions (^1^H NMR yield from chemical conditions determined against 1,3,5-trimethoxybenzene).

## Conclusions

In summary, we report a base-free C–N XEC coupling that proceeds under mild conditions that enable broad functional group tolerance, including base-sensitive substrates and late-stage applications. Strategic application of reductive C–N XEC enables rapid, modular synthesis of designer amines—including unsymmetrical 1,2-diamines—via novel retrosynthetic disconnections. Additionally, native amines can be incorporated by traceless installation of a phenothiazinium linchpin, further enhancing the versatility of the methodology. C–N XEC proceeds with exclusive cross-selectivity. Mechanistic studies indicate that the observed selectivity arises from *N*-centered radical capture by a Ni­(II)–aryl-amido intermediate. Together, this philicity-mismatched coupling provides complementary tools for C–N bond construction, enables base-free C–N coupling by exploiting an orthogonal mode of amine activation, and introduces a new mechanism in XEC catalysis.

## Supplementary Material



## Notes

Experimental procedures, spectral data, and diffraction data (PDF)
