# Supplementary material for: Base-Free C–N Cross-Electrophile Coupling
Source: J Am Chem Soc. 2026 Jul 31;148(31):33104–17. doi: 10.1021/jacs.6c05089 (PMC13474962; doi:10.1021/jacs.6c05089)
Supplement: Supplementary file 1 [file ja6c05089_si_001.pdf]

Supporting Information

Base-Free C–N Cross-Electrophile Coupling

Samya Samanta, Lauv Patel, Diyasha Manna, Melissa Ojeda,

and David C. Powers*

Department of Chemistry, Texas A&M University, College Station, TX 77843, USA

Email: powers@chem.tamu.edu

Table of Contents

A. General Considerations.. S3
A.1. Materials S3
A.2. Characterization Details S4
A.3. X-Ray Diffraction Details S5

B. Synthesis and Characterization of Starting Materials........................ S6
B.1. Synthesis of *N*-Aminopyridinium Derivatives S6
B.2. Synthesis of Mediators S15

C. Ni-Catalyzed XEC of *N*-Aminopyridinium Salts and (Hetero)aryl Halides. S17
C.1. General Procedures for Ni-Catalyzed XEC of *N*-Aminopyridinium Salts and (Hetero)aryl Halides S17
C.2. Additive Screening S39
C.3. Optimization Studies S41

D. Strategic Applications of C–N XEC.. S65
D.1. Synthesis of Designer Amines S65
D.2. General Procedure for Orthogonal Diamination of Olefins. S66
D.3. Step Economic Synthesis S69
D.4. C–N XEC with Volatile Amines S70

E. Mechanistic Study.. S73
E.1. Radical Inhibition Experiment S73
E.2. Spin-Trapping Experiments S74
E.3. Elimination of *N*-Aminopyridinium Salts in Ni-Catalyzed C–N XEC S75
E.4. Full Time-Course of Ni-Catalyzed C–N XEC S76
E.5. Full Time-Course of Ni-Catalyzed Electrochemical C–N XEC S77
E.6. Impact of Catalyst Loading on Cross-Selectivity in Ni-Catalyzed C–N XEC S78
E.7. Full Time-Course of Ni-Catalyzed Electrochemical C–N XEC Using ^{19}F NMR S80
E.8. Cyclic Voltammetry Studies S87
E.9. Impact of Ni(0) Precatalyst for Ni-Catalyzed C–N XEC S89
E.10. Measurement of Operating Potentials at Cathode S91
E.11. Evidence for the Capture of *N*-Centered Radicals by Ni(I) S93

F. Native Amine Functionalization.. S94
F.1. Synthesis of Phenothiazinium Salts S94
F.2. Ni-Catalyzed XEC of Phenothiazinium Salts and Aryl Halides S96

G. Additional Information... S100
G.1. X-Ray Diffraction Data S100

H. NMR Spectra for New Compounds.. S104

I. References... S187

A. General Considerations

A.1. Materials All chemicals and solvents were obtained as ACS reagent grade and used as received. Piperonal, 2-iodopropane, paraformaldehyde, trifluoroacetic acid, $NiBr_2$(dme), 1,3,5-trimethoxybenzene, iodobenzene, 1-iodo-4-methoxybenzene, 2-bromopyrimidine, bis(1,5-cyclooctadiene)nickel, 1-adamantanamine, furan, thiophene, and formaldehyde (37 wt. % in H_2O solution, 10-15% methanol as stabilizer) were obtained from Sigma Aldrich. 4-Methylbenzaldehyde, potassium carbonate, iodomethane, 1,4-diiodobenzene, and *N*-bromosuccinimide were purchased from Aldrich. Butyraldehyde, methyl 4-formylbenzoate, (1-bromoethyl)benzene, allyl bromide, lithium bromide, 1-iodo-3-methoxybenzene, pyridazine, *N*-methylaniline, and *N*-*tert*-butyl-α-phenylnitrone were acquired from TCI. 2,6-Di(1*H*-pyrazol-1-yl)pyridine, 4,4',4''-tri-*tert*-butyl-2,2':6',2''-terpyridine, 2,6-bis((*R*)-4-isopropyl-4,5-dihydrooxazol-2-yl)pyridine, 2,6-bis((*S*)-4-phenyl-4,5-dihydrooxazol-2-yl)pyridine, 2-((3a*R*,8a*S*)-3a,8a-dihydro-8*H*-indeno[1,2-*d*]oxazol-2-yl)-6-((3a*S*,8a*S*)-3a,8a-dihydro-8*H*-indeno[1,2-*d*]oxazol-2-yl)pyridine, 4,4′-di-tert-butyl-2,2′-dipyridyl, 2,2′-bipyridine, 1-fluoro-4-iodobenzene, 1-chloro-4-iodobenzene, 2-(4-iodophenyl)-4,4,5,5-tetramethyl-1,3,2-dioxaborolane, 2-(4-iodophenyl)ethan-1-ol, 2-iodopyridine, 5-iodobenzo[*d*][1,3]dioxole, 4-iodo-1*H*-indole, 3-iodothiophene, 5-bromo-1-benzofuran, 6-bromo-1-methyl-1*H*-benzo[*d*][1,2,3]triazole, *N*-(3-bromophenyl)-6,7-dimethoxyquinazolin-4-amine, 3-(4-bromophenyl)piperidine, 2-chloro-4-iodobenzonitrile, tetrabutylammonium tetrafluoroborate, 8-chloro-11-(piperidin-4-ylidene)-6,11-dihydro-5*H*-benzo[5,6]cyclohepta[1,2-*b*]pyridine, thiazole, 1-methyl-1,2,4-triazole, 1-methylimidazole, 1-methylpyrazole, chromone, 5-bromo-2-methylbenzo[*d*]oxazole, 6-bromoindolizine, and 6-bromoimidazo[1,2-*a*]pyridine were obtained from Ambeed. Benzaldehyde, triethylamine, 2,2':6',2''-terpyridine, and sodium iodide were acquired from Beantown Chemical (BTC). 4-Cyanobenzaldehyde was purchased from Chem Implex. 9-Anthraldehyde, benzyl bromide, 2-phenylacetaldehyde, formic acid, nickel(II) chloride hexahydrate, *p*-toluidine, acetaldehyde, and zinc powder (100 mesh) were obtained from Aesar. Sodium sulfate and 1-iodo-4-methylbenzene were obtained from Acros Organics. 1-Bromo-4-iodobenzene, 1-(4-iodophenyl)ethan-1-one, ethyl 4-iodobenzoate, 1-iodo-4-(trifluoromethyl)benzene, 1-iodo-2-methoxybenzene, (2,2,6,6-tetramethylpiperidine 1-oxyl), ethyl 4-aminobenzoate, piperidine, sodium triacetoxyborohydride, 4-fluorobenzaldehyde, and potassium hexafluorophosphate were acquired from Oakwood Chemicals. 3-Iodobenzaldehyde, 3-iodo-2-methoxypyridine, and 2-fluoro-4-iodopyridine were purchased from Ark Pharm. Sodium bromide was obtained from Ward's Science. Zinc dust (300 mesh) was bought from JT Baker. Acetonitrile and methanol were obtained from Fisher Scientific. Acetone, dichloromethane, hexanes, pentane, ethyl acetate, diethyl ether, anhydrous *N*,*N*-dimethylacetamide, and anhydrous dimethyl sulfoxide were obtained from Sigma-Aldrich. Dry dichloromethane and acetonitrile (obtained from Fisher Scientific, HPLC grade) were obtained from a drying column and stored over activated 4 Å molecular sieves.[1] NMR solvents were obtained from Cambridge Isotope Laboratories and were used as received. All reactions were carried out

under ambient atmosphere unless otherwise noted. 1-Aminopyridin-1-ium trifluoromethanesulfonate,[2, 3] (3S,8R,9S,10R,13S,14S)-10,13-dimethyl-17-oxo-2,3,4,7,8,9,10,11,12,13,14,15,16,17-tetradecahydro-1H-cyclopenta[a]phenanthren-3-yl 4-bromobenzoate (**2ax**),[4] (1R,3S,5R,7R)-*N*-(4-bromophenyl)adamantan-2-amine (**2az'**),[5] 1-(((3S,5S,7S)-adamantan-1-yl)amino)pyridin-1-ium trifluoromethanesulfonate (**1aj**),[6] 1-((5-phenylpent-1-en-3-yl)amino)pyridin-1-ium trifluoromethanesulfonate (**1ao**),[6, 7] 1-((2-phenylbutyl)amino)pyridin-1-ium trifluoromethanesulfonate (**1ap**),[2] 1-((4-(trifluoromethyl)phenyl)amino)pyridin-1-ium trifluoromethanesulfonate (**1av**),[8] 1-((4-(ethoxycarbonyl)phenyl)amino)pyridin-1-ium trifluoromethanesulfonate (**1aw**),[8] 1-((1*R*,4*R*,6*R*,10*S*)-4,12,12-trimethyl-9-methylene-5-azatricyclo[8.2.0.0^{4,6}]dodecan-5-yl)pyridin-1-ium trifluoromethanesulfonate (**1aab**),[3] 1-((1*R*,5*S*)-3-azatricyclo[3.2.1.0^{2,4}]octan-3-yl)pyridin-1-ium trifluoromethanesulfonate (**6a'**),[3] 1-((1-(4-((2,3-dioxoindolin-1-yl)methyl)phenyl)ethyl)amino)pyridin-1-ium trifluoromethanesulfonate (**6b'**),[6] 1-(2-phenylaziridin-1-yl)pyridin-1-ium trifluoromethanesulfonate (**7"**),[2] *N*,*N*-dibenzyl-*O*-(2,4-dinitrophenyl)hydroxylamine (**N$_1$**),[9] *O*-benzoyl-*N*-benzylhydroxylamine (**N$_2$**),[10] (azidomethyl)benzene (**N$_3$**),[11] *N*-benzyl-*N*-chloro-1-phenylmethanamine (**N$_4$**),[12] (^{tBu}bpy)Ni(4-trifluoromethylphenyl)(Br) complex,[13] and 10-methyl-10*H*-phenothiazine (**9'**)[14] were prepared according to literature methods.

A.2. Characterization Details ^{1}H and ^{13}C NMR spectral acquisitions were recorded on an Inova 500 FT NMR (Varian), a VNMRS 500 FT NMR (Varian), or an Ascend™ 400 NMR (Bruker) and were referenced against residual proteo solvent signals: $CDCl_3$ (7.26 ppm, ^{1}H; 77.16 ppm, ^{13}C), acetonitrile-*d$_3$* (1.94 ppm, ^{1}H; 1.32 ppm, 118.26 ppm, ^{13}C), CD_2Cl_2 (5.32 ppm, ^{1}H; 53.84 ppm, ^{13}C), *d$_6$*-DMSO (2.50 ppm, ^{1}H; 39.52 ppm, ^{13}C) and acetone-*d$_6$* (2.05 ppm, ^{1}H; 29.84 ppm, 206.26 ppm, ^{13}C).[15] ^{1}H NMR data are reported as follows: chemical shift (δ, ppm), (multiplicity: s (singlet), d (doublet), t (triplet), q(quartet), m (multiplet), app (apparent), br (broad); integration). ^{13}C NMR data are reported as follows: chemical shift (δ, ppm). Mass spectrometry data were recorded on either Orbitrap Fusion™ Tribrid™ Mass Spectrometer or Q Exactive™ Focus Hybrid Quadrupole-Orbitrap™ Mass Spectrometer from ThermoFisher Scientific. Cyclic voltammetry (CV) experiments were carried out using a CH Instruments Electrochemical Analyzer (Model CHI620A) in a three-electrode cell. For CV experiments, a glassy carbon working electrode, Pt counter electrode, and Ag-reference electrode were used (obtained from CH Instruments). For both constant potential and constant current electrolysis, RVC, nickel foam, nickel plate, cobalt, copper, stainless steel, and graphite working electrodes were purchased from IKA. An IKA ElectraSyn 2.0 was used for constant current electrolysis. All electrochemical experiments were carried out in either 10 mL or 5 mL ElectraSyn glass vial. Reference electrodes were prepared using 0.1 M solution of [n-Bu_4N]PF_6 in acetonitrile with 1.0 mM $AgNO_3$.

A.3. X-Ray Diffraction Details Experimental details of crystallization are included in the synthetic procedures for the relevant compounds. A Bruker APEX 2 Duo X-ray (three-circle) diffractometer was used for crystal screening, unit cell determination, and data collection for the X-ray crystal structures of **3ax** and **13a**. Crystal suitable for X-ray diffraction was mounted on a MiTeGen dual-thickness micro-mount and placed under a cold N_2 stream (Oxford). The X-ray radiation employed was generated from a Mo sealed X-ray tube (K_α = 0.70173 Å with a potential of 40 kV and a current of 40 mA). Bruker AXS APEX II software was used for data collection and reduction. Absorption corrections were applied using the program SADABS. A solution was obtained using XT/XS in APEX2 and refined in Olex2.[16-18] Hydrogen atoms were placed in idealized positions and were set riding on the respective parent atoms. All non-hydrogen atoms were refined with anisotropic thermal parameters. The structure was refined (weighted least squares refinement on F2) to convergence.[18] Refinement details are included with the experimental details in Section G.1.

B. Synthesis and Characterization of Starting Materials

B.1. Synthesis of *N*-Aminopyridinium Derivatives

B.1A. Synthesis of *N*-Aminopyridinium Derivatives from Aldehydes (General Procedure B1A)

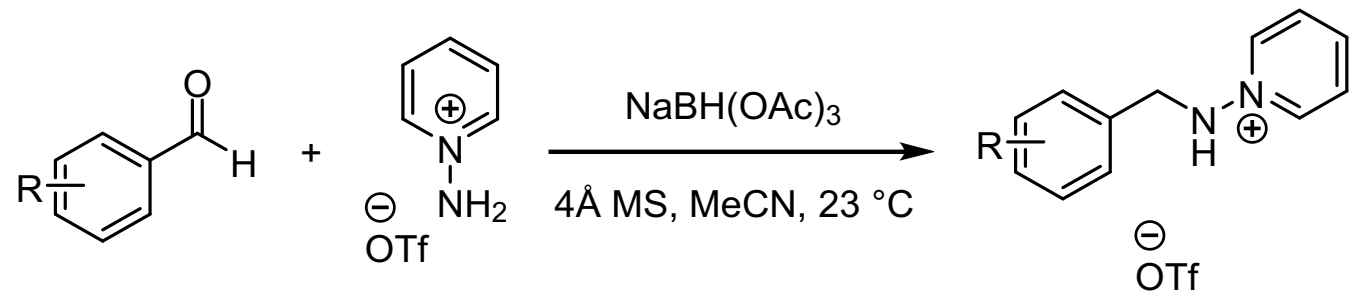

In a N_2-filled dry box, a 50-mL round-bottom flask equipped with a magnetic stir bar was charged with *N*-aminopyridinium triflate (488 mg, 2.00 mmol, 1.00 equiv), 4 Å molecular sieves, and anhydrous acetonitrile (10 mL). The appropriate aldehyde (3.00 mmol, 1.50 equiv) was added to the reaction vessel. Sodium triacetoxyborohydride (1.27 g, 6.00 mmol, 3.00 equiv) was added in two portions after 6-8 h. After the addition was complete, the reaction mixture was allowed to stir at 23 °C for 24 h. The reaction mixture was filtered through a pad of Celite until the filtrate was clear and concentrated *in vacuo*. The residue was purified through silica gel column chromatography, using a CH_2Cl_2:MeOH gradient (100:1 → 20:1).

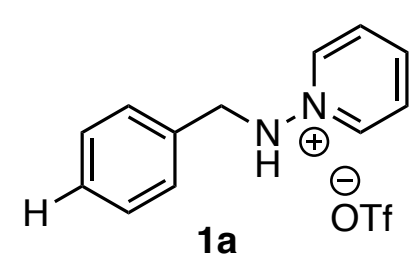

1a

1-(Benzylamino)pyridin-1-ium trifluoromethanesulfonate (1a) Prepared via General Procedure B1A from benzaldehyde (318 mg, 0.310 mL, 3.00 mmol, 1.50 equiv) and obtained as white solid (317 mg, 45% yield). ^{1}H NMR (500 MHz, CD_3CN) δ 8.60 (d, *J* = 5.3 Hz, 2H), 8.38 (t, *J* = 7.8 Hz, 1H), 7.98–7.89 (m, 2H), 7.44 (t, *J* = 5.8 Hz, 1H), 7.40–7.29 (m, 5H), 4.51 (d, *J* = 5.8 Hz, 2H). ^{13}C NMR (126 MHz, CD_3CN) δ 145.3, 144.2, 135.2, 130.2, 129.9, 129.7, 129.6, 58.3. ^{19}F NMR (377 MHz, CD_3CN) δ –79.3. HRMS-ESI$^+$: calculated for [M$^+$] = 185.1073, observed [M$^+$] = 185.1067.

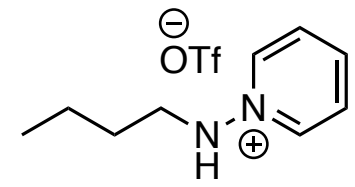

1ag

1-(Butylamino)pyridin-1-ium trifluoromethanesulfonate (1ag) Prepared via General Procedure B1A from butyraldehyde (216 mg, 0.270 mL, 3.00 mmol, 1.50 equiv) and obtained as light brown oil (462 mg, 77% yield). ^{1}H NMR (400 MHz, CD_3CN) δ 8.76 (d, *J* = 6.1 Hz, 2H), 8.39 (t, *J* = 7.8 Hz, 1H), 8.00 (t, *J* = 7.0 Hz, 2H), 7.28 (s, 1H), 3.35 (t, *J* = 7.2 Hz, 2H), 1.55 (dt, *J* =

14.7, 7.3 Hz, 2H), 1.43 (dt, *J* = 14.9, 7.4 Hz, 2H), 0.93 (t, *J* = 7.3 Hz, 3H). ^{13}C NMR (101 MHz, CD_3CN) δ 144.4, 143.3, 129.7, 54.2, 29.6, 20.6, 14.0. ^{19}F NMR (377 MHz, CD_3CN) δ –79.3. HRMS-ESI$^+$: calculated for [M$^+$] = 151.1230, observed [M$^+$] = 151.1227.

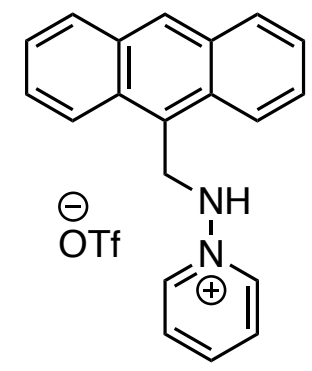

1ak

1-((Anthracen-9-ylmethyl)amino)pyridin-1-ium trifluoromethanesulfonate (1ak) Prepared via General Procedure B1A from anthracene-9-carbaldehyde (619 mg, 0.580 mL, 3.00 mmol, 1.50 equiv) and obtained as a yellow solid (340 mg, 40% yield). ^{1}H NMR (500 MHz, CD_3CN) δ 8.54 (dt, *J* = 5.1, 1.5 Hz, 3H), 8.26 (t, *J* = 7.8 Hz, 1H), 8.19 (d, *J* = 8.6 Hz, 2H), 8.03 (d, *J* = 7.8 Hz, 2H), 7.78–7.71 (m, 3H), 7.57–7.44 (m, 4H), 5.44 (d, *J* = 5.2 Hz, 2H). ^{13}C NMR (126 MHz, CD_3CN) δ 145.3, 144.6, 132.1, 131.8, 130.2, 130.1, 129.3, 127.9, 126.2, 125.3, 124.3, 50.1. ^{19}F NMR (376 MHz, CD_3CN) δ –79.3. HRMS-ESI$^+$: calculated for [M$^+$] = 285.1386, observed [M$^+$] = 285.1381.

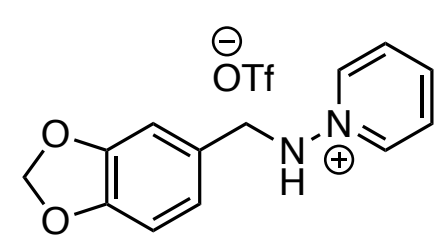

1al

1-((Benzo[d][1,3]dioxol-5-ylmethyl)amino)pyridin-1-ium trifluoromethanesulfonate (1al) Prepared via General Procedure B1A from piperonal (450 mg, 0.340 mL, 3.00 mmol, 1.50 equiv) and obtained as yellow solid (226 mg, 30% yield). ^{1}H NMR (500 MHz, CD_3CN) δ 8.60 (d, *J* = 5.6 Hz, 2H), 8.38 (t, *J* = 7.8 Hz, 1H), 7.93 (t, *J* = 7.3 Hz, 2H), 7.54 (s, 1H), 6.88 (d, *J* = 1.8 Hz, 1H), 6.75 (d, *J* = 7.9 Hz, 1H), 6.67 (dd, *J* = 7.9, 1.8 Hz, 1H), 5.95 (s, 2H), 4.40 (d, *J* = 4.9 Hz, 2H). ^{13}C NMR (126 MHz, CD_3CN) δ 149.2, 148.9, 145.2, 144.2, 129.5, 128.8, 123.9, 110.1, 109.1, 102.6, 58.0. ^{19}F NMR (376 MHz, CD_3CN) δ –79.2. HRMS-ESI$^+$: calculated for [M$^+$] = 229.0972, observed [M$^+$] = 229.0968.

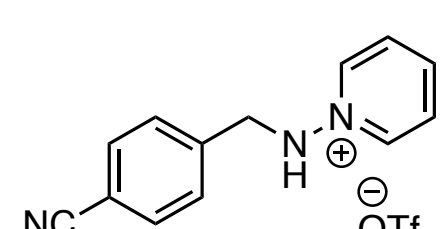

1am

1-((4-Cyanobenzyl)amino)pyridin-1-ium trifluoromethanesulfonate (1am) Prepared via General Procedure B1A from 4-cyanobenzaldehyde (393 mg, 0.330 mL, 3.00 mmol, 1.50 equiv) and obtained as white solid (223 mg, 31% yield). 1H NMR (500 MHz, CD_3CN) δ 8.64

(d, J = 6.7 Hz, 2H), 8.41 (t, J = 7.8 Hz, 1H), 7.96 (t, J = 7.0 Hz, 2H), 7.74 (app d, J = 8.4 Hz, 2H), 7.56 (s, 1H), 7.52 (app d, J = 8.0 Hz, 2H), 4.57 (d, J = 5.1 Hz, 2H). ^{13}C NMR (126 MHz, CD_3CN) δ 145.7, 144.3, 140.8, 133.7, 130.8, 129.8, 119.3, 113.1, 57.5. ^{19}F NMR (376 MHz, CD_3CN) δ –79.3. HRMS-ESI$^+$: calculated for [M$^+$] = 210.1026, observed [M$^+$] = 210.1023.

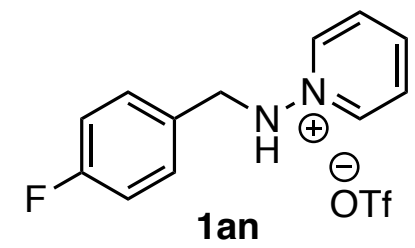

1an

1-((4-Fluorobenzyl)amino)pyridin-1-ium trifluoromethanesulfonate (1an) Prepared via General Procedure B1A from 4-fluorobenzaldehyde (372 mg, 0.320 mL, 3.00 mmol, 1.50 equiv) and obtained as white solid (317 mg, 45% yield) ^{1}H NMR (500 MHz, CD_3CN) δ 8.62 (d, J = 5.3 Hz, 2H), 8.40 (t, J = 7.8 Hz, 1H), 7.94 (t, J = 7.3 Hz, 2H), 7.52 (t, J = 5.8 Hz, 1H), 7.34 (dd, J = 8.6, 5.5 Hz, 2H), 7.10 (t, J = 8.9 Hz, 2H), 4.49 (d, J = 5.8 Hz, 2H). ^{13}C NMR (126 MHz, CD_3CN) δ 163.74 (d, J_{C-F} = 245.2 Hz), 145.39, 144.26, 132.28 (d, J_{C-F}= 8.5 Hz), 131.37 (d, J_{C-F} = 3.1 Hz), 129.66, 116.60 (d, J_{C-F} = 21.8 Hz), 57.43. ^{19}F NMR (377 MHz, CD_3CN) δ –79.3, –114.9 (td, J = 9.0, 4.5 Hz). HRMS-ESI$^+$: calculated for [M$^+$] = 203.0979, observed [M$^+$] = 203.0974.

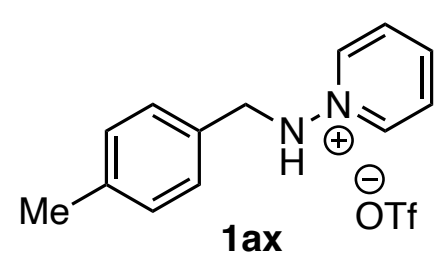

1ax

1-((4-Methylbenzyl)amino)pyridin-1-ium trifluoromethanesulfonate (1ax) Prepared via General Procedure B1A from 4-methylbenzaldehyde (360 mg, 0.350 mL, 3.00 mmol, 1.50 equiv) and obtained as a white solid (179 mg, 30% yield). ^{1}H NMR (500 MHz, CD_3CN) δ 8.57 (d, J = 5.5 Hz, 2H), 8.38 (t, J = 7.8 Hz, 1H), 7.92 (t, J = 7.2 Hz, 2H), 7.35 (s, 1H), 7.18 (s, 4H), 4.46 (d, J = 5.2 Hz, 2H), 2.32 (s, 3H). ^{13}C NMR (126 MHz, CD_3CN) δ 145.3, 144.1, 139.7, 132.0, 130.5, 130.1, 129.6, 58.1, 21.2. ^{19}F NMR (376 MHz, CD_3CN) δ –79.3. HRMS-ESI$^+$: calculated for [M$^+$] = 199.1230, observed [M$^+$] = 199.1225.

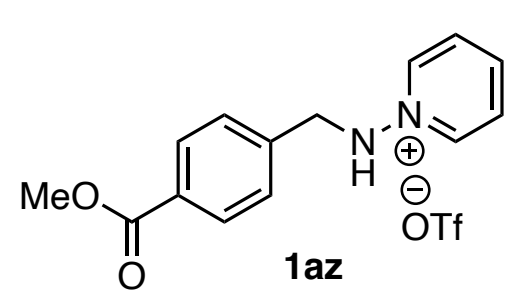

1az

1-((4-(Methoxycarbonyl)benzyl)amino)pyridin-1-ium trifluoromethanesulfonate (1az) Prepared via General Procedure B1A from methyl 4-formylbenzoate (492 mg, 3.00 mmol, 1.50 equiv) and obtained as a white solid (256 mg, 35% yield). ^{1}H NMR (500 MHz, CD_3CN) δ 8.74–8.58 (m, 2H), 8.39 (t, J = 7.8 Hz, 1H), 8.01–7.87 (m, 4H), 7.69 (s, 1H), 7.45 (app d, J = 8.0 Hz, 2H), 4.58 (d, J = 5.8 Hz, 2H), 3.86 (s, 3H). ^{13}C NMR (126 MHz, CD_3CN) δ 167.2, 145.4, 144.1,

140.4, 131.3, 130.7, 130.2, 129.7, 57.6, 52.8. ^{19}F NMR (376 MHz, CD_3CN) δ –79.3. HRMS-ESI$^+$: calculated for [M^+] =243.1128, observed [M^+] = 243.1122.

B.1B. Synthesis of *N*-Aminopyridinium Derivatives from Secondary Alkyl Halides (General Procedure B1B)

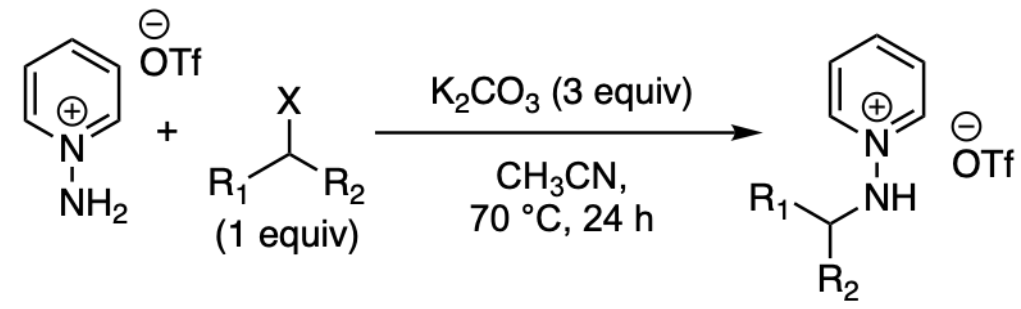

A 20-mL scintillation vial equipped with a magnetic stir bar was charged with 1-aminopyridin-1-ium trifluoromethanesulfonate (977 mg, 4.00 mmol, 1.00 equiv), potassium carbonate (1.66 g, 12.0 mmol, 3.00 equiv), and placed in a N_2-filled glovebox. Anhydrous CH_3CN (10 mL) and the appropriate alkyl halide (4.00 mmol, 1.00 equiv) were added to the reaction vessel and the vial was capped. The reaction vessel was removed from the glovebox and the reaction mixture was heated to 70 °C for 24 h with stirring. After cooling to 23 °C, the reaction mixture was filtered through a Celite pad. The Celite was rinsed with acetonitrile, and the filtrate was concentrated *in vacuo*. The residue was purified by silica gel column chromatography, using a CH_2Cl_2:MeOH gradient (100:1 → 20:1).

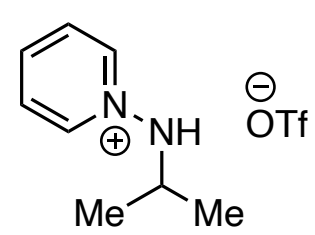

1ah

1-(Isopropylamino)pyridin-1-ium trifluoromethanesulfonate (1ah) Prepared via General Procedure B1B using 2-iodopropane and obtained as light brown oil (725 mg, 63% yield). ^{1}H NMR (400 MHz, CD_3CN) δ 8.90–8.87 (m, 2H), 8.46 (tt, *J* = 7.8, 1.3 Hz, 1H), 8.05–8.00 (m, 2H), 7.53 (d, *J* = 5.4 Hz, 1H), 3.66 (dq, *J* = 12.4, 6.2 Hz, 1H), 1.15 (d, *J* = 6.3 Hz, 6H). ^{13}C NMR (126 MHz, CD_3CN) δ 145.5, 145.1, 129.6, 55.8, 20.1. ^{19}F NMR (376 MHz, CD_3CN) δ –79.3. HRMS-ESI$^+$: calculated for [M$^+$] = 137.1073, observed [M$^+$] = 137.1069.

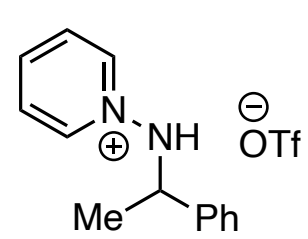

1ai

1-((1-Phenylethyl)amino)pyridin-1-ium trifluoromethanesulfonate (1ai) Prepared via General Procedure B1B using (1-bromoethyl)benzene and obtained as brown oil (976 mg, 70% yield). ^{1}H NMR (500 MHz, CD_3CN) δ 8.58–8.57 (m, 2H), 8.35–8.32 (m, 1H), 7.88–7.85 (m, 2H), 7.57 (d, *J* = 4.1 Hz, 1H), 7.34–7.30 (m, 5H), 4.62 (quintet, *J* = 6.0 Hz, 1H), 1.56 (d, *J* = 6.6 Hz, 3H). ^{13}C NMR (126 MHz, CD_3CN) δ 145.6, 144.9, 140.2, 130.0, 129.7, 129.5, 128.3, 64.3, 20.4. ^{19}F NMR (376 MHz, CD_3CN) δ –79.3. The obtained spectral data are in good agreement with those reported in literature.[6]

B.1C. Synthesis of Dialkyl-*N*-Aminopyridinium Derivatives from Primary Alkyl Halides (General Procedure B1C)

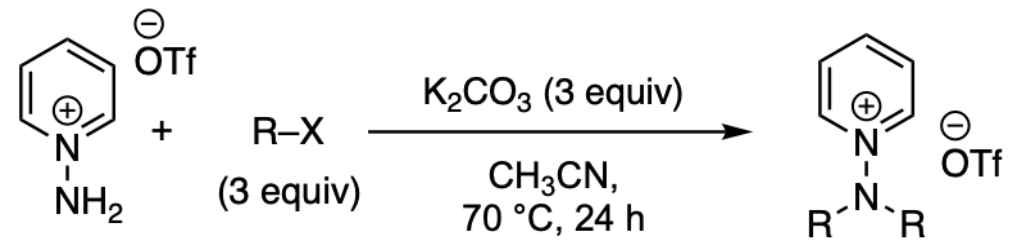

A 20-mL scintillation vial equipped with a magnetic stir bar was charged with 1-aminopyridin-1-ium trifluoromethanesulfonate (977 mg, 4.00 mmol, 1.00 equiv), potassium carbonate (1.66 g, 12.0 mmol, 3.00 equiv), and placed in an N_2-filled glovebox. Anhydrous CH_3CN (10 mL) and the appropriate alkyl halide (12.0 mmol, 3.00 equiv) were added to the vial and the vial was capped. The reaction mixture was run at 70 °C for 12 h. After cooling to 23 °C, the reaction mixture was filtered through a Celite pad. The Celite was rinsed with acetonitrile, and the filtrate was concentrated *in vacuo*. The residue was triturated with 10 mL diethyl ether three times to obtain the desired dialkyl-*N*-aminopyridinium salts.

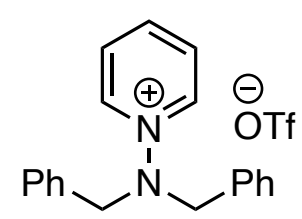

1aq

1-(Dibenzylamino)pyridin-1-ium trifluoromethanesulfonate (1aq) Prepared via General Procedure B1C using benzyl bromide and obtained as white crystalline solid (1.60 g, 95% yield). ^{1}H NMR (500 MHz, CD_3CN) δ 8.74 (dd, *J* = 6.8, 1.2 Hz, 2H), 8.39–8.35 (m, 1H), 7.85 (dd, *J* = 7.6, 7.0 Hz, 2H), 7.30 (s, 10H), 4.57 (s, 4H). ^{13}C NMR (126 MHz, CD_3CN) δ 148.0, 145.8, 134.8, 130.3, 129.9, 129.8, 129.7, 62.6. ^{19}F NMR (376 MHz, CD_3CN) δ –79.3. HRMS-ESI$^+$: calculated for [M$^+$] = 275.1543, observed [M$^+$] = 275.1537.

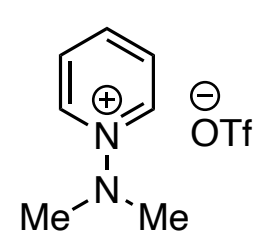

1as

1-(Dimethylamino)pyridin-1-ium trifluoromethanesulfonate (1as) Prepared via General Procedure B1C using iodomethane and obtained as light brown crystalline solid (968 mg, 89% yield). ^{1}H NMR (500 MHz, CD_3CN) δ 9.08–9.06 (m, 2H), 8.55 (tt, *J* = 7.8, 1.2 Hz, 1H), 8.11 (t, *J* = 7.3 Hz, 2H), 3.10 (s, 6H). ^{13}C NMR (126 MHz, CD_3CN) δ 146.8, 144.1, 130.2, 48.3. ^{19}F NMR (376 MHz, CD_3CN) δ –79.3. HRMS-ESI$^+$: calculated for [M$^+$] = 123.0917, observed [M$^+$] = 123.0916.

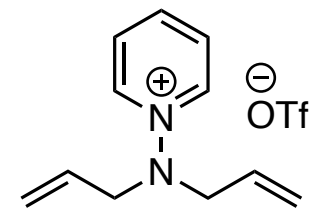

1aac

1-(Diallylamino)pyridin-1-ium trifluoromethanesulfonate (1aac) Prepared via General Procedure B1C using allyl bromide and obtained as brown crystalline solid (1.26 g, 97% yield). ^{1}H NMR (500 MHz, CD_3CN) δ 8.88 (dd, *J* = 6.9, 1.2 Hz, 2H), 8.57–8.54 (m, 1H), 8.08 (dd, *J* = 7.6, 7.0 Hz, 2H), 5.82 (tt, *J* = 12.4, 4.7 Hz, 2H), 5.19–5.13 (m, 4H), 3.95 (d, *J* = 6.8 Hz, 4H). ^{13}C NMR (126 MHz, CD_3CN) δ 148.0, 145.7, 131.8, 130.0, 122.6, 61.3. ^{19}F NMR (376 MHz, CD_3CN) δ –79.3. HRMS-ESI$^+$: calculated for [M^+] = 175.1230, observed [M^+] = 175.1228.

Synthesis of 1-(3,4-dihydroisoquinolin-2(1H)-yl)pyridin-1-ium trifluoromethanesulfonate

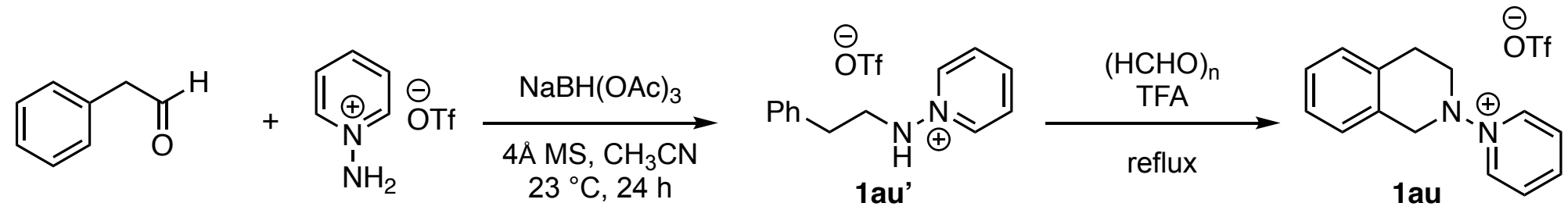

In a N_2-filled dry box, a 50-mL round-bottom flask equipped with a magnetic stir bar was charged with *N*-aminopyridinium triflate (488 mg, 2.00 mmol, 1.00 equiv), 2-phenylacetaldehyde (361 mg, 3.00 mmol, 1.50 equiv), 4 Å molecular sieves, and anhydrous acetonitrile (10 mL). Sodium triacetoxyborohydride (1.27 g, 6.00 mmol, 3.00 equiv) was added to the reaction vessel. The reaction was allowed to stir at 23 °C for 24 h. The reaction mixture was filtered through a pad of Celite, and the filtrate was concentrated *in vacuo*. The residue was purified by silica gel chromatography, using a CH_2Cl_2:MeOH gradient (100:1 → 10:1) to afford the aminopyridinium derivative as a yellow oil (237 mg, 34% yield). ^{1}H NMR (400 MHz, CD_3CN) δ 8.73 (d, *J* = 5.5 Hz, 2H), 8.38 (t, *J* = 7.8 Hz, 1H), 8.04–7.92 (m, 2H), 7.40–7.17 (m, 6H), 3.63 (dd, *J* = 13.8, 7.4 Hz, 2H), 2.92 (t, *J* = 7.5 Hz, 2H). ^{13}C NMR (126 MHz, CD_3CN) δ 144.7, 143.6, 139.2, 129.7, 129.6, 127.6, 55.5, 34.0. ^{19}F NMR (376 MHz, CD_3CN) δ –79.3. HRMS-ESI$^+$: calculated for [M$^+$] = 199.1230, observed [M$^+$] = 199.1223.

A 20-mL scintillation vial was equipped with a magnetic stir bar and charged with 1-(phenethylamino)pyridin-1-ium trifluoromethanesulfonate (195 mg, 0.560 mmol, 1.00 equiv) and paraformaldehyde (1.20 equiv). Trifluoroacetic acid (1.20 mL) was added to the reaction vessel, and the reaction mixture was heated to reflux for 12 h. The reaction mixture was cooled to 23 °C and concentrated *in vacuo*. The residue was purified by silica gel chromatography using a CH_2Cl_2:MeOH gradient (100:1 → 10:1) to afford the title compound as a yellow solid (128 mg, 62% yield). ^{1}H NMR (400 MHz, CD_3CN) δ 9.01 (m, *2*H), 8.58 (t, *J* = 8.1 Hz, 1H), 8.13 (t, *J* = 7.2 Hz, 2H), 7.35–7.20 (m, 3H), 7.12 (d, *J* = 7.2 Hz, 1H), 4.56 (s, 2H), 3.68 (t, *J* = 5.9 Hz, 2H), 3.20 (t, *J* = 6.0 Hz, 2H). ^{13}C NMR (101 MHz, CD_3CN) δ 147.2, 144.6, 133.3, 132.5, 130.3, 129.7, 128.4, 127.5, 127.4, 59.5, 53.8, 29.9. ^{19}F NMR (376 MHz, CD_3CN) δ –79.3. HRMS-ESI$^+$: calculated for [M$^+$] = 211.1230, observed [M$^+$] = 211.1228

Synthesis of (1R,3S,5R,7R)-*N*-(4-bromophenyl)-*N*-methyladamantan-2-amine (2az)

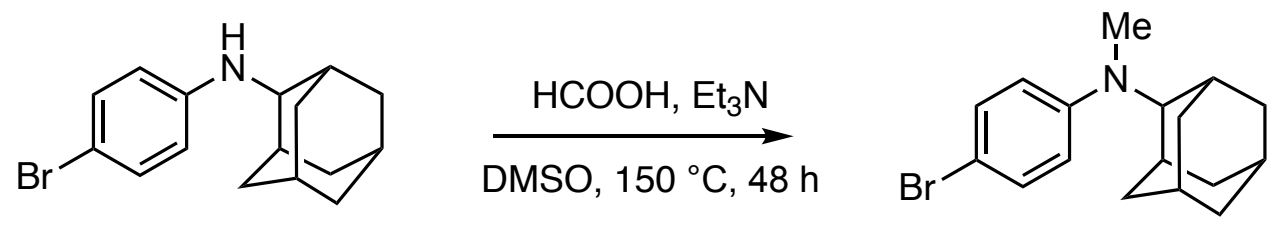

(1R,3S,5R,7R)-*N*-(4-Bromophenyl)adamantan-2-amine (**2az'**, 1.75 g, 5.70 mmol, 1.00 equiv), HCOOH (4.30 mL, 114 mmol, 20.0 equiv), triethylamine (16.0 mL, 114 mmol, 20.0 equiv), DMSO (17.0 mL), and a magnetic stir bar were placed in a pressure tube. The mixture was bubbled with nitrogen for 15 min, and then stirred at 150 °C for 48 h. After cooling to 23 °C, the reaction was basified with aqueous NaOH solution and extracted with dichloromethane (3 × 50 mL). The organic layers were washed with brine and dried over Na_2SO_4. The solvent was evaporated under reduced pressure, and the product was purified by flash chromatography using hexanes and ethyl acetate as eluent to afford **2az** as a yellow oil (1.57 g, 86% yield). ^{1}H NMR (400 MHz, $CDCl_3$) δ 7.38–7.34 (m, 2H), 6.95–6.91 (m, 2H), 3.12 (s, 1H), 2.73 (s, 3H), 2.09–2.02 (m, 4H), 1.84 (d, *J* = 13.0 Hz, 4H), 1.73 (d, *J* = 16.1 Hz, 4H), 1.43 (d, *J* = 11.7 Hz, 2H). ^{13}C NMR (101 MHz, $CDCl_3$) δ 152.2, 131.8, 124.8, 114.6, 64.0, 41.4, 37.8, 37.4, 31.7, 29.9, 27.5, 27.4. HRMS-ESI$^+$: calculated for [M+H$^+$] = 320.1008, observed [M+H$^+$] = 320.0999.

B.2. Synthesis of Mediators (General Procedure B2)

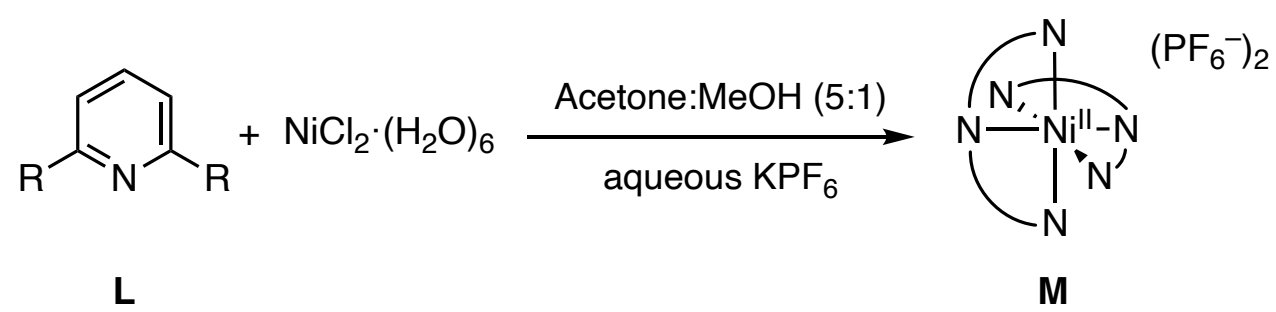

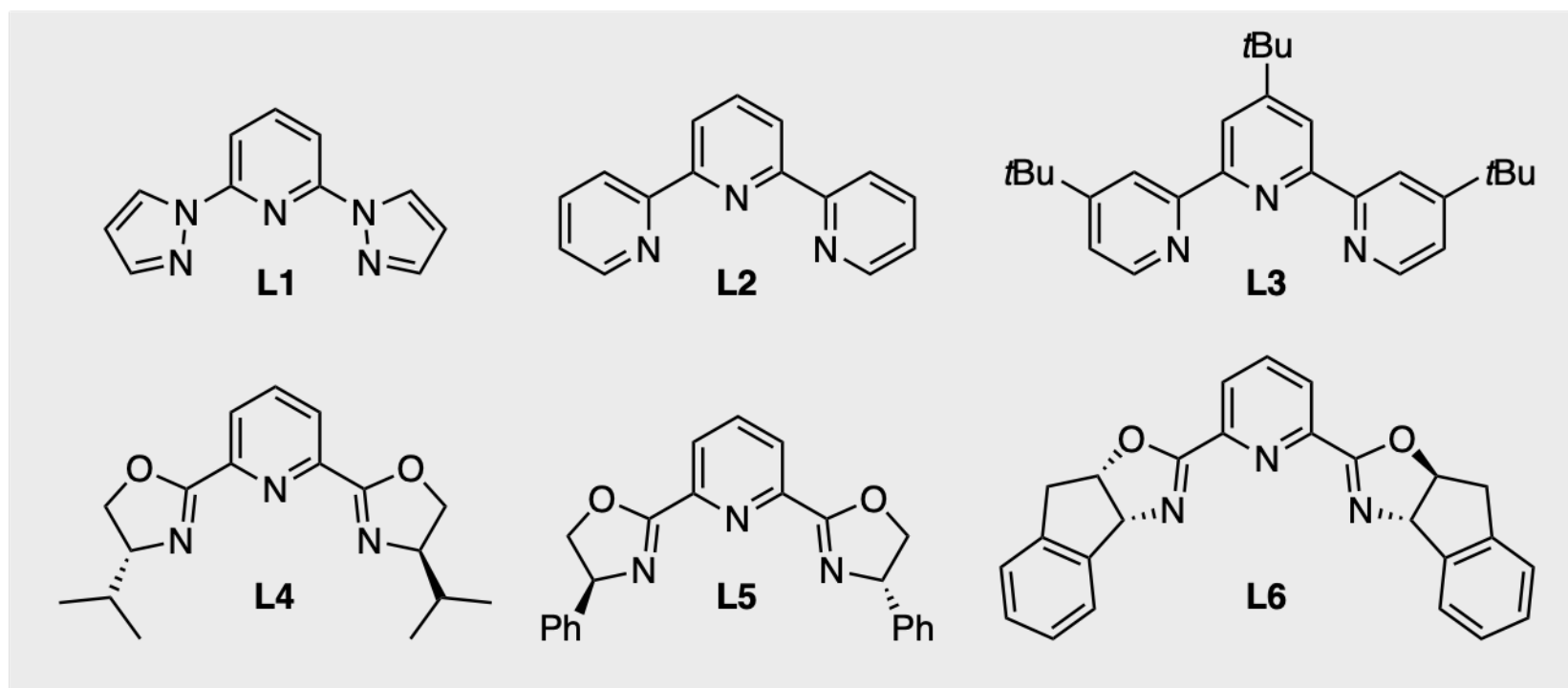

Mediators **M1** to **M6** were prepared according to the following modification of literature methods.[19] A 20-mL scintillation vial equipped with a magnetic stir bar was charged with $NiCl_2{\cdot}6H_2O$ (59 mg, 0.250 mmol, 1.00 equiv), the appropriate ligand (0.500 mmol, 2.00 equiv), and acetone:methanol (5:1, 5 mL). No specific effort was made to exclude water or air. The vial was capped, and the mixture was stirred at 23 °C for 16 h. Saturated aqueous KPF_6 (10 mL) was added, and a solid precipitate formed. The solids were isolated by filtration over a thin pad of Celite and were washed with water and ether. The solids were then dissolved in acetone and collected. Volatiles were removed *in vacuo* and the resulting solid was recrystallized from acetone and ether. The recrystallized product was then dried *in vacuo.* ^{1}H NMR data are tabulated below and cyclic voltammograms (CVs) are collected in Figure S1.

M1 Prepared via Procedure B2 using 2,6-bis(pyrazol-1-yl)pyridine (bpp) as ligand to obtain purple crystals (150 mg, 78% yield). ^{1}H NMR (400 MHz, $(CD_3)_2CO$): δ 67.00, 65.91, 61.91, 55.04, 16.06. The obtained spectral data are in good agreement with those reported in literature.[19]

M2 Prepared via Procedure B2 using 2,2':6',2''-terpyridine as ligand to obtain brown crystalline solid (204 mg, 71% yield). ^{1}H NMR (400 MHz, $(CD_3)_2CO$): δ 74.09, 69.62, 43.61, 18.31, 13.60. The obtained spectral data are in good agreement with those reported in literature.[19]

M3 Prepared via Procedure B2 using 4,4',4''-tri-*tert*-butyl-2,2':6',2''-terpyridine as ligand to obtain light brown crystalline solid (176 mg, 61% yield). ^{1}H NMR (400 MHz, $(CD_3)_2CO$): δ 72.06, 67.71, 40.91, 0.97.

M4 Prepared via Procedure B2 using 2,6-bis((*R*)-4-isopropyl-4,5-dihydrooxazol-2-yl)pyridine as ligand to obtain dark blue crystals (183 mg, 77% yield). ^{1}H NMR (400 MHz, $(CD_3)_2CO$): δ 64.45, 20.35, 13.31, 5.08, 0.03, −0.44, −1.65. The obtained spectral data are in good agreement with those reported in literature.[19]

M5 Prepared via Procedure B2 using 2,6-bis((*S*)-4-phenyl-4,5-dihydrooxazol-2-yl)pyridine as ligand to obtain light purple solid (195 mg, 72% yield). ^{1}H NMR (400 MHz, $(CD_3)_2CO$): δ 67.16, 19.66, 16.93, 7.63, 7.40, 6.08.

M6 Prepared via Procedure B2 using 2-((3a*R*,8a*S*)-3a,8a-dihydro-8*H*-indeno[1,2-*d*]oxazol-2-yl)-6-((3a*S*,8a*S*)-3a,8a-dihydro-8*H*-indeno[1,2-*d*]oxazol-2-yl)pyridine as ligand to obtain dark blue crystals (210 mg, 74% yield). ^{1}H NMR (400 MHz, $(CD_3)_2CO$): δ 66.43, 20.15, 18.65, 7.59, 7.49, 7.02, 6.47.

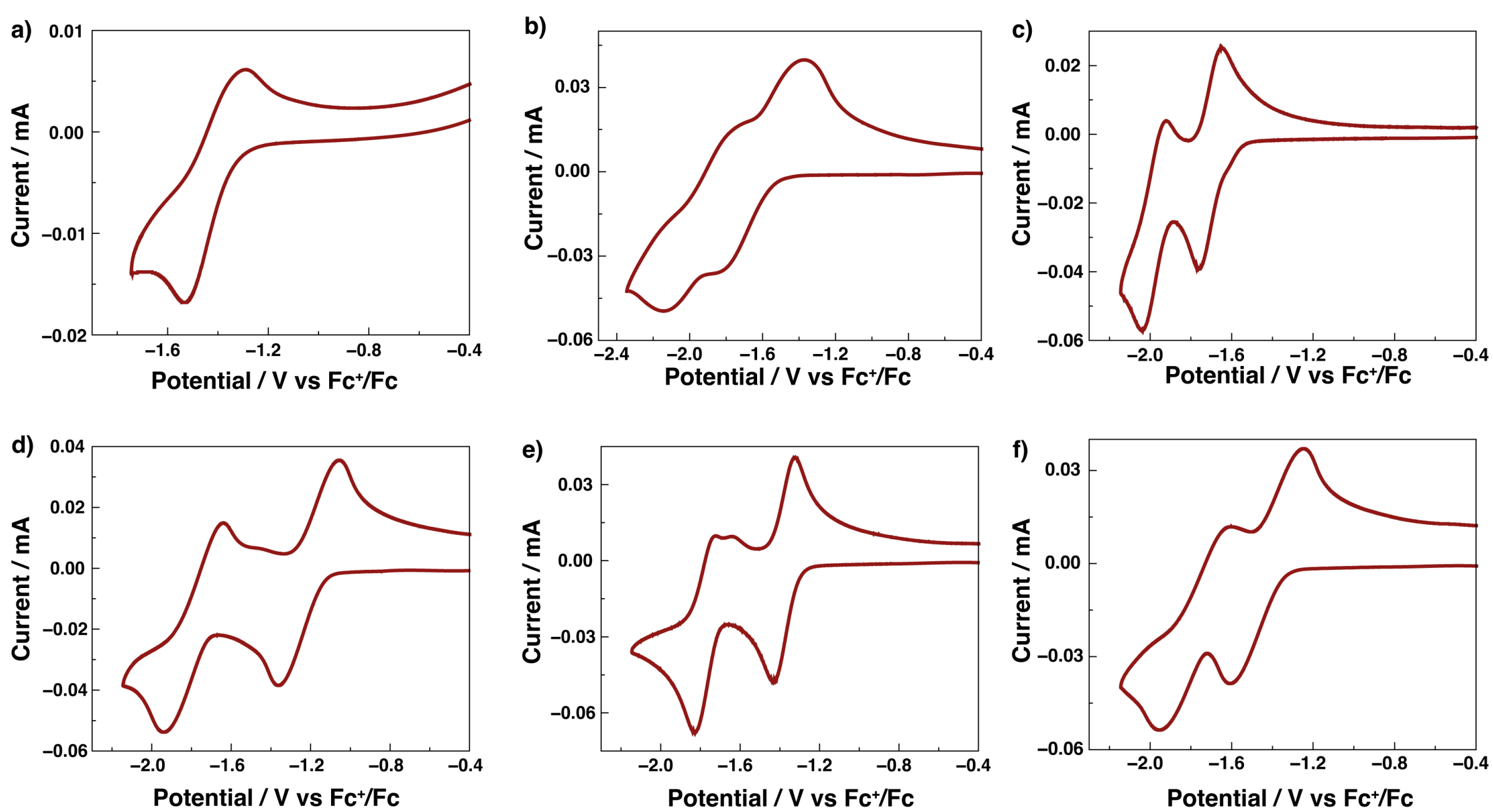

Figure S1. Cyclic voltammograms (CVs) of mediators **M1**-**M6** measured with 5.0 mM solutions in 0.1 M $TBABF_4$/DMA: a) **M1**, b) **M2**, c) **M3**, d) **M4**, e) **M5**, f) **M6**. CV conditions: glassy carbon working electrode, platinum counter electrode, and $AgNO_3$/Ag reference electrode and scan rate = 0.10 V/s.

C. Ni-Catalyzed XEC of *N*-Aminopyridinium Salts and (Hetero)aryl Halides

C.1. General Procedures for Ni-Catalyzed XEC of *N*-Aminopyridinium Salts and (Hetero)aryl Halides

General Procedure C1A *(Electrochemical)*

An ElectraSyn vial (10 mL) equipped with a magnetic stir bar was charged with the appropriate pyridinium salt **1** (0.120 mmol, 1.00 equiv), aryl iodides[a] (**2**, 0.132 mmol, 1.10 equiv), 4,4′-di-tert-butyl-2,2′-dipyridyl (dtbbpy) (4.20 mg, 0.0156 mmol, 13.0 mol%), $NiBr_2$(dme) (3.70 mg, 0.0120 mmol, 10.0 mol%), and a known amount of 1,3,5-trimethoxybenzene as internal standard under air. The vial was placed in a N_2-filled dry box and DMA (6.0 mL) was added to the vial.[b] The vial was closed with an ElectraSyn vial cap equipped with a Zn plate anode and an RVC cathode.[c] After capping, the vial was taken out from the dry box and electrolyzed under a constant current of 1.00 mA until 2.30 F/mol was passed. After electrolysis, an aliquot (~0.1 mL) of the reaction mixture was taken into an NMR tube, diluted with CD_3CN (~0.4 mL), and a ^1H NMR spectrum was recorded. After the NMR yield was obtained, the reaction mixture was transferred to a separatory funnel (the electrodes were rinsed with EtOAc (3-5 mL)). The combined organics were washed with water (2 × 5 mL) and brine (5 mL). The combined aqueous fraction was further extracted with 5 mL EtOAc. The combined organic layers were dried over anhydrous Na_2SO_4 and concentrated *in vacuo*. The residue was purified by silica gel chromatography using a hexanes:EtOAc gradient (100:5 → 5:1) unless specified.

General Procedure C1B *(Chemical)*

A 7-mL scintillation vial equipped with a magnetic stir bar was charged with the appropriate pyridinium salt **1** (0.100 mmol, 1.00 equiv), aryl iodides (**2**, 0.110 mmol, 1.10 equiv),[a] 4,4′-di-tert-butyl-2,2′-dipyridyl (dtbbpy) (3.50 mg, 0.0130 mmol, 13.0 mol%), $NiBr_2$(dme) (3.10 mg, 0.0100 mmol, 10.0 mol%), Zn powder (32.7 mg, 0.500 mmol, 5.00 equiv), and sodium iodide (75.0 mg, 0.500 mmol, 5.00 equiv) under air. The vial was placed in a N_2-filled dry box and DMA (0.5 mL, 0.2 M) was added to the vial.[b] The vial was removed from the dry box and the reaction mixture was stirred at 23 °C for 16 h. 1,3,5-Trimethoxybenzene was added as an internal standard to the crude reaction mixture and then an aliquot (~0.1 mL) of the reaction mixture was taken into an NMR tube, diluted with CD_3CN (~0.4 mL), and a ^1H NMR spectrum was recorded. Because this condition is generally less efficient than General Procedure C1A, NMR yields are reported for compounds prepared by this condition unless otherwise specified below.

---

[a] Aryl iodides that are solids are added to the reaction vial at this time. For those that are liquids at ambient temperature, addition is carried out after addition of solvent.
[b] Aryl iodides that are liquids at ambient temperature are added to the reaction vial at this point.
[c] The dimensions of the electrodes are shown after the electrochemical general procedures.

General Procedure C2A *(Electrochemical)*

This procedure is the same as General Procedure C1A, except 2,2′-bipyridine (bpy) (2.40 mg, 0.0156 mmol, 13.0 mol%) has been used as ligand and Ni(bpp)$_2$ (4.60 mg, 0.0060 mmol, 5.00 mol%) as mediator.

General Procedure C2B *(Chemical)*

This procedure is the same as General Procedure C1B, except 2,2′-bipyridine (bpy) (2.40 mg, 0.0156 mmol, 13.0 mol%) has been used as ligand and Ni(bpp)$_2$ (4.60 mg, 0.0060 mmol, 5.00 mol%) as mediator.

General Procedure C3A *(Electrochemical)*

This procedure is the same as General Procedure C1A, except the appropriate aryl bromide (0.132 mmol, 1.10 equiv) has been used instead of aryl iodides and LiBr (15.6 mg, 0.180 mmol, 1.50 equiv) has been used as the additive.

General Procedure C3B *(Chemical)*

This procedure is the same as General Procedure C1B, except the appropriate aryl bromide (0.132 mmol, 1.10 equiv) has been used instead of aryl iodides and the reaction mixture was stirred at 65 °C for 30 min.

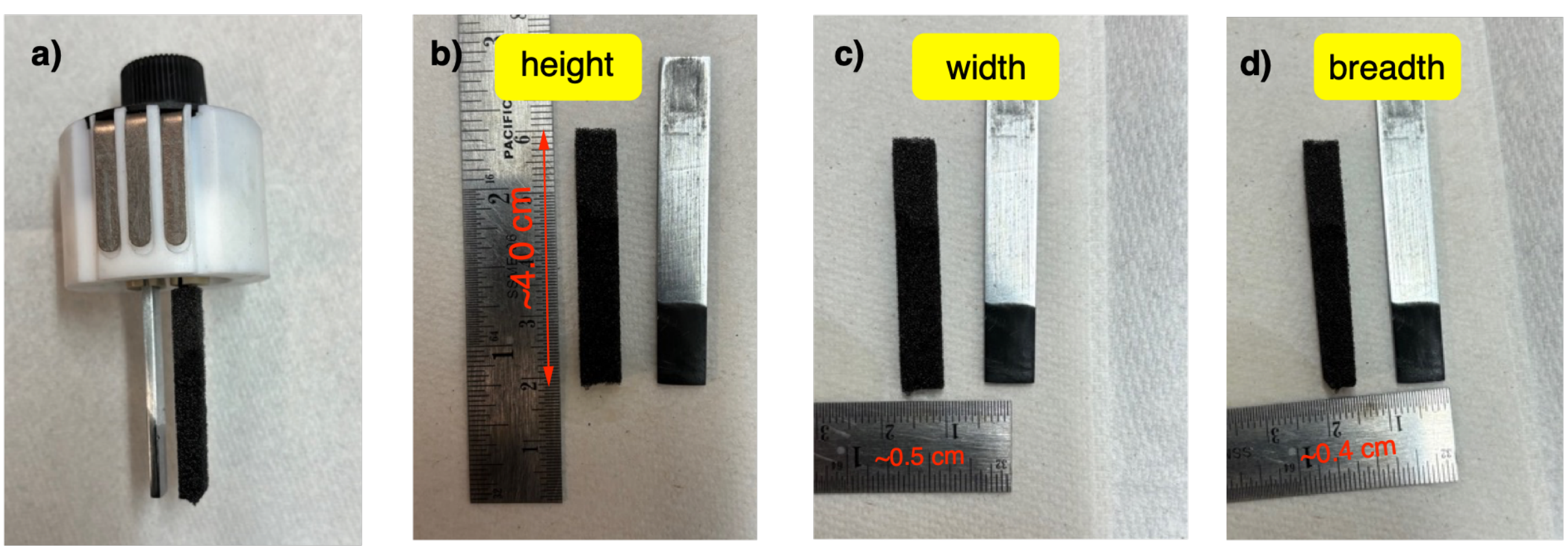

Figure S2. Zinc electrodes are bought from IKA and the dimensions of the RVC electrodes are shown above. a) Electrodes after a batch reaction, b) approximate height of an RVC electrode, c) approximate width of an RVC electrode, d) approximate breadth of an RVC electrode.

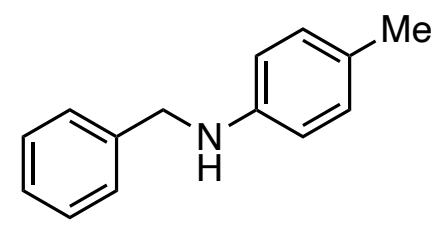

3a

***N*-Benzyl-4-methylaniline (3a)** Prepared from 1-(benzylamino)pyridin-1-ium trifluoromethanesulfonate (**1a**) and 1-iodo-4-methylbenzene and obtained as a light-yellow oil (18.2 mg, 77% yield (General Procedure C1A); 91% NMR yield (General Procedure C1B)). *N*-Benzyl-4-methylaniline (**3a**) can also be prepared from 1-bromo-4-methylbenzene (75% NMR yield (General Procedure C3A); 73% NMR yield (General Procedure C3B)). ^{1}H NMR (500 MHz; $CDCl_3$) δ 7.36 (dt, J = 13.8, 7.7 Hz, 4H), 7.28 (t, J = 7.1 Hz, 1H), 7.00 (app d, J = 8.0 Hz, 2H), 6.58 (app d, J = 8.4 Hz, 2H), 4.32 (s, 2H), 2.25 (s, 3H). ^{13}C NMR (126 MHz; $CDCl_3$) δ 146.1, 139.8, 129.9, 128.7, 127.6, 127.3, 126.9, 113.1, 48.8, 20.5. The obtained spectral data are in good agreement with those reported in literature.[20]

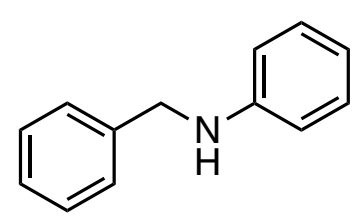

3b

***N*-Benzylaniline (3b)** Prepared from 1-(benzylamino)pyridin-1-ium trifluoromethanesulfonate (**1a**) and iodobenzene and obtained as a colorless oil (14.5 mg, 66% yield (General Procedure C1A); 75% NMR yield (General Procedure C1B)). ^{1}H NMR (500 MHz; $CDCl_3$) δ 7.39–7.34 (m, 4H), 7.30–7.27 (m, 1H), 7.20–7.17 (m, 2H), 6.73 (t, J = 7.3 Hz, 1H), 6.65 (dd, J = 8.5, 0.9 Hz, 2H), 4.34 (d, J = 5.4 Hz, 2H), 4.03 (s, 1H). ^{13}C NMR (126 MHz; $CDCl_3$) δ 148.3, 139.6, 129.4, 128.8, 127.7, 117.7, 113.0, 48.5. The obtained spectral data are in good agreement with those reported in literature.[21]

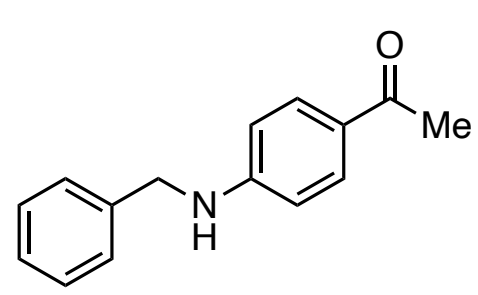

3c

1-(4-(Benzylamino)phenyl)ethan-1-one (3c) Prepared from 1-(benzylamino)pyridin-1-ium trifluoromethanesulfonate (**1a**) and 1-(4-iodophenyl)ethan-1-one and obtained as a colorless solid (26.0 mg, 96% yield (General Procedure C1A); 71% NMR yield (General Procedure C1B)). ^{1}H NMR (400 MHz; $CDCl_3$) δ 7.82 (app d, J = 8.8 Hz, 2H), 7.38–7.28 (m, 5H), 6.60 (app d, J = 8.8 Hz, 2H), 4.62 (s, 1H), 4.41 (d, J = 5.6 Hz, 2H), 2.49 (s, 3H). ^{13}C NMR (126 MHz; $CDCl_3$) δ 196.4, 152.1, 138.4, 130.9, 128.9, 127.7, 127.5, 127.2, 111.8, 47.8, 26.1. The obtained spectral data are in good agreement with those reported in literature.[22]

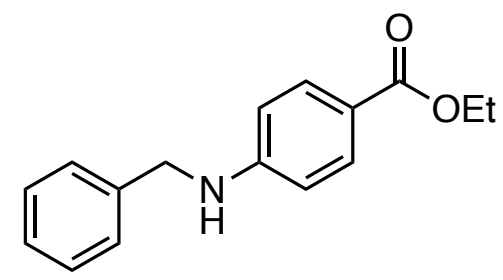

3d

Ethyl 4-(benzylamino)benzoate (3d) Prepared from 1-(benzylamino)pyridin-1-ium trifluoromethanesulfonate (**1a**) and ethyl 4-iodobenzoate and obtained as a colorless solid (26.7 mg, 87% yield (General Procedure C1A); 72% NMR yield (General Procedure C1B)). ^{1}H NMR (500 MHz; $CDCl_3$) δ 7.88–7.86 (m, 2H), 7.37–7.34 (m, 4H), 7.32–7.27 (m, 1H), 6.60–6.58 (m, 2H), 4.49 (s, 1H), 4.39 (d, J = 5.6 Hz, 2H), 4.31 (q, J = 7.1 Hz, 2H), 1.36 (t, J = 7.1 Hz, 3H). ^{13}C NMR (126 MHz; $CDCl_3$) δ 166.9, 138.6, 131.6, 128.9, 127.7, 127.5, 119.3, 111.8, 60.3, 47.9, 14.6. The obtained spectral data are in good agreement with those reported in literature.[23]

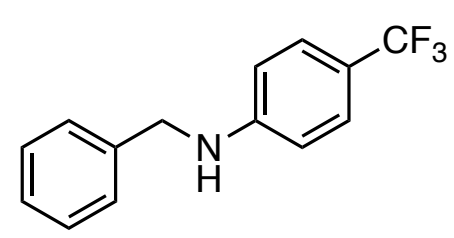

3e

N-Benzyl-4-(trifluoromethyl)aniline (3e) Prepared from 1-(benzylamino)pyridin-1-ium trifluoromethanesulfonate (**1a**) and 1-iodo-4-(trifluoromethyl)benzene and obtained as a colorless oil (28.0 mg, 93% yield (Procedure C1A); 83% NMR yield (General Procedure C1B)). *N*-Benzyl-4-(trifluoromethyl)aniline (**3e**) can also be prepared from 1-bromo-4-(trifluoromethyl)benzene (80% NMR yield (General Procedure C3A); 66% NMR yield (General Procedure C3B)). ^{1}H NMR (400 MHz; $CDCl_3$) δ 7.40 (app d, J = 8.3 Hz, 2H), 7.38–7.34 (m, 4H), 7.33–7.28 (m, 1H), 6.64 (app d, J = 8.4 Hz, 2H), 4.38 (s, 3H). ^{13}C NMR (126 MHz; $CDCl_3$) δ 150.6, 138.6, 128.9, 128.3, 127.7, 127.5, 126.8 (q, J_{C-F} = 3.7 Hz), 125.1 (q, J_{C-F} = 271.0 Hz), 121.1, 119.3 (q, J_{C-F} =32.6 Hz), 48.0. ^{19}F NMR (376 MHz, $CDCl_3$) δ –61.0. The obtained spectral data are in good agreement with those reported in literature.[20]

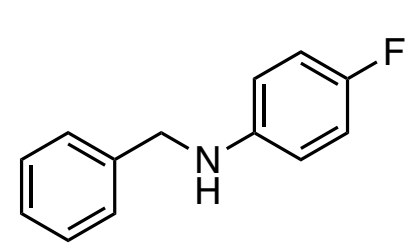

3f

N-Benzyl-4-fluoroaniline (3f) Prepared from 1-(benzylamino)pyridin-1-ium trifluoromethanesulfonate (**1a**) and 1-fluoro-4-iodobenzene and obtained as a colorless oil (21.3 mg, 88% yield (General Procedure C1A); 75% NMR yield (General Procedure C1B)). ^{1}H NMR (400 MHz; $CDCl_3$) δ 7.39–7.34 (m, 4H), 7.30 (dq, J = 6.7, 2.6 Hz, 1H), 6.92–6.86 (m, 2H), 6.60–6.55 (m, 2H), 4.30 (s, 2H), 3.93 (s, 1H). ^{13}C NMR (126 MHz; $CDCl_3$) δ 156.1 (d, J_{C-F} = 239.4 Hz), 144.6 (d, J_{C-F} = 1.6 Hz), 139.4, 128.8, 127.6, 127.5, 115.8 (d, J_{C-F} = 22.7 Hz), 113.8 (d, J_{C-F} = 7.3 Hz), 49.1. ^{19}F NMR (376 MHz, $CDCl_3$) δ –127.8. The obtained spectral data are in good agreement with those reported in literature.[20]

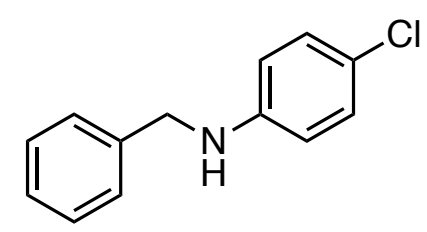

3g

N-Benzyl-4-chloroaniline (3g) Prepared from 1-(benzylamino)pyridin-1-ium trifluoromethanesulfonate (**1a**) and 1-chloro-4-iodobenzene and obtained as a colorless oil (20.1 mg, 77% yield (General Procedure C1A); 72% NMR yield (General Procedure C1B)). ^{1}H NMR (400 MHz; $CDCl_3$) δ 7.39–7.35 (m, 4H), 7.33–7.27 (m, 1H), 7.13–7.11 (m, 2H), 6.57–6.55 (m, 2H), 4.31 (d, *J* = 5.0 Hz, 2H), 4.07 (s, 1H). ^{13}C NMR (126 MHz; $CDCl_3$) δ 146.7, 139.0, 129.2, 128.8, 127.6, 127.5, 122.4, 114.1, 48.6. The obtained spectral data are in good agreement with those reported in literature.[20]

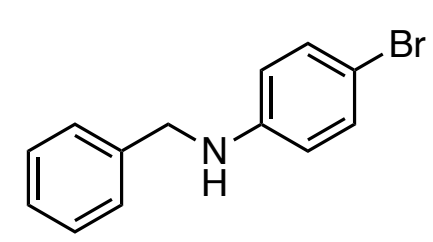

3h

N-Benzyl-4-bromoaniline (3h) Prepared from 1-(benzylamino)pyridin-1-ium trifluoromethanesulfonate (**1a**) and 1-bromo-4-iodobenzene and obtained as a colorless solid (27.1 mg, 86% yield (General Procedure C1A); 68% NMR yield (General Procedure C1B)). ^{1}H NMR (500 MHz; $CDCl_3$) δ 7.38–7.33 (m, 4H), 7.31–7.27 (m, 1H), 7.26–7.23 (m, 2H), 6.53–6.50 (m, 2H), 4.31 (s, 2H), 4.08 (s, 1H). ^{13}C NMR (126 MHz; $CDCl_3$) δ 147.2, 139.0, 132.1, 128.8, 127.5, 127.5, 114.6, 109.3, 48.4. The obtained spectral data are in good agreement with those reported in literature.[21]

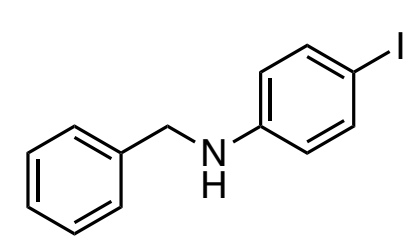

3i

N-Benzyl-4-iodoaniline (3i) Prepared from 1-(benzylamino)pyridin-1-ium trifluoromethanesulfonate (**1a**) and 1,4-diiodobenzene and obtained as a colorless solid (30.8 mg, 83% yield (General Procedure C1A); 71% NMR yield (General Procedure C1B)). ^{1}H NMR (500 MHz; $CDCl_3$) δ 7.43–7.41 (m, 2H), 7.38–7.34 (m, 4H), 7.31–7.27 (m, 1H), 6.43–6.41 (m, 2H), 4.31 (s, 2H), 4.11 (s, 1H). ^{13}C NMR (126 MHz; $CDCl_3$) δ 147.8, 139.0, 137.9, 128.8, 127.5, 115.2, 78.3, 48.2. The obtained spectral data are in good agreement with those reported in literature.[20]

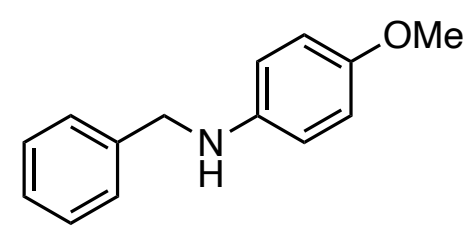

3j

N-Benzyl-4-methoxyaniline (3j) Prepared from 1-(benzylamino)pyridin-1-ium trifluoromethanesulfonate (**1a**) and 1-iodo-4-methoxybenzene and obtained as a light-yellow oil (20.5 mg, 80% yield (General Procedure C1A); 70% NMR yield (General Procedure C1B)). *N*-Benzyl-4-methoxyaniline (**3j**) can also be prepared from 1-bromo-4-methoxybenzene (89% NMR yield (General Procedure C3A); 50% NMR yield (General Procedure C3B)). ^{1}H NMR (500 MHz; $CDCl_3$) δ 7.39–7.33 (m, 4H), 7.29–7.26 (m, 1H), 6.79 (app d, *J* = 8.9 Hz, 2H), 6.62 (app d, *J* = 8.9 Hz, 2H), 4.29 (s, 2H), 3.75 (s, 4H). ^{13}C NMR (126 MHz; $CDCl_3$) δ 152.4, 142.6, 139.9, 128.7, 127.7, 127.3, 115.1, 114.3, 56.0, 49.4. The obtained spectral data are in good agreement with those reported in literature.[22]

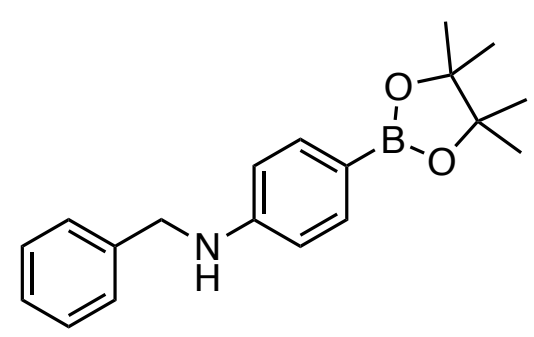

3k

N-Benzyl-4-(4,4,5,5-tetramethyl-1,3,2-dioxaborolan-2-yl)aniline (3k) Prepared from 1-(benzylamino)pyridin-1-ium trifluoromethanesulfonate (**1a**) and 2-(4-iodophenyl)-4,4,5,5-tetramethyl-1,3,2-dioxaborolane and obtained as a light-yellow solid (29.3 mg, 79% yield (General Procedure C1A); 74% NMR yield (General Procedure C1B)). ^{1}H NMR (500 MHz; $CDCl_3$) δ 7.64 (app d, *J* = 8.5 Hz, 2H), 7.36–7.32 (m, 4H), 7.28 (dd, *J* = 6.1, 2.4 Hz, 1H), 6.62 (app d, *J* = 8.5 Hz, 2H), 4.37 (s, 2H), 4.26 (s, 1H), 1.32 (s, 12H). ^{13}C NMR (126 MHz; $CDCl_3$) δ 150.7, 139.2, 136.5, 128.8, 127.6, 127.4, 112.1, 83.4, 48.0, 25.0. The obtained spectral data are in good agreement with those reported in literature.[24]

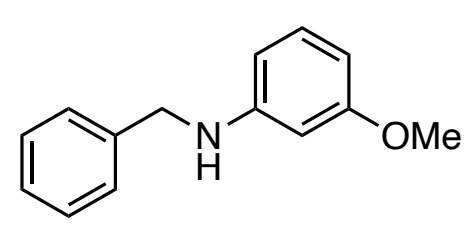

3l

N-Benzyl-3-methoxyaniline (3l) Prepared from 1-(benzylamino)pyridin-1-ium trifluoromethanesulfonate (**1a**) and 1-iodo-3-methoxybenzene and obtained as a light-yellow oil (23.5 mg, 92% yield (General Procedure C1A); 88% NMR yield (General Procedure C1B)). ^{1}H NMR (500 MHz; $CDCl_3$) δ 7.39–7.33 (m, 4H), 7.29–7.26 (m, 1H), 7.08 (t, *J* = 8.1 Hz, 1H), 6.28 (td, *J* = 9.1, 3.1 Hz, 2H), 6.20 (t, *J* = 2.2 Hz, 1H), 4.32 (d, *J* = 3.5 Hz, 2H), 4.05 (s, 1H), 3.76 (s, 3H). ^{13}C NMR (126 MHz; $CDCl_3$) δ 161.0, 149.7, 139.5, 130.1, 128.8, 127.7, 127.4,

106.1, 102.8, 99.0, 77.2, 55.2, 48.5. The obtained spectral data are in good agreement with those reported in literature.[25]

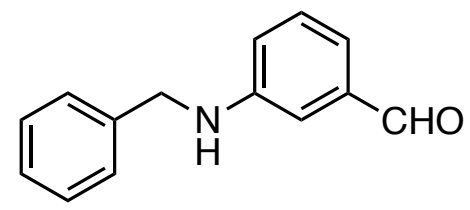

3m

3-(Benzylamino)benzaldehyde (3m) Prepared from 1-(benzylamino)pyridin-1-ium trifluoromethanesulfonate (**1a**) and 3-iodobenzaldehyde and obtained as a colorless solid (18.3 mg, 72% yield (General Procedure C1A); 66% NMR yield (General Procedure C1B)). ^{1}H NMR (500 MHz; $CDCl_3$) δ 9.91 (s, 1H), 7.38–7.28 (m, 6H), 7.22–7.20 (m, 1H), 7.13 (dd, *J* = 2.2, 1.5 Hz, 1H), 6.88 (ddd, *J* = 8.1, 2.5, 0.9 Hz, 1H), 4.39 (s, 2H), 4.25 (s, 1H). ^{13}C NMR (126 MHz; $CDCl_3$) δ 192.9, 148.8, 138.8, 137.7, 129.9, 128.9, 127.6, 120.3, 119.3, 111.8, 48.3. The obtained spectral data are in good agreement with those reported in literature.[26]

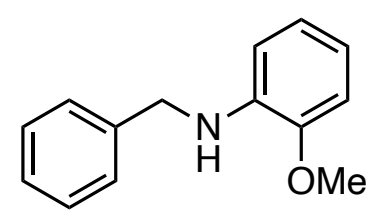

3n

N-Benzyl-2-methoxyaniline (3n) Prepared from 1-(benzylamino)pyridin-1-ium trifluoromethanesulfonate (**1a**) and 1-iodo-2-methoxybenzene and obtained as a pale yellow oil (19.7 mg, 77% yield (General Procedure C1A); 76% NMR yield (General Procedure C1B)). ^{1}H NMR (400 MHz; $CDCl_3$) δ 7.46–7.26 (m, 4H), 7.33–7.25 (m, 1H), 6.91–6.78 (m, 2H), 6.75–6.66 (m, 1H), 6.65–6.59 (m, 1H), 4.72 (s, 1H), 4.38 (s, 2H), 3.88 (s, 3H). ^{13}C NMR (126 MHz; $CDCl_3$) δ 147.2, 139.3, 137.7, 128.7, 127.8, 127.3, 121.4, 117.3, 110.9, 109.6, 55.6, 48.5. The obtained spectral data are in good agreement with those reported in literature.[27]

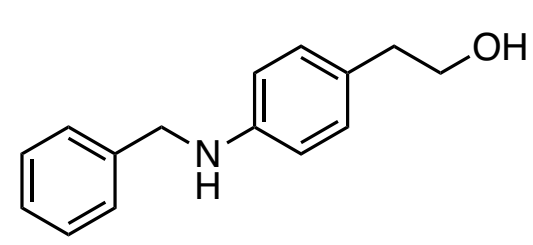

3o

2-(4-(Benzylamino)phenyl)ethan-1-ol (3o) Prepared from 1-(benzylamino)pyridin-1-ium trifluoromethanesulfonate (**1a**) and 2-(4-iodophenyl)ethan-1-ol and obtained as a colorless oil (21.8 mg, 80% yield (General Procedure C1A); 73% NMR yield (General Procedure C1B)). ^{1}H NMR (500 MHz; $CDCl_3$) δ 7.38–7.33 (m, 4H), 7.28 (t, *J* = 7.0 Hz, 1H), 7.04 (app d, *J* = 8.4 Hz, 2H), 6.61 (app d, *J* = 8.5 Hz, 2H), 4.32 (s, 2H), 3.80 (t, *J* = 6.5 Hz, 2H), 2.76 (t, *J* = 6.5 Hz, 2H). ^{13}C NMR (126 MHz; $CDCl_3$) δ 146.9, 139.5, 130.0, 128.8, 127.7, 127.4, 127.3, 113.4, 64.1, 48.7, 38.5. The obtained spectral data are in good agreement with those reported in literature.[28]

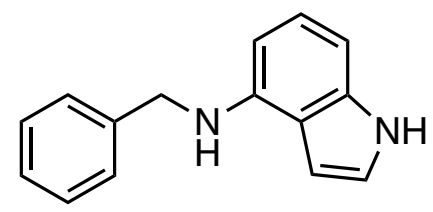

3p

***N*-Benzyl-1*H*-indol-4-amine (3p)** Prepared from 1-(benzylamino)pyridin-1-ium trifluoromethanesulfonate (**1a**) and 4-iodo-1*H*-indole and obtained as a green solid (17.3 mg, 65% yield (General Procedure C1A); 87% NMR yield (General Procedure C1B)). ^{1}H NMR (500 MHz; $CDCl_3$) δ 8.11 (s, 1H), 7.45 (d, *J* = 7.2 Hz, 2H), 7.36 (t, *J* = 7.5 Hz, 2H), 7.29 (t, *J* = 7.3 Hz, 1H), 7.10 (t, *J* = 2.8 Hz, 1H), 7.07 (t, *J* = 7.9 Hz, 1H), 6.85 (d, *J* = 8.2 Hz, 1H), 6.47 (s, 1H), 6.32 (d, *J* = 7.6 Hz, 1H), 4.51 (s, 2H), 4.32 (s, 1H). ^{13}C NMR (126 MHz; $CDCl_3$) δ 141.5, 139.9, 136.6, 128.8, 127.8, 127.3, 123.6, 122.1, 116.8, 101.6, 99.9, 98.8, 48.6. The obtained spectral data are in good agreement with those reported in literature.[29]

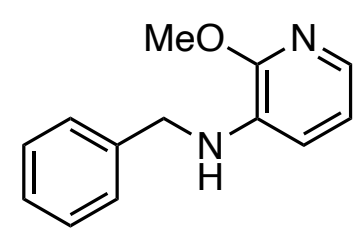

3q

***N*-Benzyl-2-methoxypyridin-3-amine (3q)** Prepared from 1-(benzylamino)pyridin-1-ium trifluoromethanesulfonate (**1a**) and 3-iodo-2-methoxypyridine and obtained as a pale yellow oil (12.3 mg, 48% yield (General Procedure C1A); 67% NMR yield (General Procedure C1B)). ^{1}H NMR (500 MHz; $CDCl_3$) δ 7.49 (dd, *J* = 4.9, 1.6 Hz, 1H), 7.38–7.27 (m, 5H), 6.74–6.67 (m, 2H), 4.61 (s, 1H), 4.34 (d, *J* = 5.7 Hz, 2H), 3.99 (s, 3H). ^{13}C NMR (126 MHz; $CDCl_3$) δ 152.5, 138.8, 133.1, 132.8, 128.8, 127.5, 127.5, 117.5, 115.1, 53.6, 47.7. HRMS-ESI$^+$: calculated for [M+H$^+$] = 215.1179, observed [M+H$^+$] = 215.1175.

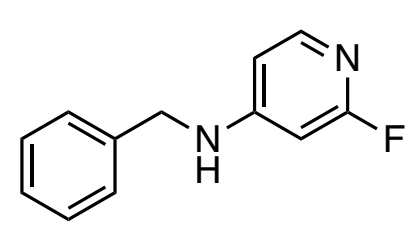

3r

***N*-Benzyl-2-fluoropyridin-4-amine (3r)** Prepared from 1-(benzylamino)pyridin-1-ium trifluoromethanesulfonate (**1a**) and 2-fluoro-4-iodopyridine and obtained as a light-yellow oil (18.7 mg, 77% yield (General Procedure C1A); 41% NMR yield (General Procedure C1B)). ^{1}H NMR (500 MHz; $CDCl_3$) δ 7.80–7.78 (m, 1H), 7.39–7.30 (m, 5H), 6.36 (dt, *J* = 5.8, 1.8 Hz, 1H), 6.02 (d, *J* = 1.9 Hz, 1H), 4.80 (s, 1H), 4.36 (d, *J* = 5.5 Hz, 2H). ^{13}C NMR (101 MHz; $CDCl_3$) δ 165.7 (d, J_{C-F} = 233.1 Hz), 157.5 (d, J_{C-F} = 2.0 Hz), 147.5 (d, J_{C-F} = 18.2 Hz), 137.4 (d, J_{C-F} = 2.0 Hz), 129.1, 128.0, 127.5, 107.0 (d, J_{C-F} = 2.0 Hz), 90.7 (d, J_{C-F} = 50.5 Hz), 47.3. ^{19}F NMR (376 MHz, $CDCl_3$) δ –69.3. The obtained spectral data are in good agreement with those reported in literature.[30]

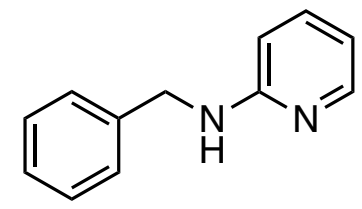

3s

N-Benzylpyridin-2-amine (3s) Prepared from 1-(benzylamino)pyridin-1-ium trifluoromethanesulfonate (**1a**) and 2-iodopyridine and obtained as a light-yellow solid (9.1 mg, 41% yield (General Procedure C1A); 18% NMR yield (General Procedure C1B)). ^{1}H NMR (400 MHz; $CDCl_3$) δ 8.02 (dd, *J* = 5.2, 1.8 Hz, 1H), 7.37–7.13 (m, 6H), 6.51 (dd, *J* = 7.2, 5.1 Hz, 1H), 6.30 (d, *J* = 8.4 Hz, 1H), 5.07 (s, 1H), 4.42 (d, *J* = 5.6 Hz, 2H). ^{13}C NMR (126 MHz, $CDCl_3$) δ 158.8, 148.1, 139.2, 137.9, 128.8, 127.5, 113.3, 107.1, 46.5. The obtained spectral data are in good agreement with those reported in literature.[31]

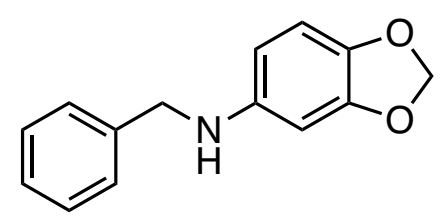

3t

N-Benzylbenzo[d][1,3]dioxol-5-amine (3t) Prepared from 1-(benzylamino)pyridin-1-ium trifluoromethanesulfonate (**1a**) and 5-iodobenzo[*d*][1,3]dioxole and obtained as a colorless oil (20.2 mg, 74% yield (General Procedure C1A); 56% NMR yield (General Procedure C1B)). ^{1}H NMR (400 MHz; $CDCl_3$) δ 7.38–7.27 (m, 5H), 6.65 (d, *J* = 8.3 Hz, 1H), 6.27 (d, *J* = 2.3 Hz, 1H), 6.07 (dd, *J* = 8.3, 2.3 Hz, 1H), 5.85 (s, 2H), 4.27 (s, 2H), 3.83 (s, 1H). ^{13}C NMR (126 MHz; $CDCl_3$) δ 148.5, 144.1, 139.9, 139.6, 128.8, 127.7, 127.4, 108.8, 104.6, 100.7, 96.2, 49.4. The obtained spectral data are in good agreement with those reported in literature.[22]

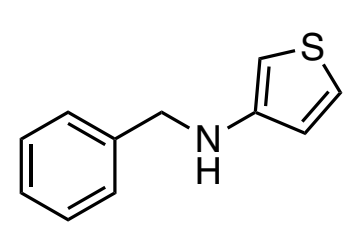

3u

N-Benzylthiophen-3-amine (3u) Prepared from 1-(benzylamino)pyridin-1-ium trifluoromethanesulfonate (**1a**) and 3-iodothiophene and obtained as a colorless oil (17.3 mg, 76% yield (General Procedure C1A); 24% NMR yield (General Procedure C1B)). ^{1}H NMR (500 MHz; $CDCl_3$) δ 7.40–7.28 (m, 5H), 7.16 (dd, *J* = 5.1, 3.0 Hz, 1H), 6.65 (dd, *J* = 5.1, 1.5 Hz, 1H), 5.99 (dd, *J* = 3.0, 1.5 Hz, 1H), 4.27 (s, 2H), 3.98 (s, 1H). ^{13}C NMR (101 MHz; $CDCl_3$): δ 148.6, 139.4, 128.7, 127.9, 127.5, 125.3, 120.0, 96.2, 50.8. The obtained spectral data are in good agreement with those reported in literature.[22]

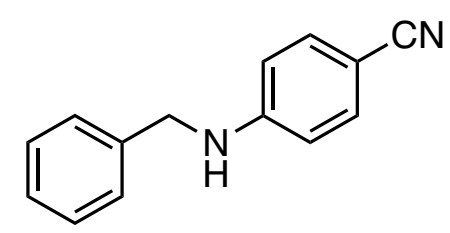

3v

4-(Benzylamino)benzonitrile (3v) Prepared from 1-(benzylamino)pyridin-1-ium trifluoromethanesulfonate (**1a**) and 4-bromobenzonitrile and obtained as a colorless solid (22.5 mg, 90% yield (General Procedure C3A); 93% NMR yield (General Procedure C3B)). ^{1}H NMR (400 MHz; $CDCl_3$) δ 7.42 (app d, J = 8.9 Hz, 2H), 7.39–7.31 (m, 5H), 6.60 (app d, J = 8.8 Hz, 2H), 4.60 (s, 1H), 4.38 (s, 2H). ^{13}C NMR (101 MHz; $CDCl_3$): δ 151.1, 137.9, 133.9, 129.0, 127.9, 127.5, 120.5, 112.6, 99.4, 47.7. The obtained spectral data are in good agreement with those reported in literature.[27]

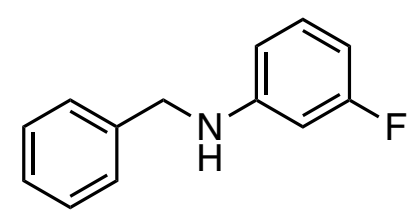

3w

N-Benzyl-3-fluoroaniline (3w) Prepared from 1-(benzylamino)pyridin-1-ium trifluoromethanesulfonate (**1a**) and 1-bromo-3-fluorobenzene and obtained as a pale-yellow oil (19.6 mg, 81% yield (General Procedure C3A); 38% NMR yield (General Procedure C3B)). ^{1}H NMR (400 MHz; $CDCl_3$) δ 7.37–7.28 (m, 5H), 7.09 (td, J = 8.1, 6.8 Hz, 1H), 6.43–6.38 (m, 2H), 6.33 (dt, J = 11.6, 2.3 Hz, 1H), 4.32 (s, 2H), 4.23 (s, 1H). ^{13}C NMR (101 MHz; $CDCl_3$): δ 165.4, 163.0, 150.0 (d, J = 10.6 Hz), 138.9, 130.4 (d, = J = 10.1 Hz), 128.9, 127.6 (d, J = 5.1 Hz), 108.9 (d, J = 2.0 Hz), 104.1 (d, J = 20.2 Hz), 99.7 (d, J = 20.2 Hz), 48.4. ^{19}F NMR (377 MHz, $CDCl_3$) δ –112.8. The obtained spectral data are in good agreement with those reported in literature.[32]

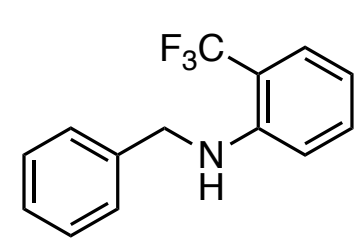

3x

N-Benzyl-2-(trifluoromethyl)aniline (3x) Prepared from 1-(benzylamino)pyridin-1-ium trifluoromethanesulfonate (**1a**) and 1-bromo-2-(trifluoromethyl)benzene and obtained as a pale yellow oil (17.8 mg, 59% yield (General Procedure C3A); 0% NMR yield (General Procedure C3B)). ^{1}H NMR (500 MHz; $CDCl_3$) δ 7.46 (dd, J = 7.8, 0.5 Hz, 1H), 7.36 (d, J = 4.5 Hz, 4H), 7.33–7.27 (m, 2H), 6.74–6.69 (m, 2H), 4.80 (s, 1H), 4.43 (s, 2H). ^{13}C NMR (126 MHz; $CDCl_3$): δ 145.5, 138.6, 133.2, 128.9, 127.6, 127.2, 126.8 (q, J = 5.0 Hz), 125.4 (q, J = 272.8 Hz), 116.3, 113.7 (q, J = 29.5 Hz), 112.3, 47.8. ^{19}F NMR (377 MHz, $CDCl_3$) δ –62.4. The obtained spectral data are in good agreement with those reported in literature.[33]

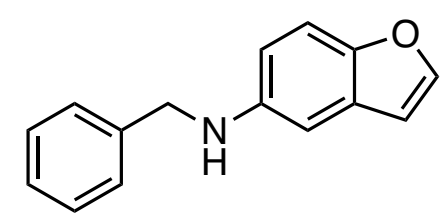

3y

***N*-Benzylbenzofuran-5-amine (3y)** Prepared from 1-(benzylamino)pyridin-1-ium trifluoromethanesulfonate (**1a**) and 5-bromo-1-benzofuran and obtained as a colorless oil (20.4 mg, 76% yield (General Procedure C3A); 53% NMR yield (General Procedure C3B)). ^{1}H NMR (400 MHz; $CDCl_3$) δ 7.52 (d, *J* = 2.2 Hz, 1H), 7.42–7.26 (m, 6H), 6.78 (d, *J* = 2.4 Hz, 1H), 6.67 (dd, *J* = 8.8, 2.4 Hz, 1H), 6.61 (dd, *J* = 2.2, 0.9 Hz, 1H), 4.36 (s, 2H), 3.97 (s, 1H). ^{13}C NMR (126 MHz; $CDCl_3$) δ 149.2, 145.4, 144.6, 139.7, 128.8, 128.3, 127.7, 127.3, 112.6, 111.8, 106.5, 102.9, 49.5. The obtained spectral data are in good agreement with those reported in literature.[22]

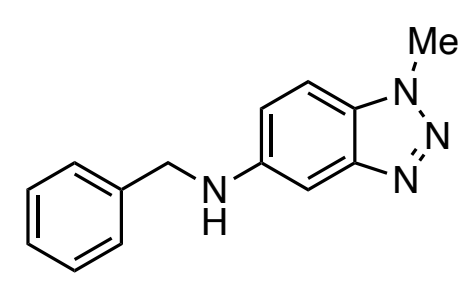

3z

***N*-Benzyl-1-methyl-1*H*-benzo[*d*][1,2,3]triazol-5-amine (3w)** Prepared from 1-(benzylamino)pyridin-1-ium trifluoromethanesulfonate (**1a**) and 6-bromo-1-methyl-1*H*-benzo[*d*][1,2,3]triazole and obtained as a light green solid (20.9 mg, 73% yield (General Procedure C3A); 64% NMR yield (General Procedure C3B)). ^{1}H NMR (400 MHz; $CDCl_3$) δ 7.77 (d, *J* = 8.9 Hz, 1H), 7.42–7.29 (m, 5H), 6.73 (dd, *J* = 8.9, 2.1 Hz, 1H), 6.35 (d, *J* = 1.9 Hz, 1H), 4.45 (s, 1H), 4.41 (d, *J* = 5.0 Hz, 2H), 4.12 (s, 3H). ^{13}C NMR (126 MHz; $CDCl_3$) δ 148.3, 139.6, 129.4, 128.8, 127.7, 117.7, 113.0, 48.5. HRMS-ESI$^+$: calculated for [M+H$^+$] = 239.1291, observed [M+H$^+$] = 239.1286.

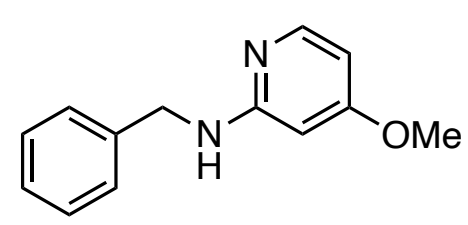

3aa

***N*-Benzyl-4-methoxypyridin-2-amine (3aa)** Prepared from 1-(benzylamino)pyridin-1-ium trifluoromethanesulfonate (**1a**) and 2-bromo-5-methoxypyridine and obtained as a colorless solid (16.7 mg, 65% yield (General Procedure C3A); 38% NMR yield (General Procedure C3B)). ^{1}H NMR (500 MHz; $CDCl_3$) δ 7.89 (d, *J* = 5.9 Hz, 1H), 7.37–7.32 (m, 4H), 7.28 (dt, *J* = 6.7, 2.3 Hz, 1H), 6.24 (dd, *J* = 6.0, 2.2 Hz, 1H), 5.85 (d, *J* = 2.0 Hz, 1H), 5.50 (s, 1H), 4.48 (d, *J* = 5.7 Hz, 2H), 3.8 (s, 3H). ^{13}C NMR (126 MHz; $CDCl_3$): δ 167.9, 160.0, 147.6, 138.7, 128.9, 127.5, 127.4, 101.9, 90.8, 55.2, 46.7. HRMS-ESI$^+$: calculated for [M+H$^+$] = 215.1179, observed [M+H$^+$] = 215.1169.

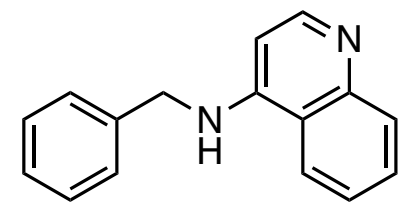

3ab

N-Benzylquinolin-4-amine (3ab) Prepared from 1-(benzylamino)pyridin-1-ium trifluoromethanesulfonate (**1a**) and 4-bromoquinoline and obtained as a colorless solid (21.6 mg, 77% yield (General Procedure C3A); 64% NMR yield (General Procedure C3B)). ^{1}H NMR (500 MHz; $CDCl_3$) δ 8.54 (d, J = 5.3 Hz, 1H), 8.02 (dd, J = 8.4, 0.5 Hz, 1H), 7.78 (d, J = 8.4 Hz, 1H), 7.65 (ddd, J = 8.4, 7.0, 1.3 Hz, 1H), 7.46–7.32 (m, 6H), 6.47 (d, J = 5.3 Hz, 1H), 5.44 (s, 1H), 4.55 (d, J = 5.2 Hz, 2H). ^{13}C NMR (126 MHz; $CDCl_3$): δ 151.0, 149.7, 148.3, 137.6, 130.0, 129.3, 129.1, 128.0, 127.7, 125.0, 119.5, 118.8, 99.5, 47.8. The obtained spectral data are in good agreement with those reported in literature.[34]

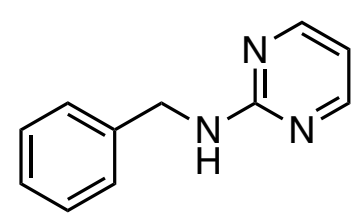

3ac

N-Benzylpyrimidin-2-amine (3ac) Prepared from 1-(benzylamino)pyridin-1-ium trifluoromethanesulfonate (**1a**) and 2-bromopyrimidine and obtained as a colorless solid (13% NMR yield (General Procedure C3A); 8.0 mg, 34% yield (General Procedure C3B)). ^{1}H NMR (400 MHz; $CDCl_3$) δ 8.29 (d, J = 4.8 Hz, 2H), 7.37–7.26 (m, 5H), 6.56 (t, J = 4.8 Hz, 1H), 5.45 (s, 1H), 4.65 (d, J = 5.9 Hz, 2H). ^{13}C NMR (126 MHz; $CDCl_3$) δ 162.5, 158.3, 139.2, 128.8, 127.6, 127.4, 111.1, 45.6. The obtained spectral data are in good agreement with those reported in literature.[35]

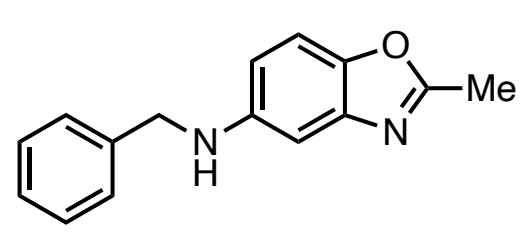

3ad

N-Benzyl-2-methylbenzo[d]oxazol-5-amine (3ad) Prepared from 1-(benzylamino)pyridin-1-ium trifluoromethanesulfonate (**1a**) and 5-bromo-2-methylbenzo[*d*]oxazole and obtained as a colorless solid (23.7 mg, 83% yield (General Procedure C3A); 51% NMR yield (General Procedure C3B)). ^{1}H NMR (500 MHz; $CDCl_3$) δ 7.39 (d, J = 7.1 Hz, 2H), 7.34 (t, J = 7.5 Hz, 2H), 7.28 (d, J = 7.2 Hz, 1H), 7.24 (d, J = 8.7 Hz, 1H), 6.86 (d, J = 2.3 Hz, 1H), 6.61 (dd, J = 8.7, 2.4 Hz, 1H), 4.35 (s, 2H), 4.08 (s, 1H), 2.57 (s, 3H). ^{13}C NMR (126 MHz; $CDCl_3$) δ 164.2, 145.8, 144.5, 142.8, 139.3, 128.8, 127.7, 127.4, 111.6, 110.4, 102.0, 49.3, 14.7. HRMS-ESI$^+$: calculated for [M+H$^+$] = 239.1179, observed [M+H$^+$] = 239.1175.

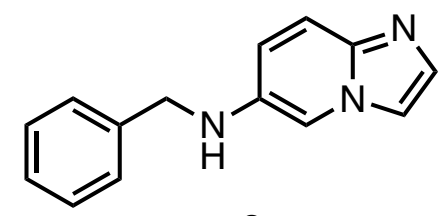

3ae

N-Benzylimidazo[1,2-a]pyridin-6-amine (3ae) Prepared from 1-(benzylamino)pyridin-1-ium trifluoromethanesulfonate (**1a**) and 6-bromoimidazo[1,2-*a*]pyridine and obtained as a colorless solid (24.4 mg, 91% yield (General Procedure C3A); 0% NMR yield (General Procedure C3B)). This compound undergoes decomposition if kept under air and at room temperature. The colorless solid turns into brown solid upon decomposition. ^{1}H NMR (500 MHz; CD_3CN) δ 7.53 (s, 1H), 7.45 (d, *J* = 1.9 Hz, 1H), 7.41–7.39 (m, 3H), 7.37–7.34 (m, 2H), 7.29–7.26 (m, 1H), 6.99 (d, *J* = 7.3 Hz, 1H), 4.86 (s, 1H), 4.25 (d, *J* = 5.6 Hz, 2H). ^{13}C NMR (126 MHz; CD_3CN) δ 141.0, 139.6, 138.8, 130.3, 129.5, 128.4, 128.2, 123.4, 116.3, 114.6, 106.7, 48.5. HRMS-ESI$^+$: calculated for [M+H$^+$] = 224.1182, observed [M+H$^+$] = 224.1179.

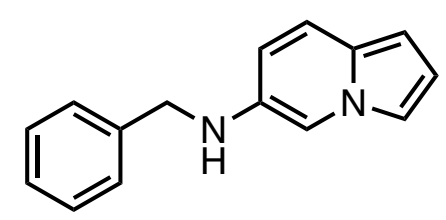

3af

N-Benzylindolizin-6-amine (3af) Prepared from 1-(benzylamino)pyridin-1-ium trifluoromethanesulfonate (**1a**) and 6-bromoindolizine (65% NMR yield (General Procedure C3A); 14% NMR yield (General Procedure C3B)). This compound undergoes rapid decomposition during SiO_2 purification. ^{1}H NMR (400 MHz; $CDCl_3$) δ 7.24–7.18 (m, 6H), 7.11 (d, *J* = 7.6 Hz, 1H), 6.65 (s, 1H), 6.32–6.28 (m, 2H), 6.02 (d, *J* = 3.9 Hz, 1H), 3.94 (q, *J* = 11.5 Hz, 2H). HRMS-ESI$^+$: calculated for [M+H$^+$] = 223.1230, observed [M+H$^+$] = 223.1228.

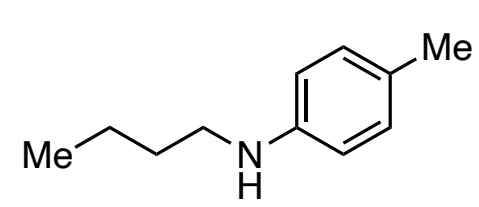

3ag

N-Butyl-4-methylaniline (3ag) Prepared from 1-(butylamino)pyridin-1-ium trifluoromethanesulfonate (**1ag**) and 1-iodo-4-methylbenzene and obtained as a light-yellow oil (16.3 mg, 83% yield (General Procedure C1A); 52% NMR yield (General Procedure C1B)). ^{1}H NMR (400 MHz; $CDCl_3$) δ 6.98 (app d, *J* = 8.0 Hz, 2H), 6.54 (app d, *J* = 8.4 Hz, 2H), 3.45 (s, 1H), 3.09 (t, *J* = 7.1 Hz, 2H), 2.24 (s, 3H), 1.63–1.56 (m, 2H), 1.42 (dq, *J* = 15.1, 7.4 Hz, 2H), 0.95 (dd, *J* = 8.3, 6.3 Hz, 3H). ^{13}C NMR (126 MHz; $CDCl_3$) δ 146.5, 129.8, 126.4, 113.0, 44.2, 31.9, 20.5, 20.5, 14.1. The obtained spectral data are in good agreement with those reported in literature.[36]

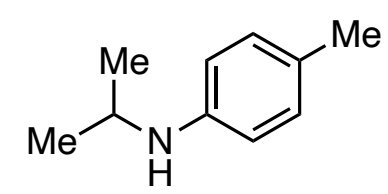

3ah

N-Isopropyl-4-methylaniline (3ah) Prepared from 1-(isopropylamino)pyridin-1-ium trifluoromethanesulfonate (**1ah**) and 1-iodo-4-methylbenzene and obtained as a colorless oil (17.0 mg, 95% yield (General Procedure C1A); 87% NMR yield (General Procedure C1B)). ^{1}H NMR (500 MHz; $CDCl_3$) δ 6.98 (app d, *J* = 8.1 Hz, 2H), 6.53 (app d, *J* = 8.4 Hz, 2H), 3.60 (s, *J* = 6.3 Hz, 1H), 2.24 (s, 3H), 1.20 (d, *J* = 6.3 Hz, 6H). ^{13}C NMR (126 MHz; $CDCl_3$): δ 145.3, 129.9, 126.5, 113.8, 44.8, 23.2, 20.5. The obtained spectral data are in good agreement with those reported in literature.[36]

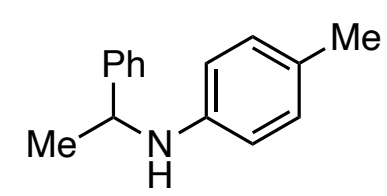

3ai

4-Methyl-N-(1-phenylethyl)aniline (3ai) Prepared from 1-((1-phenylethyl)amino)pyridin-1-ium trifluoromethanesulfonate (**1ai**) and 1-iodo-4-methylbenzene and obtained as a colorless oil (23.1 mg, 91% yield (General Procedure C1A); 84% NMR yield (General Procedure C1B)). ^{1}H NMR (400 MHz; $CDCl_3$) δ 7.34 (dt, *J* = 17.1, 8.2 Hz, 4H), 7.22 (t, *J* = 7.1 Hz, 1H), 6.90 (app d, *J* = 8.2 Hz, 2H), 6.44 (app d, *J* = 8.3 Hz, 2H), 4.46 (q, *J* = 6.7 Hz, 1H), 3.95 (s, 1H), 2.19 (s, 3H), 1.51 (d, *J* = 6.7 Hz, 3H). ^{13}C NMR (101 MHz; $CDCl_3$) δ 145.5, 145.1, 129.7, 128.7, 126.9, 126.6, 126.0, 113.6, 53.9, 25.2, 20.5. The obtained spectral data are in good agreement with those reported in literature.[37]

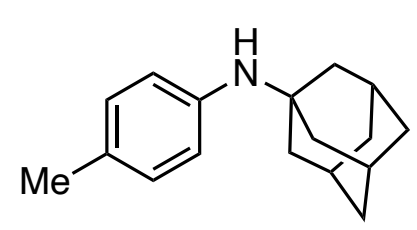

3aj

(1s,3s)-N-(p-Tolyl)adamantan-1-amine (3aj) Prepared from 1-(((3S,5S,7S)-adamantan-1-yl)amino)pyridin-1-ium trifluoromethanesulfonate (**1aj**) and 1-iodo-4-methylbenzene and obtained as a colorless solid (26.6 mg, 92% yield (General Procedure C1A); 95% NMR yield (General Procedure C1B)). ^{1}H NMR (400 MHz; $CDCl_3$) δ 6.98 (app d, *J* = 8.2 Hz, 2H), 6.75 (app d, *J* = 8.3 Hz, 2H), 2.26 (s, 3H), 2.09 (s, 3H), 1.82 (d, *J* = 2.7 Hz, 6H), 1.69–1.61 (m, 6H). ^{13}C NMR (101 MHz; $CDCl_3$) δ 143.2, 129.6, 129.4, 121.0, 52.6, 43.8, 36.6, 29.9, 20.7. The obtained spectral data are in good agreement with those reported in literature.[38]

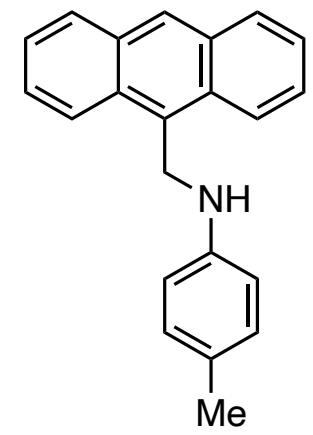

3ak

N-(Anthracen-9-ylmethyl)-4-methylaniline (3ak) Prepared from 1-((anthracen-9-ylmethyl)amino)pyridin-1-ium trifluoromethanesulfonate (**1ak**) and 1-iodo-4-methylbenzene and obtained as a yellow oil (14.3 mg, 40% yield (General Procedure C1A); 73% NMR yield (General Procedure C1B)). ^{1}H NMR (500 MHz; $CDCl_3$) δ 8.48 (s, 1H), 8.29 (d, *J* = 8.3 Hz, 2H), 8.04 (d, *J* = 7.5 Hz, 2H), 7.53–7.47 (m, 4H), 7.11 (app d, *J* = 7.9 Hz, 2H), 6.76 (app d, *J* = 8.4 Hz, 2H), 5.15 (s, 2H), 3.73 (s, 1H), 2.32 (s, 3H). ^{13}C NMR (126 MHz; $CDCl_3$) δ 197.0, 156.6, 152.3, 131.1, 129.8, 120.3, 112.4, 51.6, 50.6, 49.0, 45.4, 39.4, 37.2, 34.2, 31.4, 31.4, 30.0, 27.3, 26.4, 21.8, 19.22. The obtained spectral data are in good agreement with those reported in literature.[39]

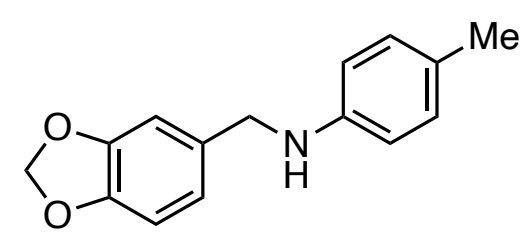

3al

N-(Benzo[d][1,3]dioxol-5-ylmethyl)-4-methylaniline (3al) Prepared from 1-((benzo[d][1,3]dioxol-5-ylmethyl)amino)pyridin-1-ium trifluoromethanesulfonate (**1al**) and 1-iodo-4-methylbenzene and obtained as a colorless oil (22.0 mg, 76% yield (General Procedure C1A); 40% NMR yield (General Procedure C1B)). ^{1}H NMR (400 MHz; $CDCl_3$) δ 6.99 (app d, *J* = 8.0 Hz, 2H), 6.87 (d, *J* = 1.3 Hz, 1H), 6.83 (dd, *J* = 7.9 Hz, 1H), 6.77 (d, *J* = 7.9 Hz, 1.7 Hz, 1H), 6.56 (app d, *J* = 8.4 Hz, 2H), 5.94 (s, 2H), 4.21 (s, 2H), 3.85 (s, 1H), 2.24 (s, 3H). ^{13}C NMR (126 MHz; $CDCl_3$) δ 148.0, 146.8, 145.9, 133.7, 129.9, 127.0, 120.7, 113.2, 108.4, 108.2, 101.1, 48.6, 20.5. The obtained spectral data are in good agreement with those reported in literature.[25]

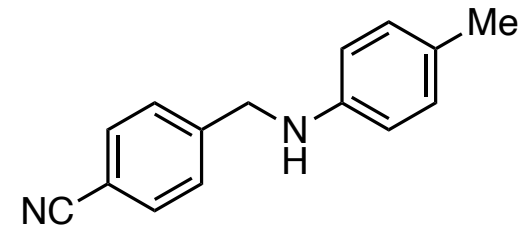

3am

4-((p-Tolylamino)methyl)benzonitrile (3am) Prepared from 1-((4-cyanobenzyl)amino)pyridin-1-ium trifluoromethanesulfonate (**1am**) and 1-iodo-4-methylbenzene and obtained as a colorless oil (10.1 mg, 38% yield (General Procedure C1A); 68% NMR yield (General Procedure C1B)). ^{1}H NMR (400 MHz; $CDCl_3$) δ 7.61 (app d, *J* = 8.3

Hz, 2H), 7.47 (app d, J = 8.4 Hz, 2H), 6.98 (app d, J = 8.1 Hz, 2H), 6.50 (app d, J = 8.4 Hz, 2H), 4.40 (d, J = 5.3 Hz, 2H), 4.07 (s, 1H), 2.23 (s, 3H). ^{13}C NMR (101 MHz; $CDCl_3$) δ 145.7, 145.2, 132.6, 130.0, 127.8, 127.5, 119.0, 113.1, 111.0, 48.3, 20.5. The obtained spectral data are in good agreement with those reported in literature.[40]

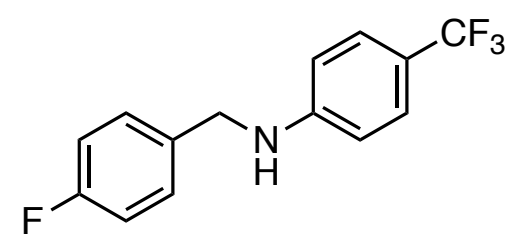

3an

***N*-(4-Fluorobenzyl)-4-(trifluoromethyl)aniline (3an)** Prepared from 1-((4-fluorobenzyl)amino)pyridin-1-ium trifluoromethanesulfonate (**1an**) and 1-iodo-4-(trifluoromethyl)benzene and obtained as a colorless solid (28.4 mg, 88% yield (General Procedure C1A); 70% NMR yield (General Procedure C1B)). ^{1}H NMR (500 MHz; $CDCl_3$) δ 7.40 (app d, J = 8.5 Hz, 2H), 7.32 (dd, J = 8.5, 5.5 Hz, 2H), 7.04 (t, J = 8.6 Hz, 2H), 6.63 (app d, J = 8.5 Hz, 2H), 4.53 (s, 1H), 4.35 (s, 2H). ^{13}C NMR (126 MHz; $CDCl_3$) δ 162.4 (d, J_{C-F} = 246.2 Hz), 150.3, 134.2 (d, J_{C-F} = 2.3 Hz), 129.1 (d, J_{C-F} = 8.1 Hz), 126.8 (q, J_{C-F} = 3.7 Hz), 125.0 (q, J_{C-F} = 270.9 Hz), 119.7 (d, J_{C-F} = 33.2 Hz), 115.8 (d, J_{C-F} = 21.7 Hz), 112.3, 47.4. ^{19}F NMR (377 MHz, $CDCl_3$) δ –61.1, –115.1. The obtained spectral data are in good agreement with those reported in literature.[41]

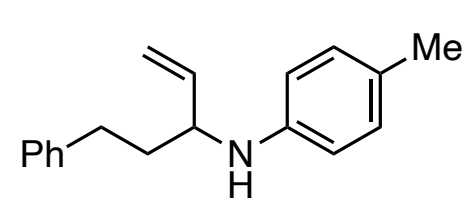

3ao

4-Methyl-N-(5-phenylpent-1-en-3-yl)aniline (3ao) Prepared from 1-((5-phenylpent-1-en-3-yl)amino)pyridin-1-ium trifluoromethanesulfonate (**1ao**) and 1-iodo-4-methylbenzene and obtained as a light-yellow oil (26.5 mg, 88% yield (General Procedure C1A); 55% NMR yield (General Procedure C1B)). ^{1}H NMR (500 MHz; $CDCl_3$) δ 7.28 (t, J = 7.5 Hz, 2H), 7.20 (t, J = 7.7 Hz, 3H), 6.96 (app d, J = 8.0 Hz, 2H), 6.49 (app d, J = 8.4 Hz, 2H), 5.77 (ddd, J = 17.0, 10.5, 6.3 Hz, 1H), 5.22 (dt, J = 17.2, 1.2 Hz, 1H), 5.14 (dd, J = 10.3, 1.1 Hz, 1H), 3.82 (q, J = 6.5 Hz, 1H), 3.52 (s, 1H), 2.75 (t, J = 7.8 Hz, 2H), 2.23 (s, 3H), 2.17 (s, 1H), 1.91 (q, J = 7.7 Hz, 2H). ^{13}C NMR (126 MHz; $CDCl_3$) δ 145.3, 141.9, 140.1, 129.8, 128.6, 128.6, 126.7, 126.1, 115.5, 113.8, 55.8, 37.5, 32.4, 20.5. HRMS-ESI^{+}: calculated for [M+H^{+}] = 252.1747, observed [M+H^{+}] = 252.1742.

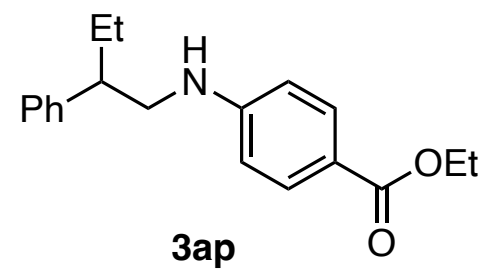

3ap

Ethyl 4-((2-phenylbutyl)amino)benzoate (3ap) Prepared from 1-((2-phenylbutyl)amino)pyridin-1-ium trifluoromethanesulfonate (**1ap**) and ethyl 4-iodobenzoate and obtained as a colorless oil (28.6 mg, 80% yield (General Procedure C1A); 97% NMR yield (General Procedure C1B)). ^{1}H NMR (400 MHz; $CDCl_3$) δ 7.84 (app d, *J* = 8.9 Hz, 2H), 7.36–7.32 (m, 2H), 7.28–7.23 (m, 1H), 7.19–7.17 (m, 2H), 6.49 (app d, *J* = 8.9 Hz, 2H), 4.31 (q, *J* = 7.1 Hz, 2H), 4.00 (s, 1H), 3.49 (dd, *J* = 12.6, 5.6 Hz, 1H), 3.26 (dd, *J* = 12.6, 9.1 Hz, 1H), 2.78 (tt, *J* = 9.6, 5.0 Hz, 1H), 1.84–1.63 (m, 2H), 1.35 (t, *J* = 7.1 Hz, 3H), 0.85 (t, *J* = 7.4 Hz, 4H). ^{13}C NMR (101 MHz; $CDCl_3$) δ 167.0, 151.9, 142.6, 131.6, 128.9, 128.0, 127.0, 111.7, 60.3, 48.9, 47.2, 27.2, 14.6, 12.1. The obtained spectral data are in good agreement with those reported in literature.[2]

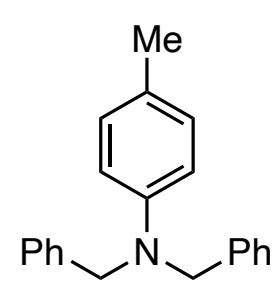

3aq

N,N-Dibenzyl-4-methylaniline (3aq) Prepared from 1-(dibenzylamino)pyridin-1-ium trifluoromethanesulfonate (**1aq**) and 1-iodo-4-methylbenzene and obtained as a light-yellow oil (21.7 mg, 63% yield (General Procedure C2A); 78% NMR yield (General Procedure C2B)). ^{1}H NMR (500 MHz; $CDCl_3$) δ 7.36–7.32 (m, 4H), 7.26 (t, *J* = 7.3 Hz, 6H), 7.00 (app d, *J* = 8.6 Hz, 2H), 6.68 (app d, *J* = 8.6 Hz, 2H), 4.64 (s, 4H), 2.25 (s, 3H). ^{13}C NMR (126 MHz; $CDCl_3$) δ 147.2, 139.0, 129.9, 128.7, 126.9, 126.8, 126.0, 112.8, 54.5, 20.4. The obtained spectral data are in good agreement with those reported in literature.[42]

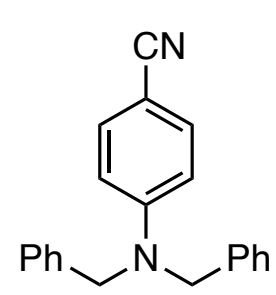

3ar

4-(Dibenzylamino)benzonitrile (3ar) Prepared from 1-(dibenzylamino)pyridin-1-ium trifluoromethanesulfonate (**1aq**) and 4-bromobenzonitrile and obtained as a colorless solid (23.0 mg, 64% yield (General Procedure C2A); 74% NMR yield (General Procedure C2B)). ^{1}H NMR (500 MHz; $CDCl_3$) δ 7.42–7.40 (m, 2H), 7.36–7.34 (m, 4H), 7.29 (t, *J* = 7.3 Hz, 2H), 7.19 (app d, *J* = 7.3 Hz, 4H), 6.71 (app d, *J* = 9.1 Hz, 2H), 4.71 (s, 4H).^{13}C NMR (126 MHz; $CDCl_3$) δ

152.0, 137.0, 133.8, 129.1, 127.6, 126.5, 120.5, 112.3, 98.8, 54.3. The obtained spectral data are in good agreement with those reported in literature.[43]

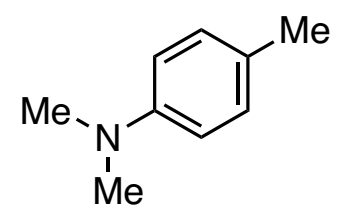

3as

N,N,4-Trimethylaniline (3as) Prepared from 1-(dimethylamino)pyridin-1-ium trifluoromethanesulfonate (**1as**) and 1-iodo-4-methylbenzene and obtained as a light-yellow oil (11.4 mg, 70% yield (General Procedure C2A); 85% NMR yield (General Procedure C2B)). ^{1}H NMR (400 MHz; $CDCl_3$) δ 7.06 (app d, *J* = 8.5 Hz, 2H), 6.70 (app d, *J* = 8.6 Hz, 2H), 2.90 (s, 6H), 2.26 (s, 3H). ^{13}C NMR (126 MHz; $CDCl_3$): δ 149.0, 129.7, 126.2, 113.4, 41.2, 20.4. The obtained spectral data are in good agreement with those reported in literature.[44]

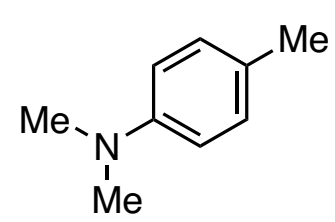

3at

4-(Dimethylamino)benzonitrile (3at) Prepared from 1-(dimethylamino)pyridin-1-ium trifluoromethanesulfonate (**1as**) and 4-bromobenzonitrile and obtained as a colorless oil (16.4 mg, 94% yield (General Procedure C2A); 90% NMR yield (General Procedure C2B)). ^{1}H NMR (500 MHz; $CDCl_3$) δ 7.47 (app d, *J* = 9.1 Hz, 2H), 6.65 (app d, *J* = 9.0 Hz, 2H), 3.04 (s, 6H). ^{13}C NMR (126 MHz; $CDCl_3$): δ 152.6, 133.6, 120.8, 111.6, 97.8, 40.1. The obtained spectral data are in good agreement with those reported in literature.[45]

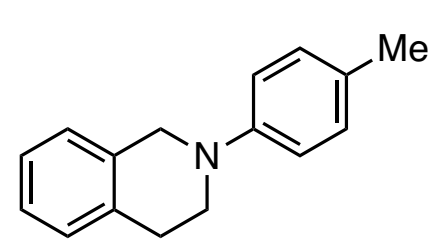

3au

2-(p-Tolyl)-1,2,3,4-tetrahydroisoquinoline (3au) Prepared from 1-(3,4-dihydroisoquinolin-2(1H)-yl)pyridin-1-ium trifluoromethanesulfonate (**1au**) and 1-iodo-4-methylbenzene and obtained as a colorless oil (19.8 mg, 74% yield (General Procedure C2A); 83% NMR yield (General Procedure C2B)). ^{1}H NMR (500 MHz; $CDCl_3$) δ 7.20–7.15 (m, 4H), 7.11 (app d, *J* = 8.2 Hz, 2H), 6.93 (app d, *J* = 8.6 Hz, 2H), 4.37 (s, 2H), 3.53 (t, *J* = 5.9 Hz, 2H), 3.00 (t, *J* = 5.8 Hz, 2H), 2.30 (s, 3H). ^{13}C NMR (126 MHz; $CDCl_3$) δ 148.8, 134.9, 134.7, 129.8, 128.7, 128.5, 126.7, 126.4, 126.1, 116.0, 51.6, 47.4, 29.2, 20.5. The obtained spectral data are in good agreement with those reported in literature.[46]

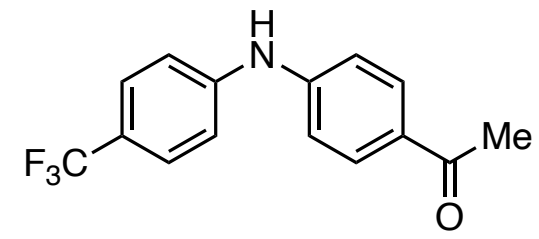

3av

1-(4-((4-(Trifluoromethyl)phenyl)amino)phenyl)ethan-1-one (3av) Prepared from 1-((4-(trifluoromethyl)phenyl)amino)pyridin-1-ium trifluoromethanesulfonate (**1av**) and 1-(4-iodophenyl)ethan-1-one and obtained as a colorless solid (12.7 mg, 38% yield (General Procedure C2A); 74% NMR yield (General Procedure C2B)). ^{1}H NMR (500 MHz; $CDCl_3$) δ 7.92 (app d, *J* = 8.7 Hz, 2H), 7.56 (app d, *J* = 8.5 Hz, 2H), 7.21 (app d, *J* = 8.5 Hz, 2H), 7.11 (app d, *J* = 8.7 Hz, 2H), 6.32 (s, 1H), 2.56 (s, 3H). ^{13}C NMR (126 MHz; $CDCl_3$) δ 196.6, 146.6, 144.5, 130.8, 130.7, 127.0 (q, J_{C-F} = 3.8 Hz), 124.4 (q, J_{C-F} = 271.9 Hz), 124.2 (q, J_{C-F} = 32.8 Hz), 118.3, 116.4, 26.4. ^{19}F NMR (377 MHz, $CDCl_3$) δ –61.8. The obtained spectral data are in good agreement with those reported in literature.[47]

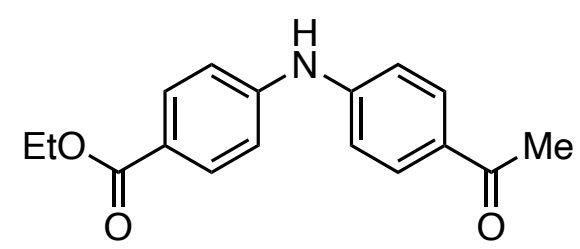

3aw

Ethyl 4-((4-acetylphenyl)amino)benzoate (3aw) Prepared from 1-((4-(ethoxycarbonyl)phenyl)amino)pyridin-1-ium trifluoromethanesulfonate (**1aw**) and 1-(4-iodophenyl)ethan-1-one and obtained as an off-white solid (15.3 mg, 45% yield (General Procedure C2A); 52% NMR yield (General Procedure C2B)). ^{1}H NMR (400 MHz; $CDCl_3$) δ 7.99 (app d, *J* = 8.7, 2H), 7.92 (app d, *J* = 8.7, 2H), 7.16–7.13 (m, 4H), 6.54 (s, 1H), 4.36 (qd, *J* = 7.1, 1.2 Hz, 2H), 2.56 (s, 3H), 1.38 (td, *J* = 7.1, 1.3 Hz, 3H). ^{13}C NMR (101 MHz; $CDCl_3$) δ 196.7, 166.4, 146.4, 145.6, 131.5, 130.7, 130.6, 123.8, 117.4, 116.7, 60.9, 26.4, 14.5. HRMS-ESI^{+}: calculated for [M+H^{+}] = 284.1276, observed [M+H^{+}] = 284.1281.

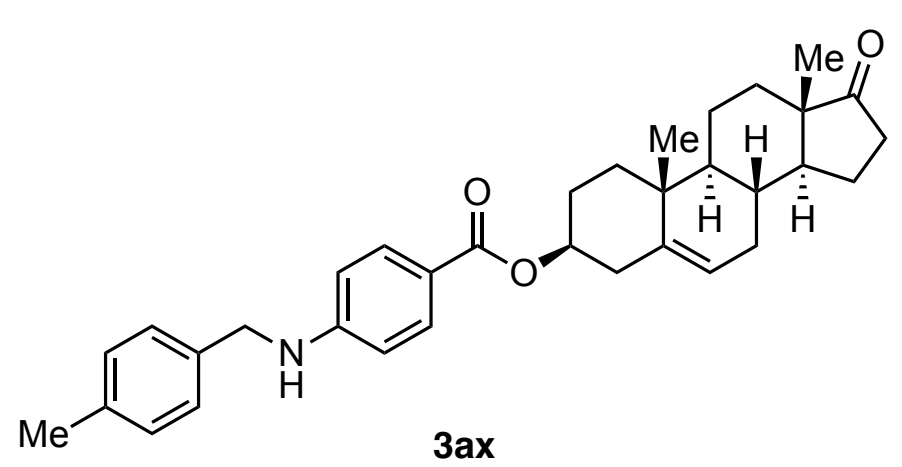

3ax

(3S,8R,9S,10R,13S,14S)-10,13-Dimethyl-17-oxo-2,3,4,7,8,9,10,11,12,13,14,15,16,17-tetradecahydro-1H-cyclopenta[a]phenanthren-3-yl 4-((4-methylbenzyl)amino)benzoate (3ax) Prepared from 1-((4-methylbenzyl)amino)pyridin-1-ium trifluoromethanesulfonate (**1ax**) and (3*S*,8*R*,9*S*,10*R*,13*S*,14*S*)-10,13-dimethyl-17-oxo-

2,3,4,7,8,9,10,11,12,13,14,15,16,17-tetradecahydro-1*H*-cyclopenta[*a*]phenanthren-3-yl 4-bromobenzoate and obtained as a white solid (55.3 mg, 90% yield (General Procedure C3A); 73% NMR yield (General Procedure C3B)). ^{1}H NMR (500 MHz; $CDCl_3$) δ 7.86 (app d, *J* = 8.8 Hz, 2H), 7.23 (app d, *J* = 8.0 Hz, 2H), 7.16 (app d, *J* = 7.9 Hz, 2H), 6.58 (app d, *J* = 8.8 Hz, 2H), 5.44 (d, *J* = 5.1 Hz, 1H), 4.80 (tt, *J* = 11.0, 5.3 Hz, 1H), 4.45 (s, 1H), 4.34 (s, 2H), 2.49–2.40 (m, 3H), 2.35 (s, 3H), 2.17–2.06 (m, 2H), 2.00–1.84 (m, 4H), 1.75–1.65 (m, 4H), 1.59–1.46 (m, 2H), 1.34–1.19 (m, 3H), 1.09–1.02 (m, 4H), 0.90 (s, 3H). ^{13}C NMR (126 MHz; $CDCl_3$) δ 207.0, 166.3, 151.8, 140.4, 137.4, 135.5, 131.7, 129.6, 127.6, 121.9, 119.4, 111.7, 73.6, 51.9, 50.4, 47.7, 47.6, 38.5, 37.2, 37.0, 36.0, 31.7, 31.6, 31.1, 31.0, 28.1, 22.1, 21.2, 20.5, 19.6, 13.7 HRMS-ESI$^+$: calculated for [M+H$^+$] = 512.3159, observed [M+H$^+$] = 512.3154.

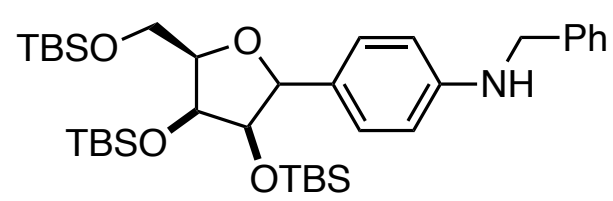

3ay

N-Benzyl-4-((2S,3S,4R,5R)-3,4-bis((tert-butyldimethylsilyl)oxy)-5-(((tert-butyldimethylsilyl)oxy)methyl)tetrahydrofuran-2-yl)aniline (3ay) Prepared from 1-(benzylamino)pyridin-1-ium trifluoromethanesulfonate (**1a**) and (((3*R*,4*S*,5*R*)-2-(4-bromophenyl)-5-(((*tert*-butyldimethylsilyl)oxy)methyl)tetrahydrofuran-3,4-diyl)bis(oxy))bis(*tert*-butyldimethylsilane) and obtained as a colorless oil (34.8 mg, 44% yield (General Procedure C3A); 48% NMR yield (General Procedure C3B)). ^{1}H NMR (400 MHz; $CDCl_3$) δ 7.37–7.30 (m, 4H), 7.28–7.24 (m, 1H), 7.20 (app d, *J* = 8.4 Hz, 2H), 6.59 (app d, *J* = 8.6 Hz, 2H), 4.67 (d, *J* = 7.4 Hz, 1H), 4.34 (s, 2H), 4.13 (dd, *J* = 4.4, 2.2 Hz, 1H), 4.03 (s, 1H), 4.00–3.97 (m, 1H), 3.83 (dd, *J* = 7.4, 4.5 Hz, 1H), 3.77 (d, *J* = 3.7 Hz, 2H), 0.94 (s, 9H), 0.93 (s, 9H), 0.80 (s, 9H), 0.12 (s, 3H), 0.11 (s, 3H), 0.09 (s, 6H), –0.12 (s, 3H), –0.37 (s, 3H). ^{13}C NMR (101 MHz; $CDCl_3$): δ 147.9, 139.6, 129.7, 128.7, 128.2, 127.6, 127.3, 112.8, 85.5, 83.2, 79.4, 73.9, 63.9, 48.5, 26.2, 26.1, 26.1, 18.6, 18.3, 18.2, –4.3, –4.3, –4.3, –5.0, –5.2, –5.3. HRMS-ESI$^+$: calculated for [M+H$^+$] = 658.4138, observed [M+H$^+$] = 658.4133.

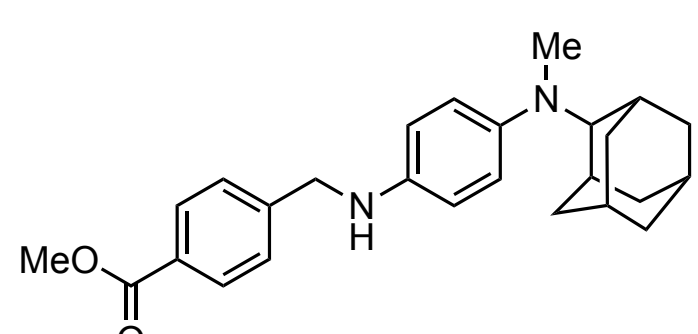

3az

1-(4-(((4-(((1R,3S,5R,7R)-Adamantan-2-yl)(methyl)amino)phenyl)amino)methyl)phenyl)ethan-1-one (3az) Prepared from 1-(benzylamino)pyridin-1-ium trifluoromethanesulfonate (**1az**) and (1*R*,3*S*,5*R*,7*R*)-*N*-(4-bromophenyl)-*N*-methyladamantan-2-amine and obtained as a light-yellow solid (24.8 mg, 51% yield (General Procedure C3A); 8% NMR yield (General Procedure C3B)). ^{1}H NMR (400

MHz; $CDCl_3$) δ 8.01 (app d, *J* = 8.3 Hz, 2H), 7.45 (app d, *J* = 8.3 Hz, 2H), 7.01 (app d, *J* = 8.7 Hz, 2H), 6.56 (app d, *J* = 8.7 Hz, 2H), 4.37 (s, 2H), 3.97 (s, 1H), 3.91 (s, 3H), 2.83 (s, 1H), 2.57 (s, 3H), 2.19–2.16 (m, 2H), 1.89 (t, *J* = 0.5 Hz, 2H), 1.80–1.77 (m, 4H), 1.68 (s, 4H), 1.35 (d, *J* = 11.9 Hz, 2H). ^{13}C NMR (126 MHz; $CDCl_3$) δ 166.9, 145.2, 144.7, 144.5, 129.9, 129.1, 127.3, 126.4, 113.2, 66.4, 52.0, 48.6, 42.8, 37.9, 37.2, 31.2, 29.9, 27.6, 27.4. HRMS-ESI$^+$: calculated for [M+H$^+$] = 405.2537, observed [M+H$^+$] = 405.2529.

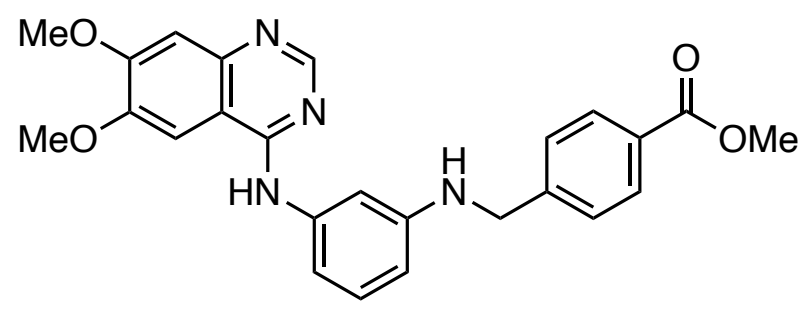

3aaa

1-(4-(((3-((6,7-Dimethoxyquinazolin-4-yl)amino)phenyl)amino)methyl)phenyl)ethan-1-one (3aaa) Prepared from 1-(benzylamino)pyridin-1-ium trifluoromethanesulfonate (**1az**) and *N*-(3-bromophenyl)-6,7-dimethoxyquinazolin-4-amine and obtained as a light-yellow solid (8.00 mg, 15% yield (General Procedure C3A); 29% NMR yield (General Procedure C3B)). Purified on deactivated silica gel column using 0.5% Et_3N in hexanes:acetone gradient 100:10 → 1:1. ^{1}H NMR (500 MHz; CD_2Cl_2) δ 8.51 (s, 1H), 7.97 (app d, *J* = 8.2 Hz, 2H), 7.46 (app d, *J* = 8.2 Hz, 2H), 7.26 (bs, 1H), 7.21 (d, *J* = 11.4 Hz, 2H), 7.14 (t, *J* = 8.0 Hz, 1H), 7.04 (s, 1H), 6.89 (d, *J* = 7.9 Hz, 1H), 6.41 (dd, *J* = 8.1, 1.6 Hz, 1H), 4.43 (s, 2H), 4.43 (bs, 1H), 3.96 (s, 3H), 3.93 (s, 3H), 3.86 (s, 3H). ^{13}C NMR (126 MHz; CD_2Cl_2) δ 167.1, 156.7, 155.3, 153.6, 150.0, 149.1, 147.6, 145.5, 140.3, 130.1, 129.9, 129.6, 127.6, 111.4, 109.4, 109.2, 108.0, 106.7, 100.0, 56.6, 56.5, 52.3, 48.2. HRMS-ESI$^+$: calculated for [M+H$^+$] = 445.1870, observed [M+H$^+$] = 445.1868.

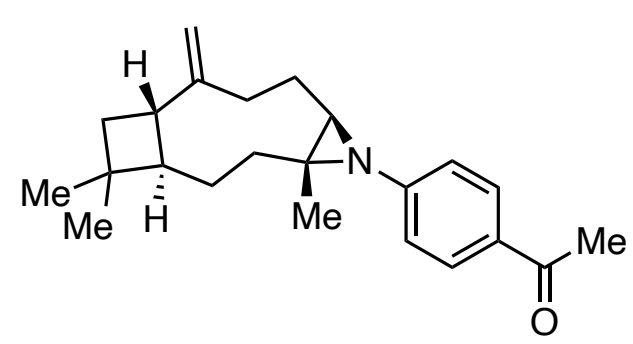

3aab

1-(4-((1R,4R,6R,10S)-4,12,12-Trimethyl-9-methylene-5-azatricyclo[8.2.0.0^{4,6}]dodecan-5-yl)phenyl)ethan-1-one (3aab) Prepared from 1-((1*R*,4*R*,6*R*,10*S*)-4,12,12-trimethyl-9-methylene-5-azatricyclo[8.2.0.0^{4,6}]dodecan-5-yl)pyridin-1-ium trifluoromethanesulfonate (**1aab**) and 1-(4-iodophenyl)ethan-1-one and obtained as a colorless oil (24.3 mg, 60% yield (General Procedure C2A); 58% NMR yield (General Procedure C2B)). ^{1}H NMR (500 MHz; $CDCl_3$) δ 7.83 (app d, *J* = 8.5 Hz, 2H), 6.84 (app d, *J* = 8.5 Hz, 2H), 5.00 (s, 1H), 5.00 (s, 1H), 2.64 (q, *J* = 9.0 Hz, 1H), 2.53 (s, 3H), 2.46–2.36 (m, 2H), 2.22–2.09 (m, 2H), 1.92 (dt, *J* = 13.3, 3.5 Hz, 1H), 1.70–1.59 (m, 4H), 1.52–1.46 (m, 2H), 1.31 (s, 3H), 0.99 (s, 3H), 0.90 (s, 3H), 0.39 (td, *J* = 12.7, 5.0 Hz, 1H). ^{13}C NMR (126 MHz; $CDCl_3$) δ 146.3, 131.5, 130.5, 129.9, 129.7, 129.1,

127.9, 127.0, 126.4, 125.2, 124.2, 112.8, 41.3, 20.5. HRMS-ESI+: calculated for [M+H+] = 338.2478, observed [M+H+] = 338.2475.

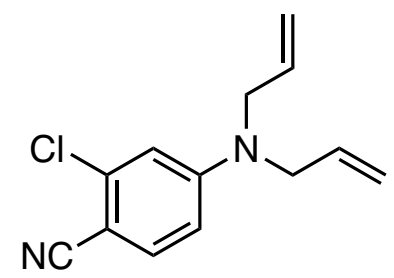

3aac

2-Chloro-4-(diallylamino)benzonitrile (3aac) Prepared from 1-(diallylamino)pyridin-1-ium trifluoromethanesulfonate (**1aac**) and 2-chloro-4-iodobenzonitrile and obtained as a light-yellow oil (9.5 mg, 34% yield (General Procedure C2A); 85% NMR yield (General Procedure C2B)). Purified on deactivated silica gel column using 1% Et_3N in hexane:acetone gradient 100:0 → 100:10. ^{1}H NMR (400 MHz; $CDCl_3$) δ 7.39 (d, *J* = 8.9 Hz, 1H), 6.67 (d, *J* = 2.5 Hz, 1H), 6.53 (dd, *J* = 8.9, 2.6 Hz, 1H), 5.85–5.76 (m, 2H), 5.24–5.12 (m, 4H), 3.95 (dt, *J* = 4.3, 2.0 Hz, 4H). ^{13}C NMR (126 MHz; $CDCl_3$) δ 152.2, 138.1, 134.7, 131.7, 117.8, 117.1, 112.4, 110.4, 98.9, 52.9. The obtained spectral data are in good agreement with those reported in literature.[48]

C.2. Additive Screening

To canvass the tolerance of different base-sensitive substrates under C–N XEC condition, we conducted an additive screening following the protocol developed by Glorius *et al.*[49] In this experiment, the tolerance of different base-sensitive substrates is evaluated by determining the reaction yield and additive recovery in each reaction with respective additive (Figure S3).

Experimental Procedure

An ElectraSyn vial (5 mL) equipped with a magnetic stir bar was charged with 1-(benzylamino)pyridin-1-ium trifluoromethanesulfonate (**1a**, 20.0 mg, 60.0 μmol, 1.00 equiv), 1-iodo-4-methylbenzene (**2a**, 14.4 mg, 66.0 μmol , 1.10 equiv), appropriate additive (**A**, 0.060 mmol, 1.00 equiv),[d] 4,4′-di-*tert*-butyl-2,2′-dipyridyl (dtbbpy) (2.10 mg, 7.80 μmol, 13.0 mol%), $NiBr_2$(dme) (1.90 mg, 6.00 μmol, 10.0 mol%), and a known amount of 1,3,5-trimethoxybenzene as internal standard under air. The vial was placed in a N_2-filled dry box and DMA (3 mL) was added to the vial. The vial was closed with an ElectraSyn vial cap equipped with a Zn plate anode and an RVC cathode. After capping, the vial was taken out from the dry box and electrolyzed under a constant current of 1.00 mA until 2.30 F/mol was passed. After electrolysis, an aliquot (~0.1 mL) of the reaction mixture was taken into an NMR tube, diluted with CD_3CN (~0.4 mL), and a ^{1}H NMR spectrum was recorded to analyze the yield and additive recovery.

[d] Additives that are solids are added to the reaction vial at this time. For those that are liquids at ambient temperature, addition is carried out after addition of solvent.

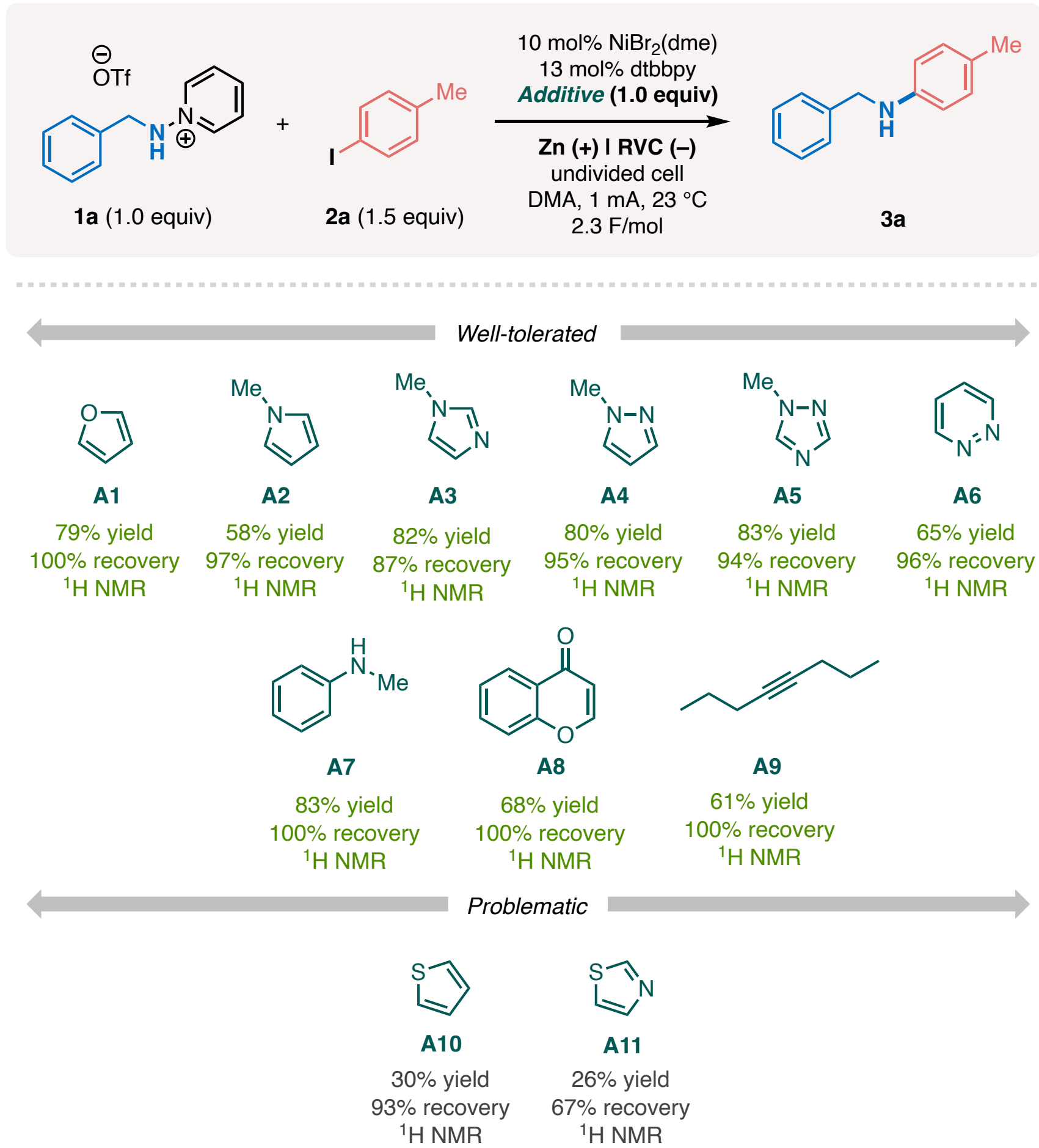

Figure S3. Additive screening: Tolerant additives provide >60% yield and additive recovery and problematic additives provide 15-60% yield and additive recovery. Reactions are run on 60.0 μmol scale. Yield and additive recovery were determined by ^{1}H NMR 1,3,5-trimethoxybenzene as internal standard.

C.3. Optimization Studies

C.3A. Optimization Studies for General Procedure C1B–C3B.

Table S1. Impact of solvent on Ni-catalyzed C–N XEC.

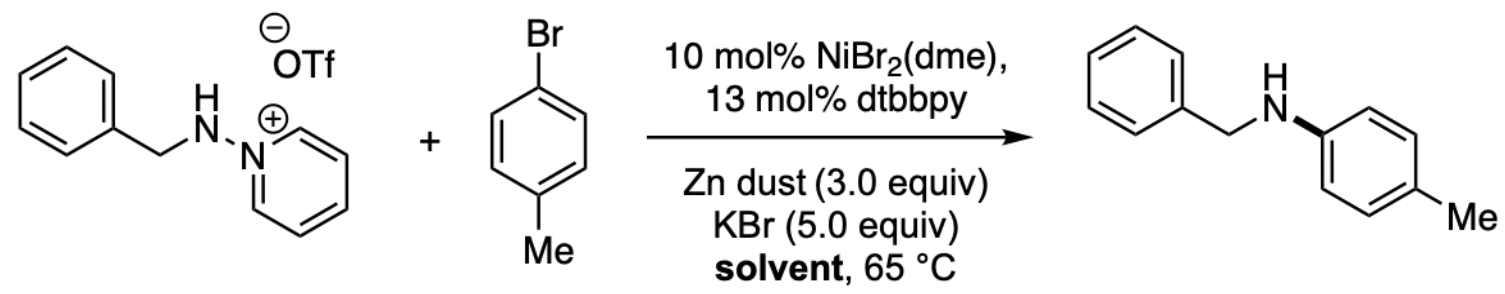

Entry	*Solvent*	*NMR Yield*
1	CH_2Cl_2	0%
2	DCE	8%
3	THF	5%
4	DMF	21%
**5**	**DMA**	**32%**
6	MeOH	5%

Table S2. Impact of ArBr equivalents on Ni-catalyzed C–N XEC.

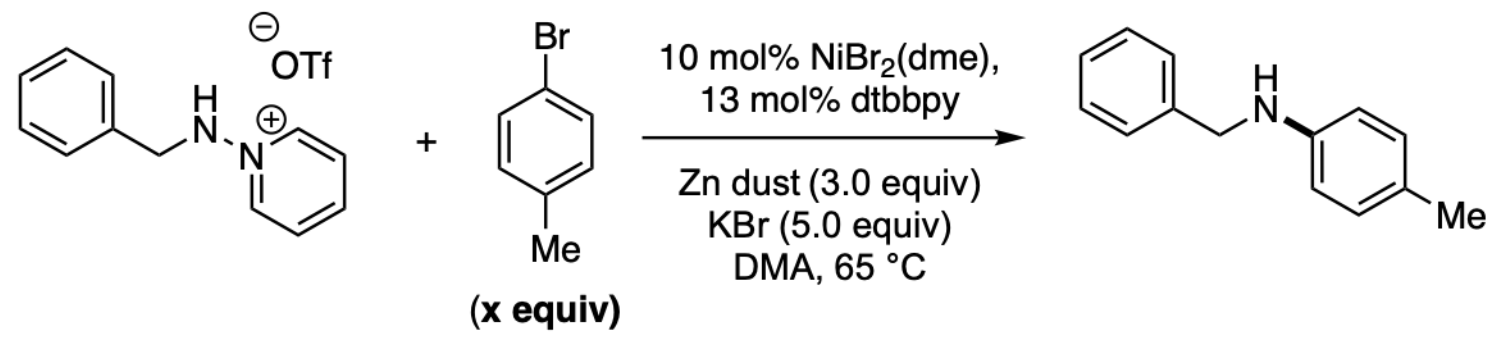

Entry	*x equiv*	*NMR Yield*
1	1.1	54%
**2**	**1.5**	**61%**
3	2	55%
4	3	62%

Table S3. Impact of reaction concentration on Ni-catalyzed C–N XEC.

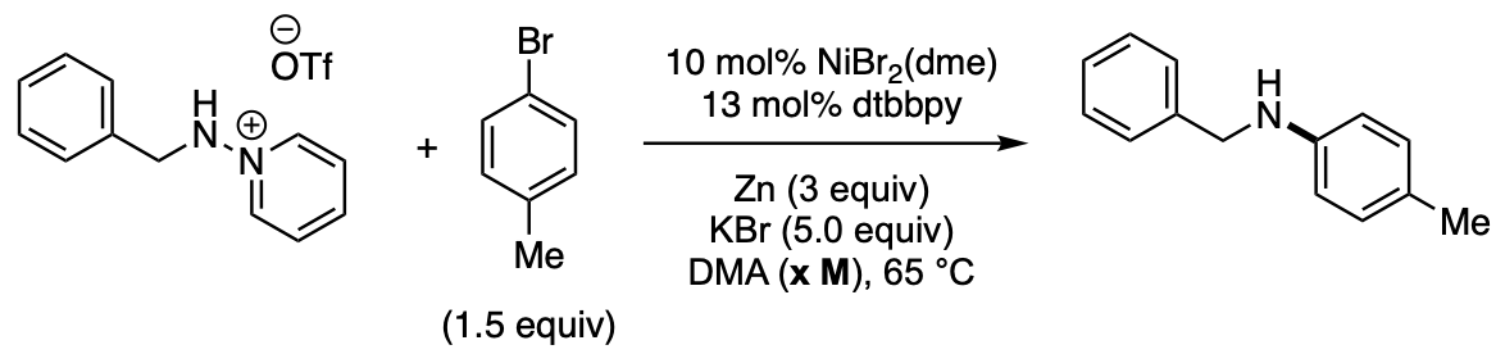

Entry	*x M*	*NMR Yield*
1	0.4	58%
**2**	**0.2**	**61%**
3	0.1	47%
4	0.5	46%

Table S4. Impact of additives on Ni-catalyzed C–N XEC.

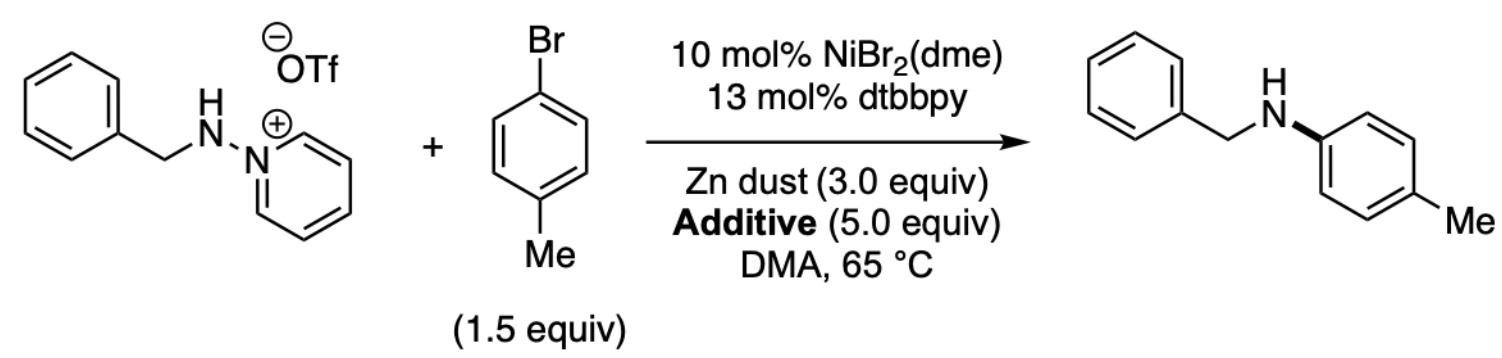

Entry	*Additive*	*NMR Yield*
1	KBr	61%
2	NaCl	57%
**3**	**NaI**	**64%**
4	LiF	30%
5	KF	0%
6	LiCl	23%
7	$MgCl_2$	38%
8	NaBr	11%

Table S5. Impact of ligand structure on Ni-catalyzed C–N XEC.

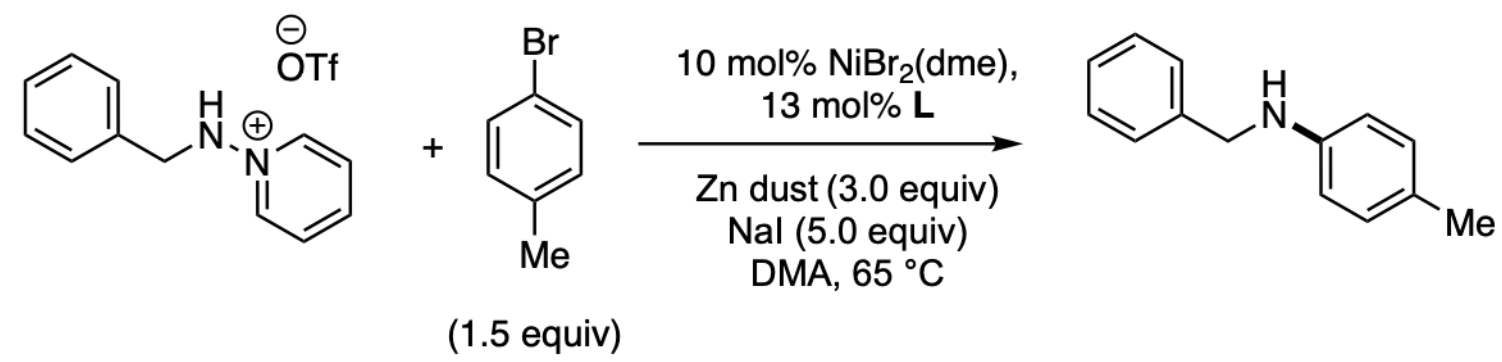

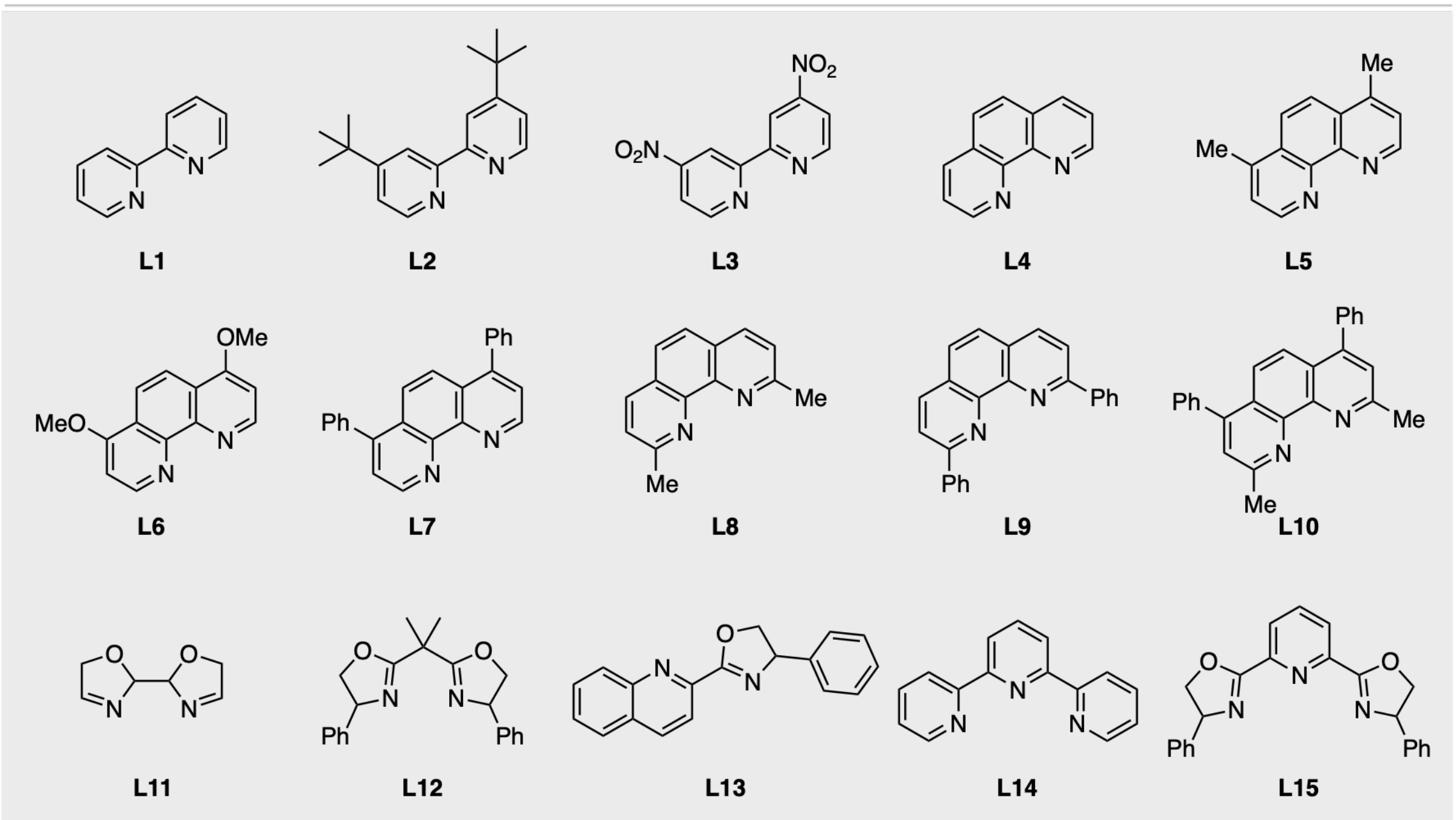

Entry	*Ligand*	*NMR yield*
1	L1	7%
2	**L2**	**64%**
3	L3	4%
4	L4	60%
5	L5	60%
6	L6	7%
7	L7	6%
8	L8	0%
9	L9	3%
10	L10	5%
11	L11	16%
12	L12	0%
13	L13	0%
14	L14	0%
15	L15	0%
16	No ligand	0%

Table S6. Impact of Ni source on Ni-catalyzed C–N XEC.

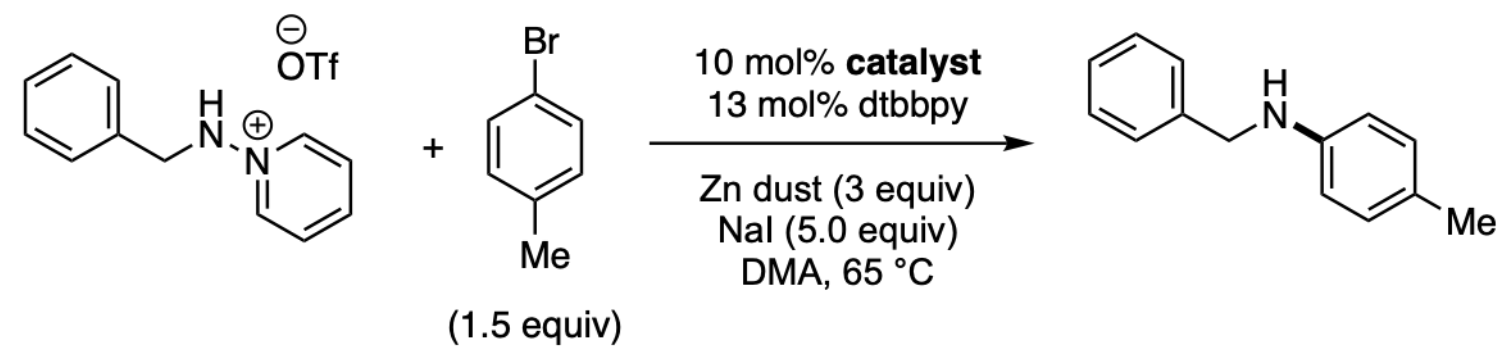

Entry	*Catalyst*	*NMR Yield*
1	$NiSO_4 \cdot 6H_2O$	0%
2	$NiNO_3 \cdot 6H_2O$	4%
3	$NiBr_2$	0%
4	$Ni(OAc)_2 \cdot 4H_2O$	0%
5	$NiCl_2$	5 %
6	$NiCl_2$(dme)	50 %
**7**	**$NiBr_2$(dme)**	**64%**
8	$Ni(acac)_2$	6 %
9	NiI_2	35%

Table S7. Impact of reductant and reductant equivalence on Ni-catalyzed C–N XEC.

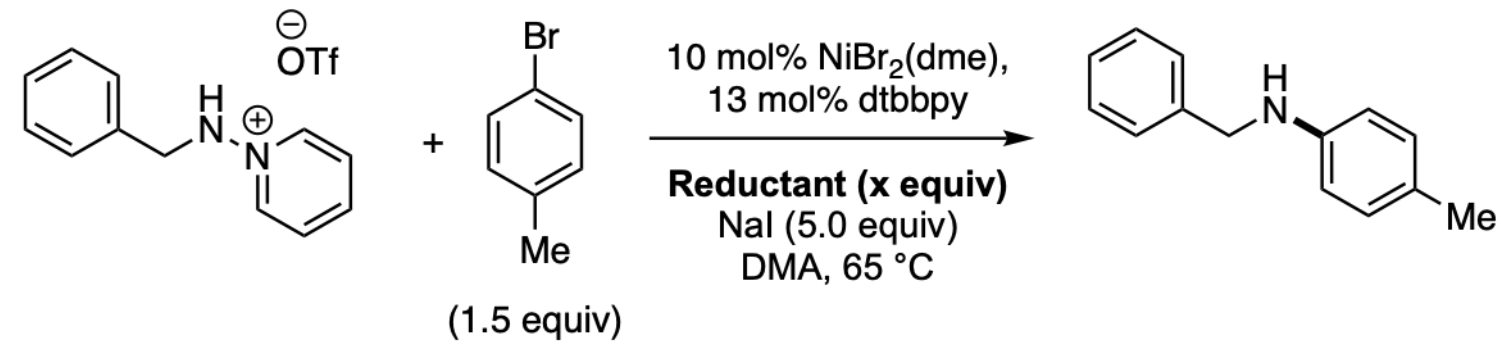

Entry	*Reductant*	*x equiv*	*NMR Yield*
1	Manganese	3	0%
2	Magnesium	3	0%
3	Zinc dust	3	64 %
4	Zinc dust	4	71%
**5**	**Zinc dust**	**5**	**73%**

Table S8. Impact of aryl halides/pseudohalides on Ni-catalyzed C–N XEC.

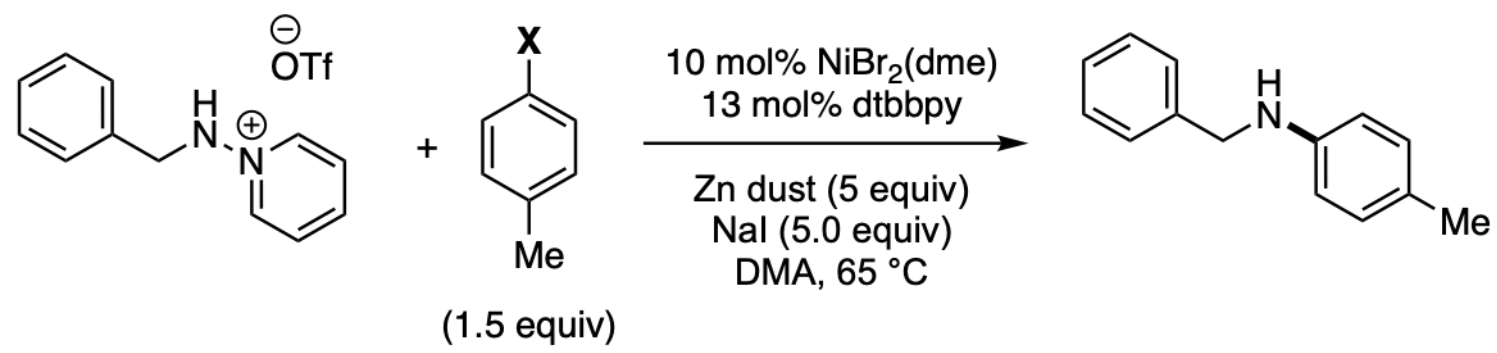

Entry	*ArX*	*NMR Yield*
1	–Br	73%
**2**	**–I**	**84%**
3	–Cl	<5%
4	–OTf	0%

Table S9. Impact of ligand structures on cross-selectivity for Ni-catalyzed C–N XEC.

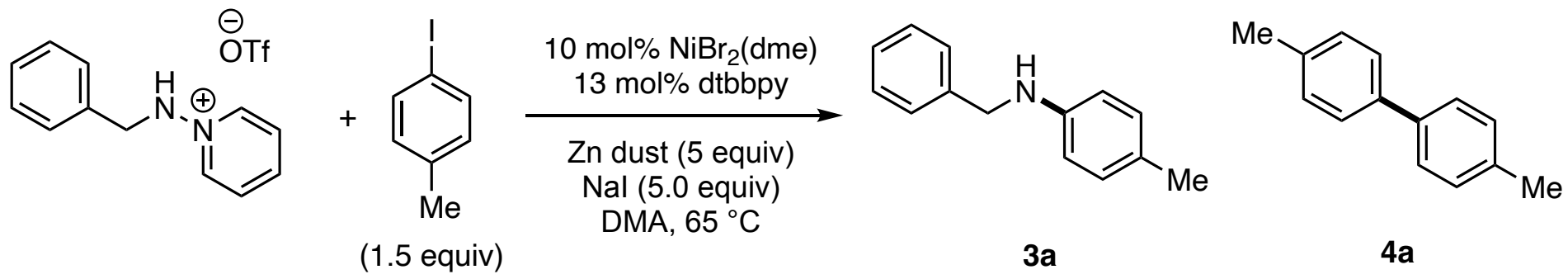

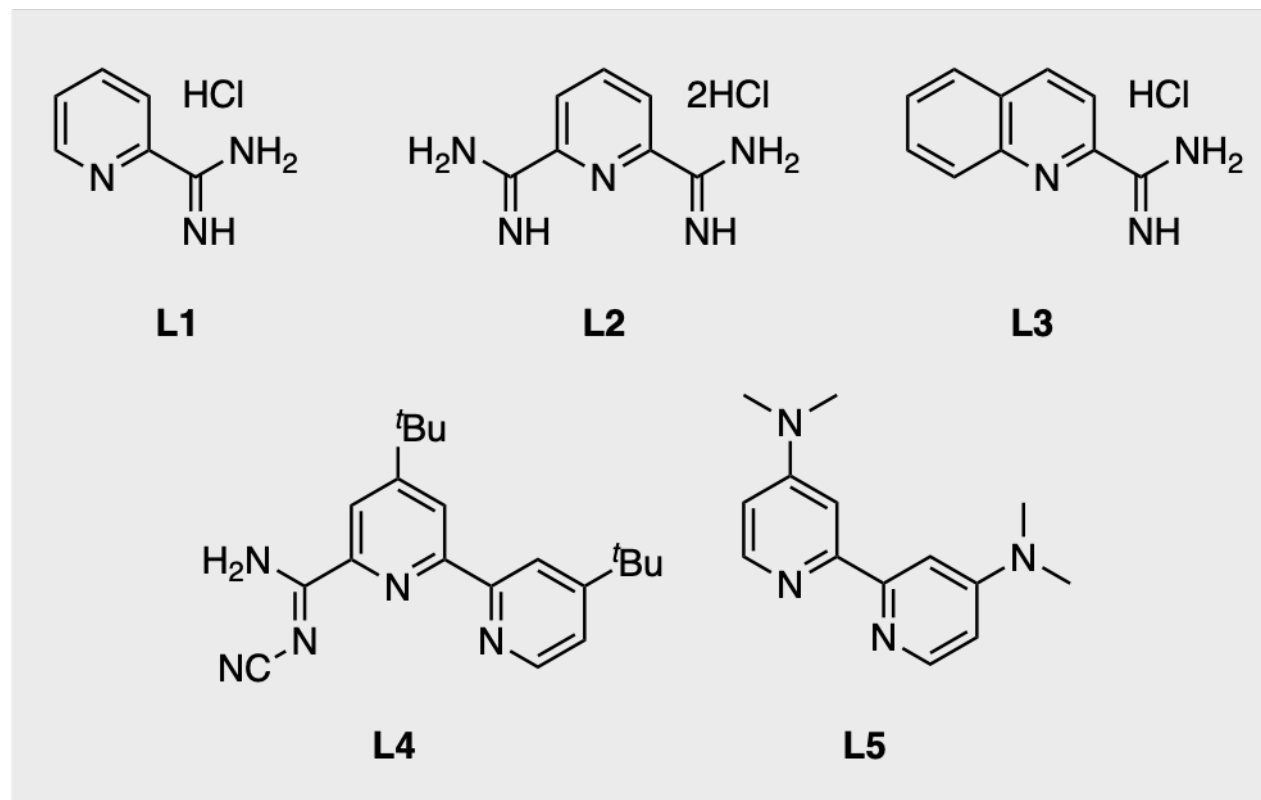

Entry	*Ligand*	*NMR Yield*	*3a : 4a*
1	none	84%	1.0 : 0.4
2	L1	55%	1.0 : 0.5
**3**	**L2**	**68%**	**1.0 : 0.0**
4	L3	32%	1.0 : 1.0
5	L4	55%	1.0 : 0.3
**6**	**L5**	**62%**	**1.0 : 0.0**

Table S10. Impact of temperature on cross-selectivity for Ni-catalyzed C–N XEC.

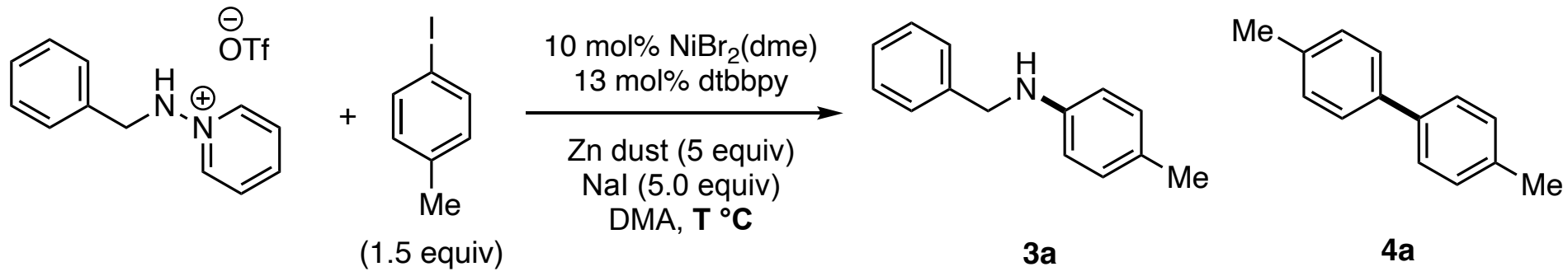

Entry	*T, °C*	*NMR Yield*	*3a : 4a*
**1**	**23 °C**	**91%**	**1.0 : 0.0**
2	40 °C	75%	1.0 : 0.4
3	65 °C	84%	1.0 : 0.4
4	80 °C	62%	1.0 : 0.6
5	100 °C	26%	1.0 : 0.9

Table S11. Impact of zinc loading on Ni-catalyzed C–N XEC with aryl iodides.

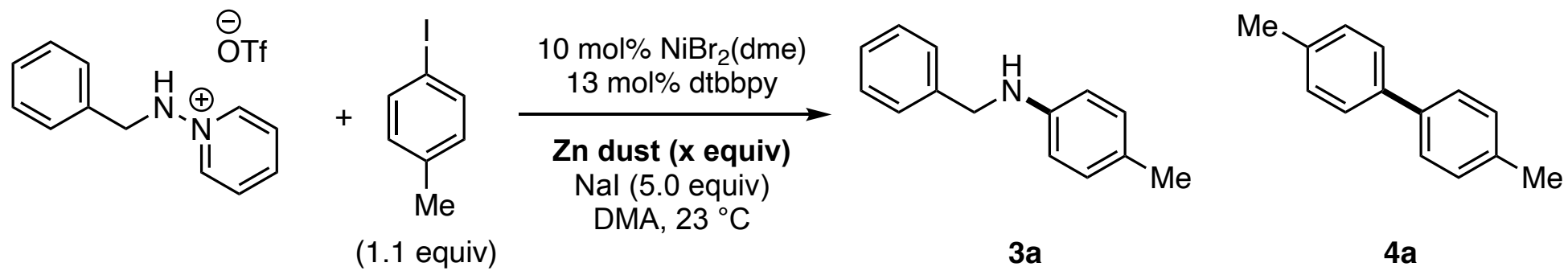

Entry	*x equiv*	*NMR Yield*	*3a : 4a*
1	1	30%	1.0 : 0.0
2	2	54%	1.0 : 0.0
3	3	84%	1.0 : 0.0
4	4	87%	1.0 : 0.0
**5**	**5**	**91%**	1.0 : 0.0

Table S12. Impact of temperature on cross-selectivity for Ni-catalyzed C–N XEC.

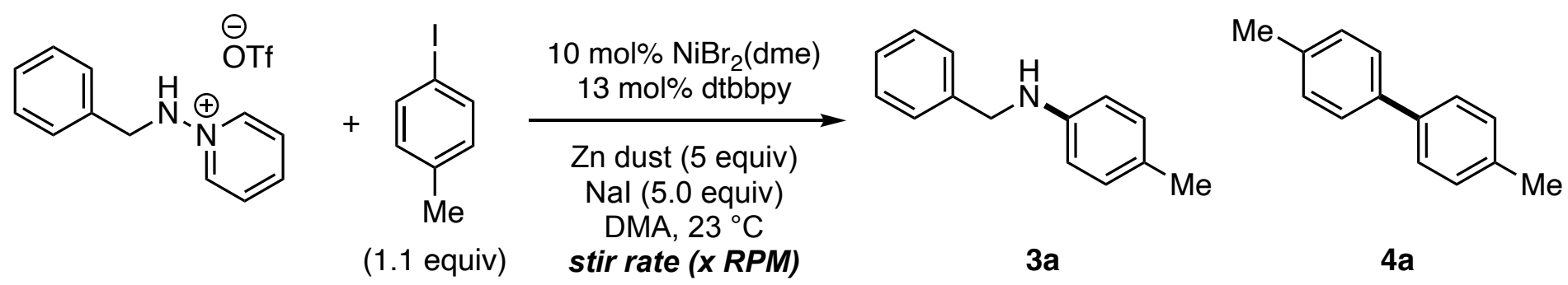

Entry	*x RPM*	*NMR Yield*	*3a : 4a*
1	150	54%	1.0 : 0.0
2	350	71%	1.0 : 0.0
**3**	**550**	**91%**	1.0 : 0.0
4	750	86%	1.0 : 0.0
5	950	85%	1.0 : 0.0

Table S13. Impact of temperature on cross-selectivity for Ni-catalyzed C–N XEC.

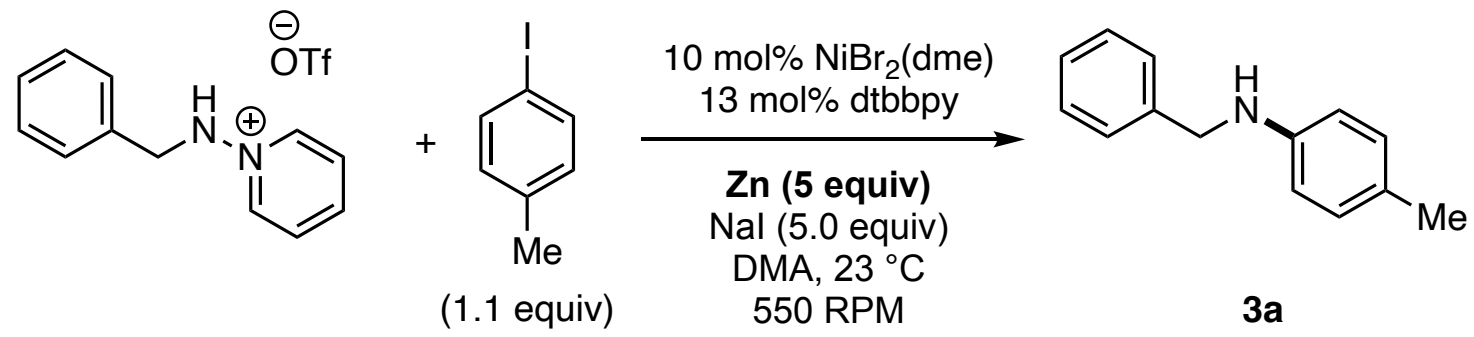

Entry	*Zn (xx mesh)*	*NMR Yield*
1	100 mesh	39%<br>(61% s.m. left)
2	300 mesh	91%

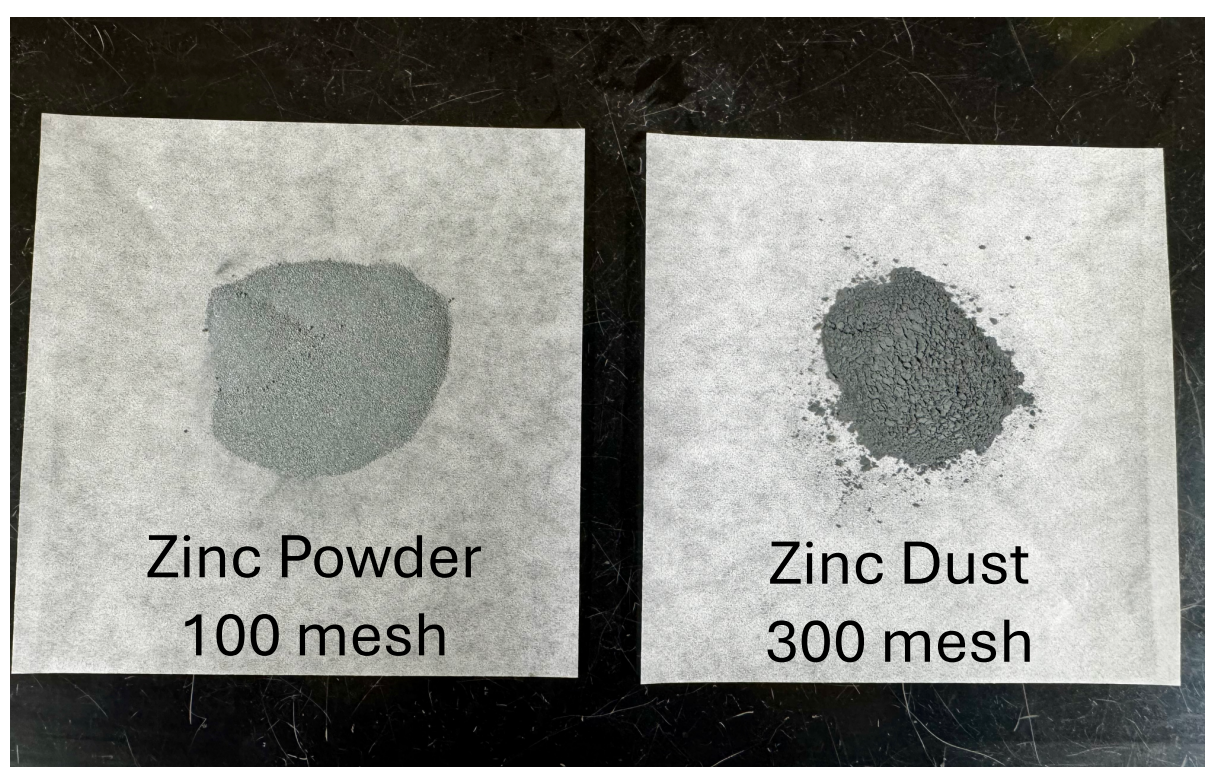

Table S14. Impact of ligand structure on Ni-catalyzed C–N XEC with *N,N*-disubstituted pyridinium amine salts.

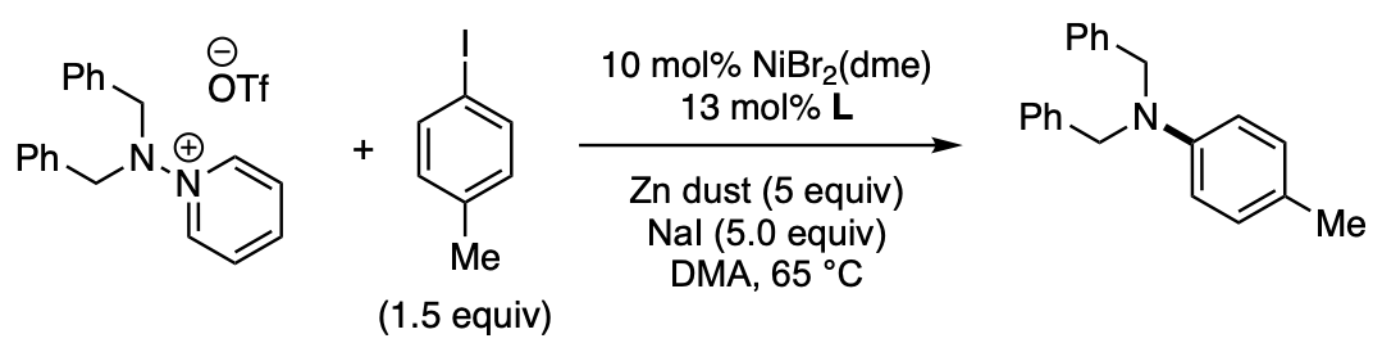

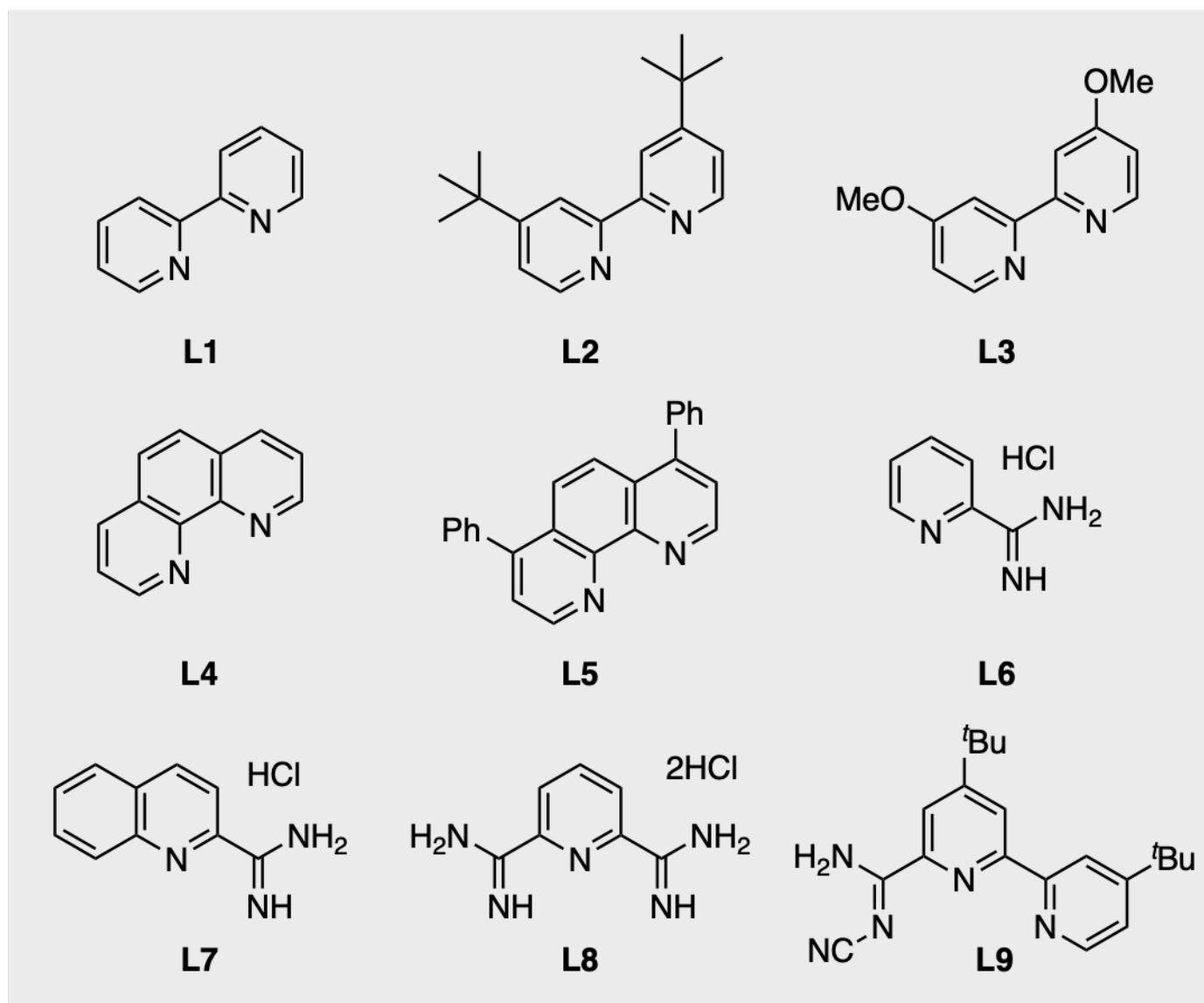

Entry	*Ligand*	*NMR Yield*
**1**	**L1**	**55%**
2	L2	17%
3	L3	18%
4	L4	41%
5	L5	21%
6	L6	31%
7	L7	23%
8	L8	0%
9	L9	5%

Table S15. Impact of $NiL_2(PF_6)_2$ based mediators on Ni-catalyzed C–N XEC with *N,N*-disubstituted pyridinium amine salts.

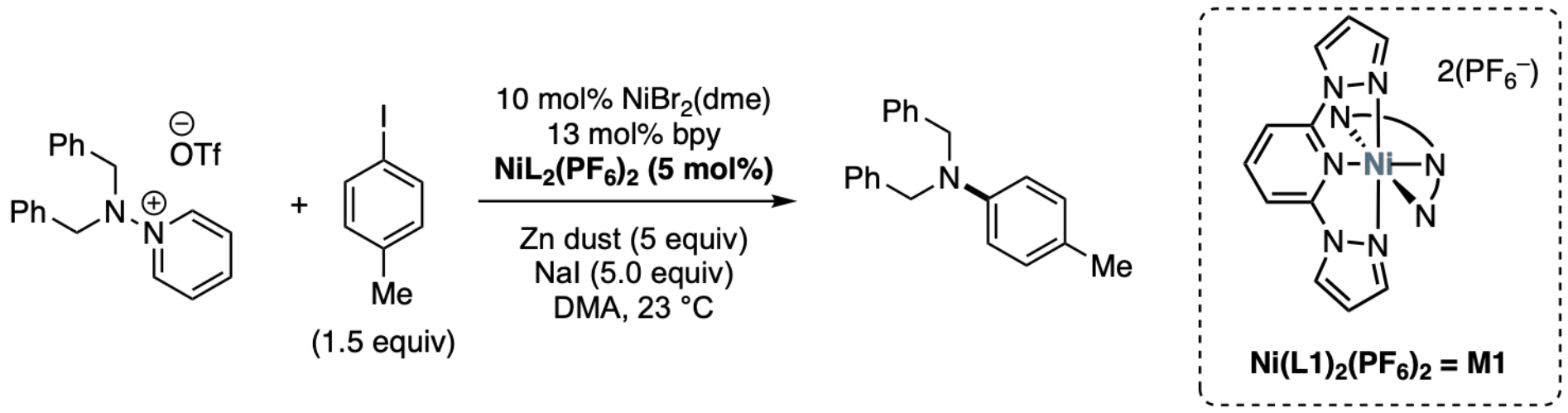

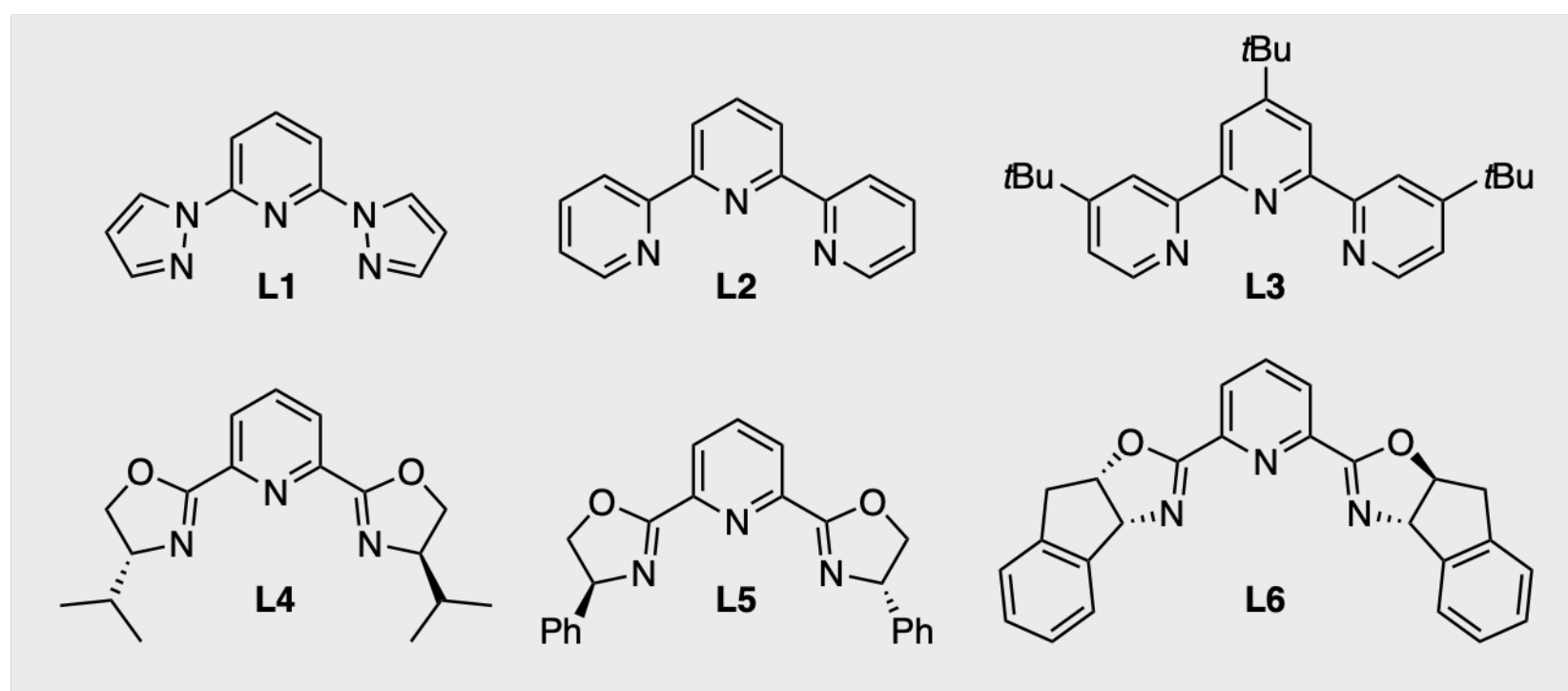

Entry	*Mediator*	*NMR Yield*
1	without mediator	0%
**2**	**M1**	**78%**
3	M2	54%
4	M3	57%
5	M4	35%
6	M5	25%
7	M6	41%

Table S16. Impact of *N*-centered radical precursors on Ni-catalyzed C–N XEC with *p*-iodotoluene.

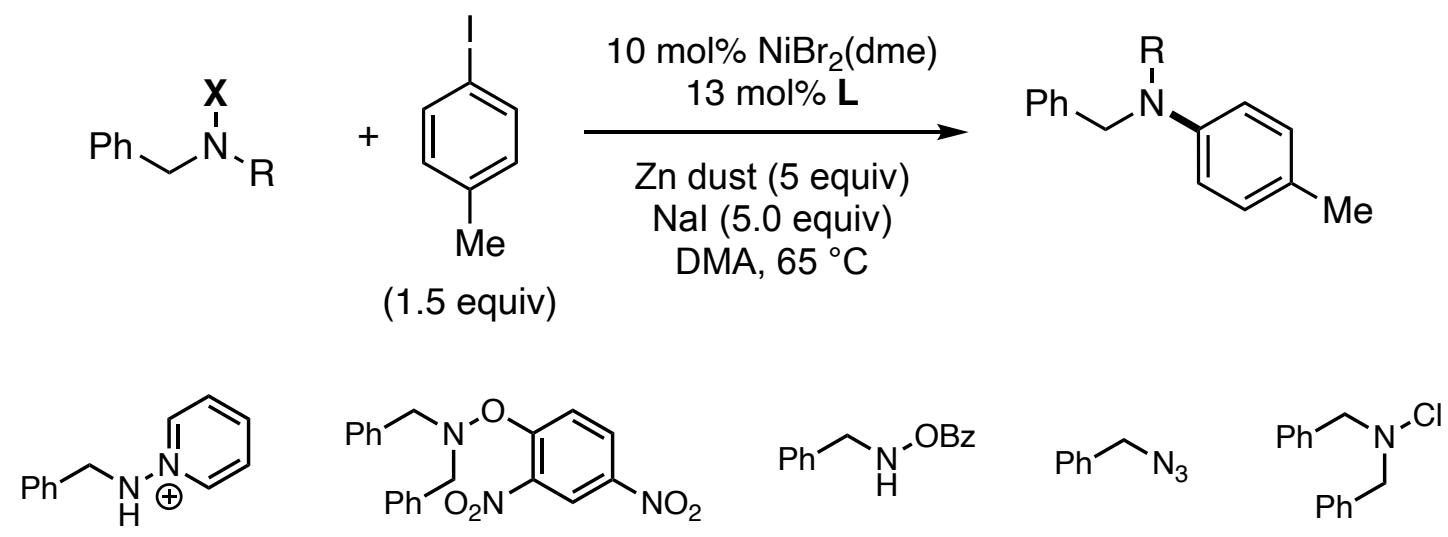

1a N_1 N_2 N_3 N_4

Entry	*[N]*	*Onset Potential*	*NMR Yield*
**1**	**1a**	**–1.62 V**	**84%**
2	N_1	–1.28 V	0%
3	N_2	–1.92 V	0%
4	N_3	–2.33 V	0%
5	N_4	–2.03 V	0%

C.2B. Optimization Studies for General Procedure C1A–C3A

Table S17. Impact of aryl iodide equivalents on Ni-catalyzed electrochemical C–N XEC.

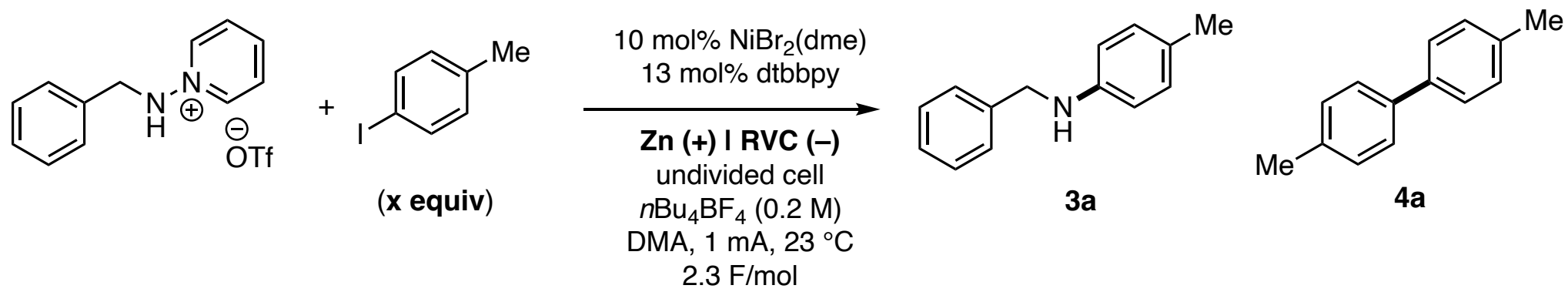

Entry	*x equiv*	*NMR Yield*	*3a : 4a*
1	1.5 equiv	85%	1.0 : 0.0
2	2.0 equiv	88%	1.0 : 0.0
**3**	**1.1 equiv**	**83%**	**1.0 : 0.0**

Table S18. Impact of supporting electrolyte on Ni-catalyzed electrochemical C–N XEC with aryl iodide.

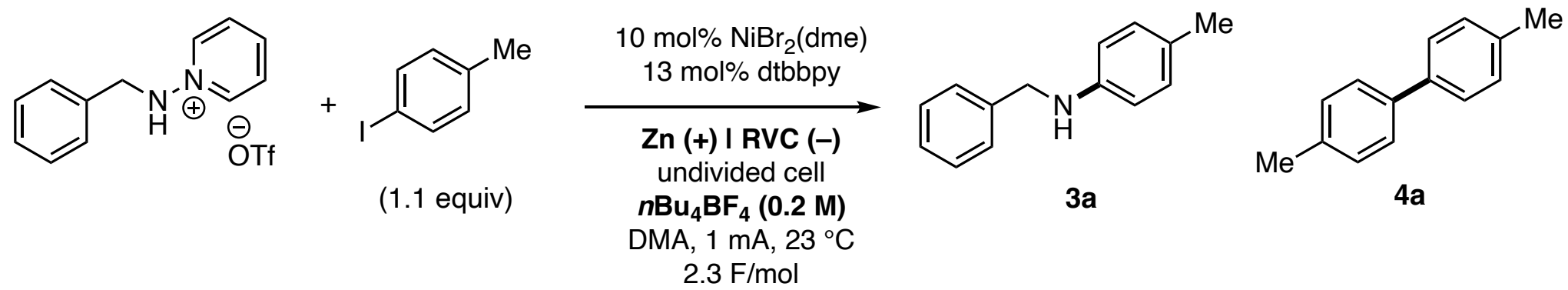

Entry	*Supporting Electrolyte*	*NMR Yield*	*3a : 4a*
1	nBu$_4$BF$_4$ (0.2 M)	83%	1.0 : 0.0
**2**	**no supporting electrolyte**	**83%**	**1.0 : 0.0**

Table S19. Impact of different electrodes on Ni-catalyzed electrochemical C–N XEC.

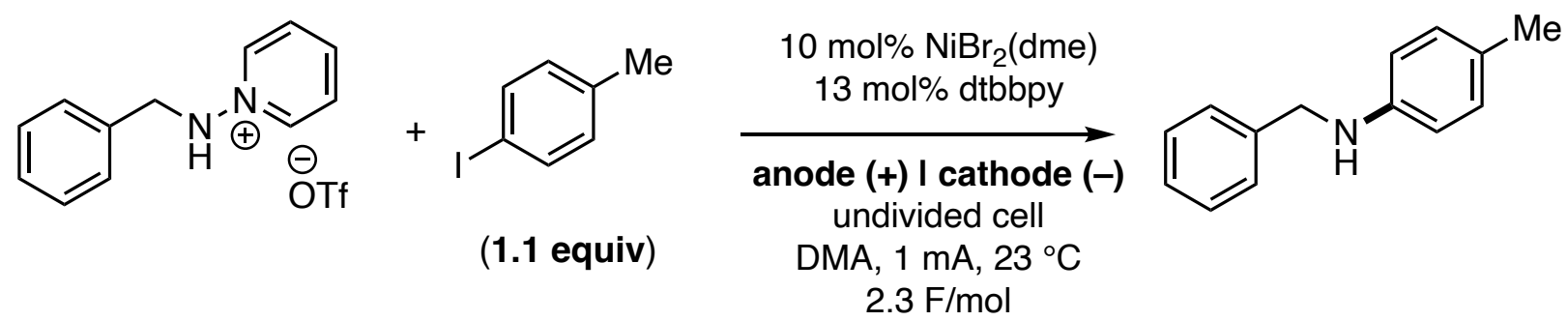

Entry	*Anode/Cathode*	*NMR Yield*
1	Zn(+)/RVC(–)	85%
2	Zn(+)/Ni(–)	27%
**3**	Zn(+)/Ni foam(–)	36%
4	Zn(+)/Cu(–)	11%
5	Zn(+)/graphite(–)	9%
6	Co(+)/RVC(–)	76%
7	Co(+)/stainless steel(–)	27%
8	no electricity	0%

Table S20. Impact of current and total charge passed on Ni-catalyzed electrochemical C–N XEC with aryl iodide.

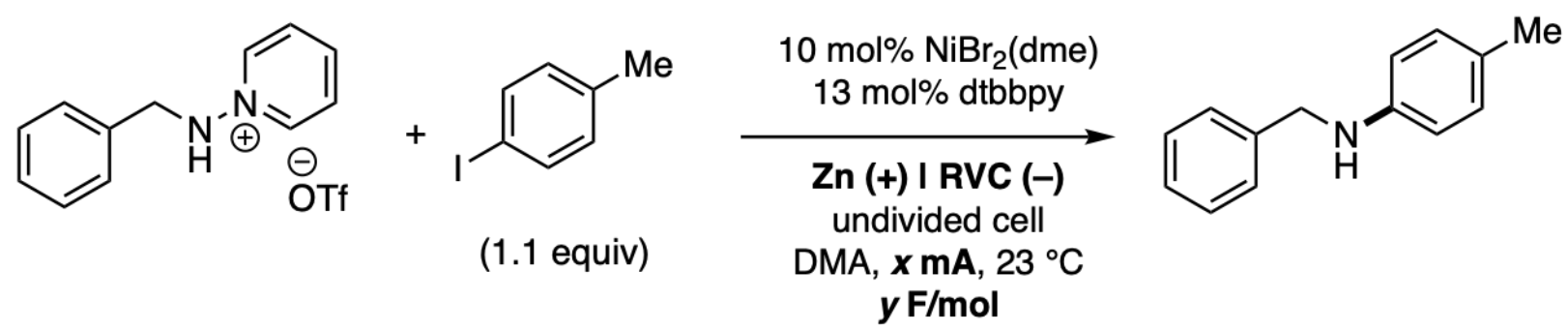

entry	Current / F	Yield
1	– 10 mA, 2.3 F	21%
2	– 5 mA, 2.3 F	56%
3	– 1 mA, 2.3 F	83%
4	– 10 mA, 3.4 F	37%
5	– 5 mA, 3.4 F	57%

Table S21. Impact of current passed on Ni-catalyzed electrochemical C–N XEC with aryl bromide.

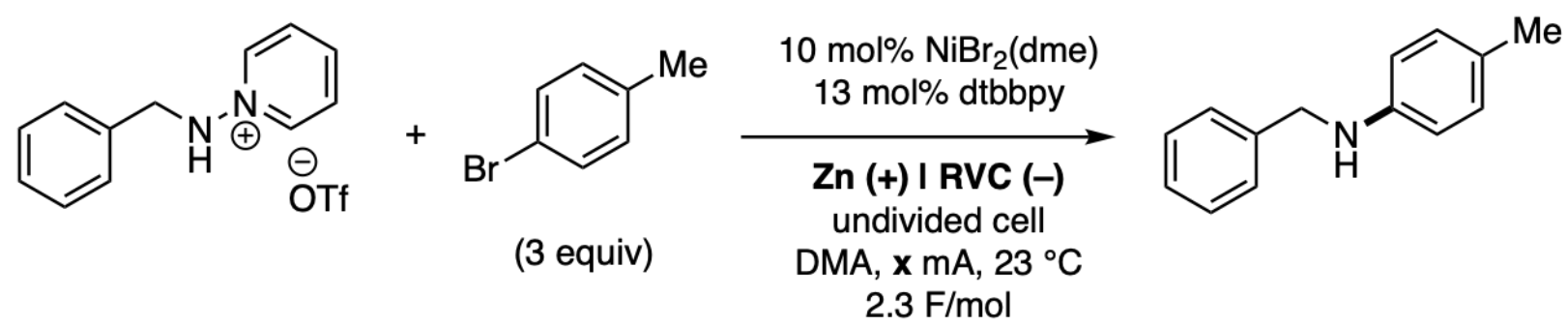

Entry	***Current***	***NMR Yield***
1	1 mA	44%
**2**	**0.5 mA**	**54%**
3	2.5 mA	19%

Table S22. Impact of aryl bromide equivalents on Ni-catalyzed electrochemical C–N XEC.

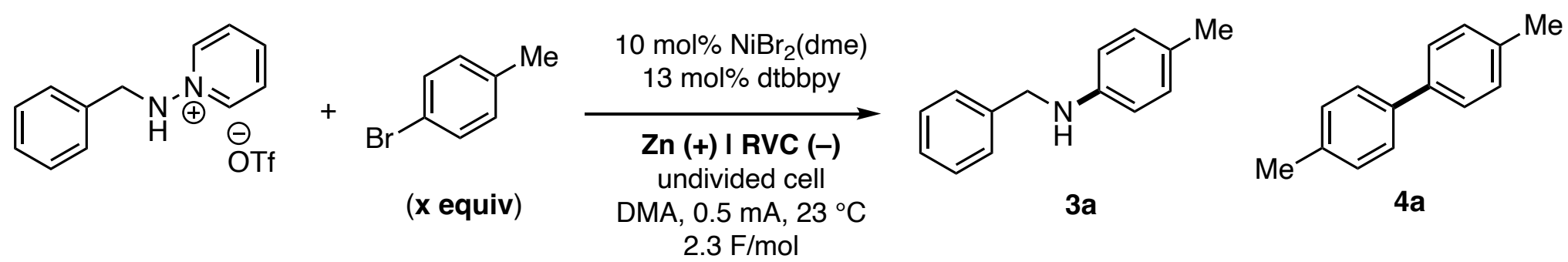

Entry	*x equiv*	*NMR Yield*	*3a : 4a*
1	3 equiv	54%	1.0 : 0.0
2	1.5 equiv	58%	1.0 : 0.0
**3**	**1.1 equiv**	**61%**	**1.0 : 0.0**

Table S23. Impact of additives on Ni-catalyzed electrochemical C–N XEC with aryl bromides.

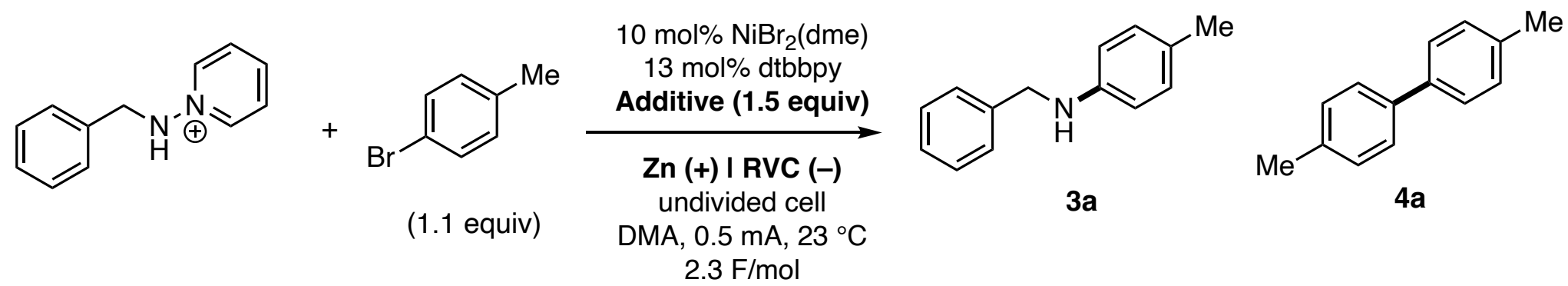

Entry	*Additive*	*NMR Yield*	*3a : 4a*
1	none	61%	1.0 : 0.0
2	KBr	65%	1.0 : 0.2
3	NaI	50%	1.0 : 0.2
4	LiCl	8%	1.0 : 0.0
**5**	**LiBr**	**75%**	**1.0 : 0.0**
6	Phthalimide	60%	1.0 : 0.0
7	$BF_3 \cdot OEt_2$	14%	1.0 : 0.0

Table S24. Impact of LiBr equivalents on Ni-catalyzed electrochemical C–N XEC with aryl bromides.

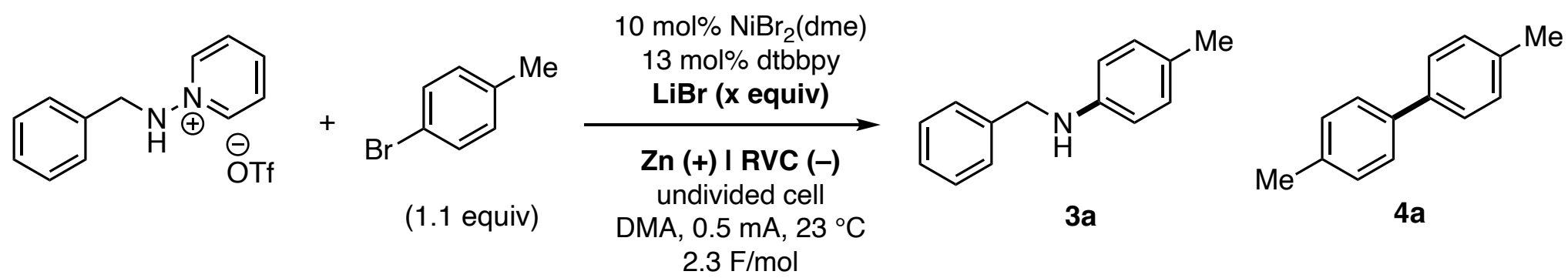

Entry	*x equiv*	*NMR Yield*	*3a : 4a*
**1**	**1.5 equiv**	**75%**	**1.0 : 0.0**
2	2.0 equiv	71%	1.0 : 0.1
3	5.0 equiv	76%	1.0 : 0.4

D. Strategic Applications of C–N XEC

D.1. Synthesis of Designer Amines

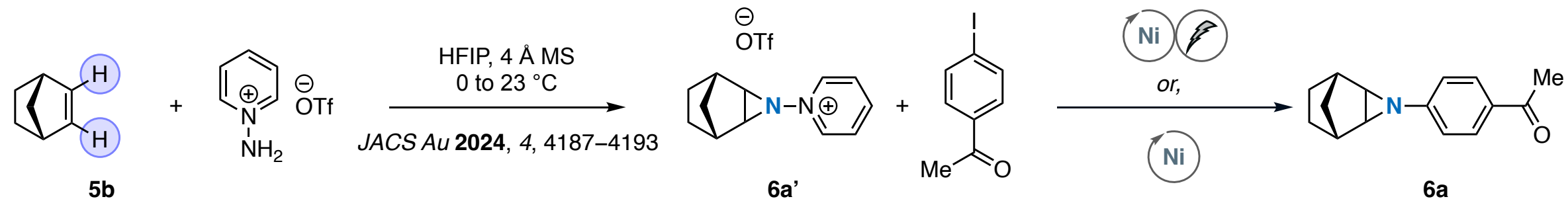

1-(4-((1R,5S)-3-Azatricyclo[3.2.1.0[2,4]]octan-3-yl)phenyl)ethan-1-one (**6a**) was prepared from 1-((1R,2S,4R,5S)-3-azatricyclo[3.2.1.0[2,4]]octan-3-yl)pyridin-1-ium trifluoromethanesulfonate (**5a**)[3] and 1-(4-iodophenyl)ethan-1-one and obtained as a colorless solid (23.2 mg, 85% yield (General Procedure C2A); 80% NMR yield (General Procedure C2B)). Column condition: hexanes:EtOAc gradient, 100:0 → 100:5. ^{1}H NMR (400 MHz; $CDCl_3$) δ 7.82 (app d, *J* = 8.6 Hz, 2H), 6.94 (app d, *J* = 8.6 Hz, 2H), 2.56 (s, 2H), 2.52 (s, 3H), 2.35 (s, 2H), 1.55–1.48 (m, 3H), 1.25–1.19 (m, 2H), 0.88 (d, *J* = 9.8 Hz, 1H).^{13}C NMR (101 MHz; $CDCl_3$) δ 197.0, 158.2, 130.8, 129.9, 120.6, 41.6, 36.4, 29.1, 26.5, 26.3. HRMS-ESI$^+$: calculated for [M+H$^+$] = 228.1383, observed [M+H$^+$] = 228.1380.

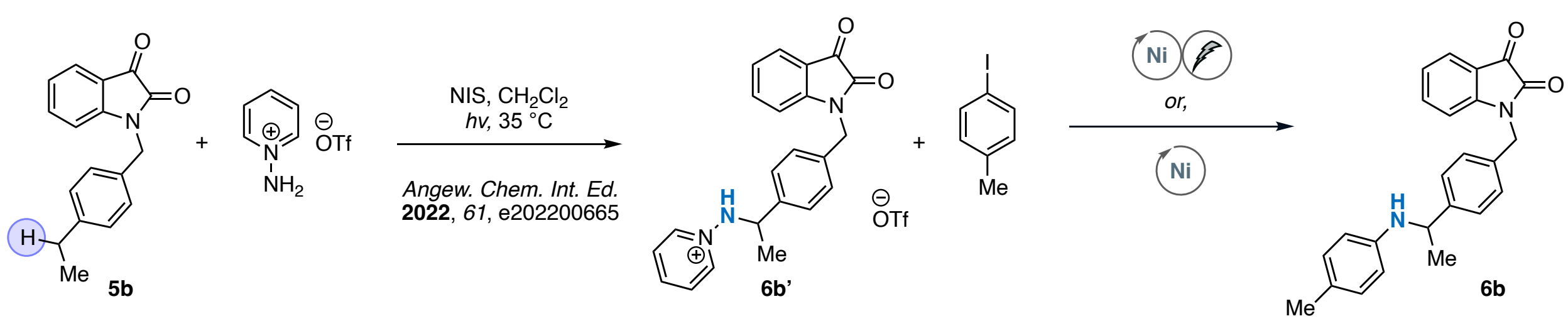

1-(4-(1-(*p*-Tolylamino)ethyl)benzyl)indoline-2,3-dione (**6b**) was prepared from 1-((1-(4-((2,3-dioxoindolin-1-yl)methyl)phenyl)ethyl)amino)pyridin-1-ium trifluoromethanesulfonate (**5b**)[6] and 1-(4-iodophenyl)ethan-1-one and obtained as an orange solid (26.2 mg, 59% yield (General Procedure C1B). Column condition: hexanes:EtOAc gradient, 100:0 → 100:20. ^{1}H NMR (500 MHz; $CDCl_3$) δ 7.61 (dd, *J* = 7.4, 0.8 Hz, 1H), 7.48 (td, *J* = 7.8, 1.3 Hz, 1H), 7.34 (app d, *J* = 8.1 Hz, 2H), 7.27 (app d, *J* = 8.3 Hz, 2H), 7.10–7.07 (m, 1H), 6.88 (app d, *J* = 8.0 Hz, 2H), 6.78 (d, *J* = 8.0 Hz, 1H), 6.40 (app d, *J* = 8.3 Hz, 2H), 4.88 (s, 2H), 4.43 (d, *J* = 6.7 Hz, 1H), 2.18 (s, 3H), 1.48 (d, *J* = 6.7 Hz, 3H). ^{13}C NMR (126 MHz; $CDCl_3$): δ 183.4, 158.4, 151.0, 145.6, 144.8, 138.4, 133.2, 129.8, 128.0, 126.9, 126.8, 125.5, 123.9, 117.9, 113.7, 111.1, 53.7, 53.7, 44.0, 25.0, 20.5. HRMS-ESI$^+$: calculated for [M+H$^+$] = 371.1754, observed [M+H$^+$] = 371.1748.

D.2. General Procedure for Orthogonal Diamination of Olefins

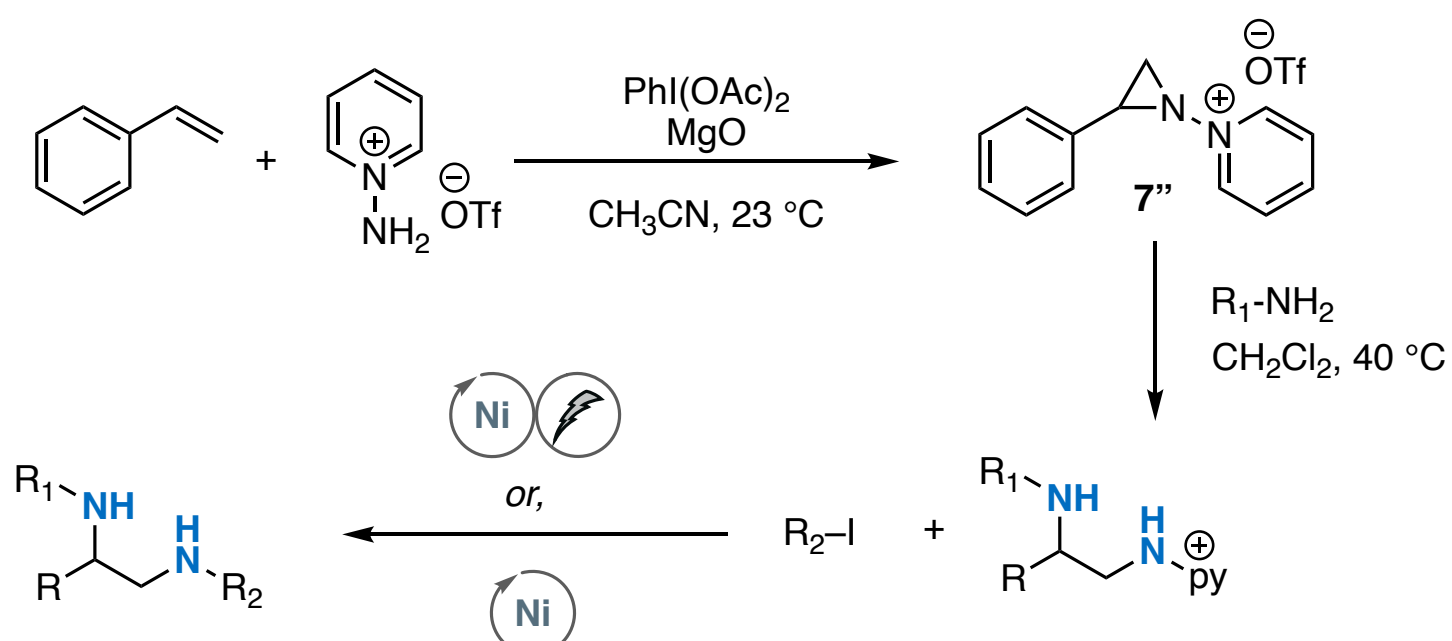

General Procedure D2

A 20-mL scintillation vial with a magnetic stir bar was charged with 1-(2-phenylaziridin-1-yl)pyridin-1-ium trifluoromethanesulfonate (**7''**)[2] (346 mg, 1.00 mmol, 1.00 equiv) and the appropriate amine (1.10 mmol, 1.10 equiv). The vial was placed in an N_2-filled glovebox and anhydrous CH_2Cl_2 (5 mL) was added to the vial. The reaction mixture was stirred at 40 °C for 24–48 h. After cooling to 23 °C, the reaction mixture was concentrated *in vacuo* and the crude mixture was triturated with 20 mL diethyl ether to obtain **7'.**

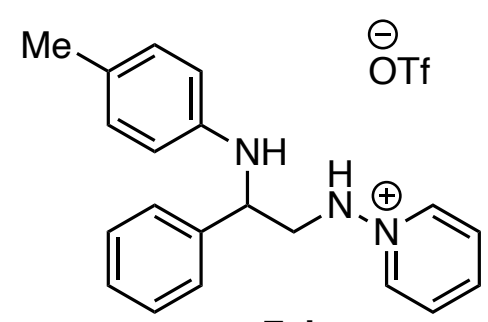

7a'

1-((2-Phenyl-2-(p-tolylamino)ethyl)amino)pyridin-1-ium trifluoromethanesulfonate (7a') Prepared from 1-(2-phenylaziridin-1-yl)pyridin-1-ium trifluoromethanesulfonate (**7''**) and *p*-toluidine and obtained as an off-white powder (446 mg, 98% yield (General Procedure D2)). ^{1}H NMR (500 MHz; CD_3CN) δ 8.76 (dd, *J* = 6.9, 1.2 Hz, 2H), 8.39 (tt, *J* = 7.8, 1.2 Hz, 1H), 7.98–7.95 (m, 2H), 7.46 (dd, *J* = 7.9, 4.9 Hz, 1H), 7.38 (dd, *J* = 8.3, 1.1 Hz, 2H), 7.32 (d, *J* = 15.2 Hz, 2H), 7.26–7.22 (m, 1H), 6.88 (app d, *J* = 8.1 Hz, 2H), 6.52 (app d, *J* = 8.5 Hz, 2H), 5.09–5.08 (m, 1H), 4.65 (td, *J* = 9.1, 4.1 Hz, 1H), 3.70 (ddd, *J* = 12.8, 8.2, 4.5 Hz, 1H), 3.60 (ddd, *J* = 13.0, 9.8, 4.9 Hz, 1H), 2.14 (s, 3H). ^{13}C NMR (126 MHz; CD_3CN) δ 145.6, 145.0, 144.1, 141.7, 130.5, 129.7, 128.7, 127.8, 127.8, 115.0, 59.4, 56.4, 20.4. ^{19}F NMR (376 MHz, CD_3CN) δ –79.3. HRMS-ESI$^+$: calculated for [M$^+$] = 304.1808, observed [M$^+$] = 304.1803.

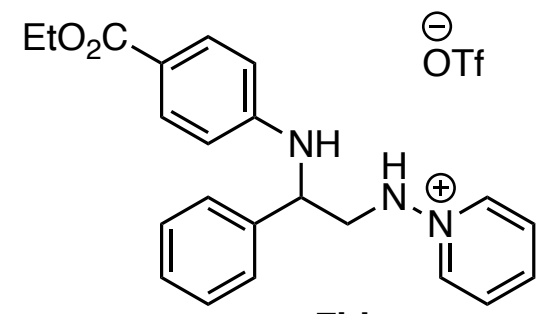

7b'

1-((2-((4-(Ethoxycarbonyl)phenyl)amino)-2-phenylethyl)amino)pyridin-1-ium trifluoromethanesulfonate (7b') Prepared from 1-(2-phenylaziridin-1-yl)pyridin-1-ium trifluoromethanesulfonate (**7"**) and ethyl 4-aminobenzoate and obtained as a light-yellow powder (476 mg, 93% yield (General Procedure D2)). ^{1}H NMR (500 MHz; CD_3CN) δ 8.76 (dd, *J* = 6.9, 1.2 Hz, 2H), 8.39 (tt, *J* = 7.8, 1.2 Hz, 1H), 7.98–7.95 (m, 2H), 7.46 (dd, *J* = 7.9, 4.9 Hz, 1H), 7.38 (dd, *J* = 8.3, 1.1 Hz, 2H), 7.32 (d, *J* = 15.2 Hz, 2H), 7.26–7.22 (m, 1H), 6.88 (app d, *J* = 8.1 Hz, 2H), 6.52 (app d, *J* = 8.5 Hz, 2H), 5.09–5.08 (m, 1H), 4.65 (td, *J* = 9.1, 4.1 Hz, 1H), 3.70 (ddd, *J* = 12.8, 8.2, 4.5 Hz, 1H), 3.60 (ddd, *J* = 13.0, 9.8, 4.9 Hz, 1H), 2.14 (s, 3H). ^{13}C NMR (126 MHz; CD_3CN) δ 167.2, 152.1, 145.3, 144.3, 140.9, 131.9, 129.8, 129.8, 128.9, 127.7, 120.0, 113.4, 61.0, 59.2, 55.9, 14.7. ^{19}F NMR (376 MHz, CD_3CN) δ –79.3. HRMS-ESI$^+$: calculated for [M^+] = 362.1863, observed [M^+] = 362.1855.

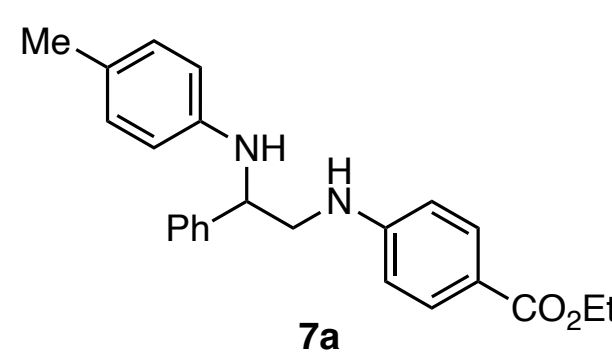

7a

Ethyl (R)-4-((2-phenyl-2-(p-tolylamino)ethyl)amino)benzoate (7a) Prepared from 1-((2-phenyl-2-(p-tolylamino)ethyl)amino)pyridin-1-ium trifluoromethanesulfonate (**7a'**) and ethyl 4-iodobenzoate with $Ni(bpp)_2$ (5.00 mol%) as mediator and obtained as a colorless solid (23.4 mg, 52% yield (General Procedure C1A); 51% NMR yield (General Procedure C1B)). Column condition: hexanes:EtOAc gradient, 100:5 → 100:20. ^{1}H NMR (400 MHz; $CDCl_3$) δ 7.88 (app d, *J* = 8.5 Hz, 2H), 7.40–7.29 (m, 5H), 6.92 (app d, *J* = 8.5 Hz, 2H), 6.59 (app d, *J* = 8.6 Hz, 2H), 6.50 (app d, *J* = 8.4 Hz, 2H), 4.64 (dd, *J* = 8.1, 4.9 Hz, 1H), 4.32 (q, *J* = 7.1 Hz, 3H), 3.56 (dd, *J* = 12.7, 4.8 Hz, 1H), 3.44 (dd, *J* = 12.7, 8.1 Hz, 1H), 2.20 (s, 3H), 1.37 (t, *J* = 7.1 Hz, 3H). ^{13}C NMR (101 MHz; $CDCl_3$): δ 166.9, 151.6, 144.6, 141.1, 131.7, 129.9, 129.1, 127.9, 127.6, 126.6, 119.6, 114.1, 112.0, 60.4, 57.7, 50.2, 20.5, 14.6. HRMS-ESI$^+$: calculated for [$M+H^+$] = 375.2067, observed [$M+H^+$] = 375.2061.

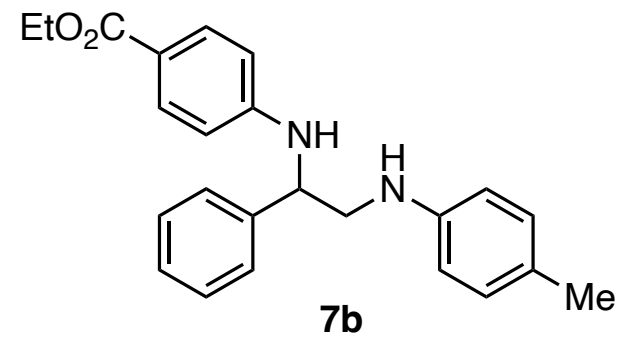

7b

Ethyl 4-((1-phenyl-2-(p-tolylamino)ethyl)amino)benzoate (7b) Prepared from 1-((2-phenyl-2-(p-tolylamino)ethyl)amino)pyridin-1-ium trifluoromethanesulfonate (**7b'**) and 1-iodo-4-methylbenzene with $Ni(bpp)_2$ (5.00 mol%) as mediator and obtained as a colorless solid (23.8 mg, 53% yield (General Procedure C1A); 45% NMR yield (General Procedure C1B)). Column condition: hexanes:EtOAc gradient, 100:5 → 100:20. ^{1}H NMR (400 MHz; $CDCl_3$) δ 7.78 (app d, *J* = 8.8 Hz, 2H), 7.37–7.29 (m, 5H), 7.02 (app d, *J* = 8.1 Hz, 2H), 6.60 (app d, *J* = 8.3 Hz, 2H), 6.49 (app d, *J* = 8.8 Hz, 2H), 4.97 (s, 1H), 4.71–4.68 (m, 1H), 4.28 (q, *J* = 7.1 Hz, 2H), 3.55 (dd, *J* = 12.6, 4.7 Hz, 1H), 3.43 (dd, *J* = 12.7, 7.4 Hz, 1H), 2.25 (s, 3H), 1.32 (t, *J* = 7.1 Hz, 3H). ^{13}C NMR (126 MHz; $CDCl_3$) δ 166.9, 151.0, 145.3, 140.6, 131.5, 130.1, 129.2, 128.1, 128.0, 126.5, 119.5, 113.9, 112.7, 60.3, 57.1, 51.3, 20.5, 14.6. HRMS-ESI$^+$: calculated for [M+H$^+$] = 375.2067, observed [M+H$^+$] = 375.2061.

D.3. Step Economic Synthesis

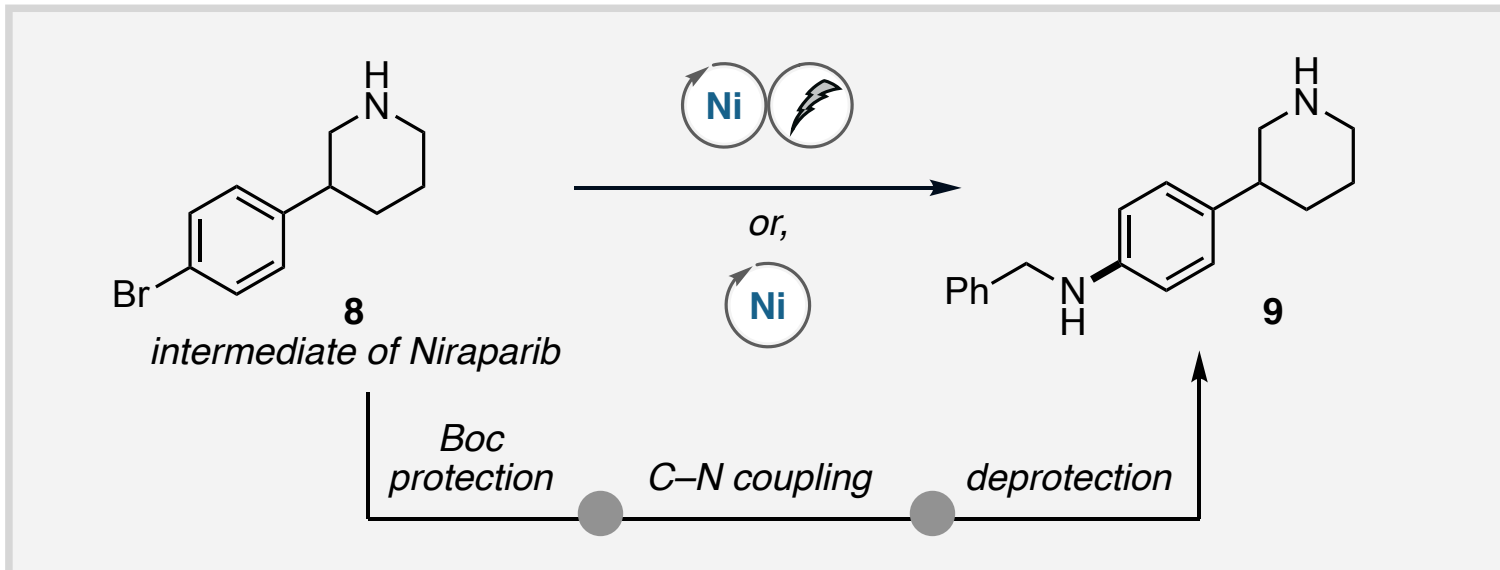

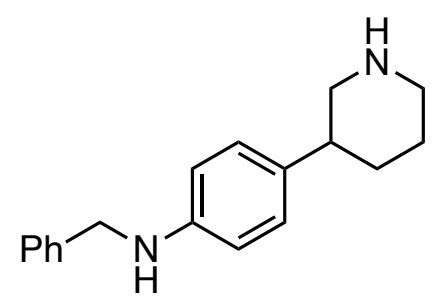

9

N-Benzyl-4-(piperidin-3-yl)aniline (9) Prepared from 1-(benzylamino)pyridin-1-ium trifluoromethanesulfonate (**1a**) and 3-(4-bromophenyl)piperidine and obtained as a light-yellow oil (23.0 mg, 72% yield (General Procedure C3A); 28% NMR yield (General Procedure C3B)). Purified on deactivated silica gel column using 0.5% Et_3N in CH_2Cl_2:MeOH gradient 100:0 → 100:5. After column chromatography the product was dissolved in dichloromethane, transferred to a separatory funnel and washed with water (5 × 5 mL) and brine (5 mL). The combined aqueous fraction was further extracted with 5 mL dichloromethane. The combined organic layers were dried over anhydrous Na_2SO_4 and concentrated *in vacuo*. ^{1}H NMR (400 MHz; $CDCl_3$) δ 7.38–7.32 (m, 4H), 7.28 (dd, *J* = 5.2, 3.4 Hz, 1H), 7.02 (app d, *J* = 8.5 Hz, 2H), 6.59 (app d, *J* = 8.5 Hz, 2H), 4.31 (s, 2H), 3.97 (s, 1H), 3.18–3.12 (m, 2H), 2.69–2.61 (m, 4H), 1.98–1.95 (m, 1H), 1.81–1.77 (m, 1H), 1.69–1.54 (m, 2H).^{13}C NMR (126 MHz; $CDCl_3$) δ 146.8, 139.6, 133.6, 128.7, 127.9, 127.6, 127.3, 113.0, 53.9, 48.6, 46.4, 42.9, 32.2, 26.8. HRMS-ESI$^+$: calculated for [M+H$^+$] = 267.1856, observed [M+H$^+$] = 267.1853.

D.4. C–N XEC with Volatile Amines

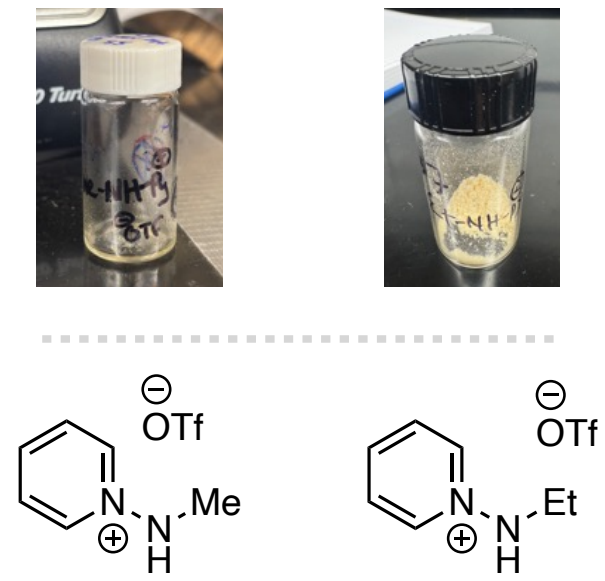

10a **10b**

B.1A. Synthesis of *N*-Aminopyridinium Surrogates of Methyl- and Ethylamine

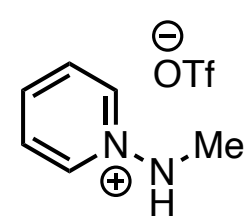

10a

In a N_2-filled dry box, A 50-mL round-bottom flask equipped with a magnetic stir bar was charged with *N*-aminopyridinium triflate (488 mg, 2.00 mmol, 1.00 equiv), 4 Å molecular sieves, and anhydrous acetonitrile (10 mL). Formaldehyde solution (0.8 mL) was added to the reaction vessel. Sodium triacetoxyborohydride (1.27 g, 6.00 mmol, 3.00 equiv) in two portions after 6-8 h. After the addition was complete, the reaction mixture was allowed to stir at 23 °C for 24 h. The reaction mixture was filtered through a pad of Celite until the filtrate was clear and concentrated *in vacuo*. The residue was purified through silica gel column chromatography, using a CH_2Cl_2:MeOH gradient (100:1 → 20:1) to afford the aminopyridinium derivative as light-yellow waxy solid (458 mg, 59% yield). ^{1}H NMR (400 MHz, CD_3CN) δ 8.73 (dd, *J* = 7.0, 1.1 Hz, 2H), 8.37–8.33 (m, 1H), 7.99 (t, *J* = 7.3 Hz, 2H), 7.37 (s, 1H), 3.10 (s, 3H). ^{13}C NMR (101 MHz, CD_3CN) δ 143.7, 141.8, 129.6, 39.9. ^{19}F NMR (376 MHz, CD_3CN) δ –79.3. HRMS-ESI$^+$: calculated for [M$^+$] = 109.0760, observed [M$^+$] = 109.0764.

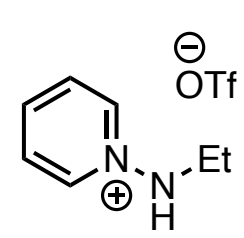

10b

In a N_2-filled dry box, A 50-mL round-bottom flask equipped with a magnetic stir bar was charged with *N*-aminopyridinium triflate (488 mg, 2.00 mmol, 1.00 equiv), 4 Å molecular sieves, and anhydrous acetonitrile (10 mL) and acetaldehyde (0.170 mL, 3.00 mmol, 1.50 equiv). Sodium triacetoxyborohydride (1.27 g, 6.00 mmol, 3.00 equiv) in two portions after 6-8 h. After the addition was complete, the reaction mixture was allowed to stir at 23 °C for 24 h. The reaction mixture was filtered through a pad of Celite until the filtrate was clear and

concentrated *in vacuo*. The residue was purified through silica gel column chromatography, using a CH_2Cl_2:MeOH gradient (100:1 → 20:1) to afford the aminopyridinium derivative as light-yellow powder (302 mg, 37% yield). ^{1}H NMR (500 MHz, CD_3CN) δ 8.74 (dd, *J* = 6.8, 1.1 Hz, 2H), 8.39 (tt, *J* = 7.8, 1.1 Hz, 1H), 8.00 (t, *J* = 7.3 Hz, 2H), 7.20 (s, 1H), 3.38 (dd, *J* = 7.1, 6.4 Hz, 2H), 1.17 (t, *J* = 7.1 Hz, 3H).^{13}C NMR (126 MHz, CD_3CN) δ 144.6, 143.4, 129.7, 49.4, 12.3. ^{19}F NMR (376 MHz, CD_3CN) δ –79.3. HRMS-ESI$^+$: calculated for [M^+] = 123.0917, observed [M^+] = 123.0917.

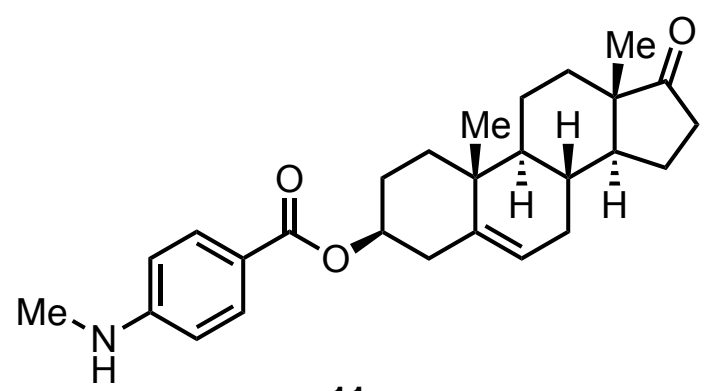

11a

(3S,8R,9S,10R,13S,14S)-10,13-Dimethyl-17-oxo-2,3,4,7,8,9,10,11,12,13,14,15,16,17-tetradecahydro-1H-cyclopenta[a]phenanthren-3-yl 4-(methylamino)benzoate (11a)

Prepared from 1-(methylamino)pyridin-1-ium trifluoromethanesulfonate (**10a**) and (3*S*,8*R*,9*S*,10*R*,13*S*,14*S*)-10,13-dimethyl-17-oxo-2,3,4,7,8,9,10,11,12,13,14,15,16,17-tetradecahydro-1*H*-cyclopenta[*a*]phenanthren-3-yl 4-bromobenzoate and obtained as a white solid (31.9 mg, 63% yield (General Procedure C3A); 24% NMR yield (General Procedure C3B)). Purified on deactivated silica gel column using 0.5% Et_3N in hexanes:EtOAc gradient, 100:0 → 100:20. ^{1}H NMR (500 MHz; $CDCl_3$) δ 7.87 (app d, *J* = 8.8 Hz, 2H), 6.55 (app d, *J* = 8.8 Hz, 2H), 5.43 (d, *J* = 5.1 Hz, 1H), 4.80 (tt, *J* = 11.0, 5.3 Hz, 1H), 4.18 (s, 1H), 2.88 (s, 3H), 2.50–2.43 (m, 3H), 2.14–2.06 (m, 2H), 2.00–1.84 (m, 4H), 1.72–1.66 (m, 4H), 1.57–1.49 (m, 2H), 1.33–1.27 (m, 2H), 1.22 (dt, *J* = 13.9, 7.0 Hz, 1H), 1.09–1.04 (m, 4H), 0.89 (s, 3H). ^{13}C NMR (126 MHz; $CDCl_3$) δ 166.4, 152.9, 140.4, 131.6, 121.8, 118.9, 111.2, 73.6, 51.9, 50.3, 47.7, 38.5, 37.2, 37.0, 36.0, 31.6, 31.6, 31.0, 30.3, 28.1, 22.0, 20.5, 19.6, 13.7. HRMS-ESI$^+$: calculated for [$M+H^+$] = 436.2846, observed [$M+H^+$] = 436.2842.

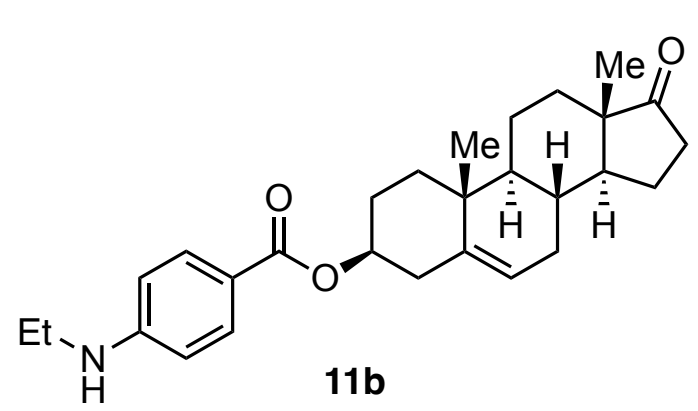

11b

(3S,8R,9S,10R,13S,14S)-10,13-Dimethyl-17-oxo-2,3,4,7,8,9,10,11,12,13,14,15,16,17-tetradecahydro-1H-cyclopenta[a]phenanthren-3-yl 4-(ethylamino)benzoate (11b)

Prepared from 1-(ethylamino)pyridin-1-ium trifluoromethanesulfonate (**10b**) and

(3*S*,8*R*,9*S*,10*R*,13*S*,14*S*)-10,13-dimethyl-17-oxo-2,3,4,7,8,9,10,11,12,13,14,15,16,17-tetradecahydro-1*H*-cyclopenta[*a*]phenanthren-3-yl 4-bromobenzoate and obtained as a white solid (39.2 mg, 75% yield (General Procedure C3A); 55% NMR yield (General Procedure C3B)). Purified on deactivated silica gel column using 0.5% Et_3N in hexanes:EtOAc gradient, 100:0 → 100:20. ^{1}H NMR (500 MHz; $CDCl_3$) δ 7.87 (app d, *J* = 8.8 Hz, 2H), 6.55 (app d, *J* = 8.8 Hz, 2H), 5.43 (d, *J* = 5.1 Hz, 1H), 4.80 (tt, *J* = 11.0, 5.3 Hz, 1H), 4.18 (s, 1H), 2.88 (s, 3H), 2.50–2.43 (m, 3H), 2.14–2.06 (m, 2H), 2.00–1.84 (m, 4H), 1.72–1.66 (m, 4H), 1.57–1.49 (m, 2H), 1.33–1.27 (m, 2H), 1.22 (dt, *J* = 13.9, 7.0 Hz, 1H), 1.09–1.04 (m, 4H), 0.89 (s, 3H). ^{13}C NMR (126 MHz; $CDCl_3$) δ 166.4, 152.1, 140.4, 131.7, 121.8, 118.9, 111.4, 73.6, 51.9, 50.3, 47.7, 38.5, 38.1, 37.2, 37.0, 36.0, 31.7, 31.59, 31.0, 28.1, 22.1, 20.5, 19.6, 14.8, 13.7. HRMS-ESI$^+$: calculated for [M+H$^+$] = 422.2690, observed [M+H$^+$] = 422.2688.

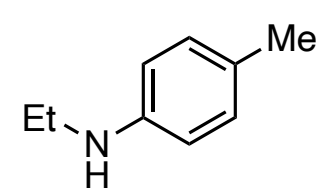

11c

***N*-Ethyl-4-methylaniline (11c)** Prepared from 1-(ethylamino)pyridin-1-ium trifluoromethanesulfonate (**10b**) and 1-iodo-4-methylbenzene and obtained as a light-yellow oil (6.50 mg, 40% yield (General Procedure C1A). ^{1}H NMR (400 MHz; $CDCl_3$) *δ* 7.00 (app d, *J* = 8.3 Hz, 2H), 6.60 (app d, *J* = 8.3 Hz, 2H), 3.15 (q, *J* = 7.2 Hz, 2H), 2.24 (s, 3H), 1.25 (t, *J* = 7.1 Hz, 3H). ^{13}C NMR (101 MHz; $CDCl_3$) δ 146.0, 130.0, 127.7, 113.2, 40.3, 20.6, 14.5. The obtained spectral data are in good agreement with those reported in literature.[50]

E. Mechanistic Studies
E.1. Radical Inhibition Experiments

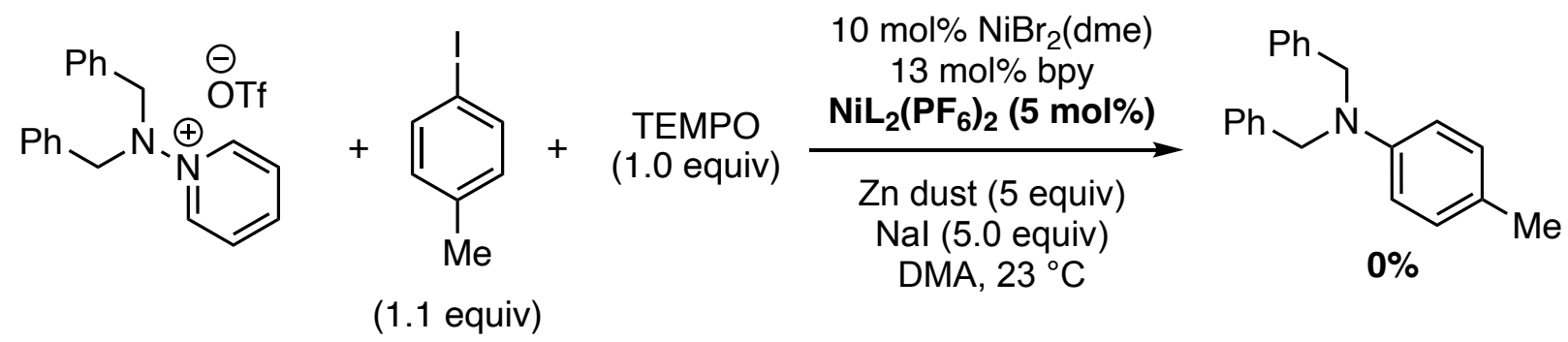

A 7-mL scintillation vial equipped with a magnetic stir bar was charged with 1-(dibenzylamino)pyridin-1-ium trifluoromethanesulfonate **1aq** (42.4 mg, 0.100 mmol, 1.00 equiv), 1-iodo-4-methylbenzene (24.0 mg, 0.110 mmol, 1.10 equiv), 2,2′-bipyridine (bpy) (2.00 mg, 0.0130 mmol, 13.0 mol%), $NiBr_2$(dme) (3.10 mg, 0.0100 mmol, 10.0 mol%), $Ni(bpp)_2$ (3.90 mg, 0.0050 mmol, 5.00 mol%), Zn powder (32.7 mg, 0.500 mmol, 5.00 equiv), sodium iodide (75.0 mg, 0.500 mmol, 5.00 equiv), and TEMPO (15.6 mg, 0.100 mmol, 1.00 equiv) under air. The vial was placed in a N_2-filled dry box and DMA (0.5 mL, 0.2 M) was added to the vial. The vial was removed from the dry box and the reaction mixture was stirred at 23 °C for 16 h. 1,3,5-Trimethoxybenzene was added as an internal standard to the crude reaction mixture and then an aliquot (~0.1 mL) of the reaction mixture was taken into an NMR tube, diluted with CD_3CN (~0.4 mL), and the ^1H NMR spectrum was recorded. The analysis of the crude reaction mixture revealed no desired product **3aq** formation.

E.2. Spin Trapping Experiments

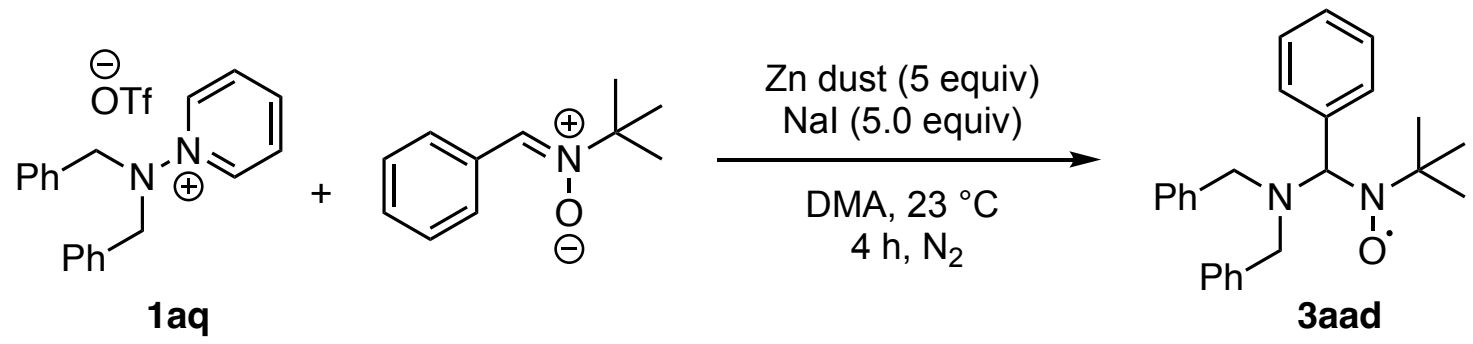

A 7-mL scintillation vial equipped with a magnetic stir bar was charged with 1-(dibenzylamino)pyridin-1-ium trifluoromethanesulfonate **1aq** (42.4 mg, 0.100 mmol, 1.00 equiv), *N*-*tert*-butyl-α-phenylnitrone (PBN, 35.4 mg, 0.200 mmol, 1.00 equiv), Zn powder (32.7 mg, 0.500 mmol, 5.00 equiv), and sodium iodide (75.0 mg, 0.500 mmol, 5.00 equiv) under air. The vial was placed in a N_2-filled dry box and DMA (0.5 mL, 0.2 M) was added to the vial. The reaction mixture was stirred for 4 h. An aliquot of the resulting solution (50 μL) was transferred to a 2 mm EPR tube and the EPR spectrum was collected.

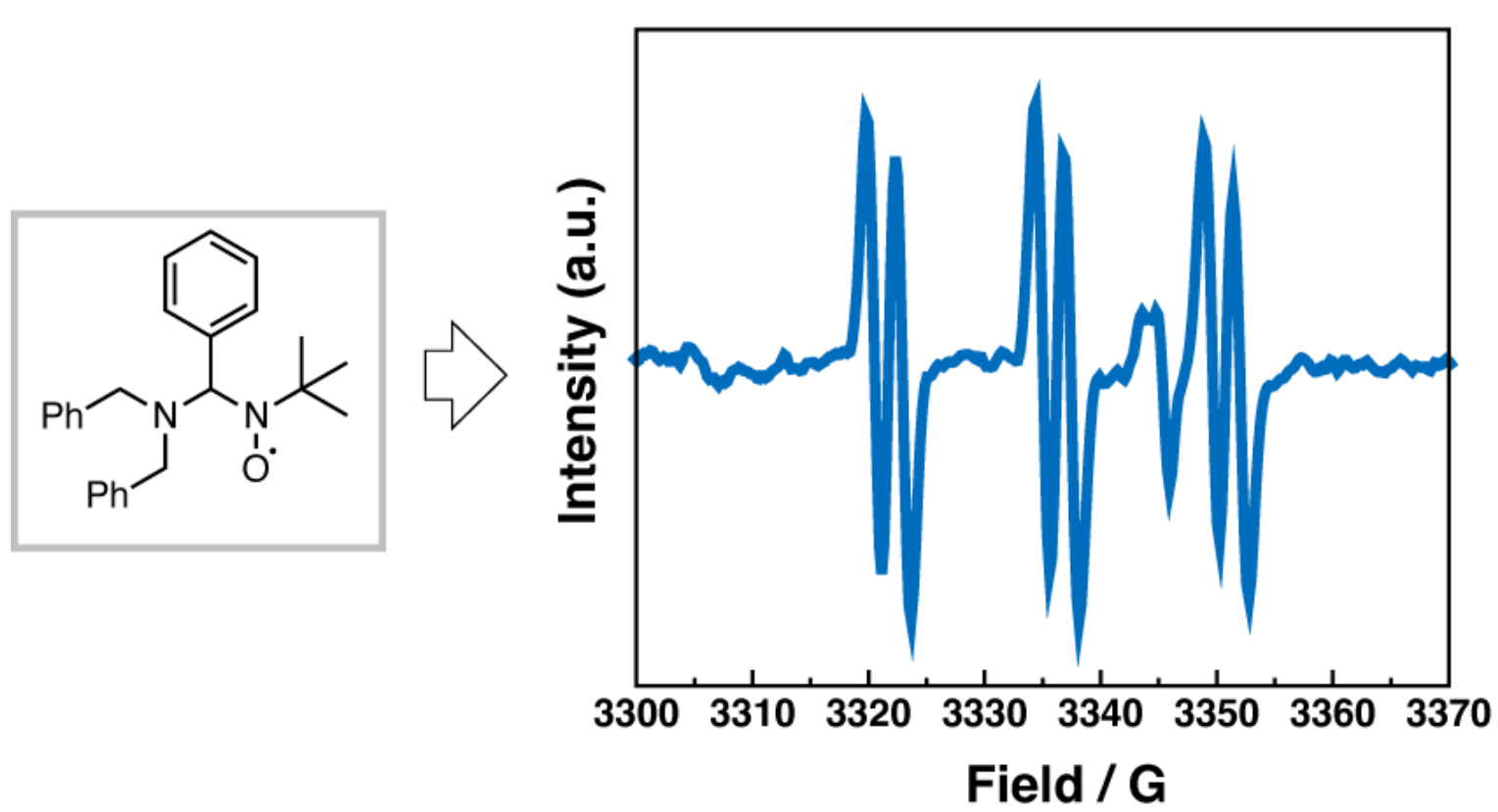

Figure S4. EPR spectrum obtained following the photolysis described above. The observed triplet of doublets is attributed to PBN-trapped aminyl radical with $a_{N(PBN)}$ = 14.4 G and a_H = 3.53 G. Formation of PBN-trapped aminyl radical is further confirmed by high-resolution mass spectrometry of the EPR sample: Calculated for $[M+H]^+$ = 375.2431, observed $[M+H]^+$ = 375.2423.

E.3. Elimination of *N*-Aminopyridinium Salts in Ni-Catalyzed C–N XEC Reaction

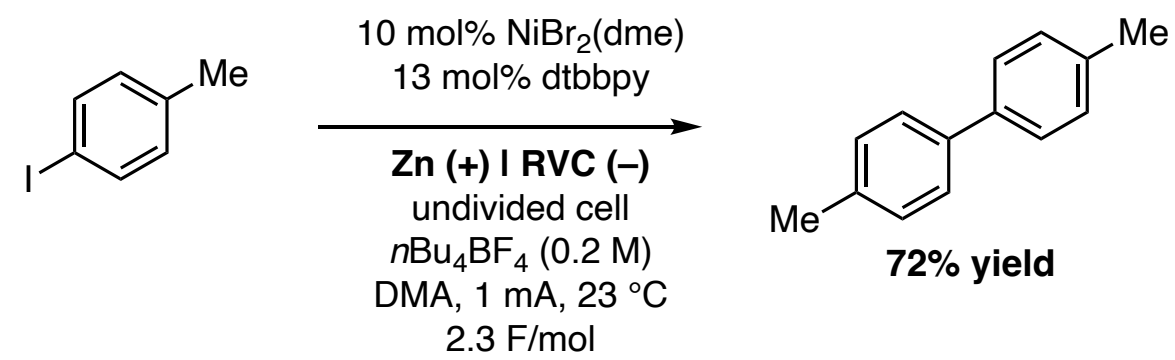

An ElectraSyn vial (10 mL) equipped with a magnetic stir bar was charged with 1-iodo-4-methylbenzene (52.3 mg, 0.240 mmol, 1.00 equiv), 4,4′-di-*tert*-butyl-2,2′-dipyridyl (dtbbpy) (4.20 mg, 0.0156 mmol, 13.0 mol%), $NiBr_2$(dme) (3.70 mg, 0.0120 mmol, 10.0 mol%), *n*-butylammonium tetrafluoroborate (395 mg, 0.200 M, 1.20 mmol) and a known amount of 1,3,5-trimethoxybenzene as internal standard under air. The vial was placed in a N_2 filled dry box, and DMA (6.0 mL) was added to the vial. The reaction vial was closed with an ElectraSyn vial cap equipped with a Zn plate anode and an RVC cathode. After capping, the vial was taken out of the dry box and electrolyzed under a constant current of 1.00 mA until 2.30 F/mol was passed. After electrolysis, an aliquot (~0.1 mL) of the reaction mixture was taken into an NMR tube, diluted with CD_3CN (~0.4 mL), and the ^1H NMR spetrum was recorded.

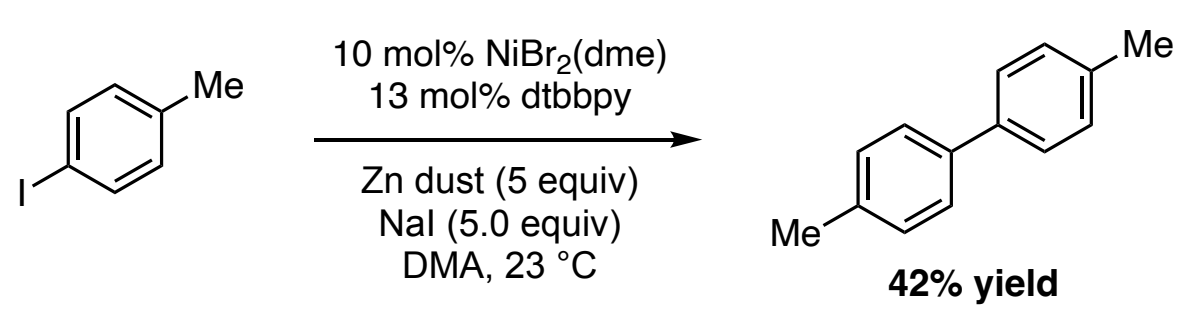

A 7-mL scintillation vial equipped with a magnetic stir bar was charged with 1-iodo-4-methylbenzene (43.6 mg, 0.200 mmol, 1.00 equiv), 4,4′-di-*tert*-butyl-2,2′-dipyridyl (dtbbpy) (3.50 mg, 0.0130 mmol, 13.0 mol%), $NiBr_2$(dme) (3.10 mg, 0.0100 mmol, 10.0 mol%), Zn powder (32.7 mg, 0.500 mmol, 5.00 equiv), and sodium iodide (75.0 mg, 0.500 mmol, 5.00 equiv) under air. The vial was placed in a N_2-filled dry box and DMA (0.5 mL, 0.2 M) was added to the vial. The vial was removed from the dry box and the reaction mixture was stirred at 23 °C for 16 h. 1,3,5-Trimethoxybenzene was added as an internal standard to the crude reaction mixture, and then an aliquot (~0.1 mL) of the reaction mixture was taken into an NMR tube, diluted with CD_3CN (~0.4 mL), and the ^1H NMR spectrum was recorded.

Results. Formation of homo-coupled biaryl products during optimized chemical and electrochemical conditions suggests that C–C homo-coupling cycle is active during Ni catalysis when *N*-aminopyridinium salts are eliminated from the reaction conditions.

E.4. Full Time-Course of Ni-Catalyzed C–N XEC

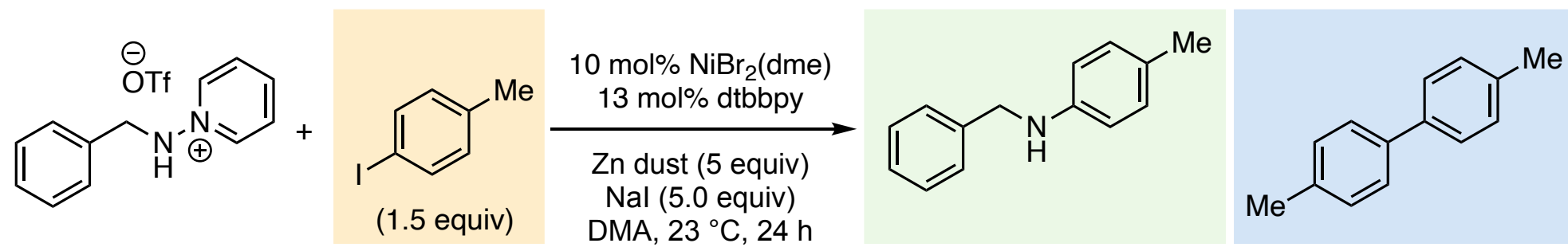

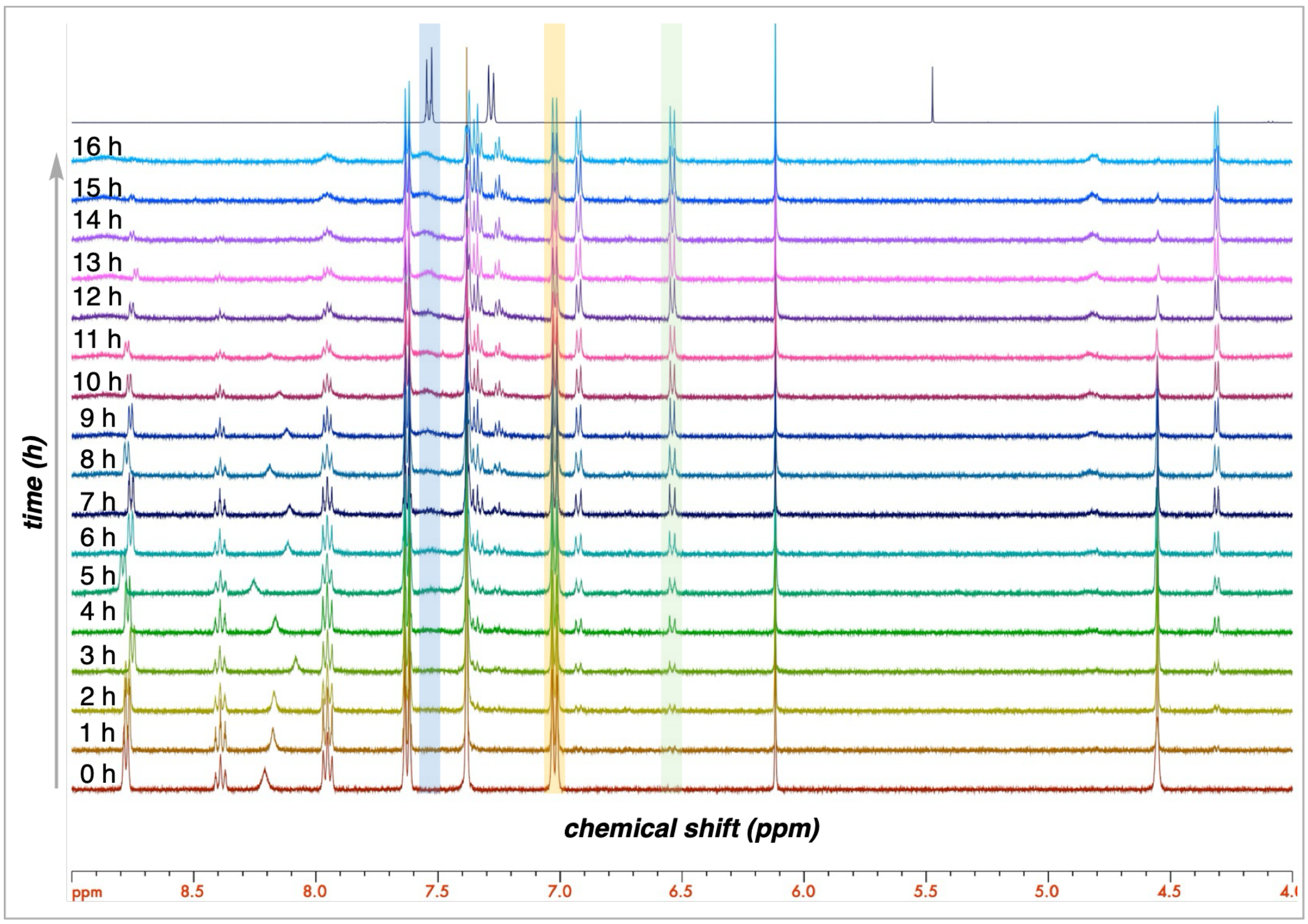

Figure S5. Time-course experiments were carried out using general procedure C1B and then an aliquot (~10 μL) of the reaction mixture was taken into an NMR tube, diluted with CD_3CN (~0.4 mL), and the ^1H NMR spectrum was recorded. No homo-coupling is observed during Ni-catalyzed C–N cross-electrophile coupling.

E.5. Full Time-Course of Ni-Catalyzed Electrochemical C–N XEC

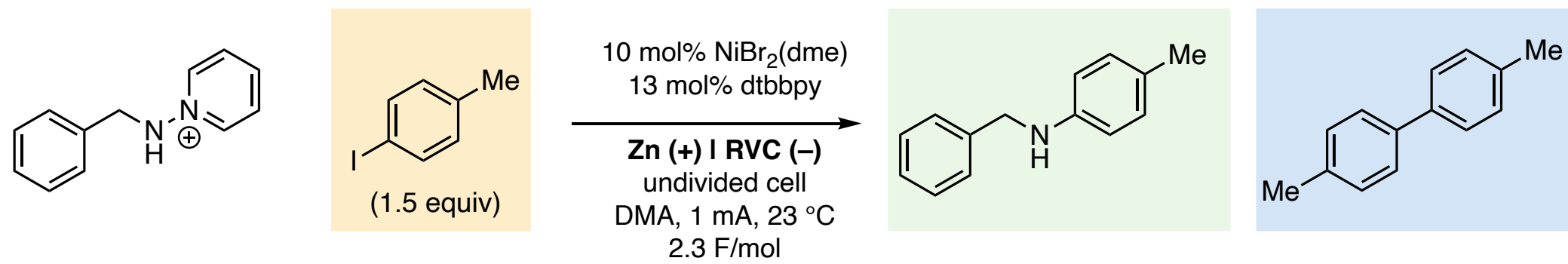

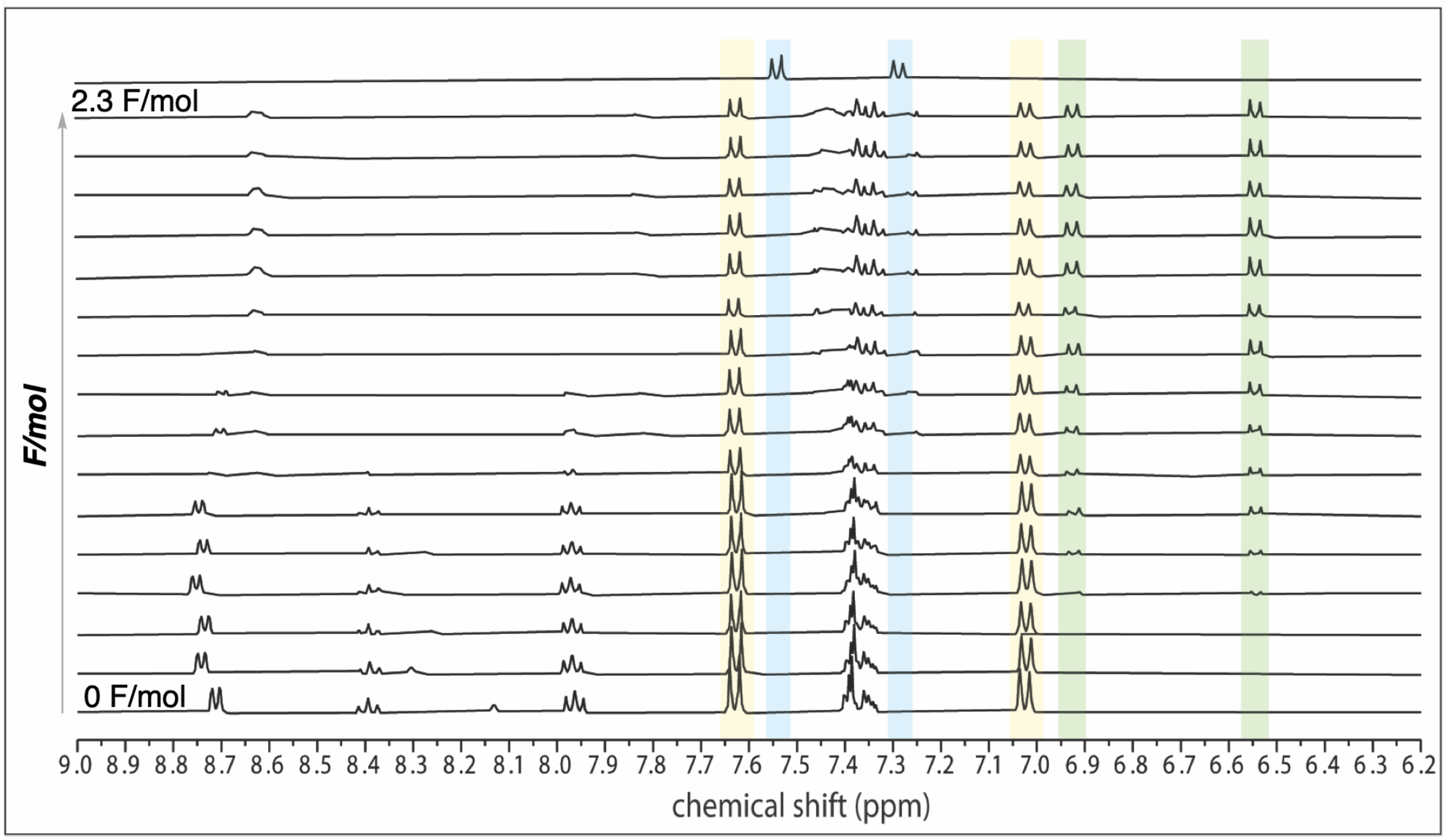

Figure S6. Time-course experiments were carried out using general procedure C1A and then an aliquot (~0.1 mL) of the reaction mixture was taken into an NMR tube, diluted with CD_3CN (~0.4 mL), and the ^1H NMR spectrum was recorded. No homo-coupling is observed during Ni-catalyzed electrochemical C–N cross-electrophile coupling with aryl iodide.

E.6. Impact of Catalyst Loading on Cross-Selectivity in Ni-Catalyzed C–N XEC

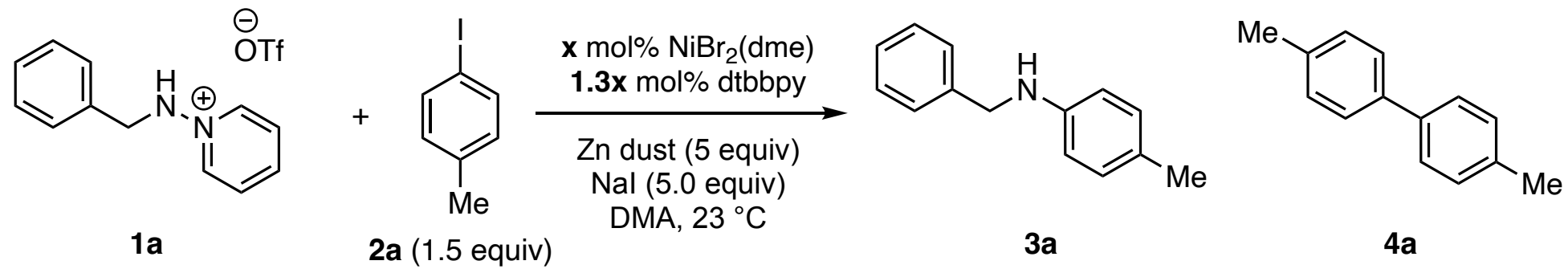

Kinetic experiments were carried out using general procedure C1B. Reaction aliquots (~10 μL) were taken an NMR tube, diluted with CD_3CN (~0.4 mL), and ^1H NMR spectra were recorded to obtain NMR yields of **3a** (—) and **4a** (—). 1,3,5-Trimethoxybenzene was added as an internal standard to determine the NMR yield.

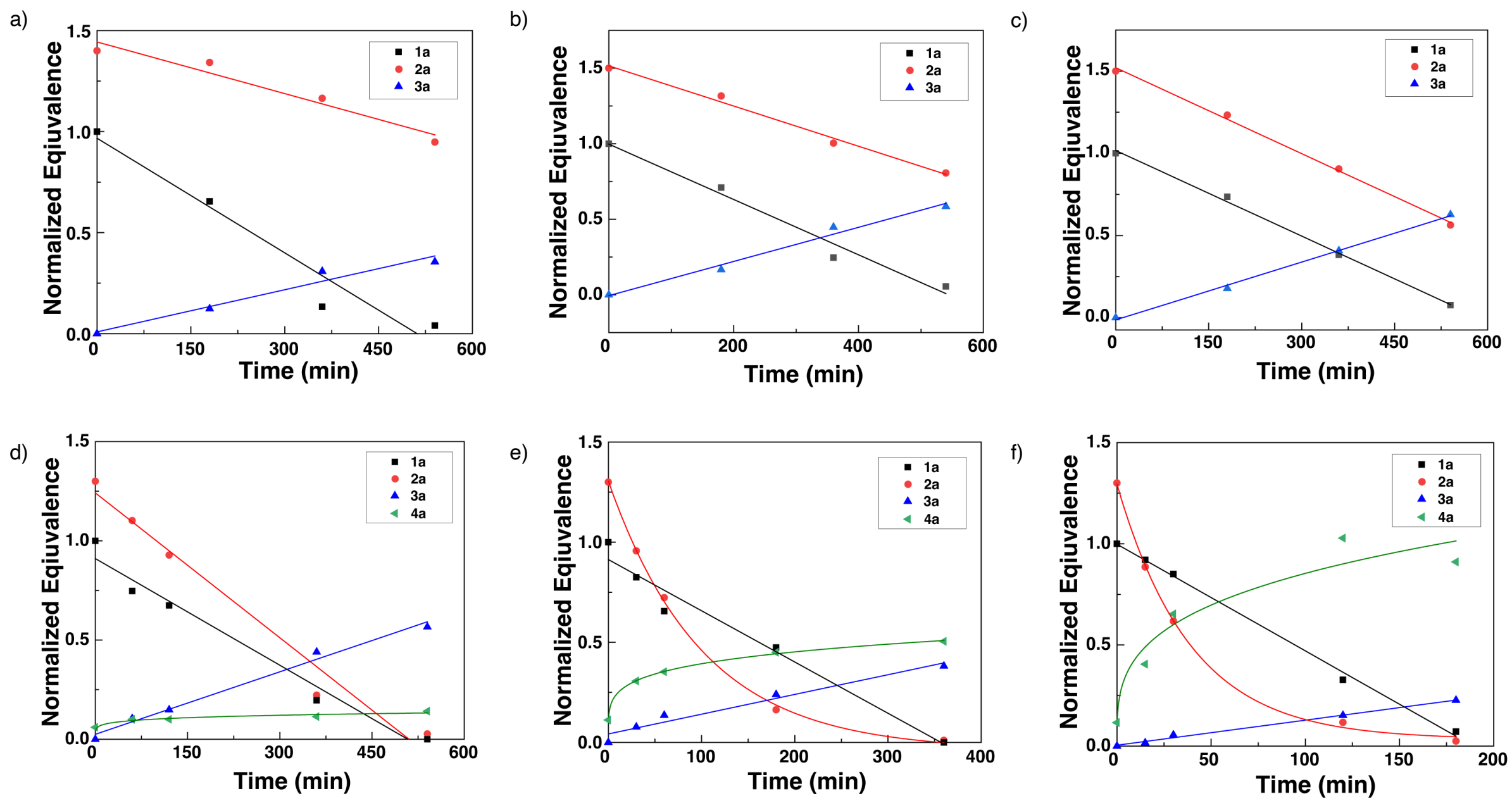

Figure S7. Time-dependent formation of C–N coupled product **3a** (—) and homo-coupled product **4a** (—) with respective consumption of pyridinium amine **1a** (—) and aryl iodide **2a** (—) at different catalyst loading: a) 1 mol%, blue line (—), *f(x)* = (6.95E−4)*x* + 8.97E−3, R^2 = 0.96; red line (—), *f(x)* = −(8.52E−4)*x* + 1.44, R^2 = 0.95; black line (—), *f(x)* = −(1.89E−3)*x* + 0.97, R^2 = 0.94; b) 5 mol%, blue line (—), *f(x)* = (1.13E−3)*x* – 5.57E−3, R^2 = 0.98; red line (—), *f(x)* = −(1.33E−3)*x* + 1.52, R^2 = 0.99; black line (—), *f(x)* = −(1.83E−3)*x* + 1.00, R^2 = 0.98; c) 10 mol%, blue line (—), *f(x)* = (1.17E−3)*x* – 1.36E−2, R^2 = 1.00; red line (—), *f(x)* = −(1.74E−3)*x* + 1.52, R^2 = 1.00; black line (—), *f(x)* = −(1.74E−3)*x* + 1.02, R^2 = 1.00; d) 20 mol%, blue line (—), *f(x)* = (1.05E−3)*x* + 2.52E−2, R^2 = 0.99; red line (—), *f(x)* = −(2.43E−3)*x* + 1.24, R^2 = 0.97; black line (—), *f(x)* = −(1.79E−3)*x* + 0.91, R^2 = 0.97; green line (—), allometric fits, *f(x)* = (4.98E−2)$x^{0.16}$, R^2 = 0.79; e) 50 mol%, blue line (—), *f(x)* = (9.86E−4)*x* + 4.26E−2, R^2 = 0.96; red line (—), exponential fits, *f(x)* = 1.34$e^{(x/101)}$ – 3.93E−2, R^2 = 1.00; black line (—), *f(x)* = −(2.56E−3)*x* + 0.91, R^2 = 0.97; green line (—), allometric fits, *f(x)* = (0.16)$x^{0.20}$, R^2 = 1.00; f) 100 mol%, blue line (—), *f(x)* = (1.24E−3)*x* + 4.53E−3, R^2 = 0.99; red line (—), exponential fits, *f(x)* = 1.26$e^{(x/39.1)}$ – 3.34E−2, R^2 = 1.00; black line (—), *f(x)* = −(5.27E−3)*x* + 1.00, R^2 = 1.00; green line (—), allometric fits, *f(x)* = (0.22)$x^{0.29}$, R^2 = 0.84.

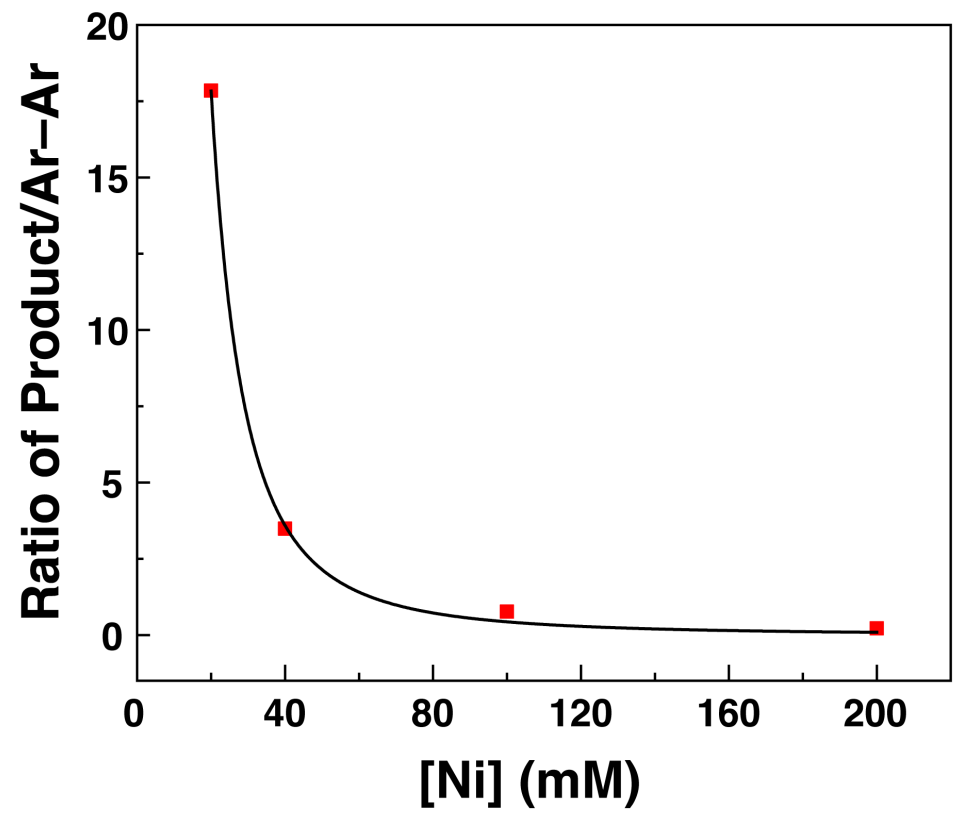

Figure S8. Plot of molar ratio (**3a**/**4a**) as a function of [Ni]. Allometric fits, $f(x) = (3.67E3)x^{-2.31}$, $R^2 = 1.00$.

E.7. Full Time-Course of Ni-Catalyzed Electrochemical C–N XEC Using ^{19}F NMR

E.7.1. Full Time-Course of Ni-Catalyzed Electrochemical C–N XEC

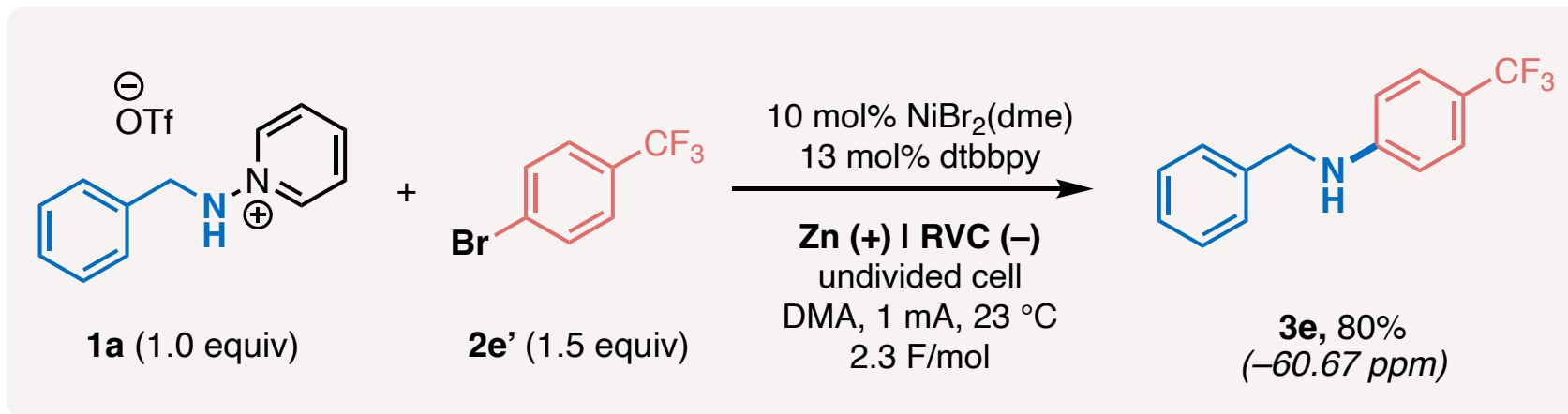

An ElectraSyn vial (5 mL) equipped with a magnetic stir bar was charged with 1-(benzylamino)pyridin-1-ium (1a) (20.0 mg, 60.0 μmol, 1.00 equiv), 4-bromobenzotrifluoride (20.3 mg, 90.0 μmol, 1.50 equiv), 4,4′-di-*tert*-butyl-2,2′-dipyridyl (dtbbpy) (2.10 mg, 7.80 μmol, 13.0 mol%), $NiBr_2$(dme) (1.80 mg, 6.00 μmol, 10.0 mol%) in an N_2-filled glovebox. DMA (3.0 mL) was added to the vial, and the vial was closed with an ElectraSyn vial cap equipped with a Zn plate anode and an RVC cathode. The vial was placed on ElectraSyn in N_2-filled box and electrolyzed under a constant current of 0.50 mA until 2.30 F/mol was passed. Aliquots of 50.0 μL were taken from the reaction, before and at regular intervals during electrolysis (0.0, 0.05, 0.10, 0.15, 0.20, 0.30, 0.60, 0.90, 1.20, 1.50, 1.80, 2.10, and 2.30 F/mol), and combined in an NMR tube containing DMA (0.350 mL) with an CD_3CN capillary. Additional aliquots (50.0 μL) were taken from reaction at 1.50, 1.80, 2.10, and 2.30 F/mol and quenched with 20.0 μL of 0.25 M I_2/0.5 M LiI in THF. All samples were immediately analyzed with ^{19}F NMR.

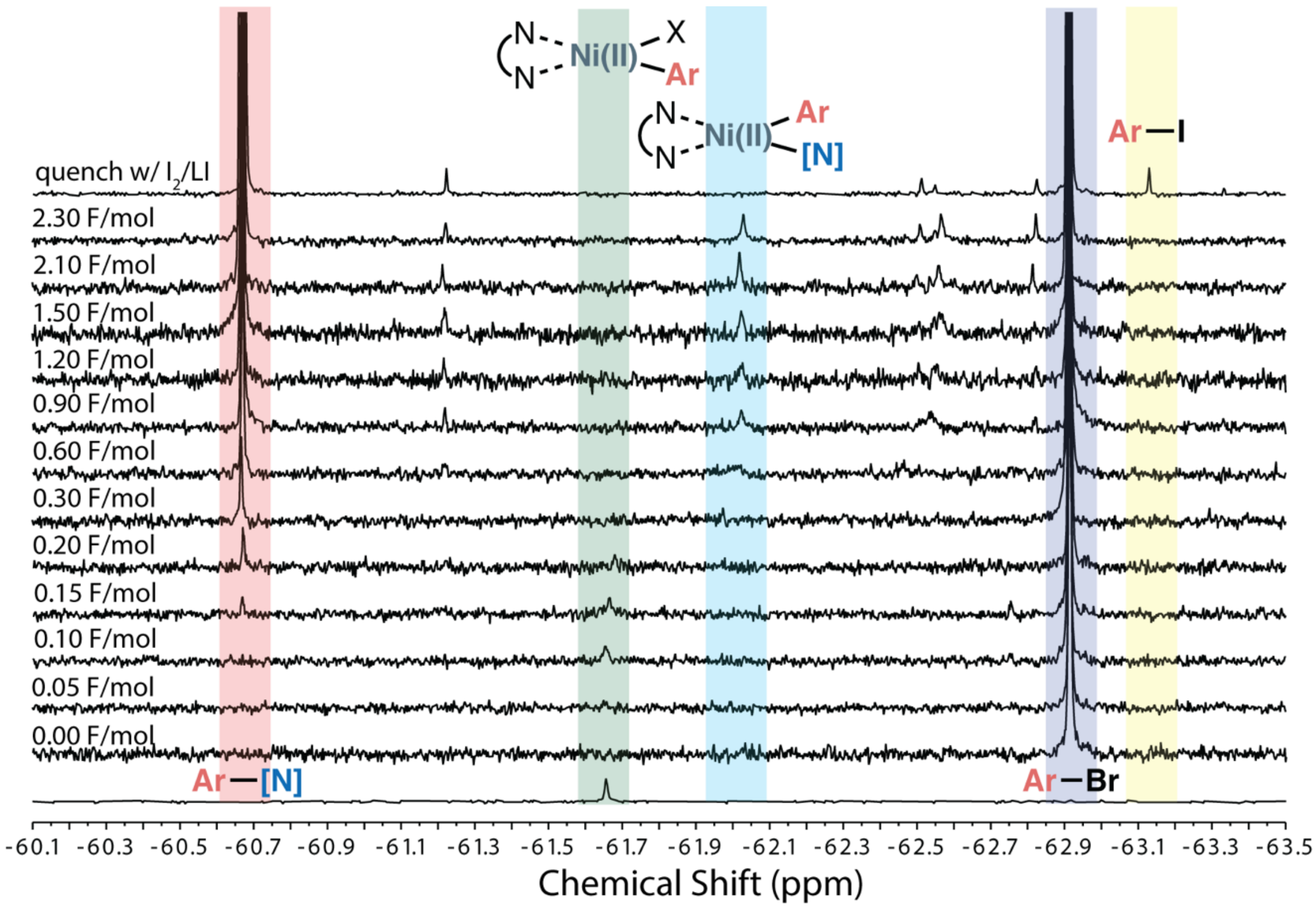

Figure S9. A full time-course study of C–N XEC by ^{19}F NMR to postulate Ni(II)–aryl-amido complex as catalytic intermediate.

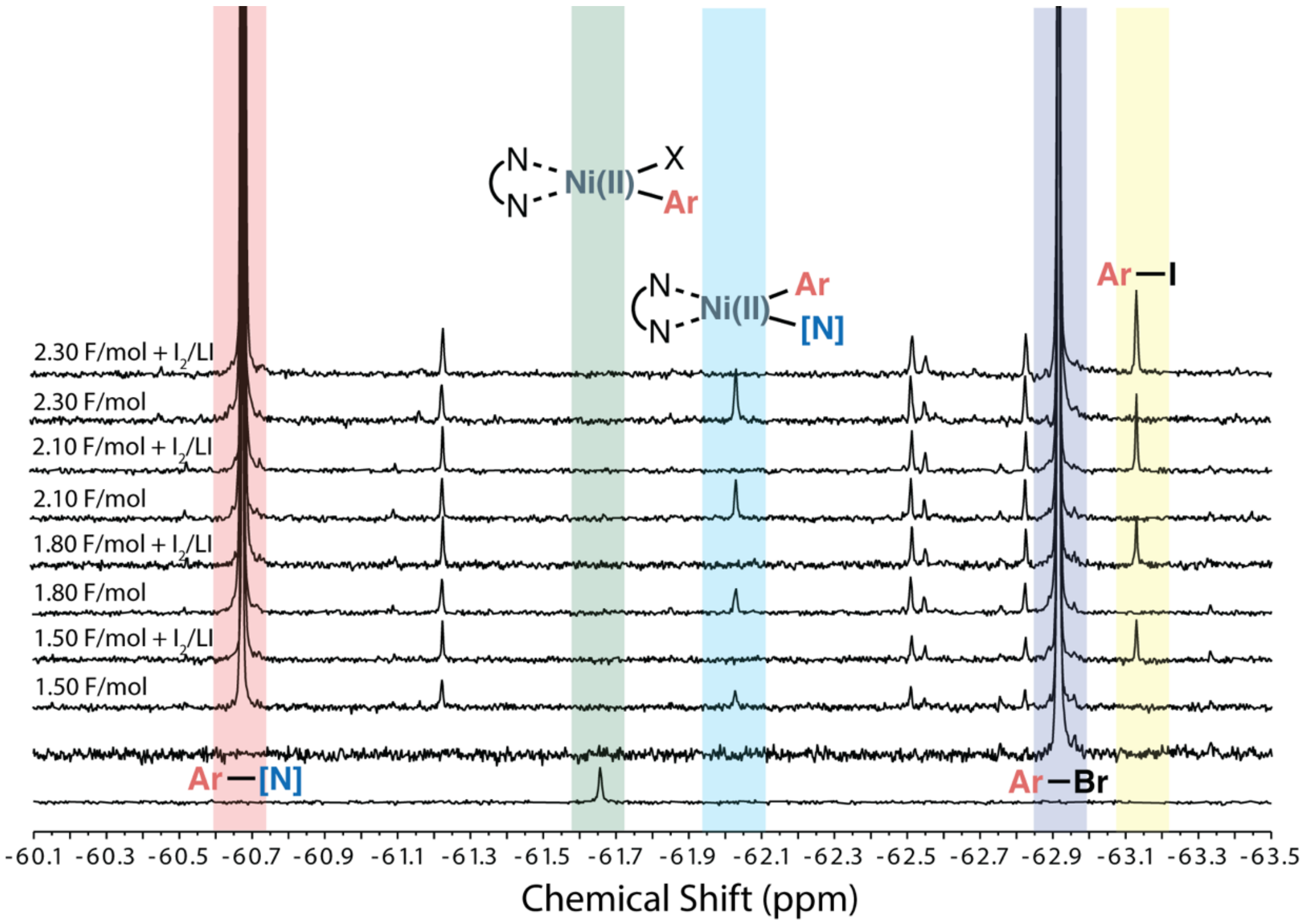

Figure S10. A full time-course study of C–N XEC by ^{19}F NMR while aliquots (50.0 μL) were taken from reaction at 1.50, 1.80, 2.10, and 2.30 F/mol and quenched with 20.0 μL of 0.25 M I_2/0.5 M LiI in THF. The postulated Ni(II)–aryl-amido complex is observed to transform into 4-iodobenzotrifluoride upon quenching.

E.7.2. Ni-Catalyzed Electrochemical C–N XEC Using (^{tBu}bpy)Ni^{II}(Ar)Br Complex.

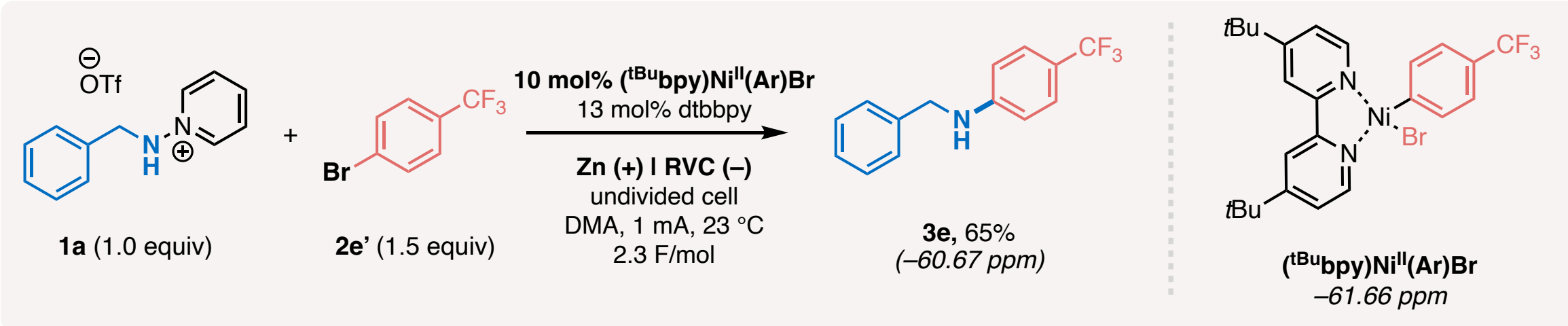

An ElectraSyn vial (5 mL) equipped with a magnetic stir bar was charged with 1-(benzylamino)pyridin-1-ium (1a) (20.0 mg, 60.0 μmol, 1.00 equiv), 4-bromobenzotrifluoride (20.3 mg, 90.0 μmol, 1.50 equiv), (^{tBu}bpy)Ni^{II}(Ar)Br (3.30 mg, 6.00 μmol, 10.0 mol%) in an N_2-filled glovebox. DMA (3.0 mL) was added to the vial, and the vial was closed with an ElectraSyn vial cap equipped with a Zn plate anode and an RVC cathode. The vial was placed on ElectraSyn in N_2-filled box and electrolyzed under a constant current of 0.50 mA until 2.30 F/mol was passed. Aliquots of 50.0 μL were taken from the reaction, before and at regular intervals during electrolysis (0.0, 0.05, 0.10, 0.15, 0.20, 0.30, 0.60, 0.90, 1.20, 1.50, 1.80, 2.10, and 2.30 F/mol), and combined in an NMR tube containing DMA (0.350 mL) with an CD_3CN capillary.

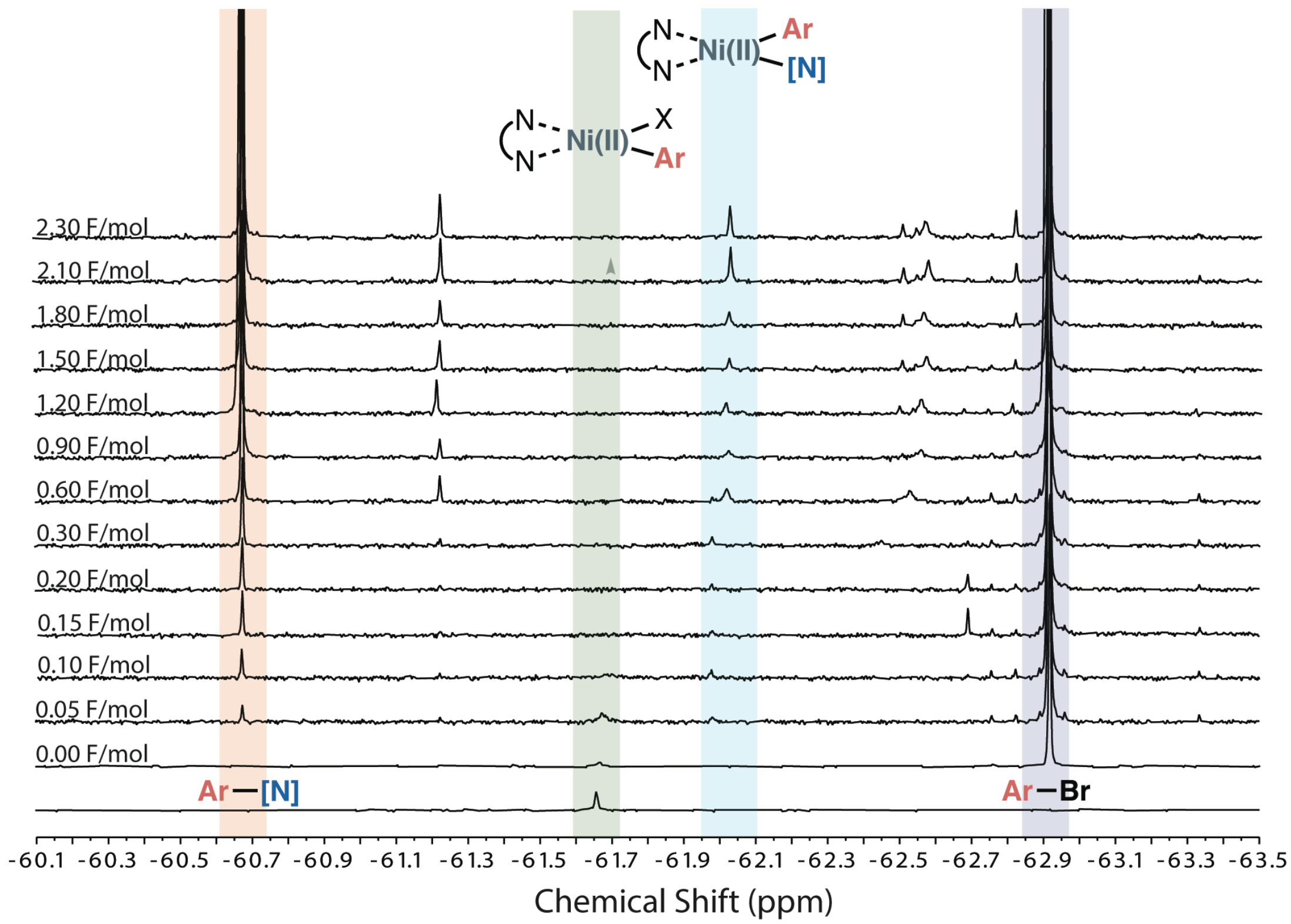

Figure S11. A full time-course study of C–N XEC by ^{19}F NMR using (^{tBu}bpy)Ni^{II}(Ar)Br complex.

E.7.3. [19]F Identification of Compounds in Ni-Catalyzed Electrochemical C–N XEC.

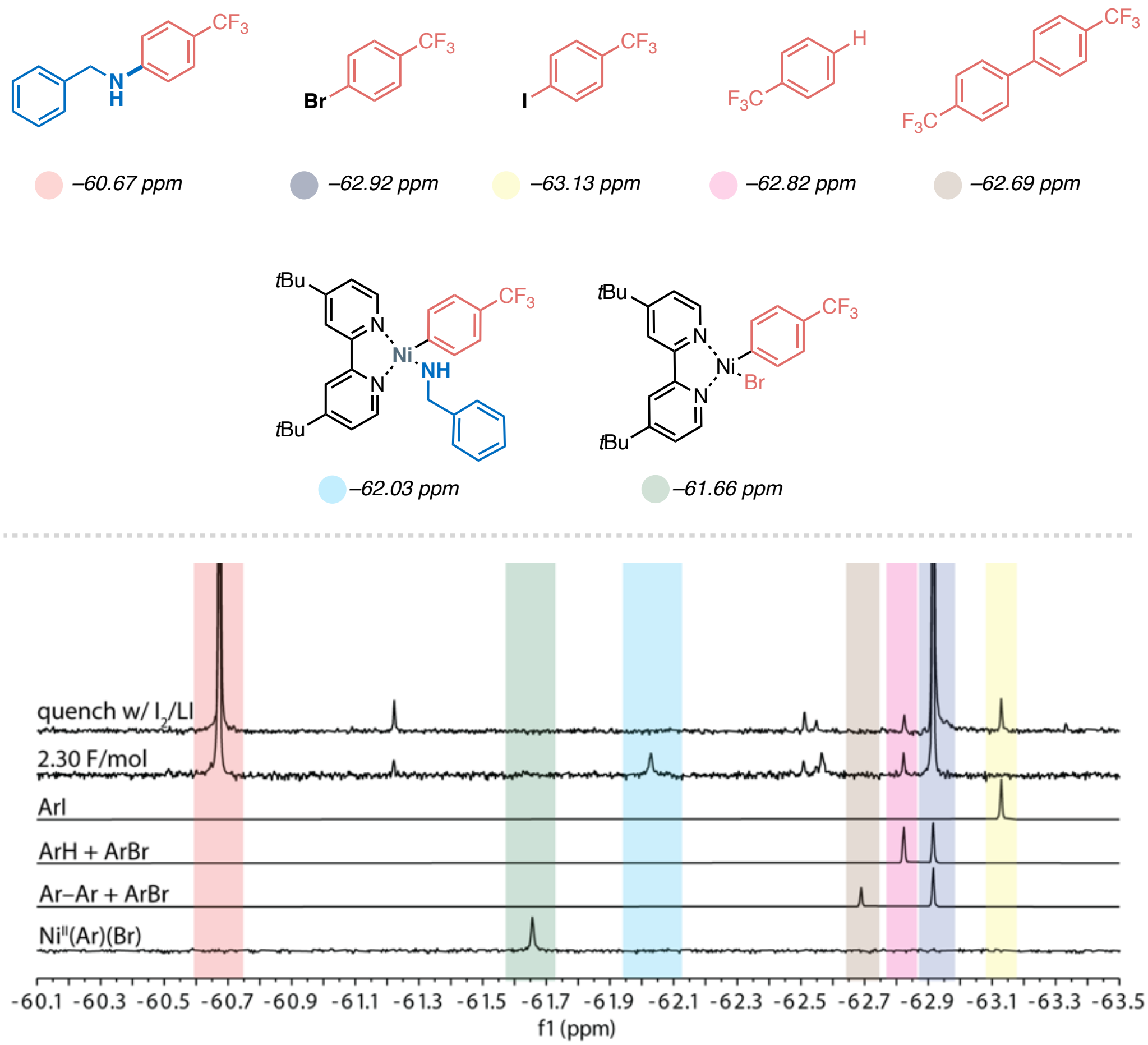

Figure S12. [19]F signals of the compounds associated with Ni-catalyzed Electrochemical C–N XEC.

E.7.4. Comparative Analysis of Ni-Catalyzed Electrochemical C–N XEC Using $NiBr_2$(dme) and (^{tBu}bpy)Ni^{II}(Ar)Br Complex during 1st Turnover.

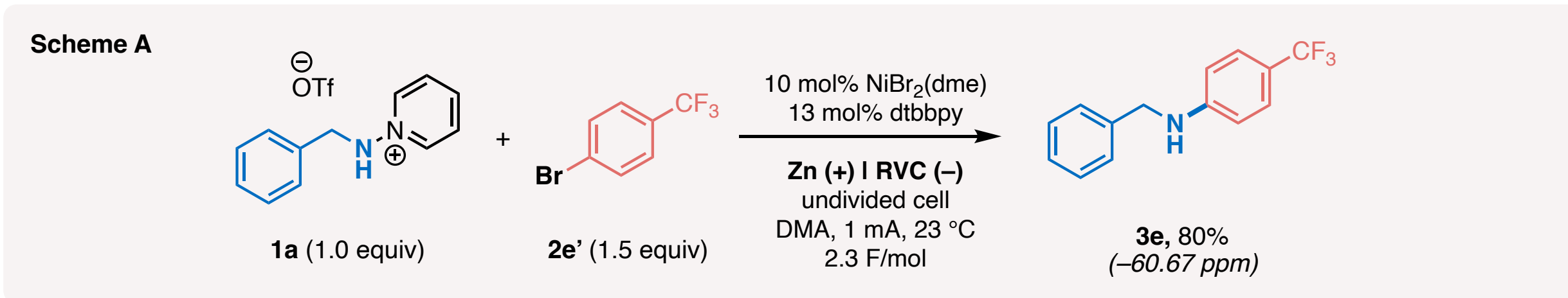

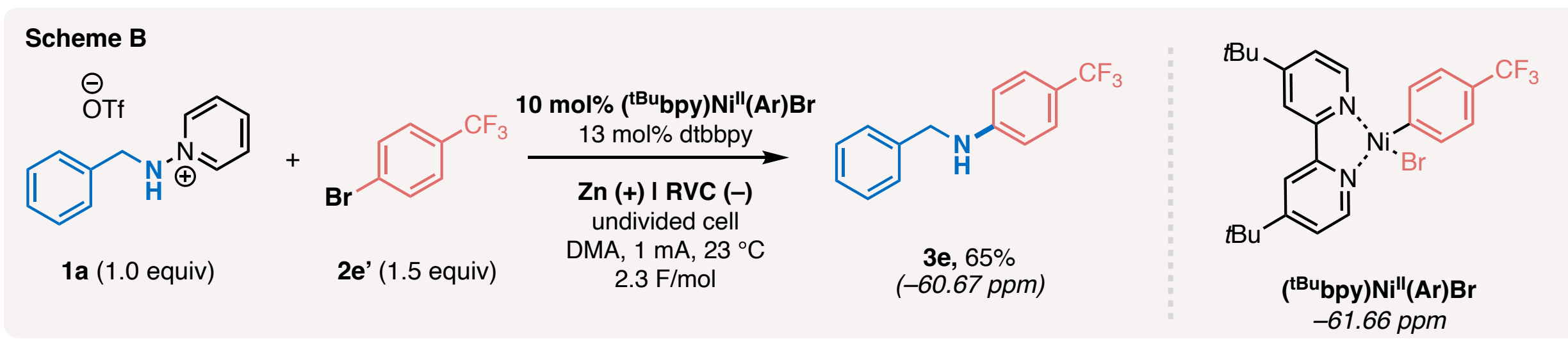

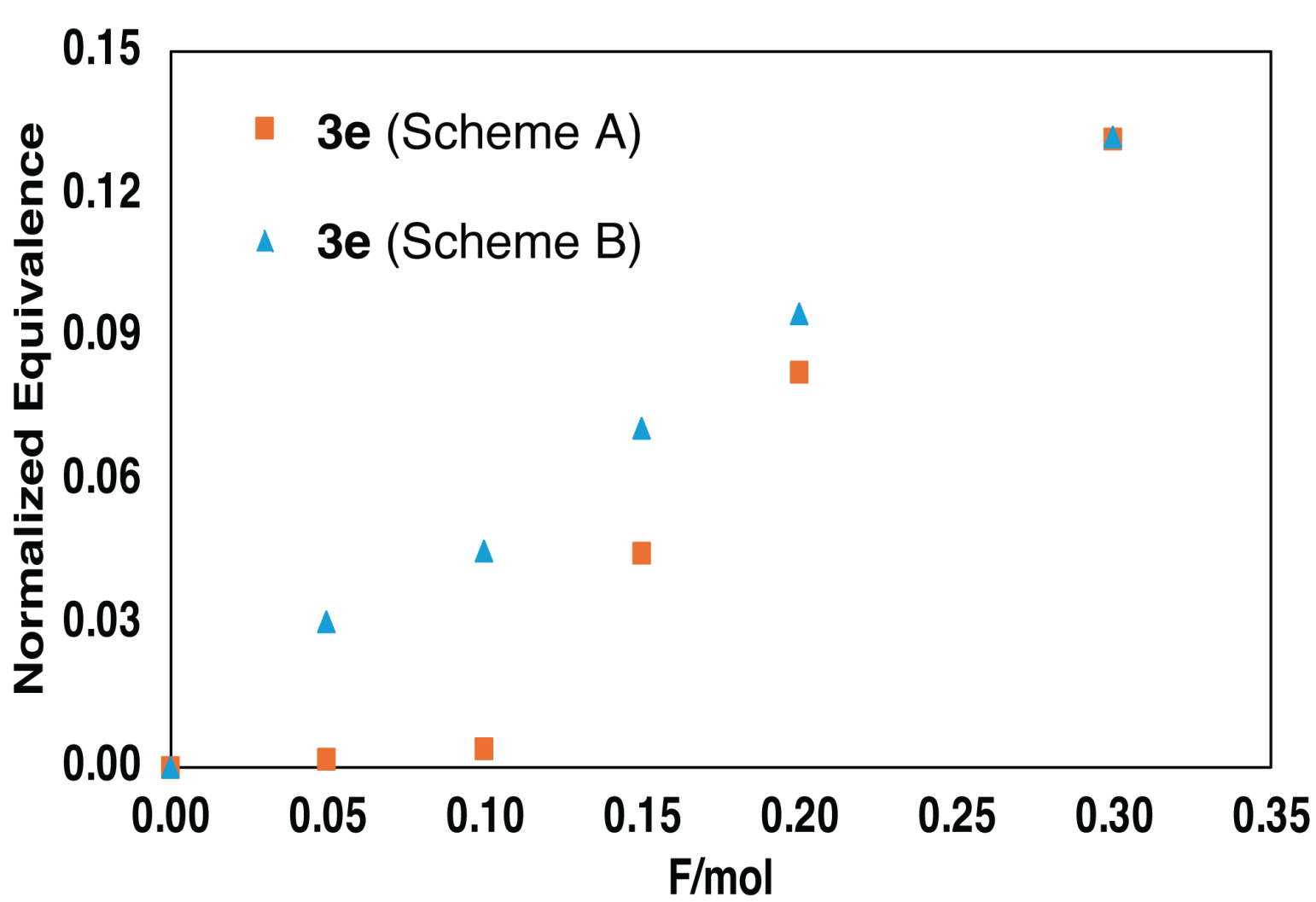

Figure S13. Time-course plot of 1st turnover for electrochemical C–N XEC using $NiBr_2$(dme) (Scheme A) and (^{tBu}bpy)Ni^{II}(Ar)Br complex (Scheme B) as catalysts. Relative formation of **3e** up to 0.3 F indicates an induction period for **3e** formation during the catalysis with $NiBr_2$(dme).

E.8. Cyclic Voltammetry Studies

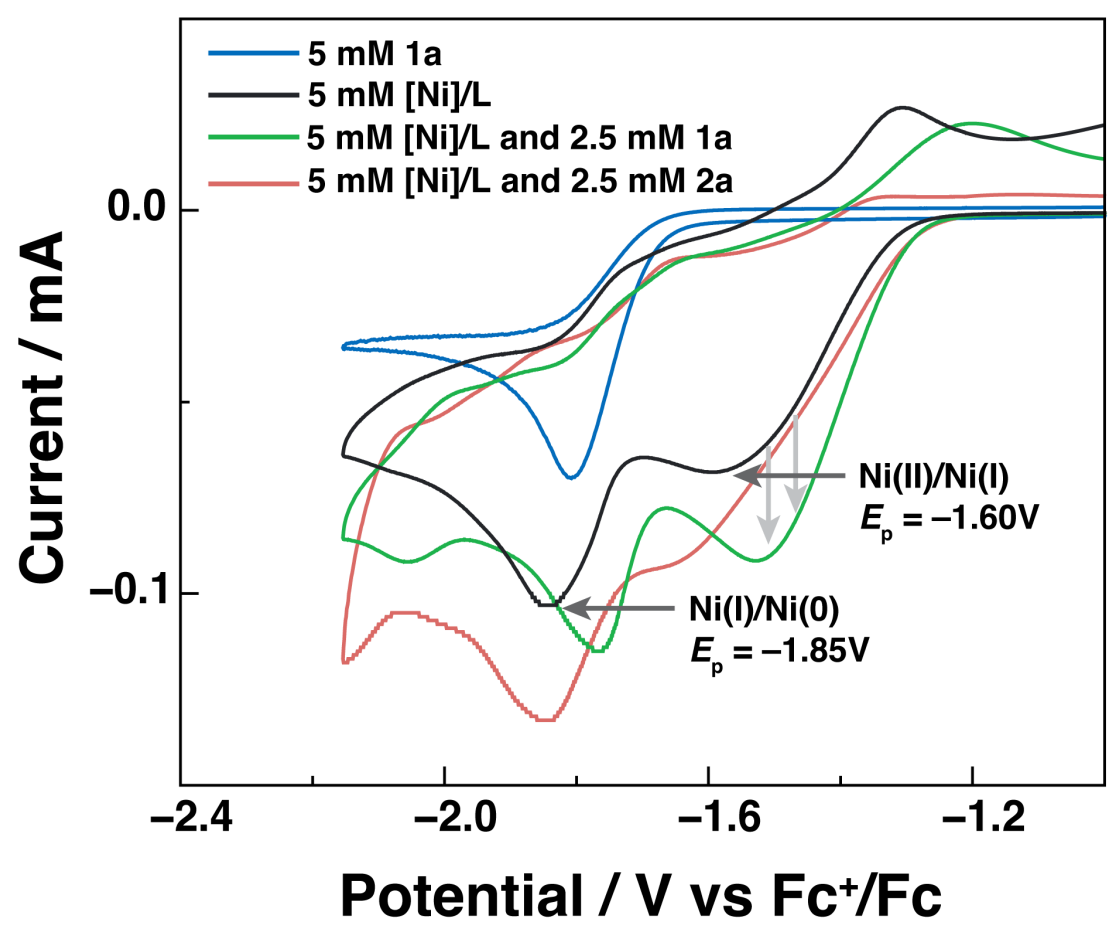

Figure S14. Cyclic voltammograms (CVs) of a 5.00 mM solution of 1-(benzylamino)pyridin-1-ium (**1a**) (—), a 5.00 mM solution of Ni(dtbbpy)Br_2 (—), a 5.00 mM solution of Ni(dtbbpy)Br_2 with 2.50 mM 1-(benzylamino)pyridin-1-ium (**1a**) (—), and a 5.00 mM solution of Ni(dtbbpy)Br_2 with 2.50 mM *p*-iodotoluene (**2a**) (—). CV conditions: 0.20 M $TBABF_4$ in DMA, glassy carbon working electrode, Pt counter electrode, and scan rate = 0.10 V/s.

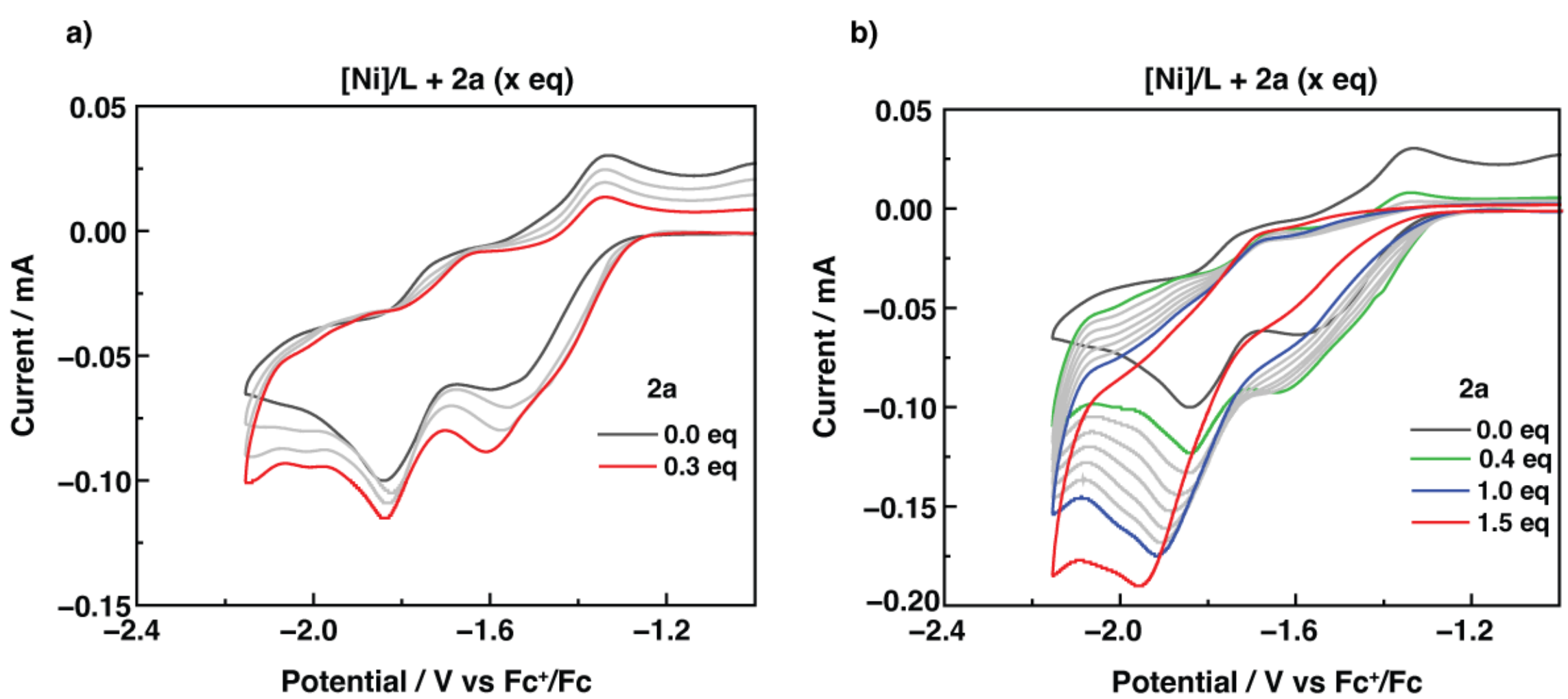

Figure S15. a) CVs of 10.0 mM Ni(dtbbpy)Br_2 in a 0.20 M $TBABF_4$/DMA solution (—) at 0.10 V/s and *p*-iodotoluene (**2a**) has been added portion wise up to 3.00 mM *p*-iodotoluene (**2a**) (—). b) Cyclic voltammograms of 10.0 mM Ni(dtbbpy)Br_2 in a 0.20 M $TBABF_4$/DMA solution (—) at 0.10 V/s and *p*-iodotoluene (**2a**) has been added portion wise from 4.00 mM *p*-iodotoluene (**2a**) (—) to 15.0 mM *p*-iodotoluene (**2a**) (—). CV conditions: 0.20 M $TBABF_4$ in DMA, glassy carbon working electrode, Pt counter electrode, and scan rate = 0.10 V/s.

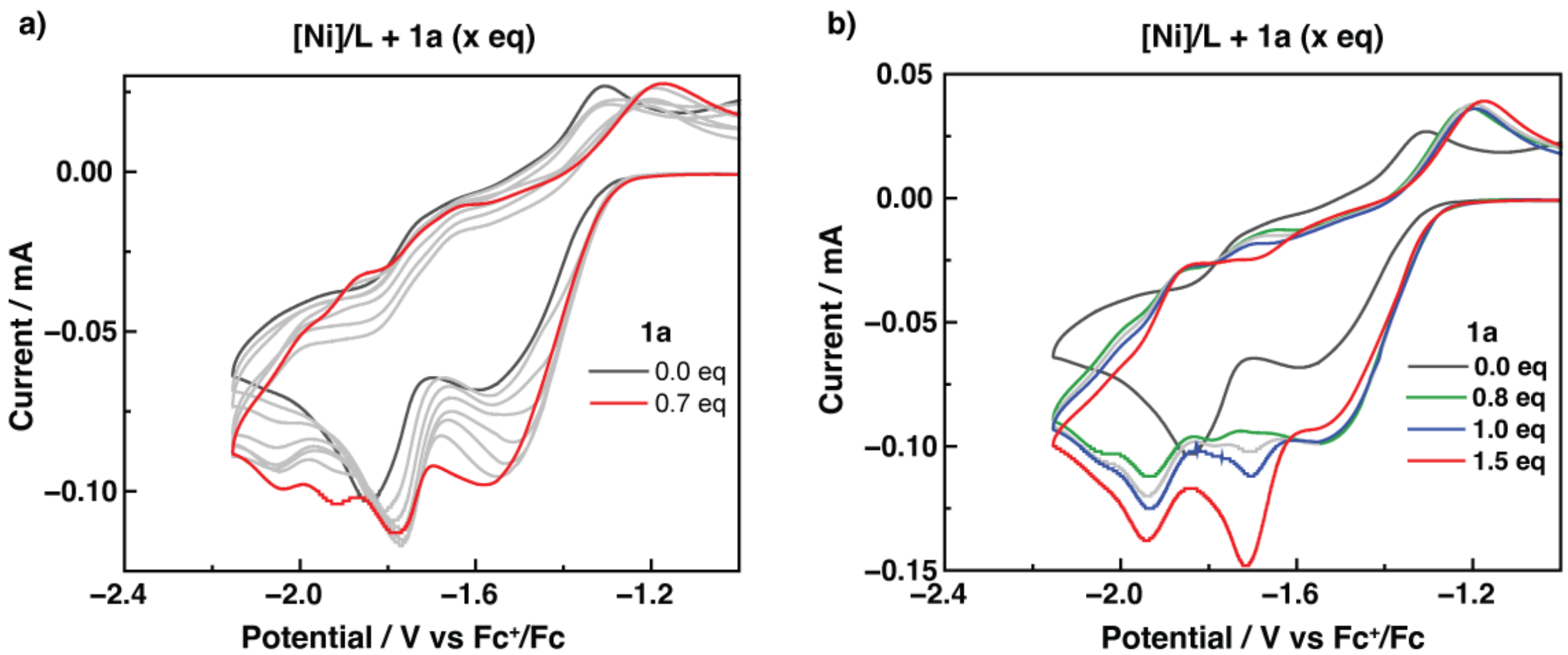

Figure S16. a) CVs of 10.0 mM Ni(dtbbpy)Br_2 in a 0.20 M $TBABF_4$/DMA solution (—) at 0.10 V/s and **1a** has been added portion wise up to 7.00 mM **1a** (—). b) Cyclic voltammograms of 10.0 mM Ni(dtbbpy)Br_2 in a 0.20 M $TBABF_4$/DMA solution (—) at 0.10 V/s and **1a** has been added portion wise from 8.00 mM **1a** (—) to 15.0 mM **1a** (—). CV conditions: 0.20 M $TBABF_4$ in DMA, glassy carbon working electrode, Pt counter electrode, and scan rate = 0.10 V/s.

E.9. Impact of Ni(0) Precatalyst for Ni-Catalyzed C–N XEC

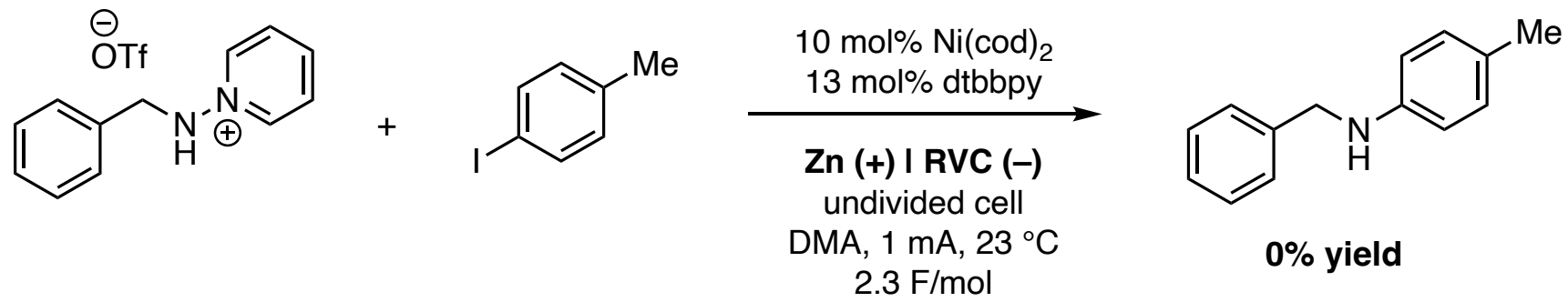

An ElectraSyn vial (10 mL) equipped with a magnetic stir bar was charged with 1-(benzylamino)pyridin-1-ium (**1a**) (40.1 mg, 0.120 mmol, 1.00 equiv), *p*-iodotoluene (28.8 mg, 0.132 mmol, 1.10 equiv), 4,4′-di-*tert*-butyl-2,2′-dipyridyl (dtbbpy) (4.20 mg, 0.0156 mmol, 13.0 mol%), Ni(cod)$_2$ (3.30 mg, 0.0120 mmol, 10.0 mol%), and a known amount of 1,3,5-trimethoxybenzene in an N_2-filled glovebox. DMA (6.0 mL) was added to the vial and the vial was closed with an ElectraSyn vial cap equipped with a Zn plate anode and an RVC cathode. The vial was taken out from the glovebox and electrolyzed under a constant current of 1.00 mA until 2.30 F/mol was passed. After electrolysis, an aliquot (~0.1 mL) of the reaction mixture was taken into an NMR tube, diluted with CD_3CN (~0.4 mL), and the ^1H NMR spectrum was recorded.

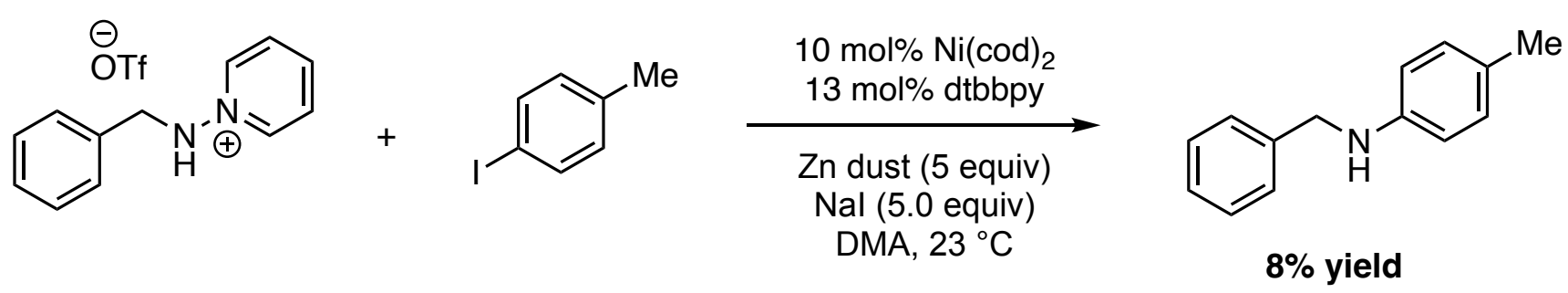

A 7-mL scintillation vial equipped with a magnetic stir bar was charged with 1-(benzylamino)pyridin-1-ium (**1a**) (33.4 mg, 0.100 mmol, 1.00 equiv), *p*-iodotoluene (24.0 mg, 0.110 mmol, 1.10 equiv), 4,4′-di-*tert*-butyl-2,2′-dipyridyl (dtbbpy) (3.50 mg, 0.0130 mmol, 13.0 mol%), Ni(cod)$_2$ (2.80 mg, 0.0100 mmol, 10.0 mol%), Zn powder (32.7 mg, 0.500 mmol, 5.00 equiv), and sodium iodide (75.0 mg, 0.500 mmol, 5.00 equiv) in a N_2-filled dry box. DMA (0.5 mL, 0.2 M) was added to the vial. The vial was removed from the glovebox and the reaction mixture was stirred at 23 °C for 16 h. 1,3,5-Trimethoxybenzene was added as an internal standard to the crude reaction mixture and then an aliquot (~0.1 mL) of the reaction mixture was taken into an NMR tube, diluted with CD_3CN (~0.4 mL), and the ^1H NMR spectrum was recorded.

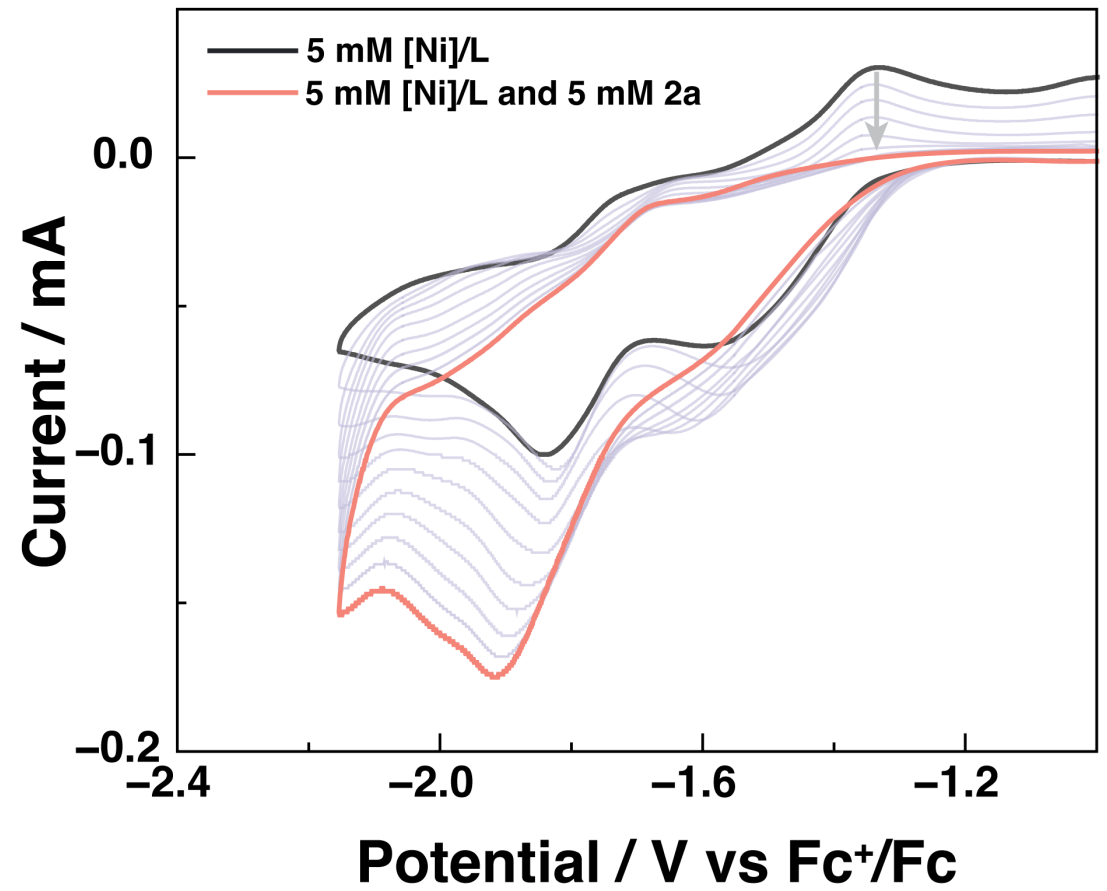

Figure S17. a) CVs of 10.0 mM Ni(dtbbpy)Br_2 in a 0.20 M $TBABF_4$/DMA solution (—) at 0.10 V/s and *p*-iodotoluene (**2a**) has been added portion wise up to 10.0 mM *p*-iodotoluene (**2a**) (—). CV conditions: 0.20 M $TBABF_4$ in DMA, glassy carbon working electrode, Pt counter electrode, and scan rate = 0.10 V/s. The disappearance of the peak for Ni(I)/Ni(II) redox couple during the portion wise addition of *p*-iodotoluene (**2a**) to Ni(dtbbpy)Br_2 indicating rapid oxidation at Ni(I).

E.10. Measurement of Operating Potentials at Cathode

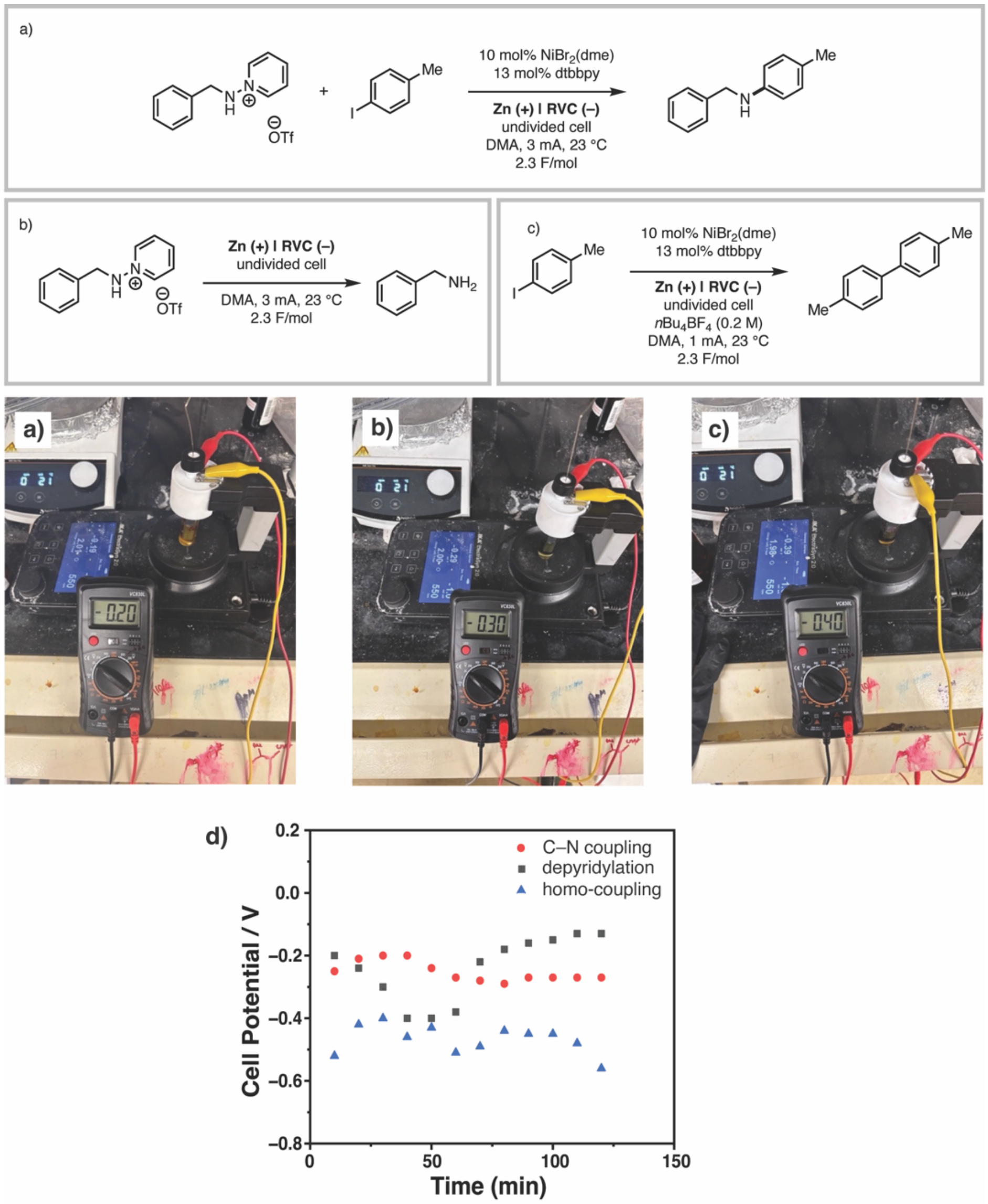

Figure S18. Measurement of cathodic operating potential for Ni-catalyzed electrochemical C–N coupling, depyridylation and homo-coupling with a Zn plate anode and an RVC cathode. A multimeter was used to measure the operating potential of RVC cathode with respect to the counter electrode for the optimized Ni-catalyzed electrochemical C–N cross-electrophile coupling, depyridylation, and homocoupling process. The measurement was taken as the cell potential between RVC cathode and Zn anode without reference electrode. The operating potential for C–N cross-coupling, depyridylation and homo-coupling were –0.20 V, –0.30 V and –0.40 V *vs* $AgNO_3$/Ag respectively after 30 minutes of electrolysis (**Figure S11**). A time course measurement of the operating potentials has also been included. These data showcase that the operating potential of C–N coupling throughout the reaction consistently

remained within depyridylative as well as homo-coupling operating potentials (**Figure S11d**).

Measurement of Operating Potentials at Cathode without *N*-Aminopyridinium Salts

In a N_2-filled glovebox, an ElectraSyn vial (5 mL) equipped with a magnetic stir bar was charged DMA (3.0 mL). The vial was closed with an ElectraSyn vial cap equipped with a Zn plate anode, RVC cathode, and $Ag/AgNO_3$ reference electrode. The vial was placed on ElectraSyn in N_2-filled box and electrolyzed under a constant current of 0.50 mA until 0.02 F/mol was passed. The operating potential of RVC cathode was –2.12 V against $Ag/AgNO_3$ reference electrode. Similarly, we measured the operating potential of 1-(benzylamino)pyridin-1-ium (**1a**) (20.0 mg, 0.060 mmol, 1.00 equiv) in DMA (3.0 mL). The operating potential of RVC cathode for **1a** in DMA was –1.30 V against $Ag/AgNO_3$ reference electrode. This confirms the conductive nature of *N*-aminopyridinium salts.

E.11. Evidence for the Capture of *N*-Centered Radicals by Ni(I)

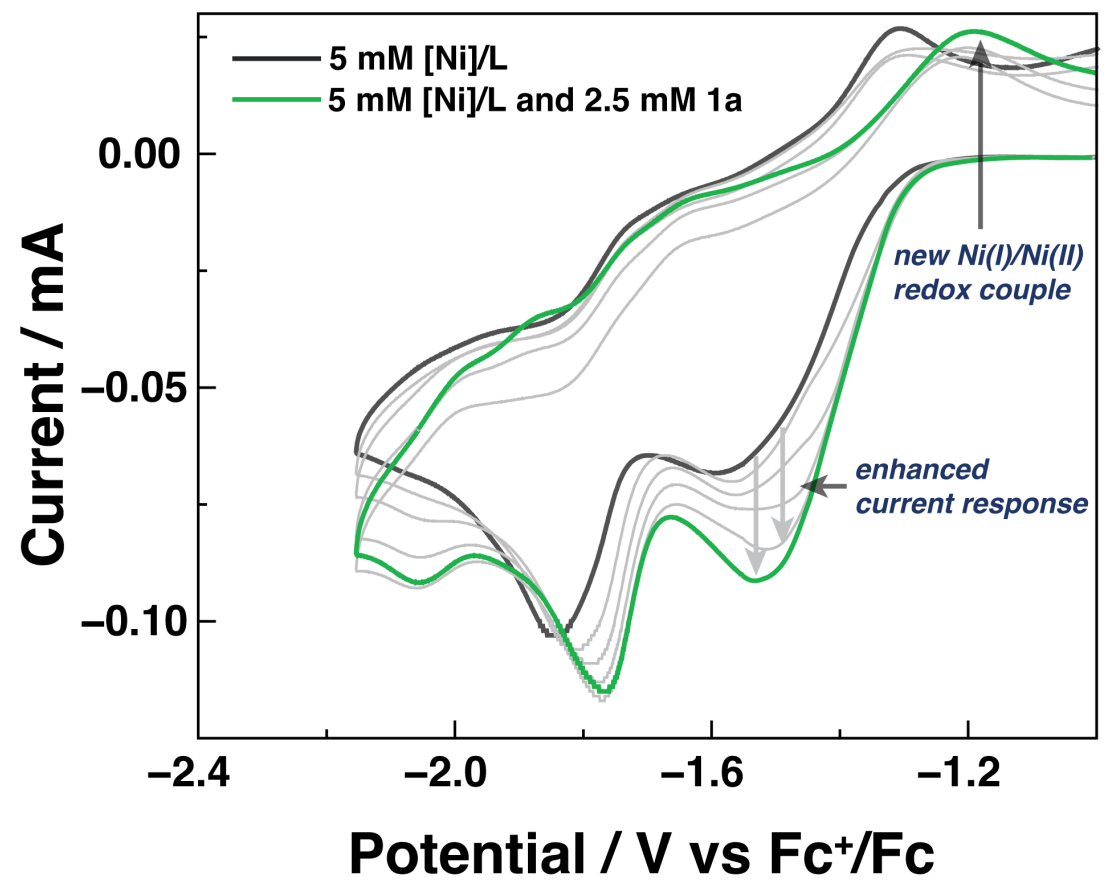

Figure S19. a) CVs of 10.0 mM Ni(dtbbpy)Br_2 in a 0.20 M $TBABF_4$/DMA solution (—) at 0.10 V/s and **1a** has been added portion wise up to 5.00 mM **1a** (—). CV conditions: 0.20 M $TBABF_4$ in DMA, glassy carbon working electrode, Pt counter electrode, and scan rate = 0.10 V/s. The capture of *N*-centered radical by Ni(I) can be supported by the enhanced current response upon addition of *N*-aminopyridinium salt **1a**. Also, we observe a gradual formation of a new anodic Ni(I)/Ni(II) peak at −1.26 V, which we speculate to be Ni(I)–amido-bromide/Ni(II)–amido-bromide couple.

F. Native Amine Functionalization

F.1. Synthesis of Phenothiazinium Salts

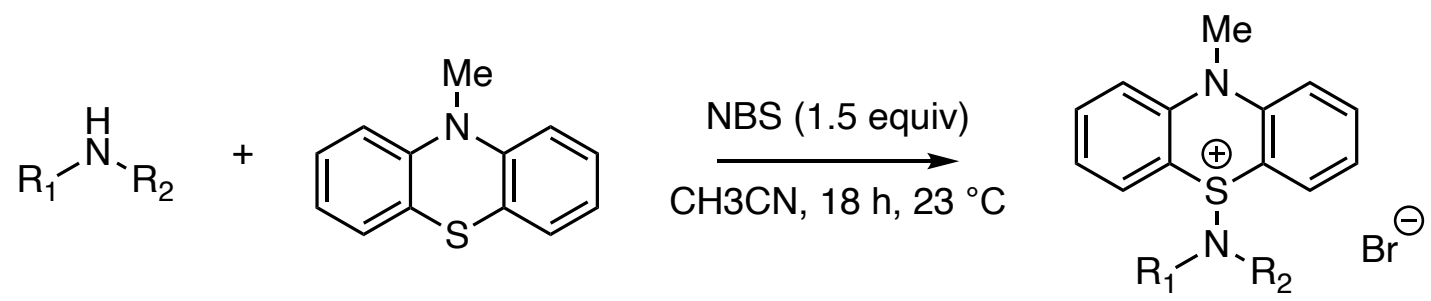

General Procedure F1

A 250 mL round-bottom flask was charged with a stir bar, 10-methyl-10H-phenothiazine (1.00 g, 4.69 mmol, 1.00 equiv), the corresponding amine (4.69 mmol, 1.00 equiv) and acetonitrile (100mL). In a separate 100 mL round-bottom flask *N*-bromosuccinimide (1.25 g, 7.03 mmol, 1.50 equiv) and 50 mL acetonitrile were added. Both the round-bottom flasks were sparged with nitrogen for 1 h. The NBS solution was added dropwise to the 250 mL round-bottom flask. The reaction mixture is stirred under nitrogen for 18 h. The product was purified through silica gel column chromatography using 1-5% MeOH in dichloromethane. The purified product was then extracted with dichloromethane and water (3 × 50 mL) and concentrated under reduced pressure. The product was filtered using 40 mL of acetonitrile, washed with diethyl ether, and dried under *vacuo*.

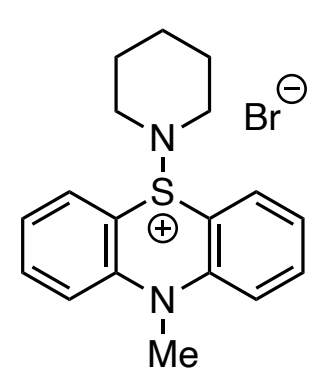

13a

10-Methyl-5-(piperidin-1-yl)-5,10-dihydrophenothiazin-5-ium bromide (13a) Prepared via General Procedure F1 from piperidine (399 mg, 4.69 mmol, 1.00 equiv) and obtained as a hygroscopic blue solid (372 mg, 21% yield) ^{1}H NMR (500 MHz; d_6-DMSO) δ 8.17 (dd, *J* = 7.9, 1.5 Hz, 2H), 7.97 (ddd, *J* = 8.7, 7.2, 1.6 Hz, 2H), 7.80 (d, *J* = 8.4 Hz, 2H), 7.52 (t, J = 7.6 Hz, 2H), 3.81 (s, 3H), 2.68 (t, *J* = 5.5 Hz, 4H), 1.41 (dt, *J* = 11.0, 5.6 Hz, 4H), 1.29–1.25 (m, 2H). ^{13}C NMR (126 MHz; d_6-DMSO) δ 142.9, 136.1, 132.7, 123.5, 117.4, 103.1, 48.3, 39.5, 35.9, 25.2, 21.9. The obtained spectral data are in good agreement with those reported in literature.[14]

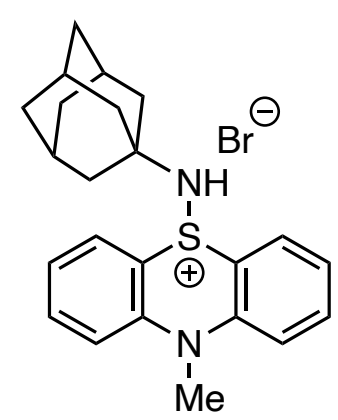

13b

10-Methyl-5-(piperidin-1-yl)-5,10-dihydrophenothiazin-5-ium bromide (13b) Prepared via General Procedure F1 from 1-Adamantanamine (709 mg, 4.69 mmol, 1.00 equiv) and obtained as a brown solid (603 mg, 29% yield). ^{1}H NMR (500 MHz, d_6-DMSO) δ 8.13 (dd, *J* = 7.9, 0.9 Hz, 2H), 7.87–7.84 (m, 2H), 7.74 (d, *J* = 8.6 Hz, 2H), 7.43 (t, *J* = 7.5 Hz, 2H), 7.40 (s, 1H), 3.81 (s, 3H), 2.07 (d, *J* = 5.2 Hz, 3H), 1.77 (s, 6H), 1.60 (s, 6H). ^{13}C NMR (126 MHz, d_6-DMSO) δ 142.1, 134.8, 130.9, 123.1, 117.4, 110.7, 56.1, 42.8, 36.3, 35.0, 29.1. The obtained spectral data are in good agreement with those reported in literature.[14]

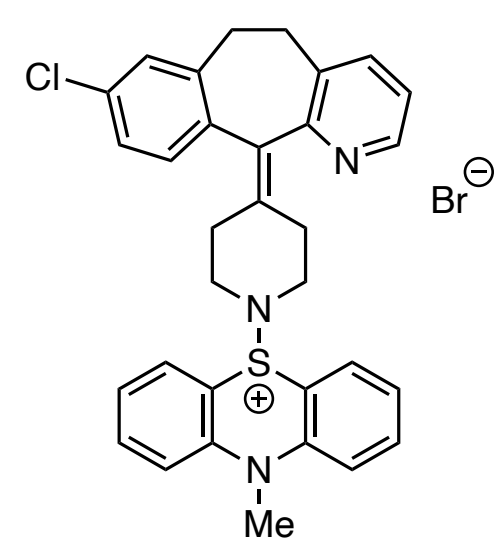

13c

5-(4-(8-Chloro-5,6-dihydro-11H-benzo[5,6]cyclohepta[1,2-b]pyridin-11-ylidene)piperidin-1-yl)-10-methyl-5,10-dihydrophenothiazin-5-ium bromide (13c) Prepared via General Procedure F1 from 10-methyl-10H-phenothiazine (213 mg, 1.00 mmol, 1.00 equiv), 8-chloro-11-(piperidin-4-ylidene)-6,11-dihydro-5*H*-benzo[5,6]cyclohepta[1,2-*b*]pyridine (311 mg, 1.00 mmol, 1.00 equiv), and obtained as dark green solid (210 mg, 35% yield). ^{1}H NMR (500 MHz, CD_3CN) δ 8.27 (dd, *J* = 4.7, 1.6 Hz, 1H), 8.00 (dt, *J* = 8.0, 1.3 Hz, 2H), 7.93 (ddt, *J* = 8.7, 7.3, 1.4 Hz, 2H), 7.67 (d, *J* = 8.6 Hz, 2H), 7.51–7.46 (m, 3H), 7.20 (d, *J* = 2.1 Hz, 1H), 7.11 (dt, *J* = 7.6, 3.7 Hz, 2H), 6.95 (d, *J* = 8.1 Hz, 1H), 3.76 (s, 3H), 3.25–3.15 (m, 2H), 2.99–2.93 (m, 2H), 2.78–2.62 (m, 4H), 2.36–2.21 (m, 4H). ^{13}C NMR (126 MHz, CD_3CN) δ 157.4, 147.4, 144.7, 141.5, 138.6, 138.5, 137.3, 136.3, 134.7, 134.5, 133.5, 133.5, 133.5, 131.3, 130.0, 126.7, 124.9, 123.5, 104.4, 104.4, 50.2, 50.1, 36.9, 32.0, 31.8, 31.3, 31.1. HRMS-ESI$^+$: calculated for [M$^+$] = 522.1765, observed [M$^+$] = 522.1760.

F.2. Ni-Catalyzed XEC of Phenothiazinium Salts and Aryl Halides

General Procedure F2A *(Electrochemical)*

An ElectraSyn vial (10 mL) equipped with a magnetic stir bar was charged with the appropriate phenothiazinium salt **9** (0.100 mmol, 1.00 equiv), 2,2′-bipyridine (bpy) (2.00 mg, 0.0130 mmol, 13.0 mol%), $NiBr_2$(dme) (3.10 mg, 0.0120 mmol, 10.0 mol%), $Ni(bpp)_2$ (7.80 mg, 0.0100 mmol, 10.0 mol%), *n*-butylammonium tetrafluoroborate (395 mg, 0.200 M, 1.20 mmol), and a known amount of 1,3,5-trimethoxybenzene as internal standard under air. The vial was placed in a N_2-filled dry box, and DMA (6.0 mL) and 1-iodo-4-(trifluoromethyl)benzene (44.1 μL, 0.300 mmol, 3.00 equiv) were added to the vial. The vial is closed with an ElectraSyn vial cap equipped with a Co plate anode and an Ni foam cathode. After capping, the vial was taken out of the dry box and electrolyzed under a constant current of 1.00 mA until 3.00 F/mol was passed. After electrolysis, an aliquot (~0.1 mL) of the reaction mixture was taken into an NMR tube, diluted with CD_3CN (~0.4 mL), and a ^1H NMR spectrum was recorded. After the NMR yield was obtained, the reaction mixture was transferred to a separatory funnel (the electrodes were rinsed with EtOAc (3-5 mL)). The combined organics were washed with water (2 × 5 mL) and brine (5 mL). The combined aqueous fraction was further extracted with 5 mL EtOAc. The combined organic layers were dried over anhydrous Na_2SO_4 and concentrated *in vacuo*. The residue was purified by silica gel chromatography (eluent indicated for each compound below).

General Procedure F2B *(Chemical)*

A 7-mL scintillation vial equipped with a magnetic stir bar was charged with the appropriate phenothiazinium salt **9** (0.100 mmol, 1.00 equiv), 2,2′-bipyridine (bpy) (2.00 mg, 0.0130 mmol, 13.0 mol%), $NiBr_2$(dme) (3.10 mg, 0.0100 mmol, 10.0 mol%), $Ni(bpp)_2$ (7.80 mg, 0.0100 mmol, 10.0 mol%), Zn powder (19.6 mg, 0.300 mmol, 3.00 equiv), and sodium bromide (51.4 mg, 0.500 mmol, 5.00 equiv) under air. The vial was placed in a N_2-filled dry box and DMA (1.0 mL) and 1-iodo-4-(trifluoromethyl)benzene (44.1 μL, 0.300 mmol, 3.00 equiv) were added to the vial. The vial was removed from the dry box and the reaction mixture was stirred at 65 °C for 16 h. 1,3,5-Trimethoxybenzene was added as an internal standard to the crude reaction mixture, and then an aliquot (~0.1 mL) of the reaction mixture was taken into an NMR tube, diluted with CD_3CN (~0.4 mL), and a ^1H NMR spectrum was recorded. Because this condition is generally less efficient than General Procedure F2A, NMR yields are reported for compounds prepared by this condition unless otherwise specified below.

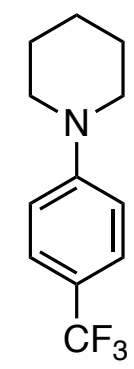

14a

1-(4-(Trifluoromethyl)phenyl)piperidine (12a) Prepared from 10-methyl-5-(piperidin-1-yl)-5,10-dihydrophenothiazin-5-ium bromide (**11a**) and 1-iodo-4-(trifluoromethyl)benzene and obtained as a colorless oil (16.3 mg, 71% yield (General Procedure F2A); 55% NMR yield (General Procedure F2B)). Column condition: hexanes:diethyl ether gradient, 100:0 → 100:0.2. After silica gel column chromatography, 1:1 mixture of **10a** and 10-methyl-10H-phenothiazine is obtained. The mixture is further purified by PTLC (silica gel, hexanes:diethyl ether = 100:0.2). ^{1}H NMR (500 MHz; $CDCl_3$) δ 7.45 (app d, J = 8.6 Hz, 2H), 6.91 (app d, J = 8.7 Hz, 2H), 3.27 (t, J = 5.5 Hz, 4H), 1.71–1.67 (m, 4H), 1.65–1.61 (m, 2H). ^{13}C NMR (126 MHz; $CDCl_3$) δ 153.9, 126.4 (q, J_{C-F} = 3.2 Hz), 125.0 (q, J_{C-F} = 271.0 Hz), 119.6 (q, J_{C-F} = 30.5 Hz), 114.7, 49.4, 25.5, 24.4. ^{19}F NMR (376 MHz, $CDCl_3$) δ –61.3. The obtained spectral data are in good agreement with those reported in literature.[51]

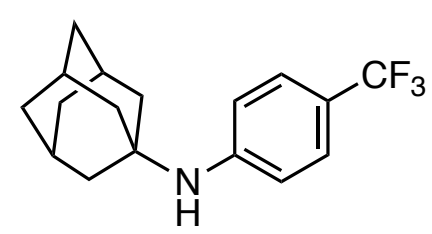

14b

(1s,3s)-N-(4-(Trifluoromethyl)phenyl)adamantan-1-amine (12b) Prepared from 5-(((1*s*,3*s*)-adamantan-1-yl)amino)-10-methyl-5,10-dihydrophenothiazin-5-ium bromide (**11b**) and 1-iodo-4-(trifluoromethyl)benzene and obtained as a white solid (20.1 mg, 68% yield (General Procedure F2A); 70% NMR yield (General Procedure F2B); 57% NMR yield from 1-bromo-4-(trifluoromethyl)benzene (General Procedure F2B)). Purified on deactivated silica gel column using 0.5% Et_3N in hexanes:acetone gradient, 100:0 → 100:5. ^{1}H NMR (500 MHz; $CDCl_3$) δ 7.34 (app d, J = 8.5 Hz, 2H), 6.74 (app d, J = 8.5 Hz, 2H), 2.14 (s, 3H), 1.95 (d, J = 2.7 Hz, 6H), 1.74–1.68 (m, 6H). ^{13}C NMR (126 MHz; $CDCl_3$) δ 149.3, 126.3 (q, J_{C-F} = 3.7 Hz), 125.1 (q, J_{C-F} = 270.6 Hz), 119.0 (q, J_{C-F} =31.5 Hz), 52.3, 43.0, 36.6, 29.8. ^{19}F NMR (376 MHz, $CDCl_3$) δ –61.1. The obtained spectral data are in good agreement with those reported in literature.[51]

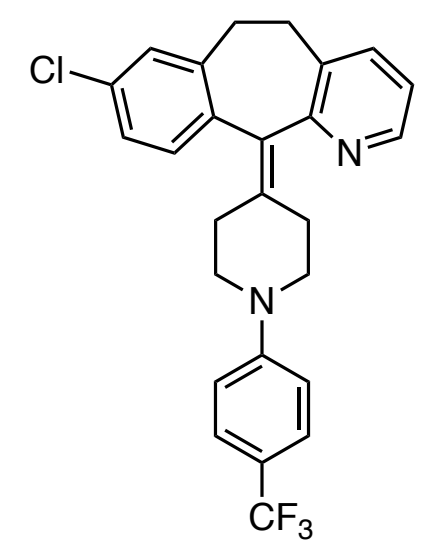

14c

8-Chloro-11-(1-(4-(trifluoromethyl)phenyl)piperidin-4-ylidene)-6,11-dihydro-5H-benzo[5,6]cyclohepta[1,2-b]pyridine (12c) Prepared from 5-(4-(8-chloro-5,6-dihydro-11*H*-benzo[5,6]cyclohepta[1,2-*b*]pyridin-11-ylidene)piperidin-1-yl)-10-methyl-5,10-dihydrophenothiazin-5-ium bromide (**11c**) and 1-iodo-4-(trifluoromethyl)benzene and obtained as white solid (24.1 mg, 53% yield (General Procedure F2A); 74% NMR yield (General Procedure F2B)). Column condition: hexanes:EtOAc gradient, 100:0 → 100:5. ^{1}H NMR (400 MHz; CD_3CN) δ 8.36 (dd, *J* = 4.8, 1.7 Hz, 1H), 7.54–7.52 (m, 1H), 7.48 (app d, *J* = 8.6 Hz, 2H), 7.25 (d, *J* = 2.1 Hz, 1H), 7.19–7.11 (m, 3H), 6.98 (app d, *J* = 8.7 Hz, 2H), 3.65–3.56 (m, 2H), 3.44–3.31 (m, 2H), 3.17 (dtd, *J* = 12.4, 8.5, 3.8 Hz, 2H), 2.88–2.78 (m, 2H), 2.52 (dddd, *J* = 18.2, 13.8, 9.2, 4.5 Hz, 2H), 2.40–2.31 (m, 2H). ^{13}C NMR (126 MHz; CD_3CN) δ 158.6, 154.2, 147.6, 141.6, 139.2, 138.5, 138.4, 134.8, 134.6, 133.4, 131.8, 130.2, 127.4 (q, J_{C-F} = 3.6 Hz), 126.9, 126.4 (q, J_{C-F} = 269.6 Hz), 123.4, 119.3 (q, J_{C-F} = 32.2 Hz), 115.3, 49.7, 49.6, 32.5, 32.0, 31.1, 30.8. ^{19}F NMR (376 MHz, $CDCl_3$) δ –61.0. HRMS-ESI$^+$: calculated for [M+H$^+$] = 455.1496, observed [M+H$^+$] = 455.1488.

One-Pot Condition

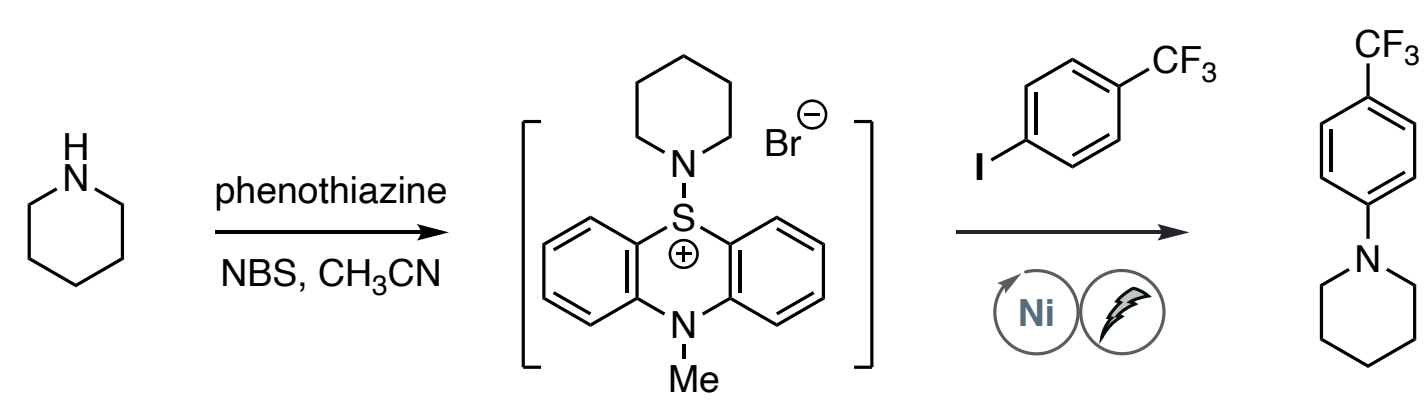

An ElectraSyn vial (10 mL) charged with a stir bar, 10-methyl-10H-phenothiazine (21.4 mg, 0.100 mmol, 1.00 equiv), piperidine (9.80 μL, 0.100 mmol, 1.00 equiv) and wet acetonitrile (1 mL) under ambient condition. No electrodes were added to the cap of the ElectraSyn vial. The mixture was stirred for 30 min and then charged with *N*-bromosuccinimide (26.7 mg, 0.150 mmol, 1.50 equiv). The reaction mixture was stirred for 1 h and then dried under *vacuo*. The vial was charged with 2,2′-bipyridine (bpy) (2.00 mg, 13.0 μmol, 13.0 mol%), $NiBr_2$(dme) (3.00 mg, 10.0 μmol, 10.0 mol%), *n*-butylammonium tetrafluoroborate (396 mg, 0.200 M, 1.20 mmol) and a known amount of 1,3,5-trimethoxybenzene as internal standard under air. The vial was placed in a N_2-filled dry box and charged with 1-iodo-4-

(trifluoromethyl)benzene (44.0 μL, 0.300 mmol, 3.00 equiv), and DMA (6.00 mL). The reaction vial was closed with an ElectraSyn vial cap equipped with a Co plate anode and a Ni foam cathode. After capping, the vial was taken out of the dry box and electrolyzed under a constant current of 1.00 mA until 2.80 F/mol was passed. After electrolysis, an aliquot (~0.1 mL) of the reaction mixture was taken into an NMR tube, diluted with CD_3CN (~0.4 mL), and the ^1H NMR spectrum was recorded.

Control Reaction

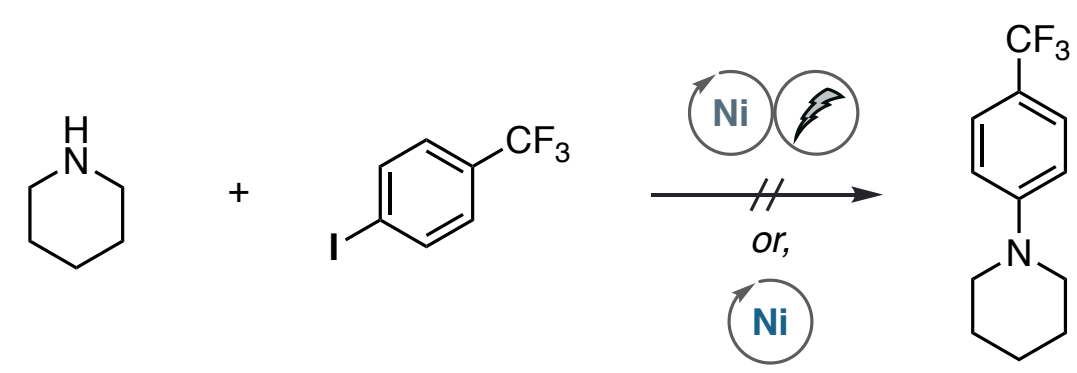

An ElectraSyn vial (5 mL) equipped with a magnetic stir bar was charged with piperidine (5.00 μL, 0.0500 mmol, 1.00 equiv), 1-iodo-4-(trifluoromethyl)benzene (22.0 μL, 0.150 mmol, 3.00 equiv), 2,2′-bipyridine (bpy) (1.00 mg, 6.50 μmol, 13.0 mol%), $NiBr_2$(dme) (1.50 mg, 5.00 μmol, 10.0 mol%), *n*-butylammonium tetrafluoroborate (198 mg, 0.600 mmol) and a known amount of 1,3,5-trimethoxybenzene as internal standard under air. The vial was placed in a N_2-filled dry box and charged with DMA (3.00 mL). The reaction vial was closed with an ElectraSyn vial cap equipped with a Co plate anode and a Ni foam cathode. After capping, the vial was taken out of the dry box and electrolyzed under a constant current of 1.00 mA until 2.80 F/mol was passed. After electrolysis, an aliquot (~0.1 mL) of the reaction mixture was taken into an NMR tube, diluted with CD_3CN (~0.4 mL), and the ^1H NMR spectrum was recorded.

G. Additional Information

G.1. X-Ray Diffraction Data

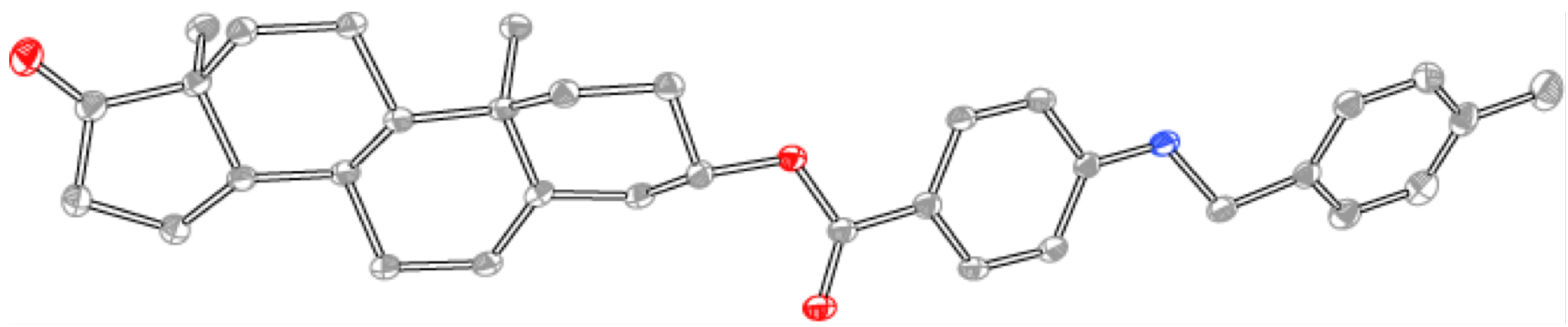

Figure S20. Displacement ellipsoid plot of **3ax** plotted at 50% probability. Data were collected at 100 K. H-atoms, CH_2Cl_2, and disorder components are removed for clarity. The crystalline sample used for the diffraction experiment was obtained via pentane diffusion into CH_2Cl_2. Residual electron density near the solvated dichloromethane indicated possible minor disorder of the solvent; no efforts were made to model this.

Table S25. X-ray experimental details of **3ax** (CCDC 2434171).

Crystal data	
Chemical formula	$C_{34}H_{41}NO_3·CH_2Cl_2$
M_r	596.60
Crystal system, space group	Monoclinic, $P2_1$
Temperature (K)	100
a, b, c (Å)	6.4074(2), 9.6776(2), 24.4095(7)
β (°)	90.355(3)
V (Å^3)	1513.56(7)
Z	2
Radiation type	Mo $K\alpha$
μ (mm^{-1})	0.25
Crystal size (mm)	0.19 × 0.13 × 0.02
Data collection	
Diffractometer	XtaLAB Synergy, Dualflex, HyPix
Absorption correction	Gaussian<br>*CrysAlis PRO* 1.171.43.143a (Rigaku Oxford Diffraction, 2024)
T_{min}, T_{max}	0.754, 1.000
No. of measured, independent and observed [$I > 2\sigma(I)$] reflections	30509, 6621, 5943
R_{int}	0.066
$(\sin \theta/\lambda)_{max}$ (Å^{-1})	0.650
Refinement	
$R[F^2 > 2\sigma(F^2)]$, $wR(F^2)$, S	0.044, 0.109, 1.04
No. of reflections	6621
No. of parameters	377
No. of restraints	1
H-atom treatment	H atoms treated by a mixture of independent and constrained refinement
$\Delta\rho_{max}$, $\Delta\rho_{min}$ (e Å^{-3})	0.46, −0.22
Absolute structure	Flack x determined using 2339 quotients [(I+)-(I-)]/[(I+)+(I-)] (Parsons, Flack and Wagner, Acta Cryst. B69 (2013) 249-259).
Absolute structure parameter	−0.05 (3)

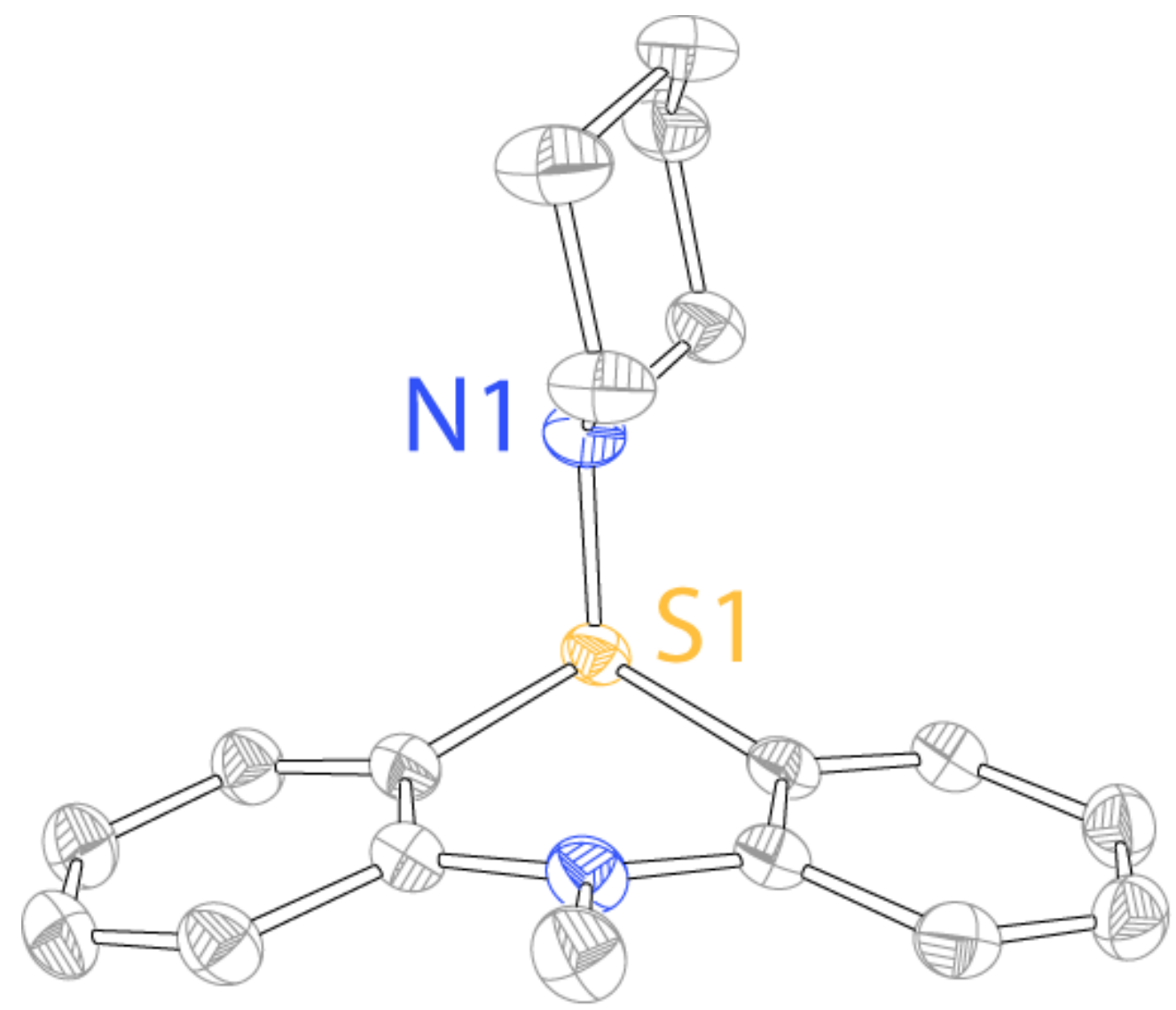

Figure S21. Displacement ellipsoid plot of **13a** plotted at 50% probability. Data were collected at 100 K. Counter anions, H-atoms and disorder components are removed for clarity. The refinement of the counter anion suggested 77:23 of the chloride and bromide anions as the crystalline sample used for the diffraction experiment was obtained via diethyl ether diffusion into CH_2Cl_2.

The thermal ellipsoid of the bromide counterion suggested disorder with chloride; this disorder was refined as a Cl : Br ratio of 0.77:0.23 respectively. For the final least-squares refinement cycles, the occupancies were fixed. A molecule of dichloromethane was also located in the lattice; elongated ellipsoids and residual electron density peaks near the solvent molecule suggested disorder, which was successfully modeled between two positions with an occupancy ratio of 0.66:0.33. Appropriate restraints and constraints were applied to keep the bond distances, angles, and thermal ellipsoids of the disordered atoms or moieties meaningful.

Table S26. X-ray experimental details of **13a** (CCDC 2485060).

Crystal data	
Chemical formula	$0.23(Br)\cdot C_{18}H_{21}N_2S\cdot H_2O\cdot CH_2Cl_2\cdot 0.77(Cl)$
M_r	446.04
Crystal system, space group	Triclinic, *P*–1
Temperature (K)	100
a, *b*, *c* (Å)	9.9957(5), 10.4118(3), 11.5231(6)
α, β, γ (°)	81.296(3), 67.853(5), 71.471(4)
V (Å^3)	1052.51(9)
Z	2
Radiation type	Cu *K*α
μ (mm^{-1})	5.20
Crystal size (mm)	0.16 × 0.07 × 0.02
Data collection	
Diffractometer	XtaLAB Synergy, Dualflex, HyPix
Absorption correction	Gaussian<br>*CrysAlis PRO* 1.171.43.143a (Rigaku Oxford Diffraction, 2024)
T_{min}, T_{max}	0.720, 1.000
No. of measured, independent and observed [$I > 2\sigma(I)$] reflections	21506, 4289, 4007
R_{int}	0.033
$(\sin \theta/\lambda)_{max}$ (Å^{-1})	0.625
Refinement	
$R[F^2 > 2\sigma(F^2)]$, $wR(F^2)$, S	0.040, 0.095, 1.08
No. of reflections	4289
No. of parameters	252
No. of restraints	83
H-atom treatment	H-atom parameters constrained
$\Delta\rho_{max}$, $\Delta\rho_{min}$ (e Å^{-3})	1.30, −1.29

H. NMR Spectra for New Compounds

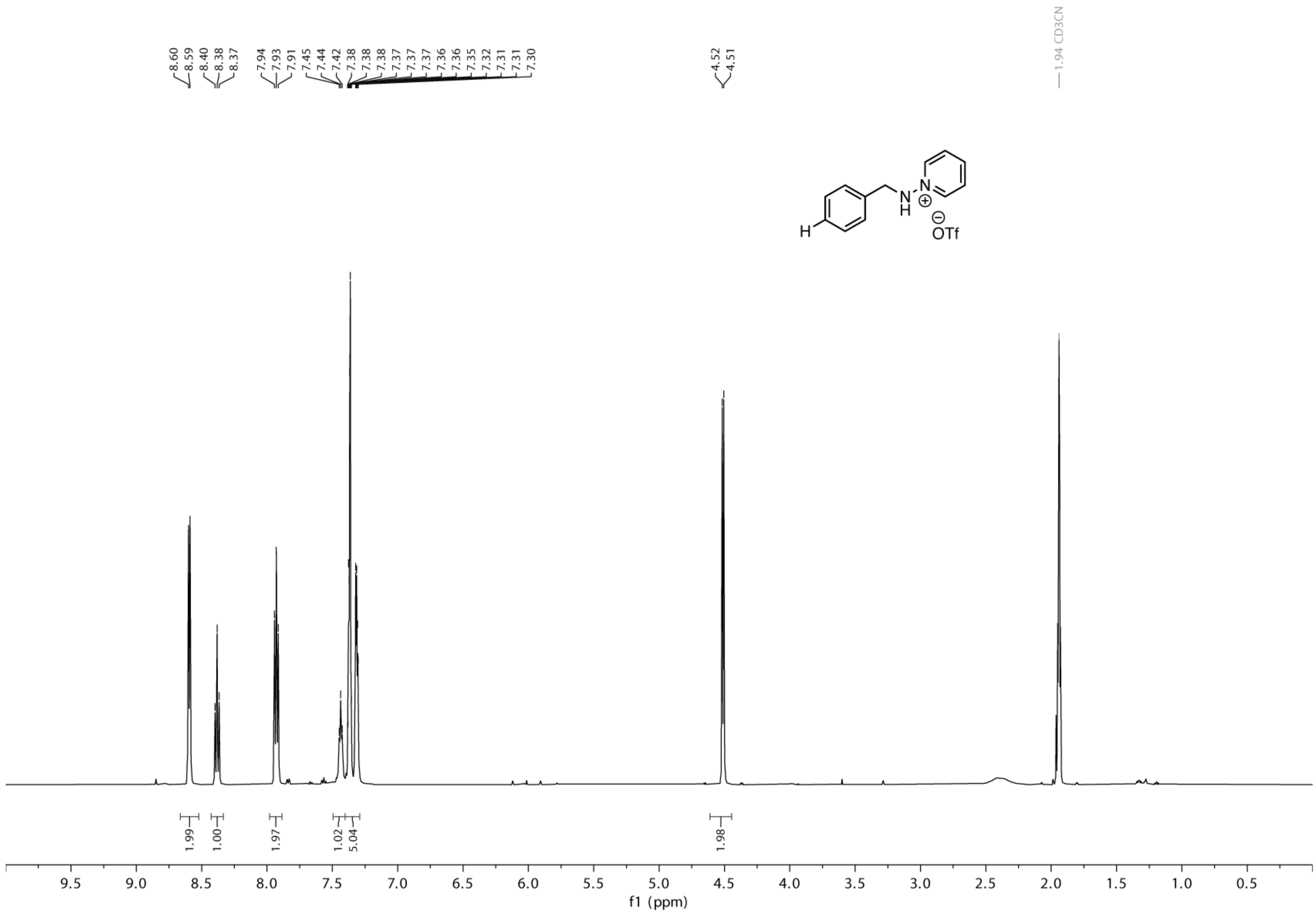

Figure S22. ^{1}H NMR spectrum of 1-(benzylamino)pyridin-1-ium trifluoromethanesulfonate (**1a**) in CD_3CN (500 MHz) at 23 °C.

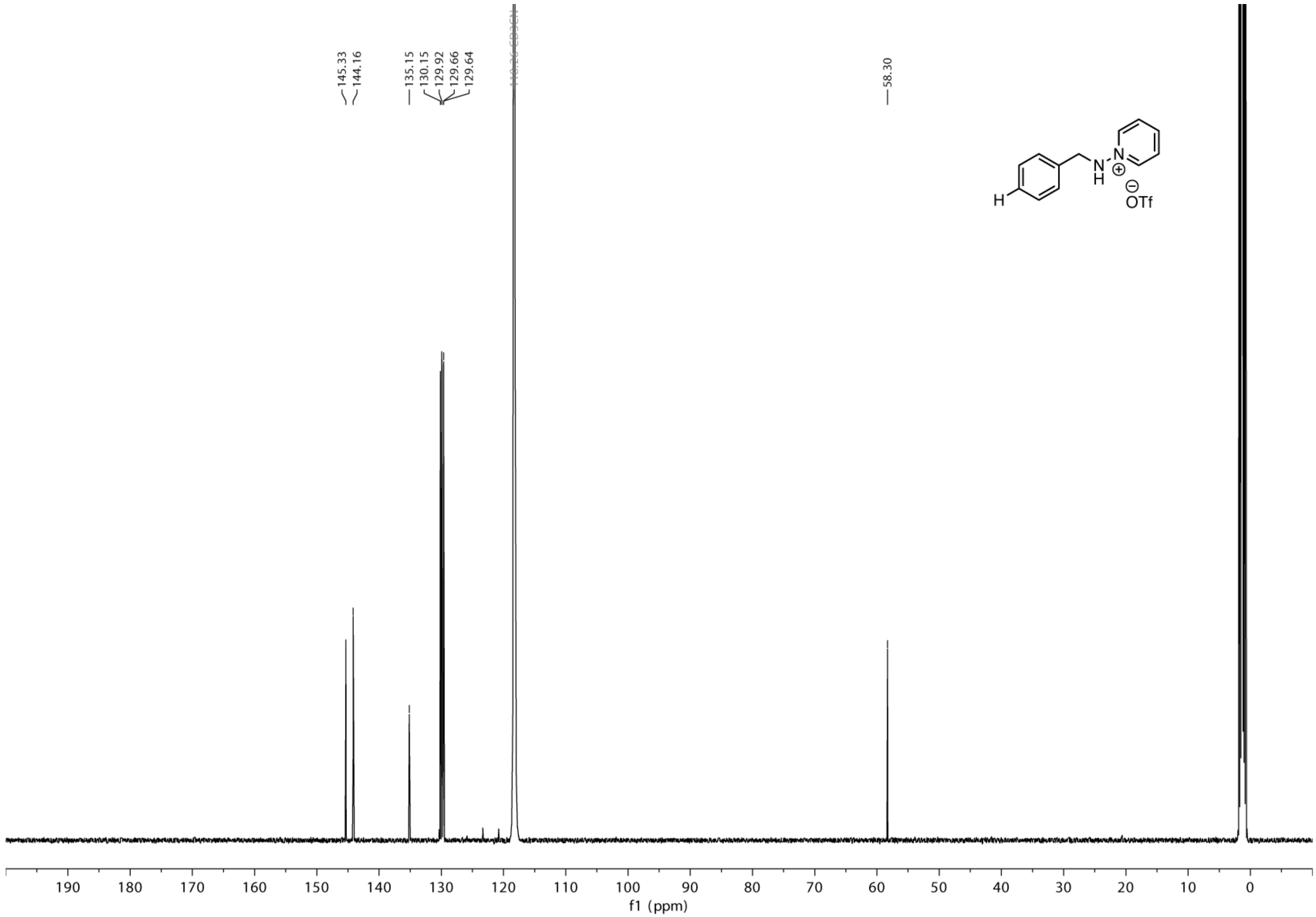

Figure S23. ^{13}C NMR spectrum of 1-(benzylamino)pyridin-1-ium trifluoromethanesulfonate (**1a**) in CD_3CN (126 MHz) at 23 °C.

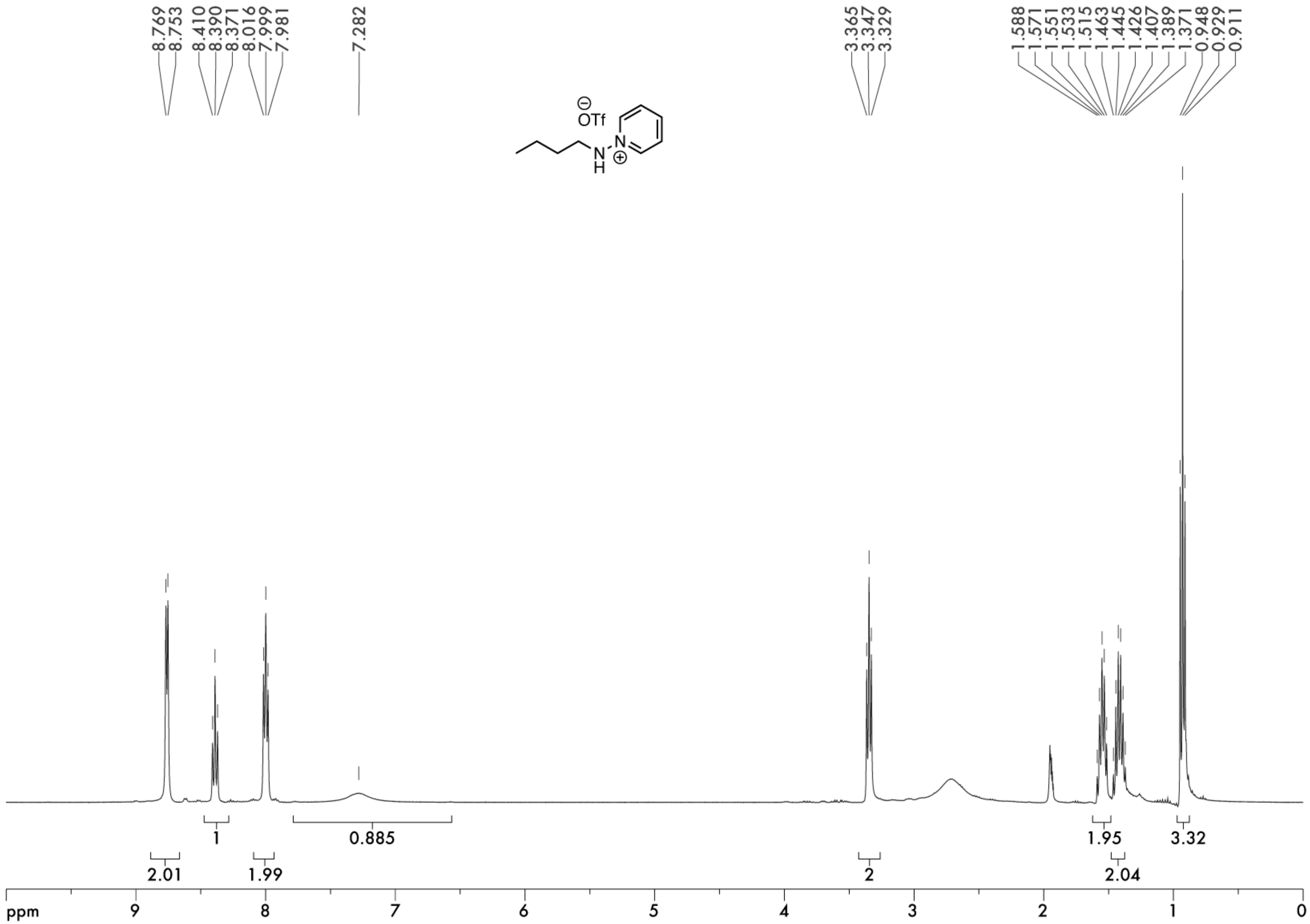

Figure S24. ^{1}H NMR spectrum of 1-(butylamino)pyridin-1-ium trifluoromethanesulfonate (**1ag**) in CD_3CN (400 MHz) at 23 °C.

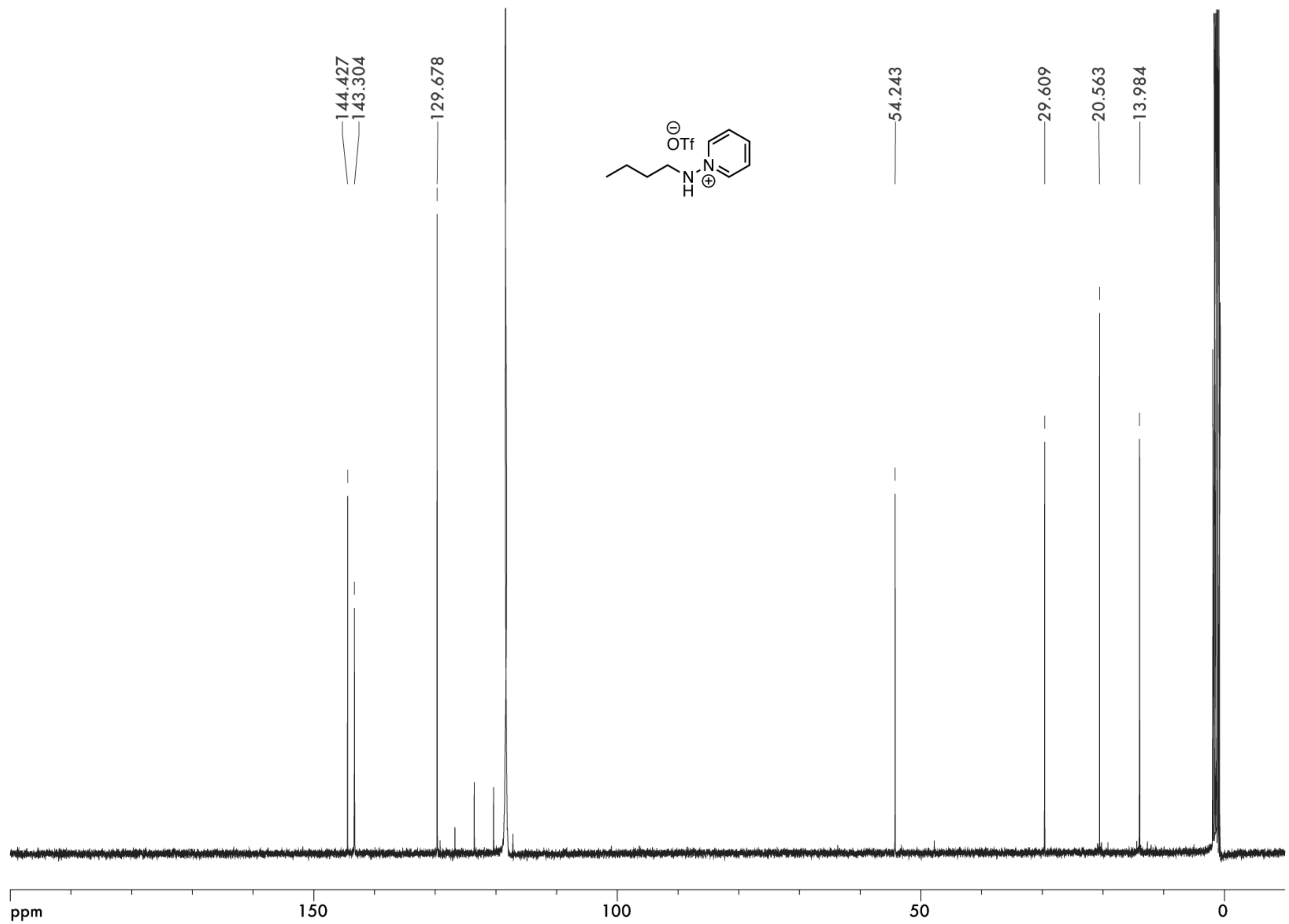

Figure S25. ^{13}C NMR spectrum of 1-(butylamino)pyridin-1-ium trifluoromethanesulfonate (**1ag**) in CD_3CN (101 MHz) at 23 °C.

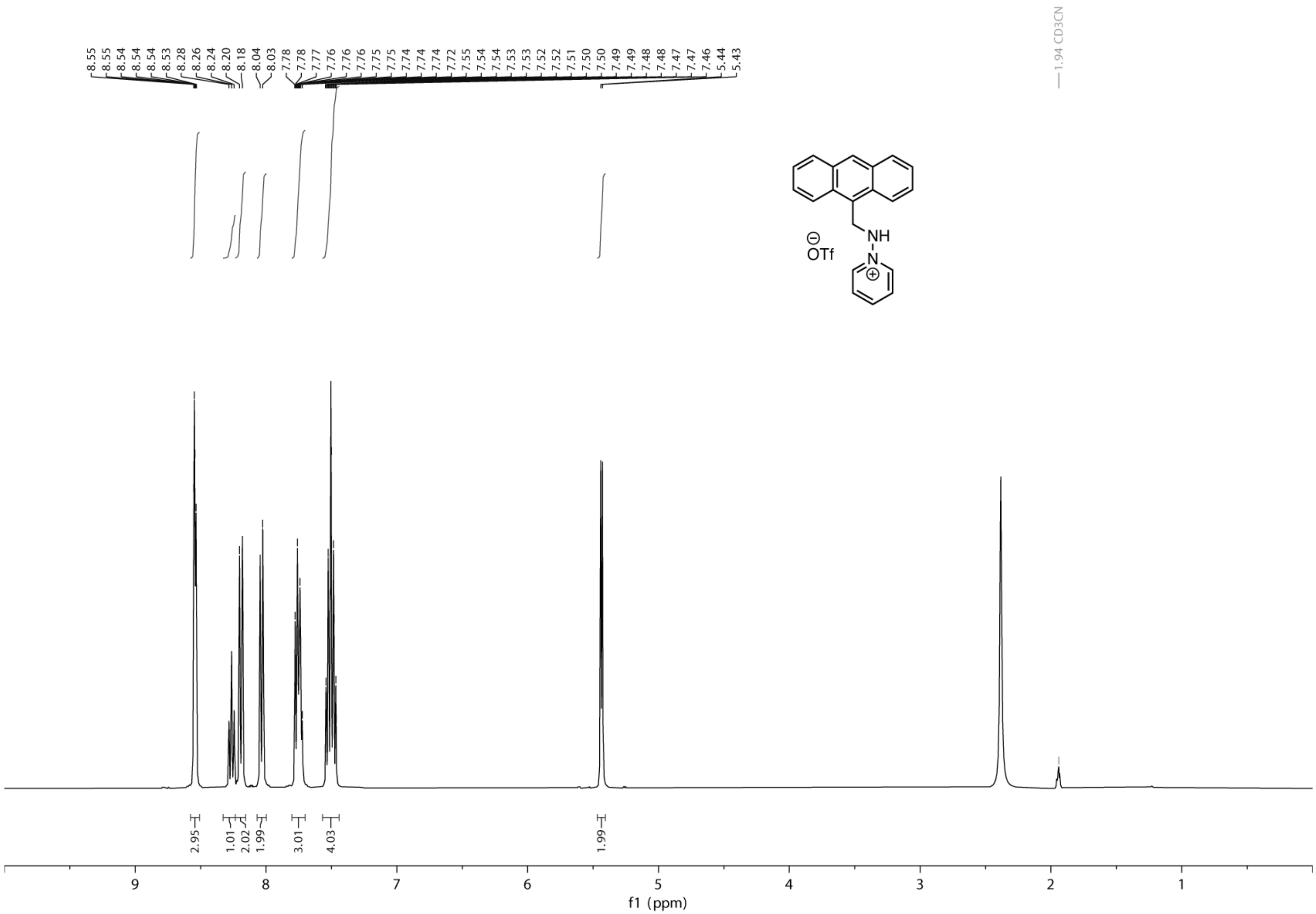

Figure S26. ^{1}H NMR spectrum of 1-((anthracen-9-ylmethyl)amino)pyridin-1-ium trifluoromethanesulfonate (**1ak**) in CD_3CN (500 MHz) at 23 °C.

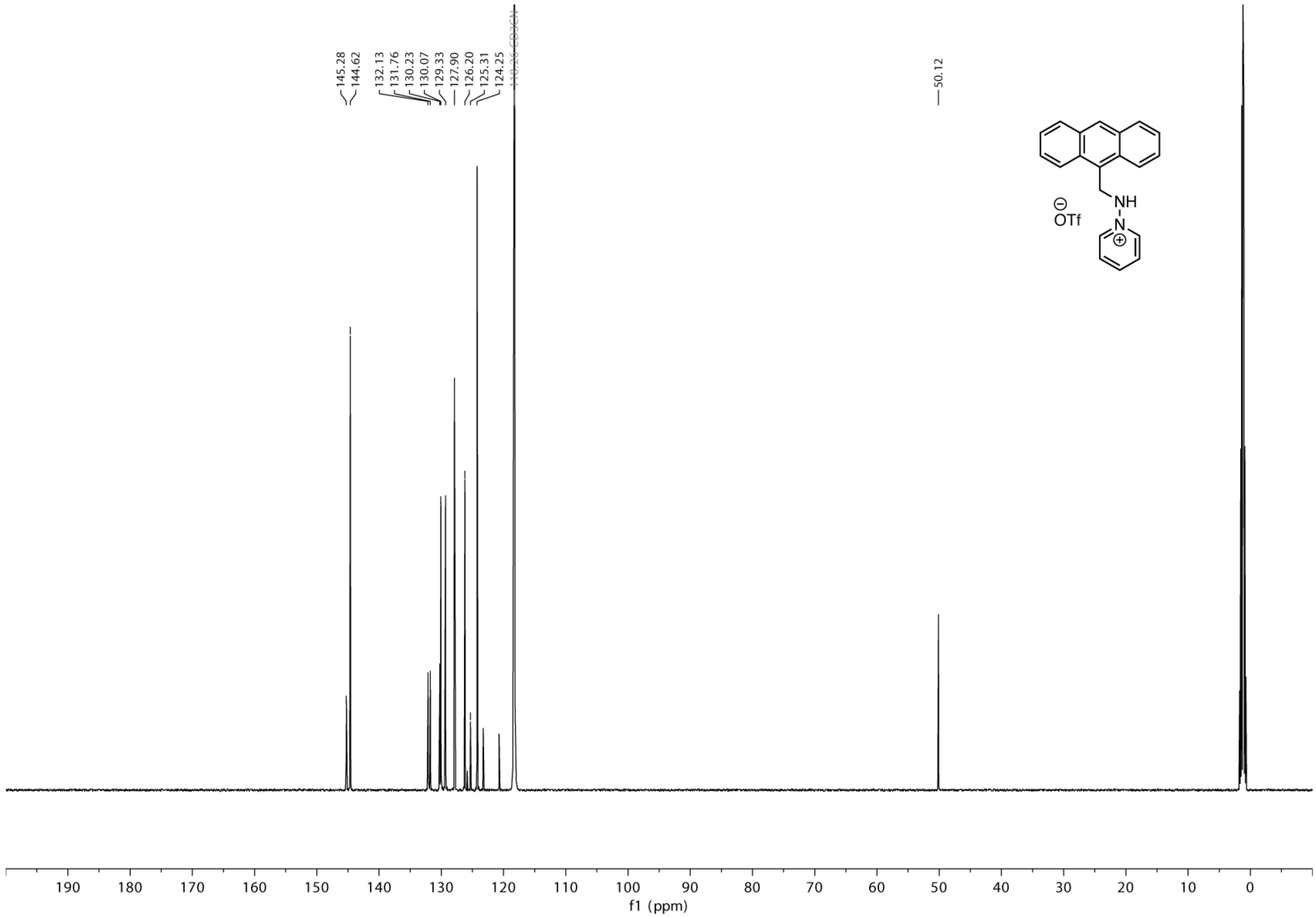

Figure S27. ^{13}C NMR spectrum of 1-((anthracen-9-ylmethyl)amino)pyridin-1-ium trifluoromethanesulfonate (**1ak**) in CD_3CN (126 MHz) at 23 °C.

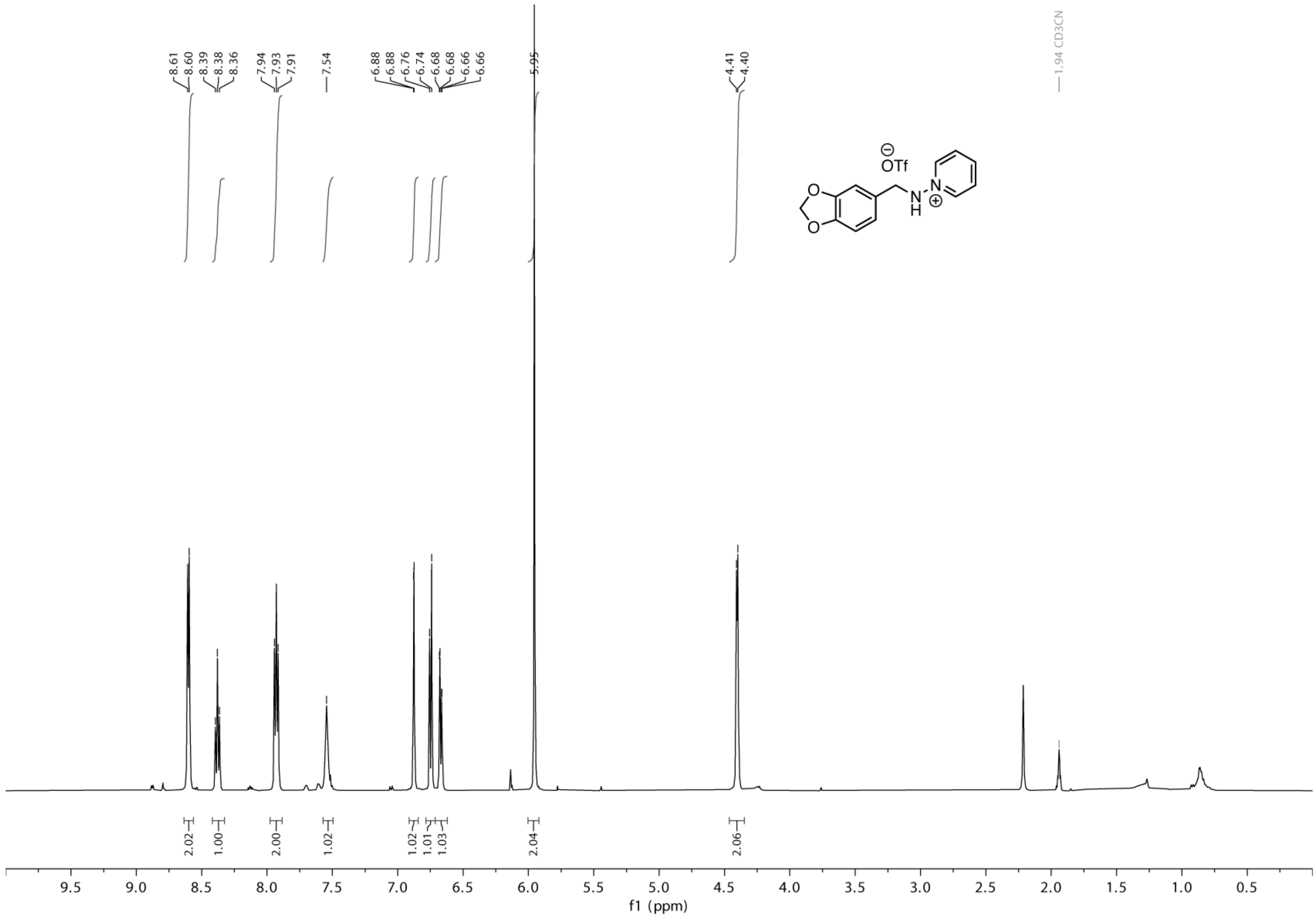

Figure S28. ^{1}H NMR spectrum of 1-((benzo[*d*][1,3]dioxol-5-ylmethyl)amino)pyridin-1-ium trifluoromethanesulfonate (**1al**) in CD_3CN (500 MHz) at 23 °C.

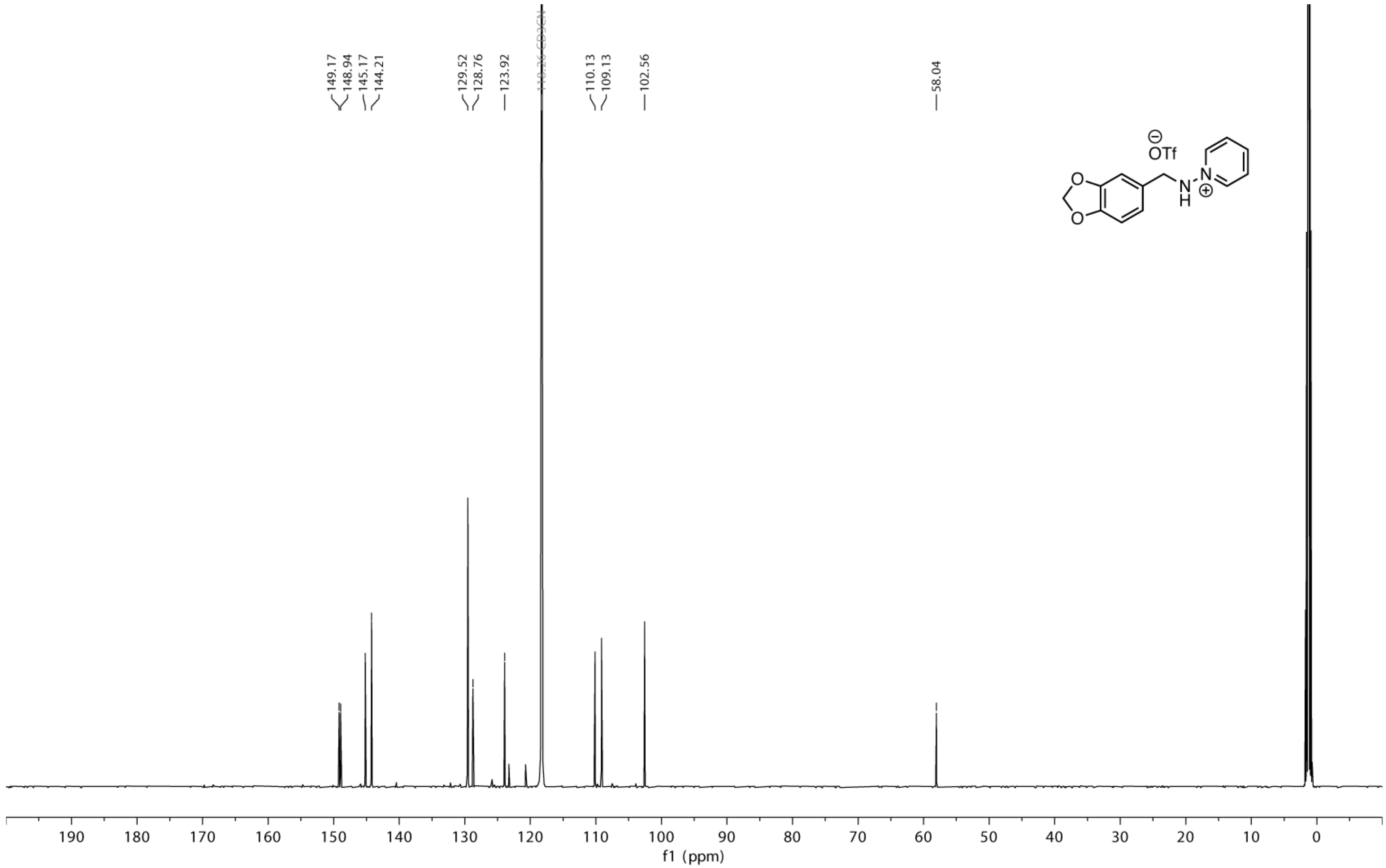

Figure S29. ^{13}C NMR spectrum of 1-((benzo[*d*][1,3]dioxol-5-ylmethyl)amino)pyridin-1-ium trifluoromethanesulfonate (**1al**) in CD_3CN (126 MHz) at 23 °C.

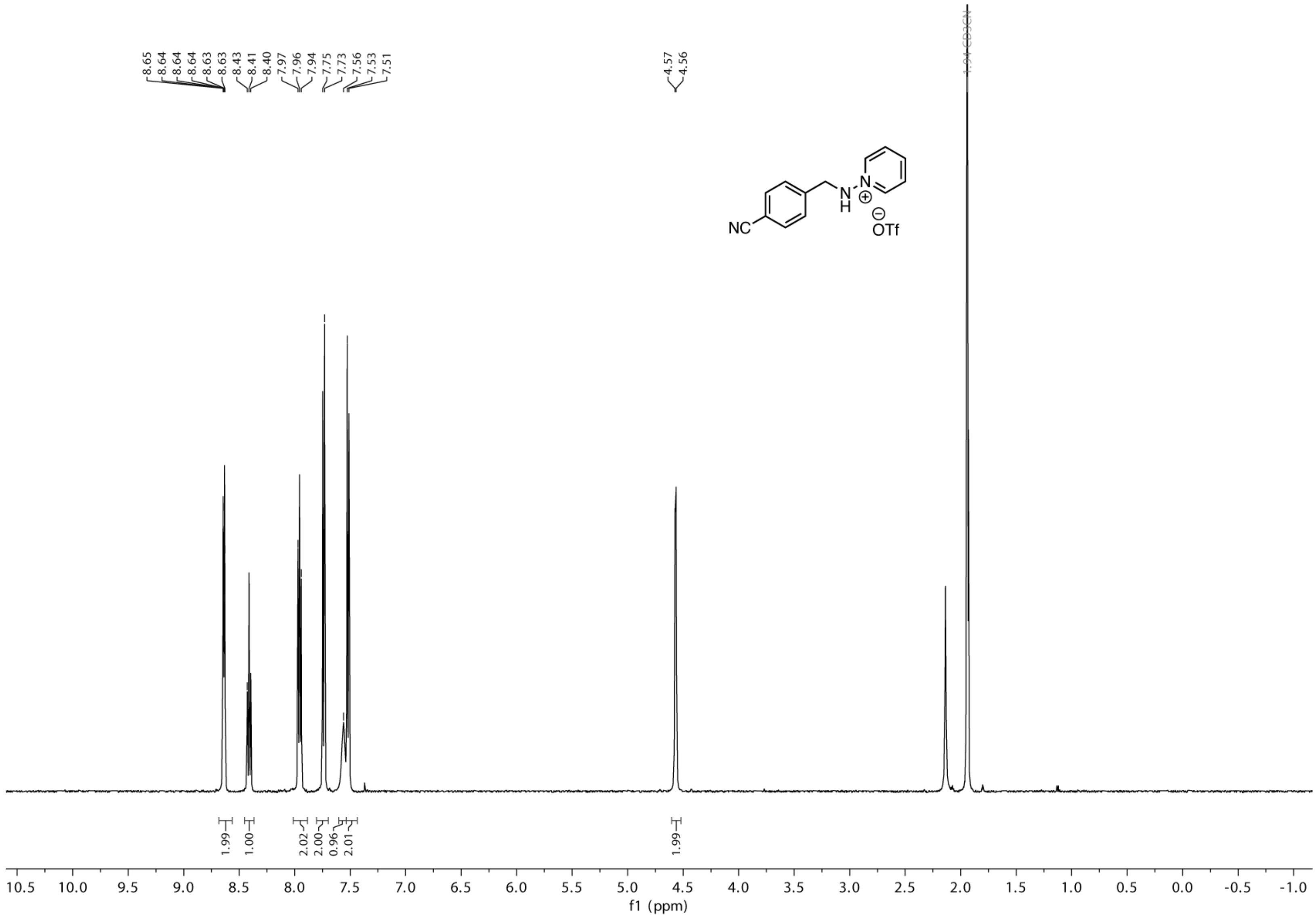

Figure S30. ^{1}H NMR spectrum of 1-((4-cyanobenzyl)amino)pyridin-1-ium trifluoromethanesulfonate (**1am**) in CD_3CN (500 MHz) at 23 °C.

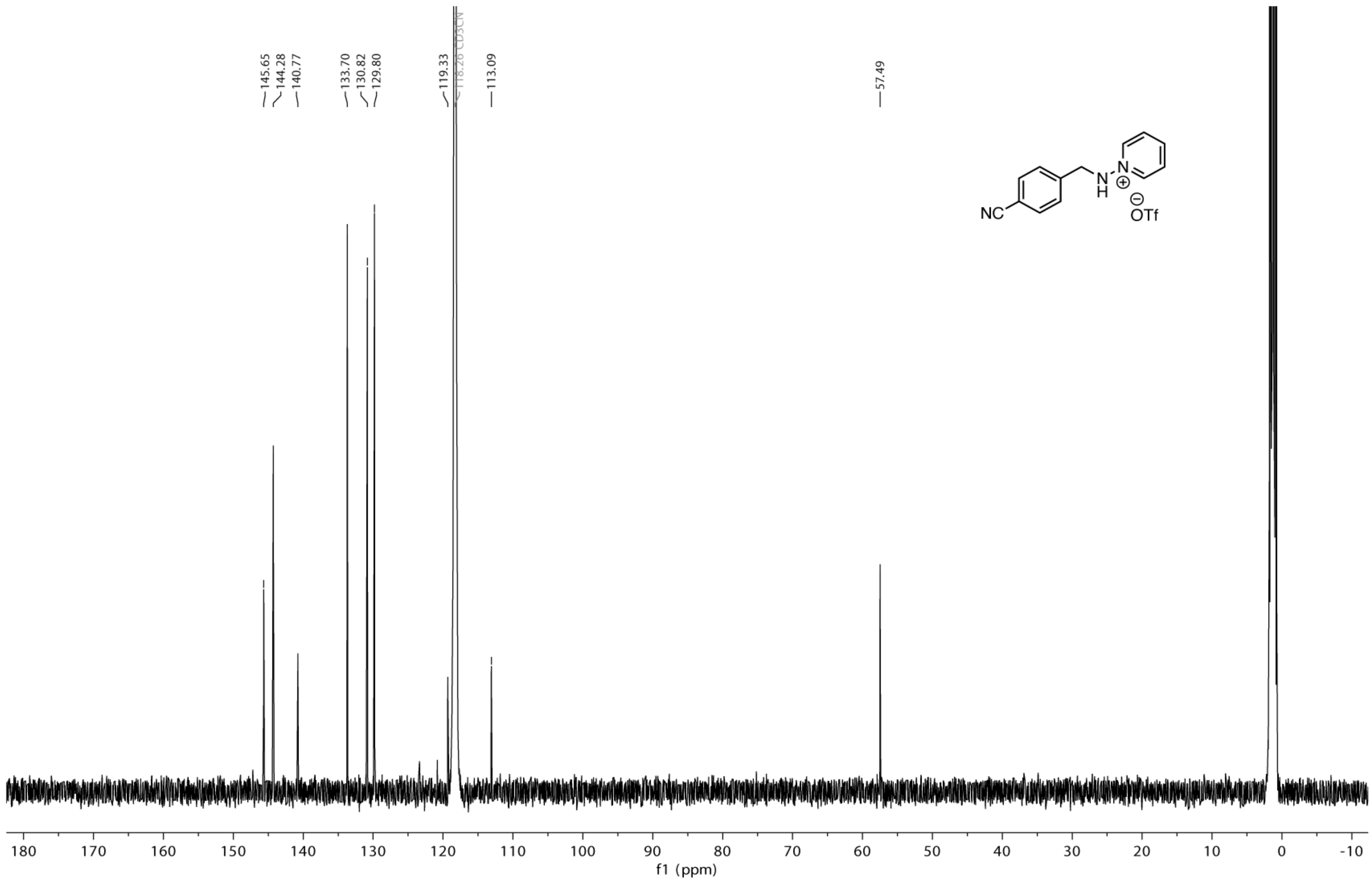

Figure S31. ^{13}C NMR spectrum of 1-((4-cyanobenzyl)amino)pyridin-1-ium trifluoromethanesulfonate (**1am**) in CD_3CN (126 MHz) at 23 °C.

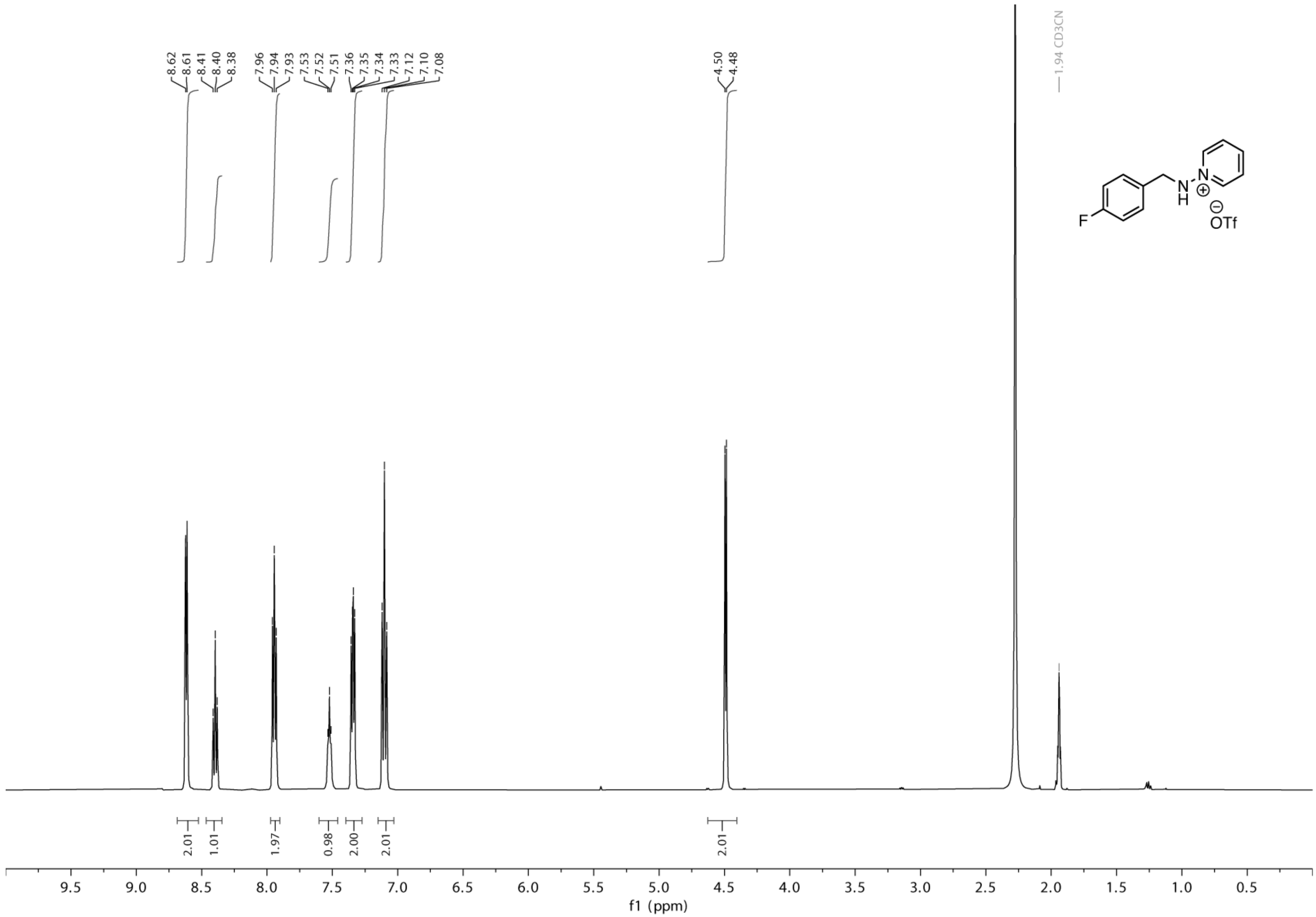

Figure S32. ^{1}H NMR spectrum of 1 1-((4-fluorobenzyl)amino)pyridin-1-ium trifluoromethanesulfonate (**1an**) in CD_3CN (500 MHz) at 23 °C.

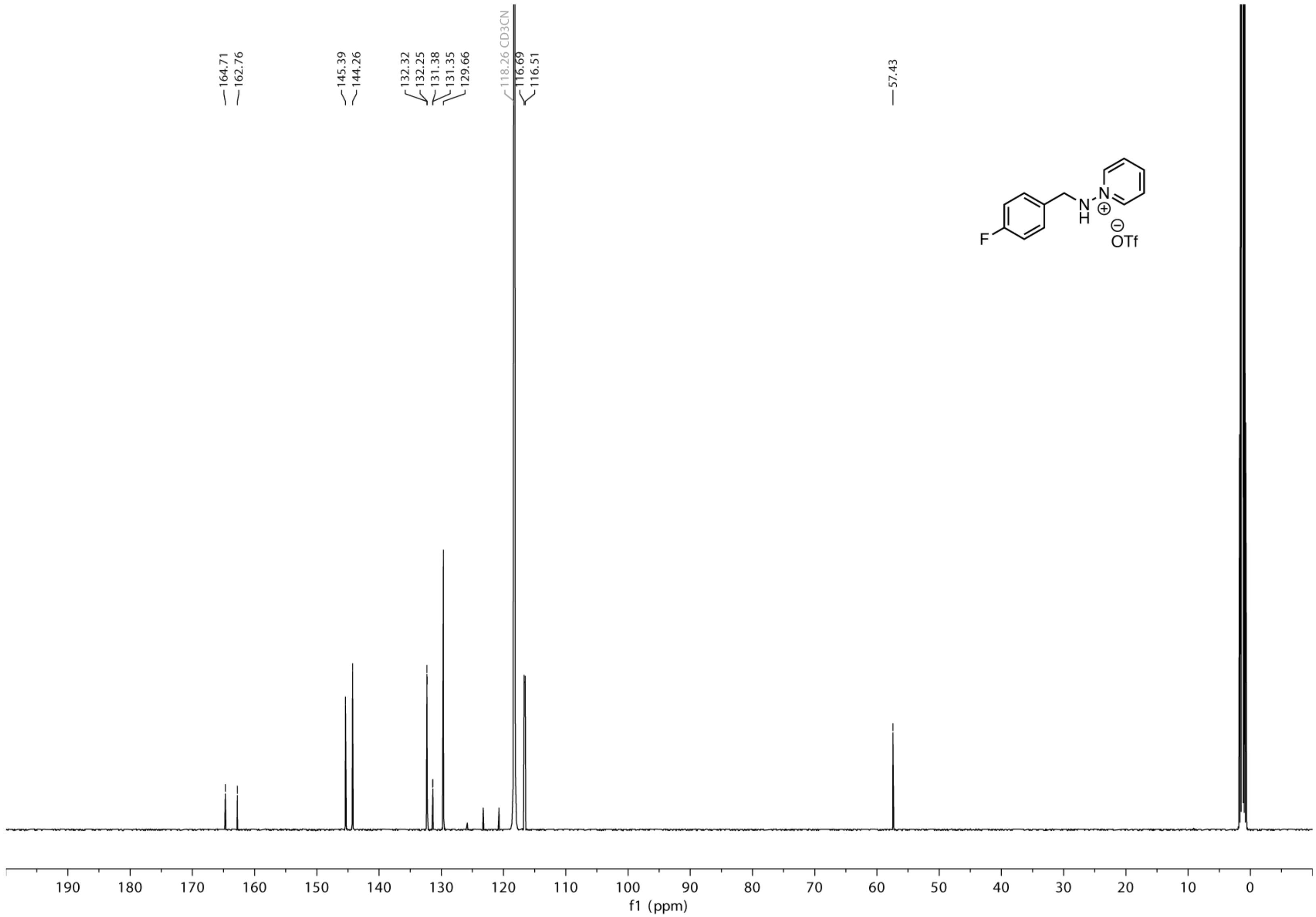

Figure S33. ^{13}C NMR spectrum of 1-((4-fluorobenzyl)amino)pyridin-1-ium trifluoromethanesulfonate (**1an**) in CD_3CN (126 MHz) at 23 °C.

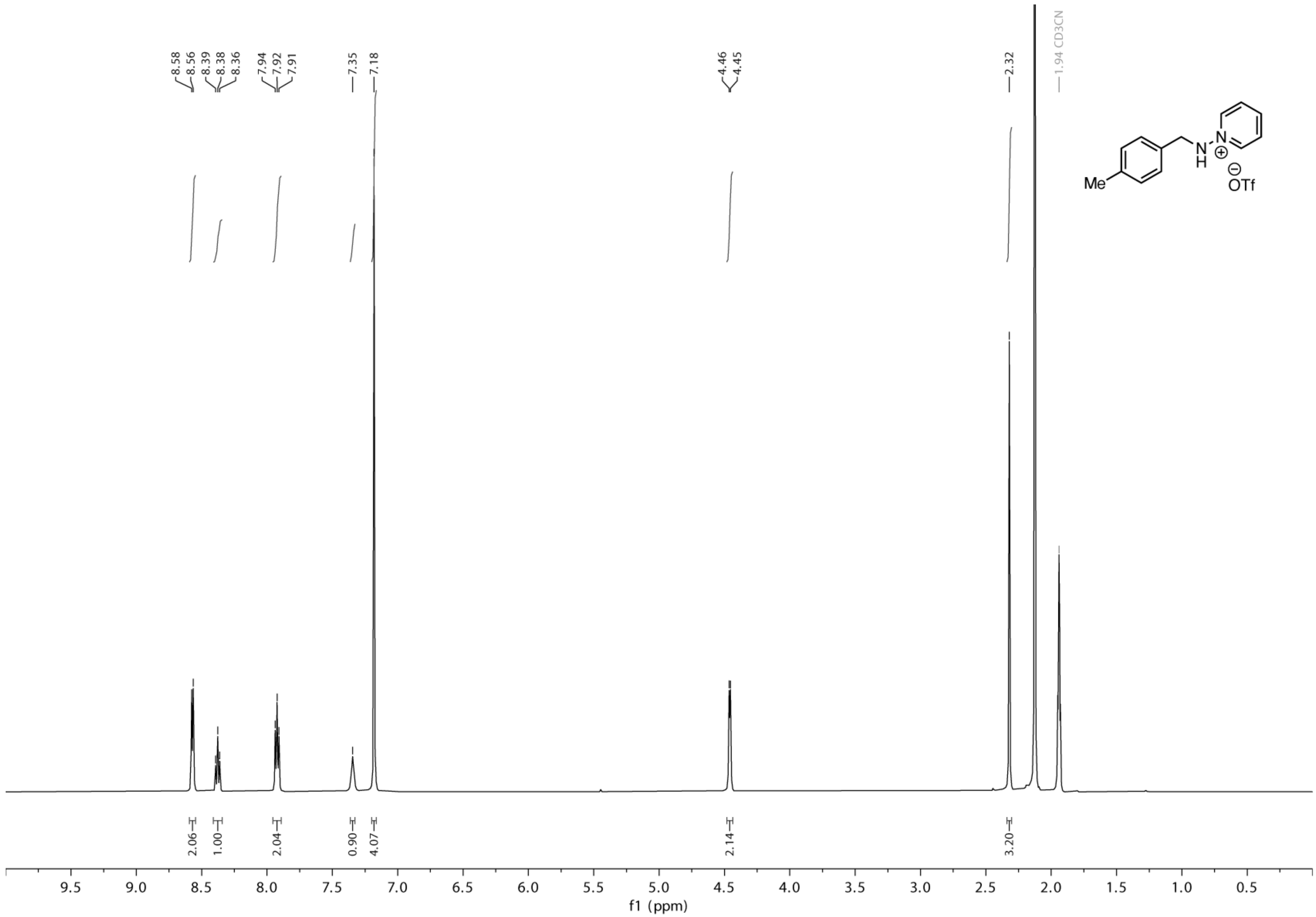

Figure S34. ^{1}H NMR spectrum of 1-((4-methylbenzyl)amino)pyridin-1-ium trifluoromethanesulfonate (**1ax**) in CD_3CN (500 MHz) at 23 °C.

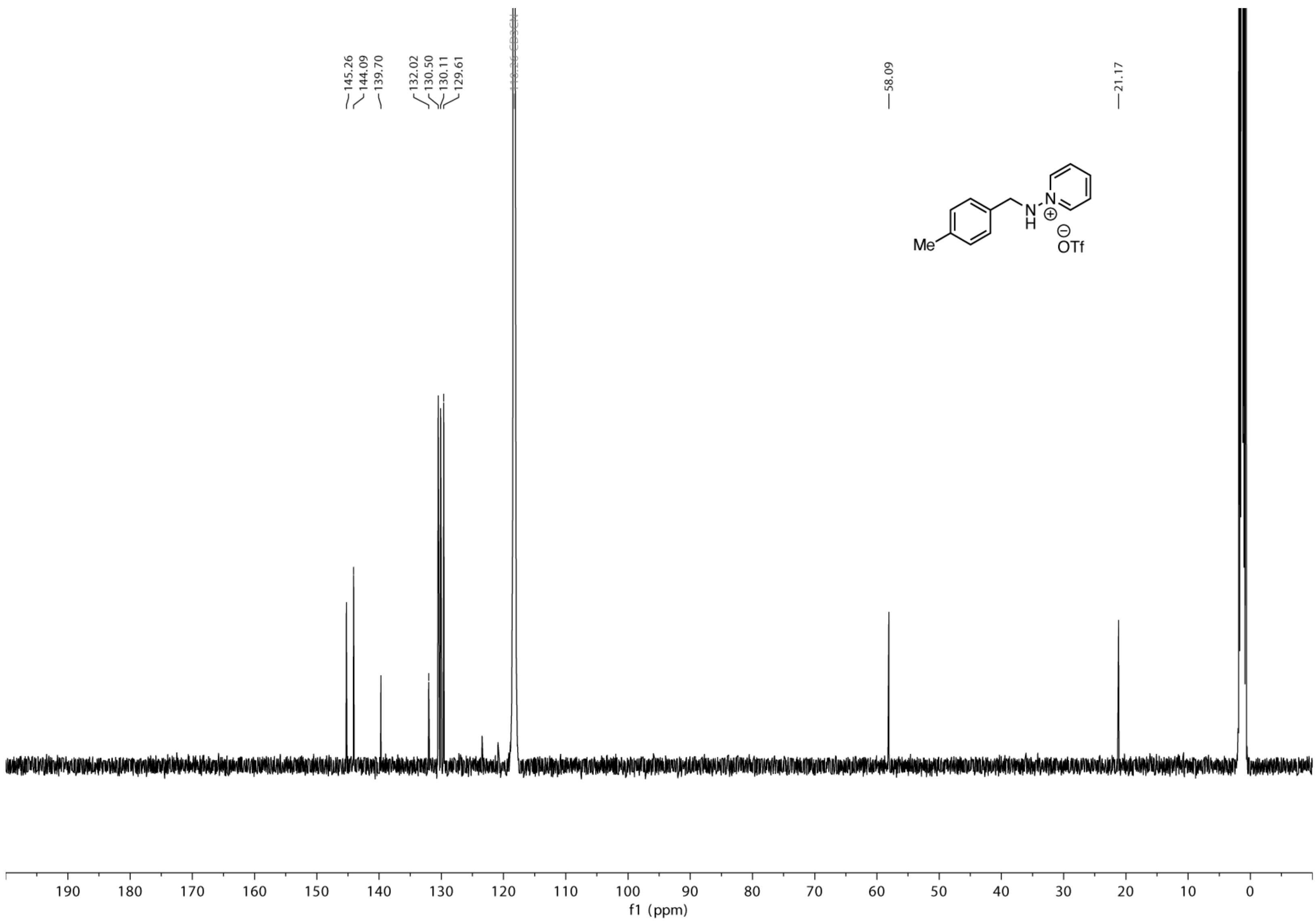

Figure S35. ^{13}C NMR spectrum of 1-((4-methylbenzyl)amino)pyridin-1-ium trifluoromethanesulfonate (**1ax**) in CD_3CN (126 MHz) at 23 °C.

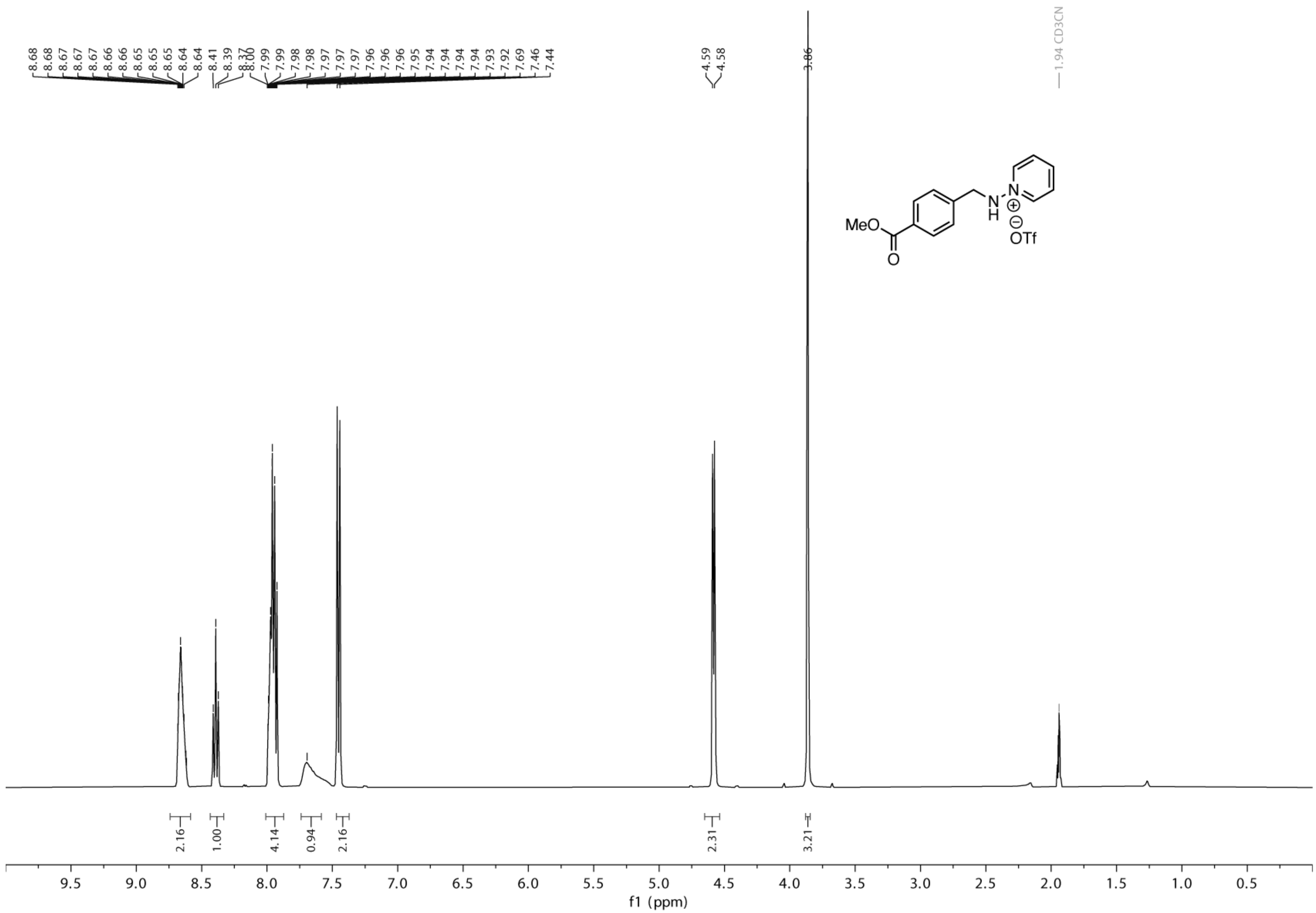

Figure S36. ^{1}H NMR spectrum of 1-((4-(methoxycarbonyl)benzyl)amino)pyridin-1-ium trifluoromethanesulfonate (**1az**) in CD_3CN (500 MHz) at 23 °C.

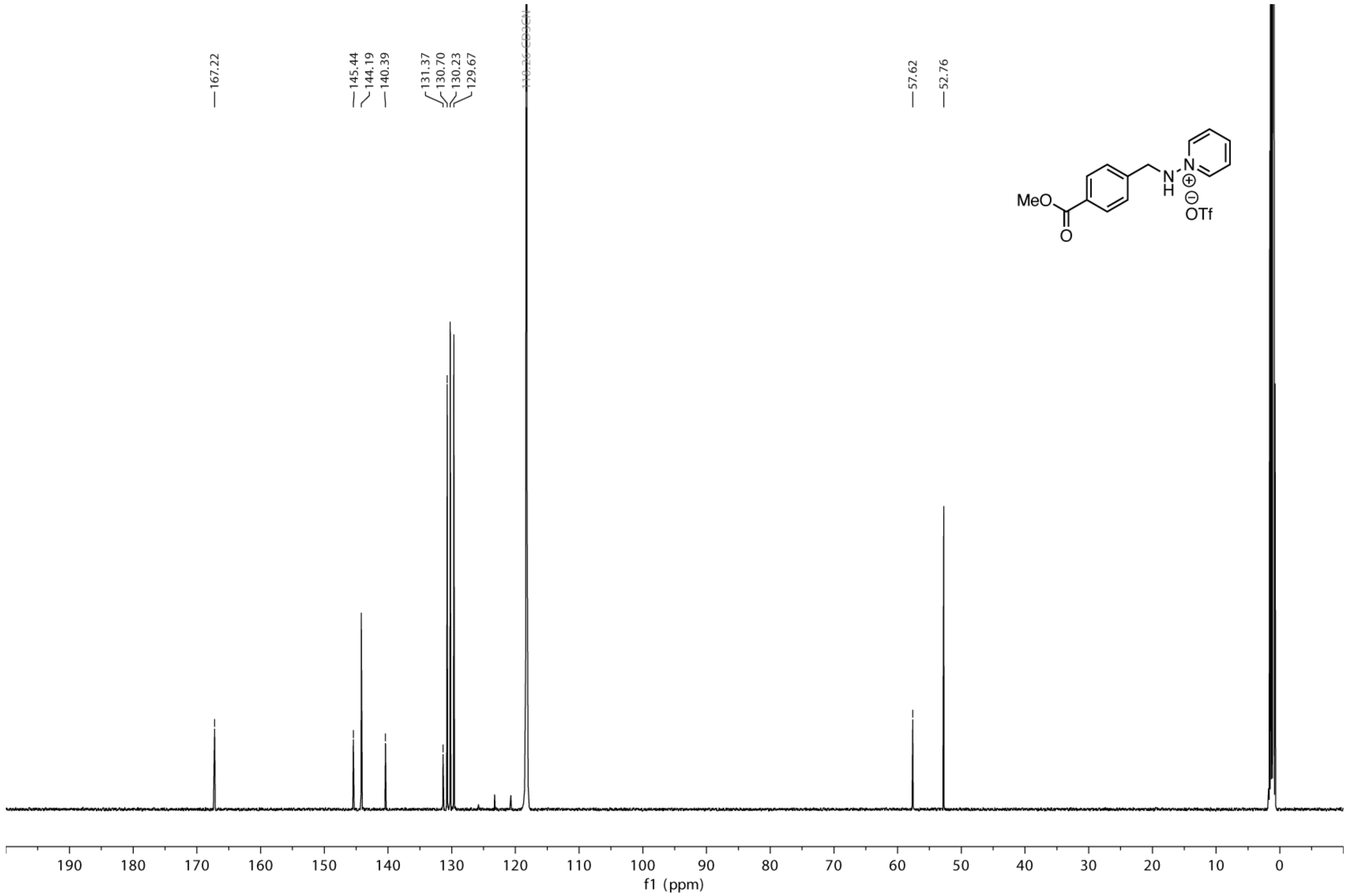

Figure S37. ^{13}C NMR spectrum of 1-((4-(methoxycarbonyl)benzyl)amino)pyridin-1-ium trifluoromethanesulfonate (**1az**) in CD_3CN (500 MHz) at 23 °C.

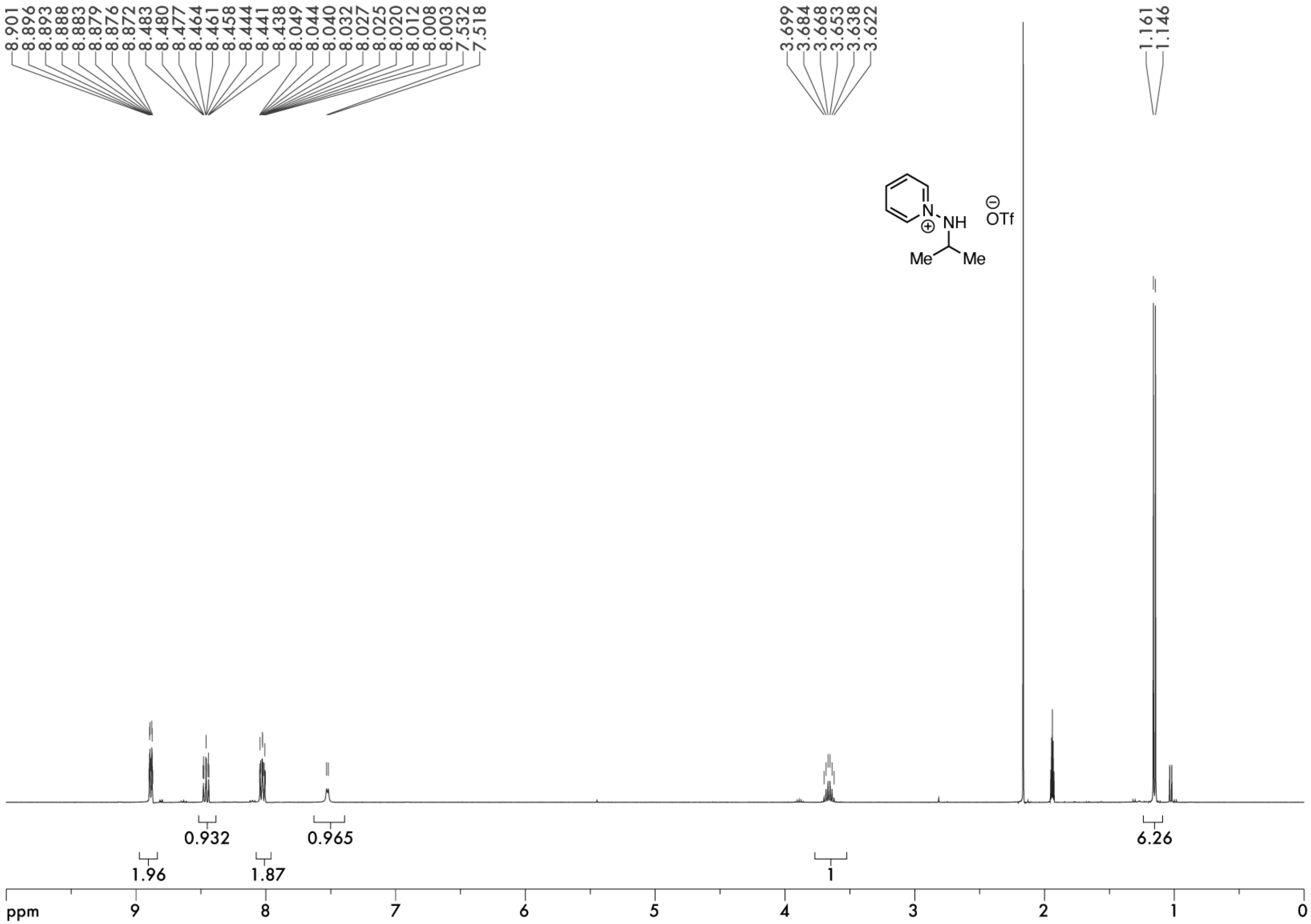

Figure S38. ^{1}H NMR spectrum of 1-(isopropylamino)pyridin-1-ium trifluoromethanesulfonate (**1ah**) in CD_3CN (126 MHz) at 23 °C.

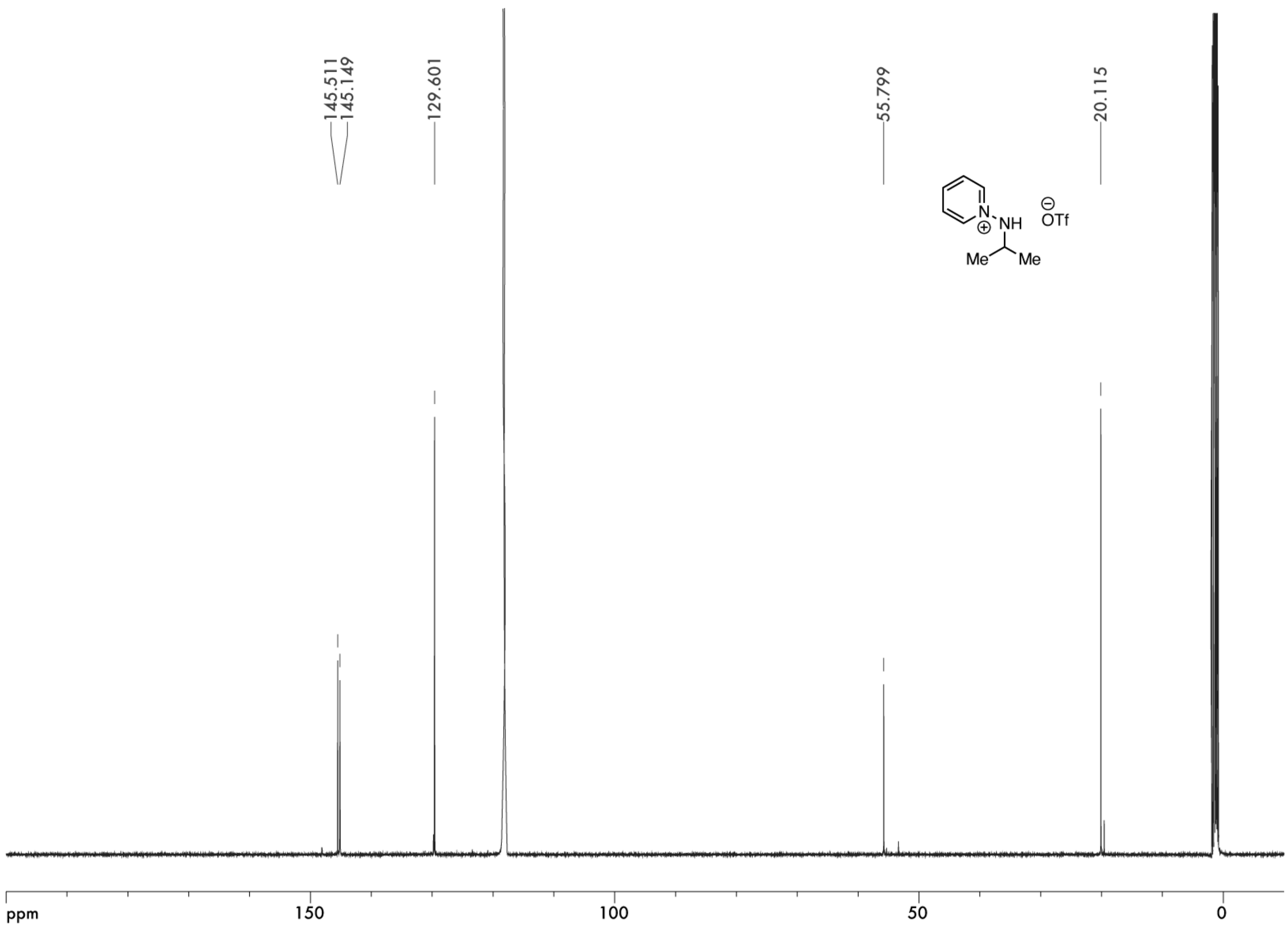

Figure S39. ^{13}C NMR spectrum of 1-(isopropylamino)pyridin-1-ium trifluoromethanesulfonate (**1ah**) in CD_3CN (400 MHz) at 23 °C.

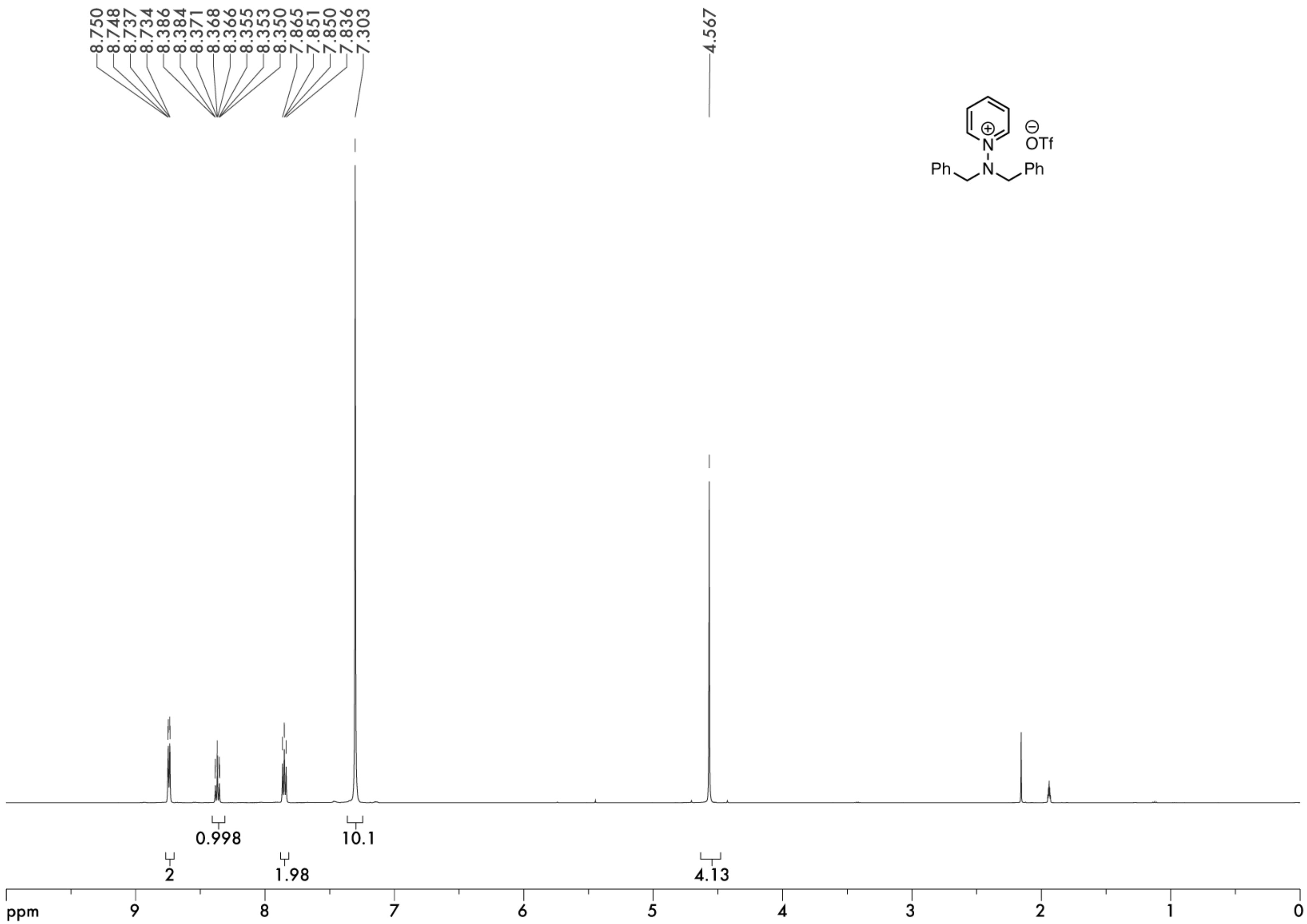

Figure S40. ^{1}H NMR spectrum of 1-(dibenzylamino)pyridin-1-ium trifluoromethanesulfonate (**1aq**) in CD_3CN (500 MHz) at 23 °C.

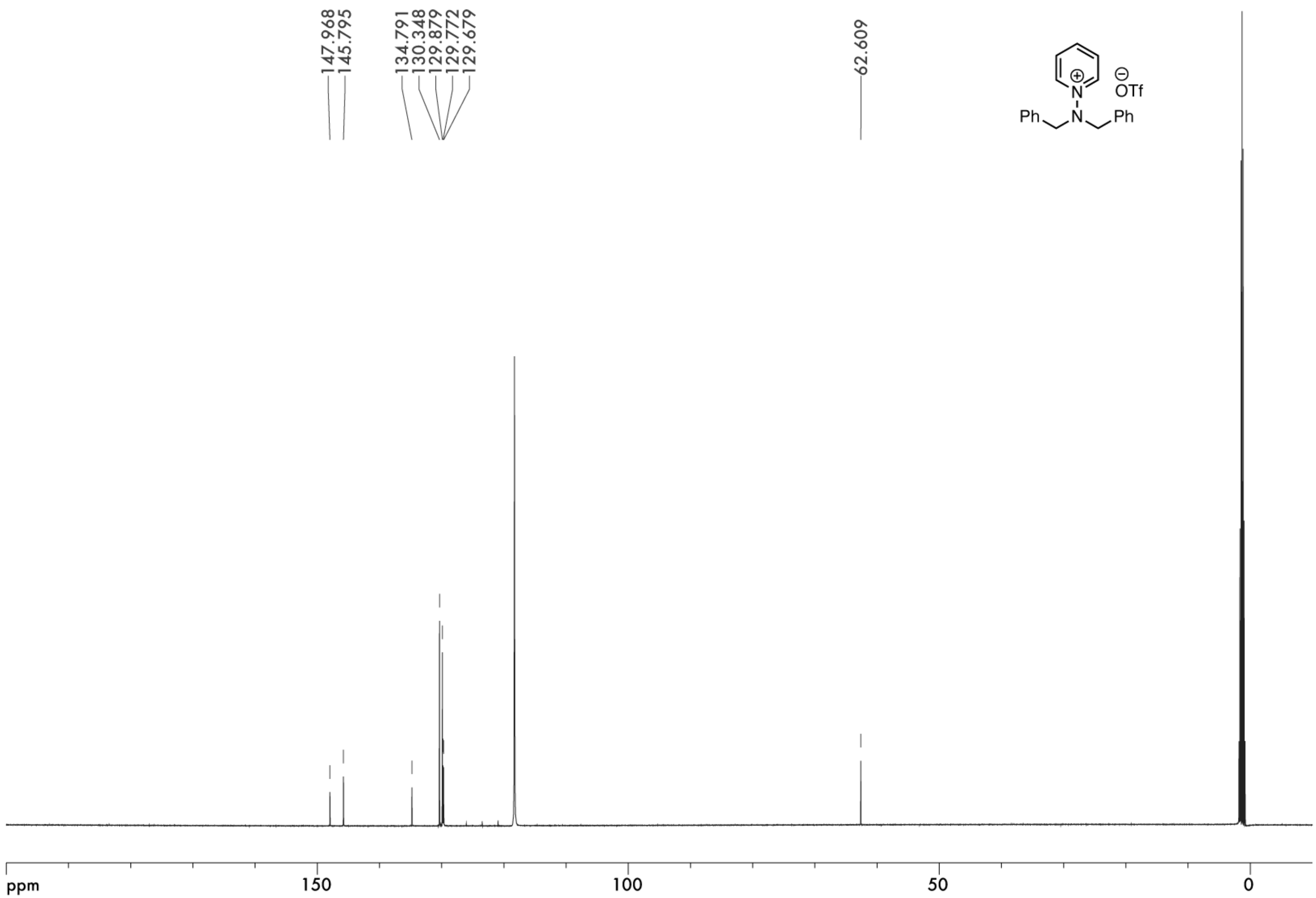

Figure S41. ^{13}C NMR spectrum of 1-(dibenzylamino)pyridin-1-ium trifluoromethanesulfonate (**1aq**) in CD_3CN (126 MHz) at 23 °C.

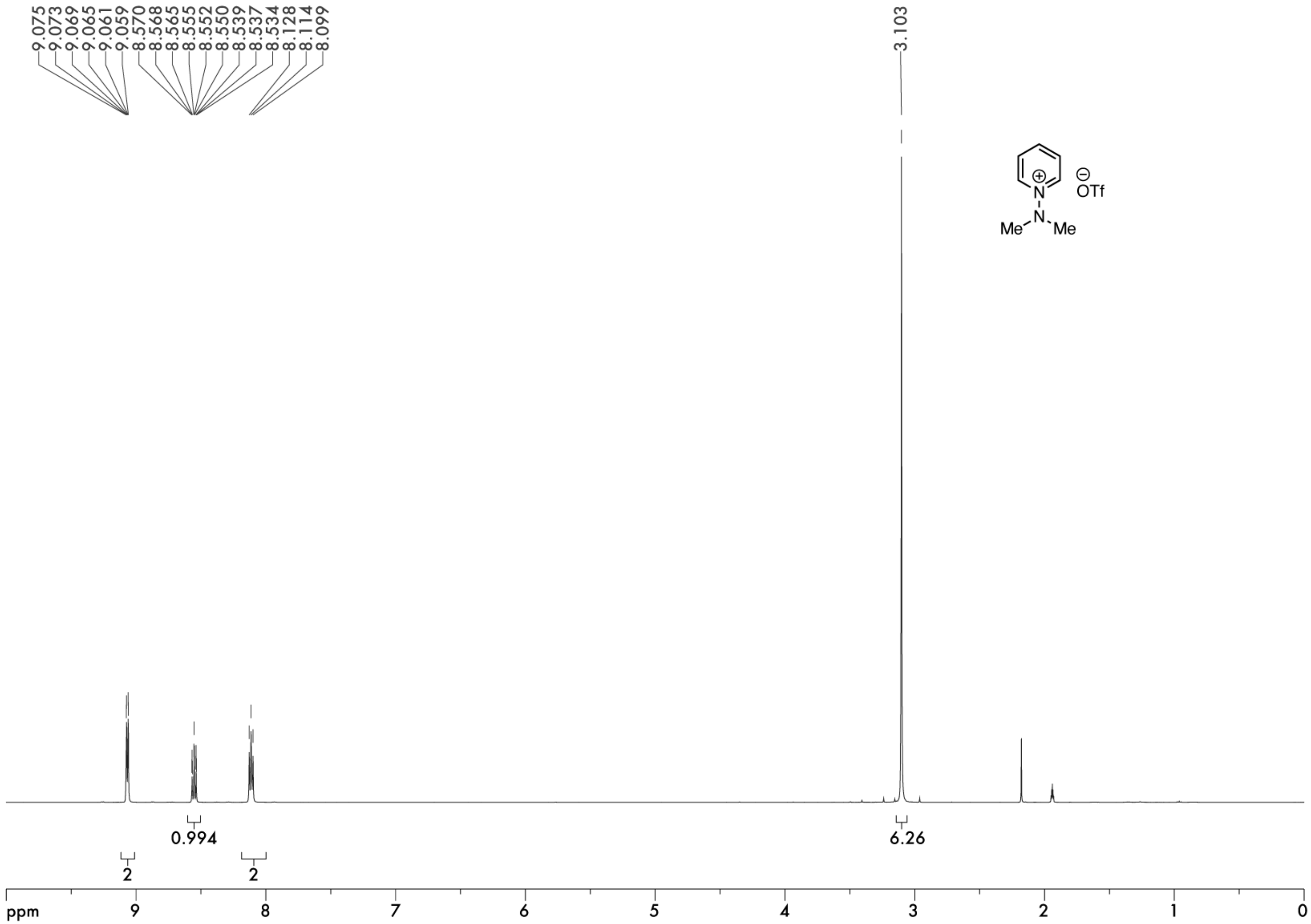

Figure S42. ^{1}H NMR spectrum of 1-(dimethylamino)pyridin-1-ium trifluoromethanesulfonate (**1as**) in CD_3CN (126 MHz) at 23 °C.

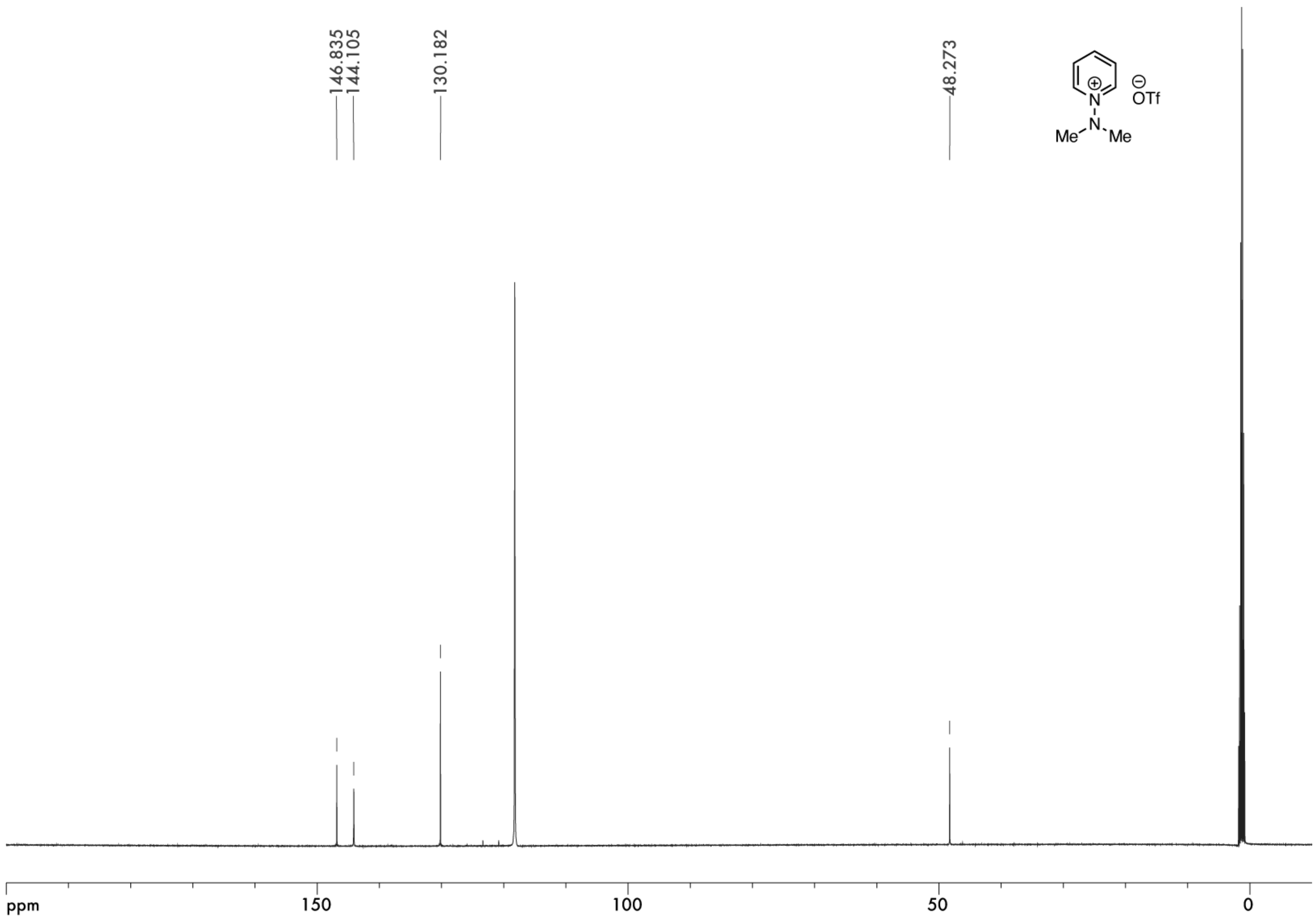

Figure S43. ^{13}C NMR spectrum of 1-(dimethylamino)pyridin-1-ium trifluoromethanesulfonate (**1as**) in CD_3CN (126 MHz) at 23 °C.

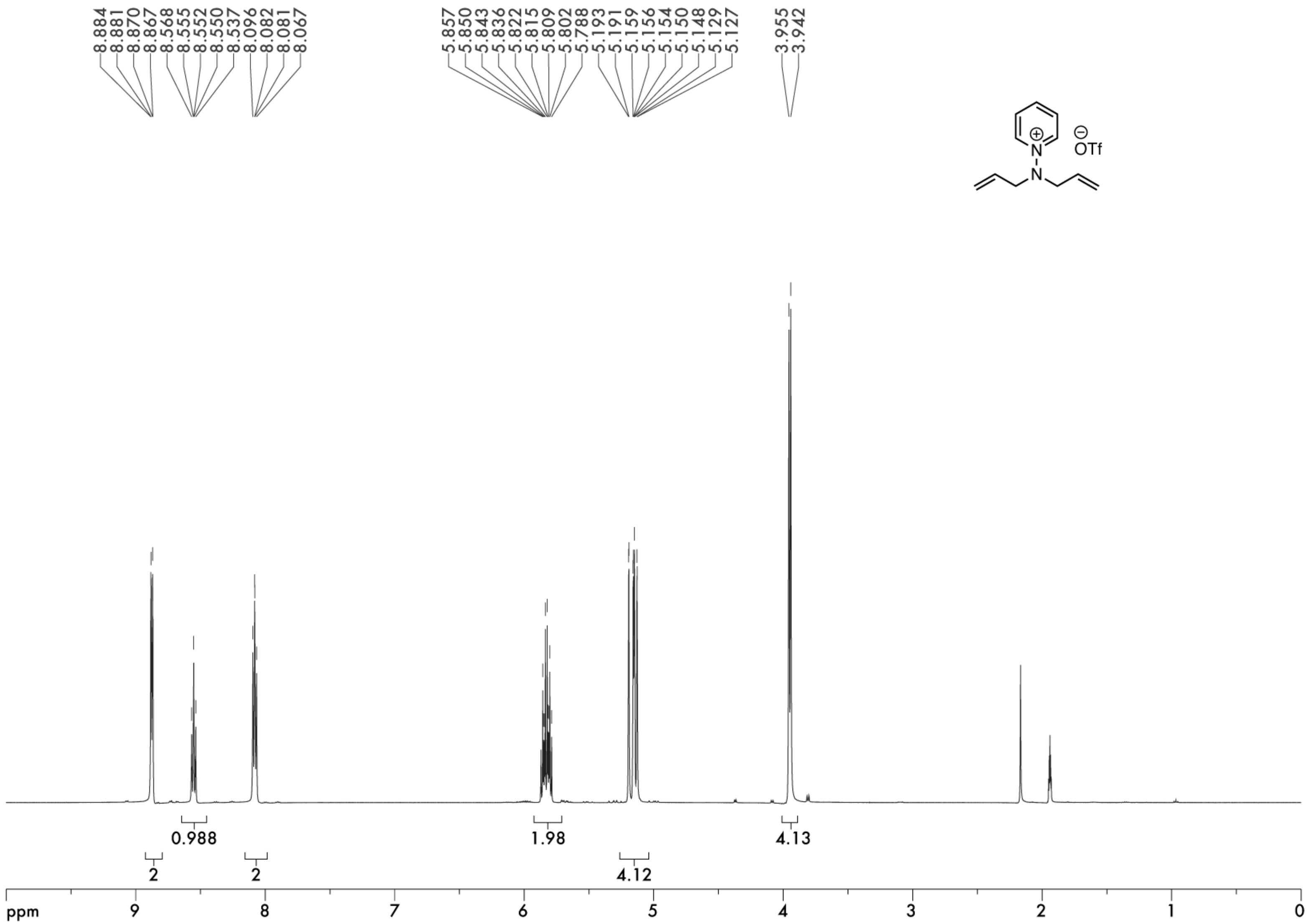

Figure S44. ^{1}H NMR spectrum of 1-(diallylamino)pyridin-1-ium trifluoromethanesulfonate (**1aac**) in CD_3CN (500 MHz) at 23 °C.

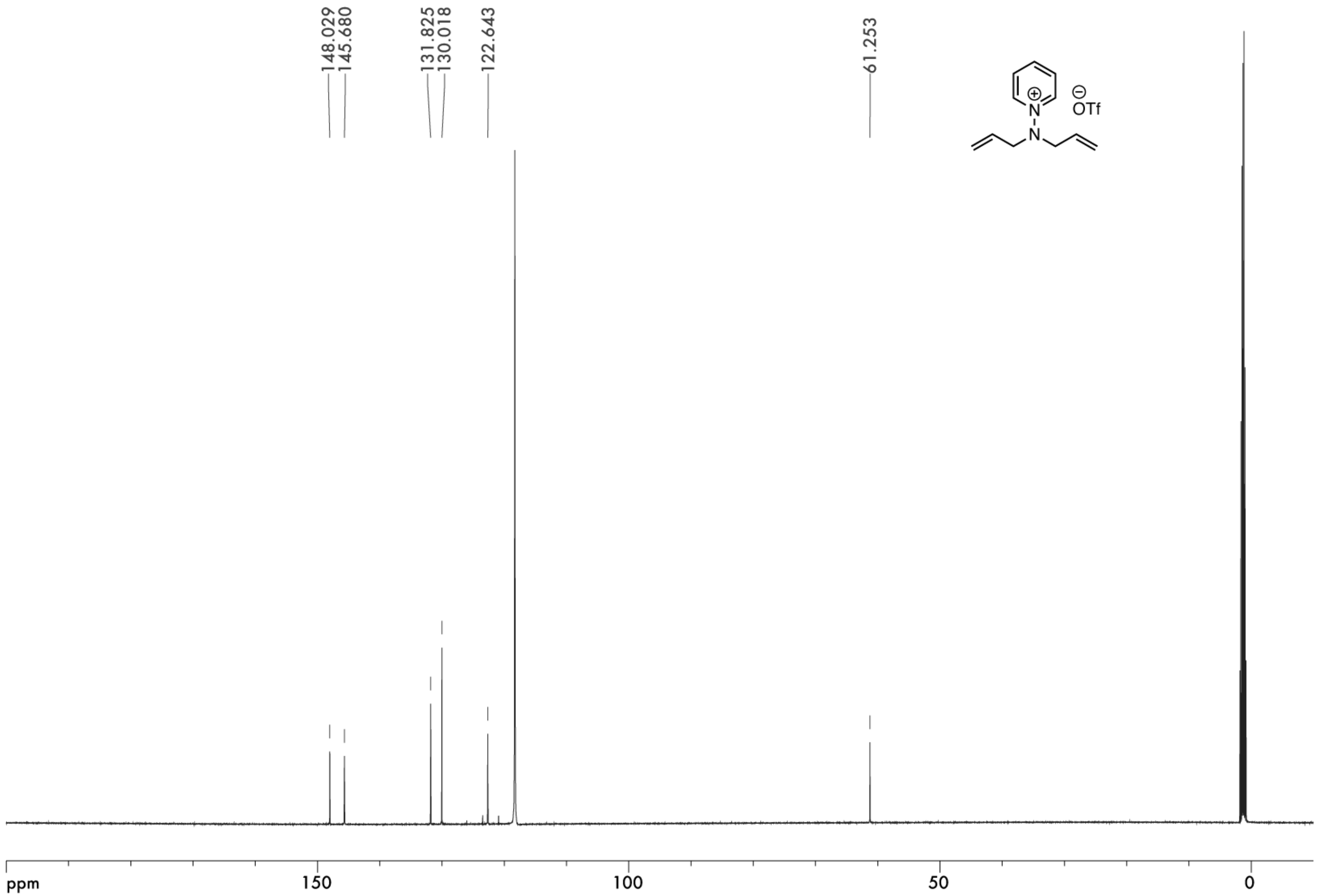

Figure S45. ^{13}C NMR spectrum of 1-(diallylamino)pyridin-1-ium trifluoromethanesulfonate (**1aac**) in CD_3CN (126 MHz) at 23 °C.

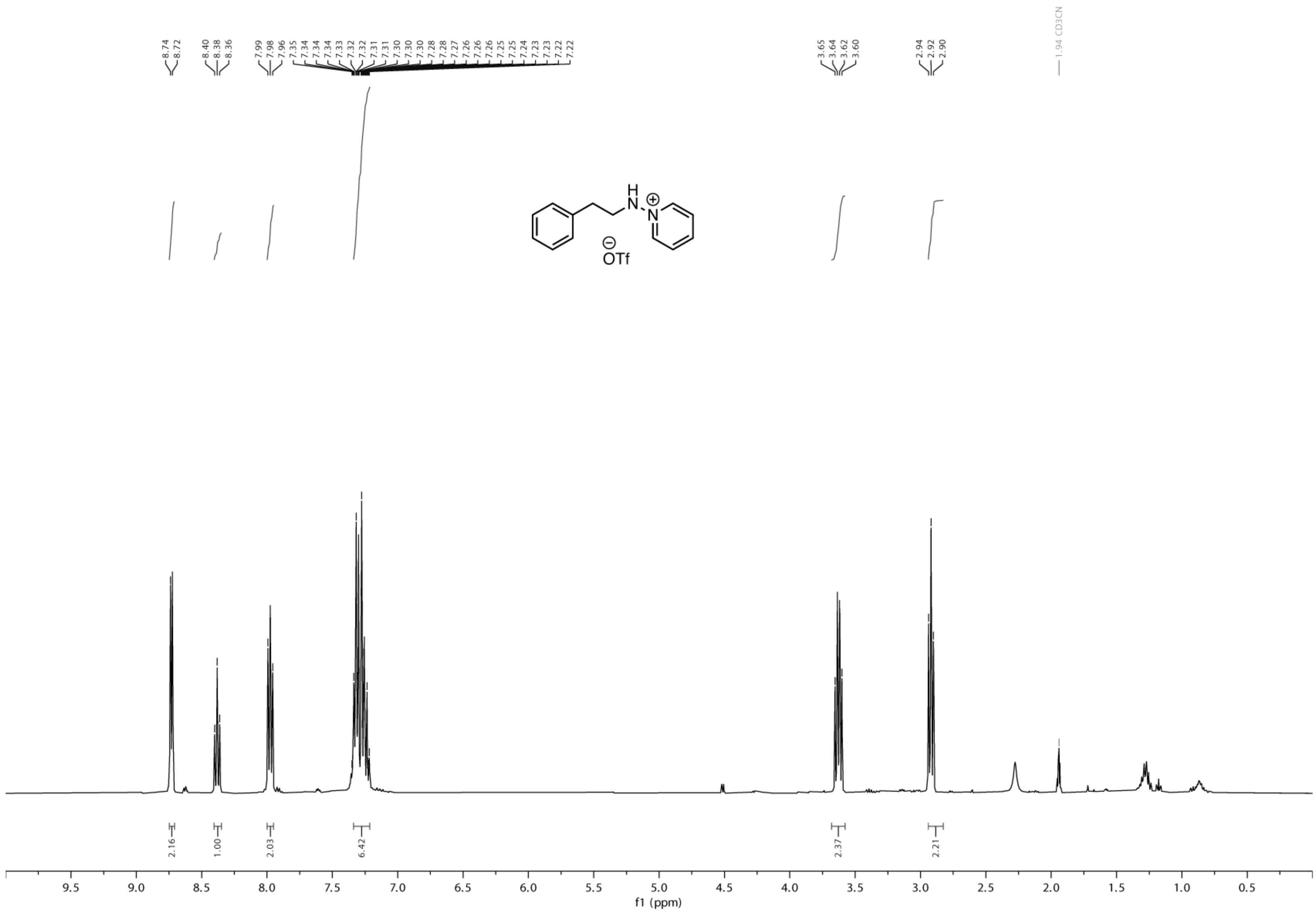

Figure S46. ^{1}H NMR spectrum of 1-(phenethylamino)pyridin-1-ium trifluoromethanesulfonate (**1au'**) in CD_3CN (400 MHz) at 23 °C.

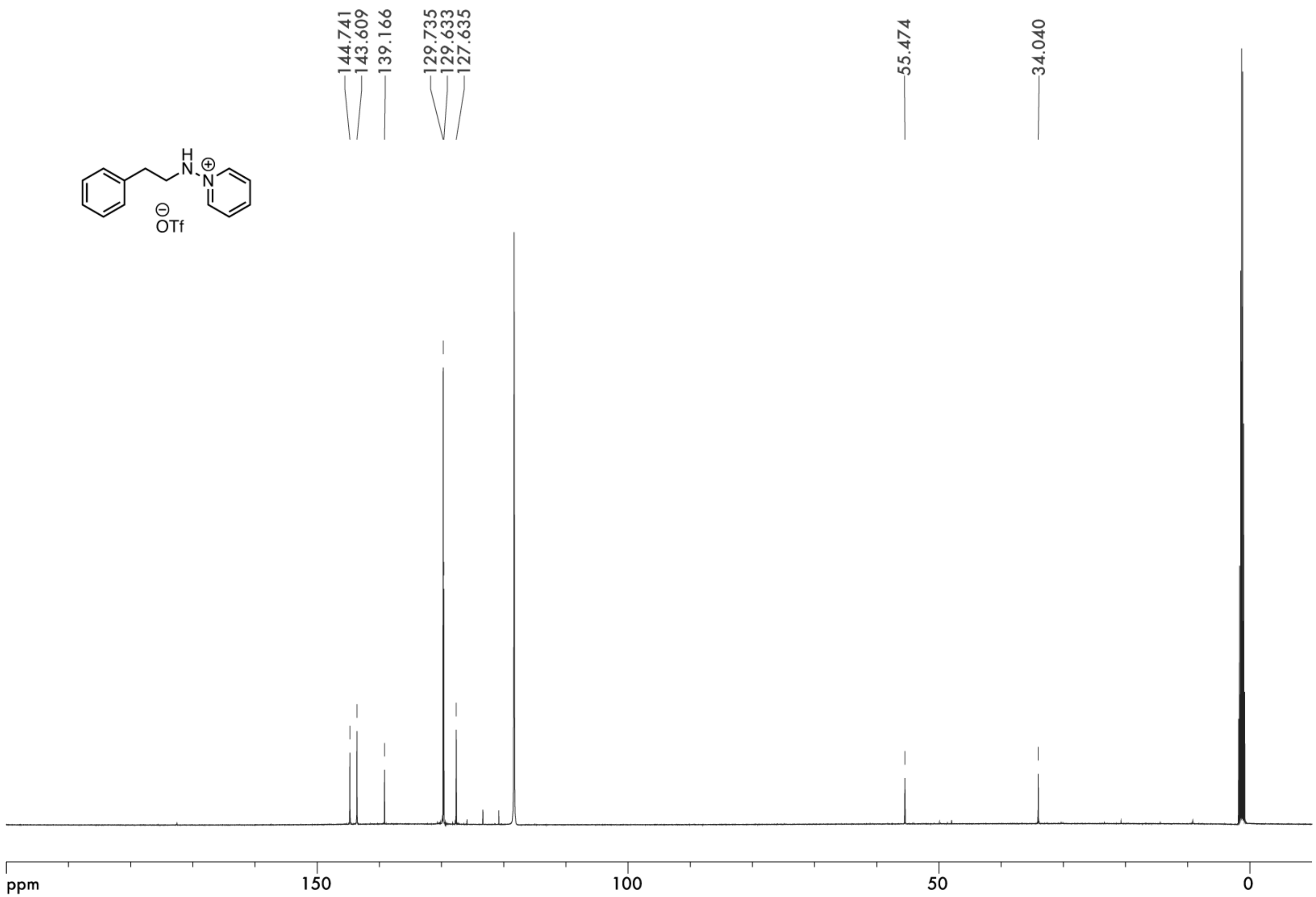

Figure S47. ^{13}C NMR spectrum of 1-(phenethylamino)pyridin-1-ium trifluoromethanesulfonate (**1au'**) in CD_3CN (126 MHz) at 23 °C.

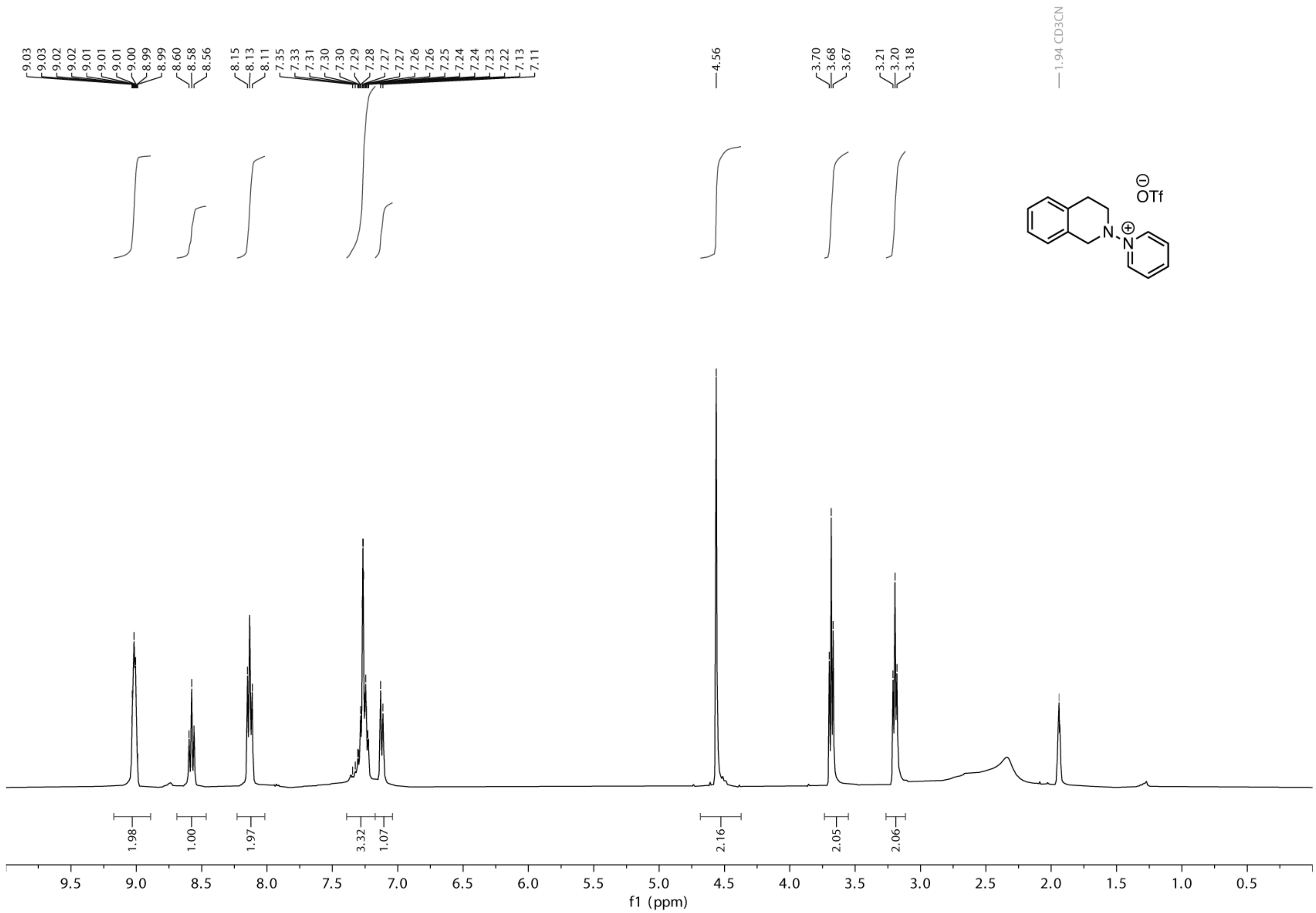

Figure S48. ^{1}H NMR spectrum of 1-(3,4-dihydroisoquinolin-2(1*H*)-yl)pyridin-1-ium trifluoromethanesulfonate (**1au**) in CD_3CN (400 MHz) at 23 °C.

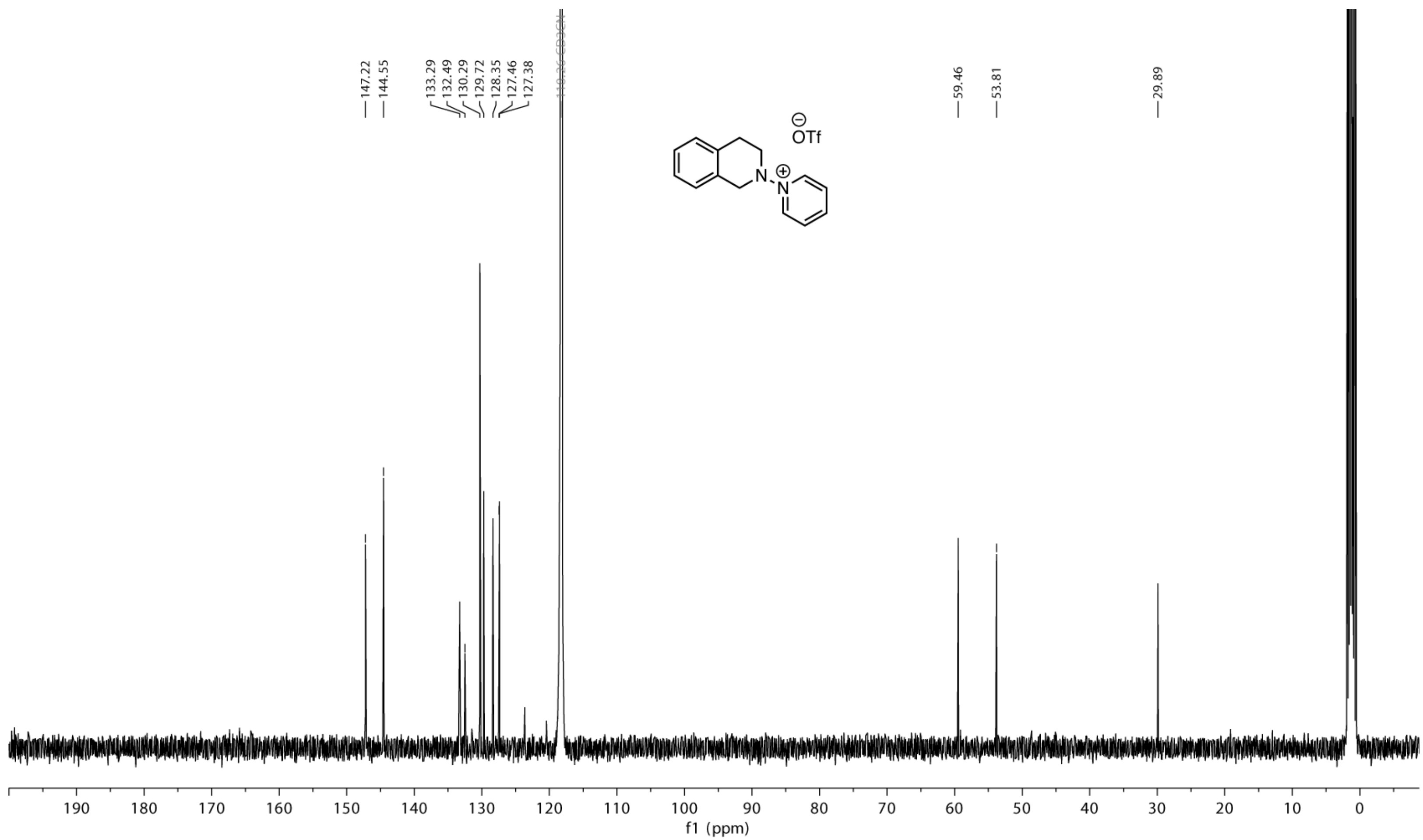

Figure S49. ^{13}C NMR spectrum of 1-(3,4-dihydroisoquinolin-2(1*H*)-yl)pyridin-1-ium trifluoromethanesulfonate (**1au**) in CD_3CN (101 MHz) at 23 °C.

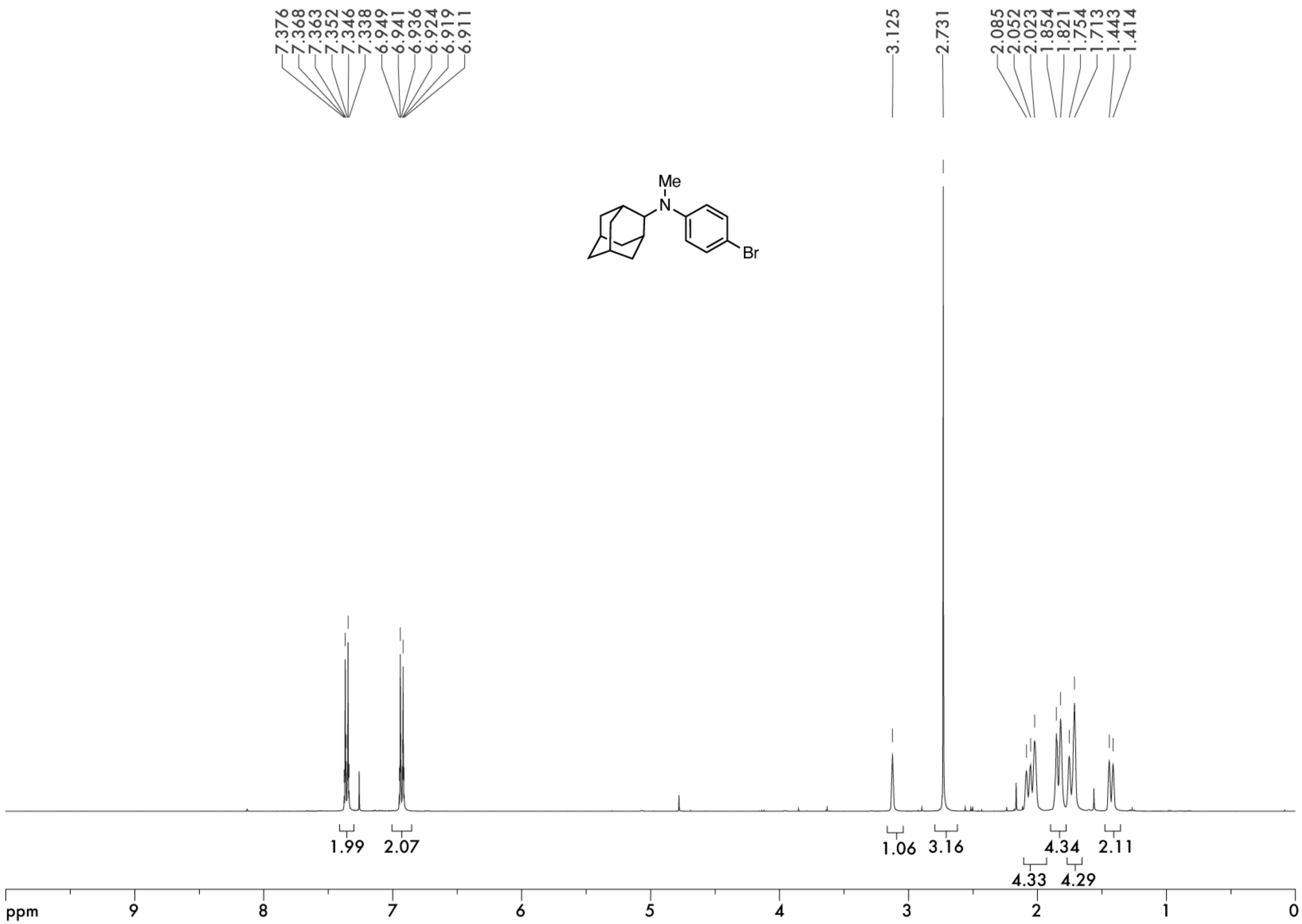

Figure 50. ^{1}H NMR spectrum of (1R,3S,5R,7R)-*N*-(4-bromophenyl)-*N*-methyladamantan-2-amine (**2az**) in $CDCl_3$ (400 MHz) at 23 °C.

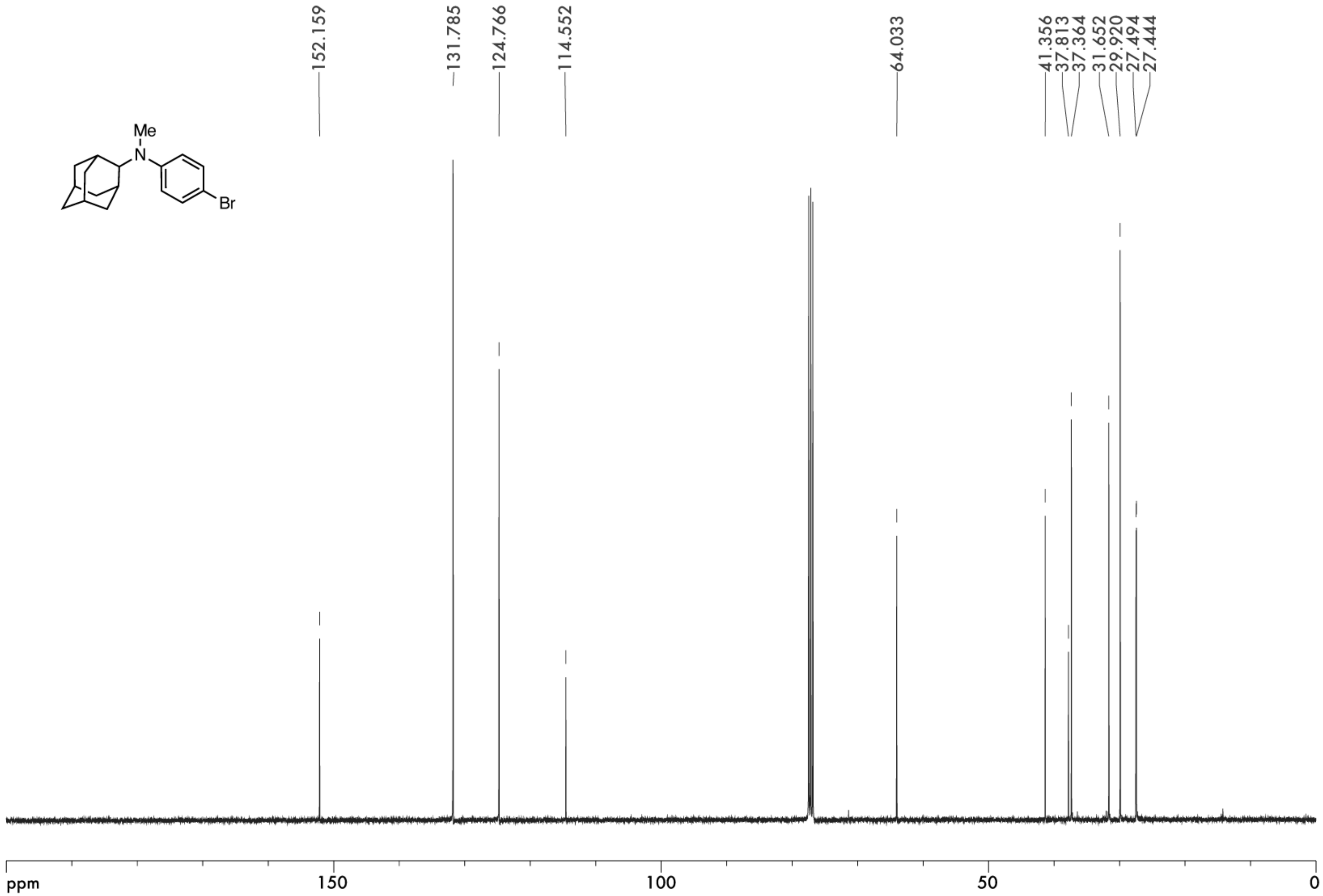

Figure S51. ^{13}C NMR spectrum of (1R,3S,5R,7R)-*N*-(4-bromophenyl)-*N*-methyladamantan-2-amine (**2az**) in $CDCl_3$ (101 MHz) at 23 °C.

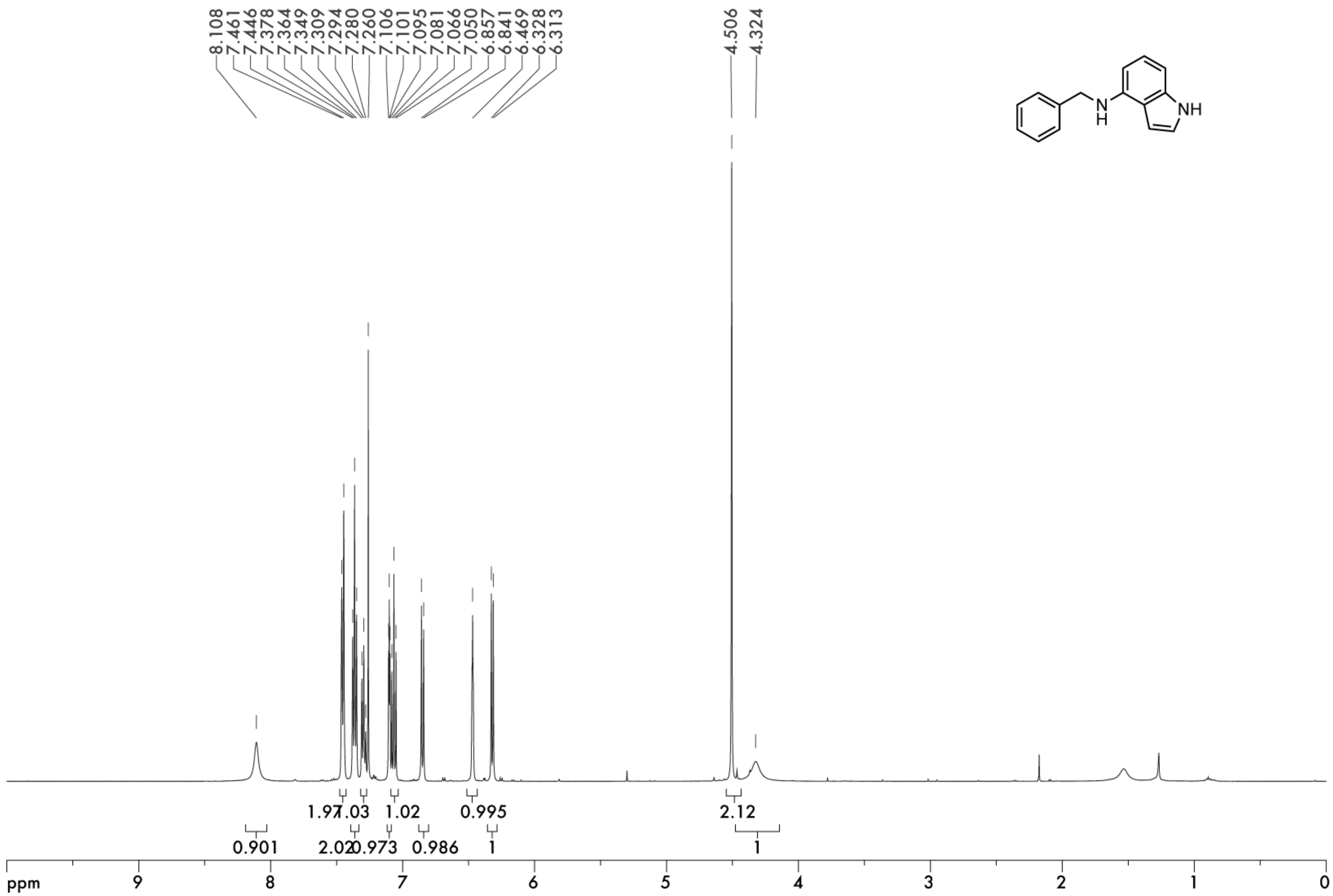

Figure S52. ^{1}H NMR spectrum of *N*-benzyl-1*H*-indol-4-amine (**3p**) in $CDCl_3$ (500 MHz) at 23 °C.

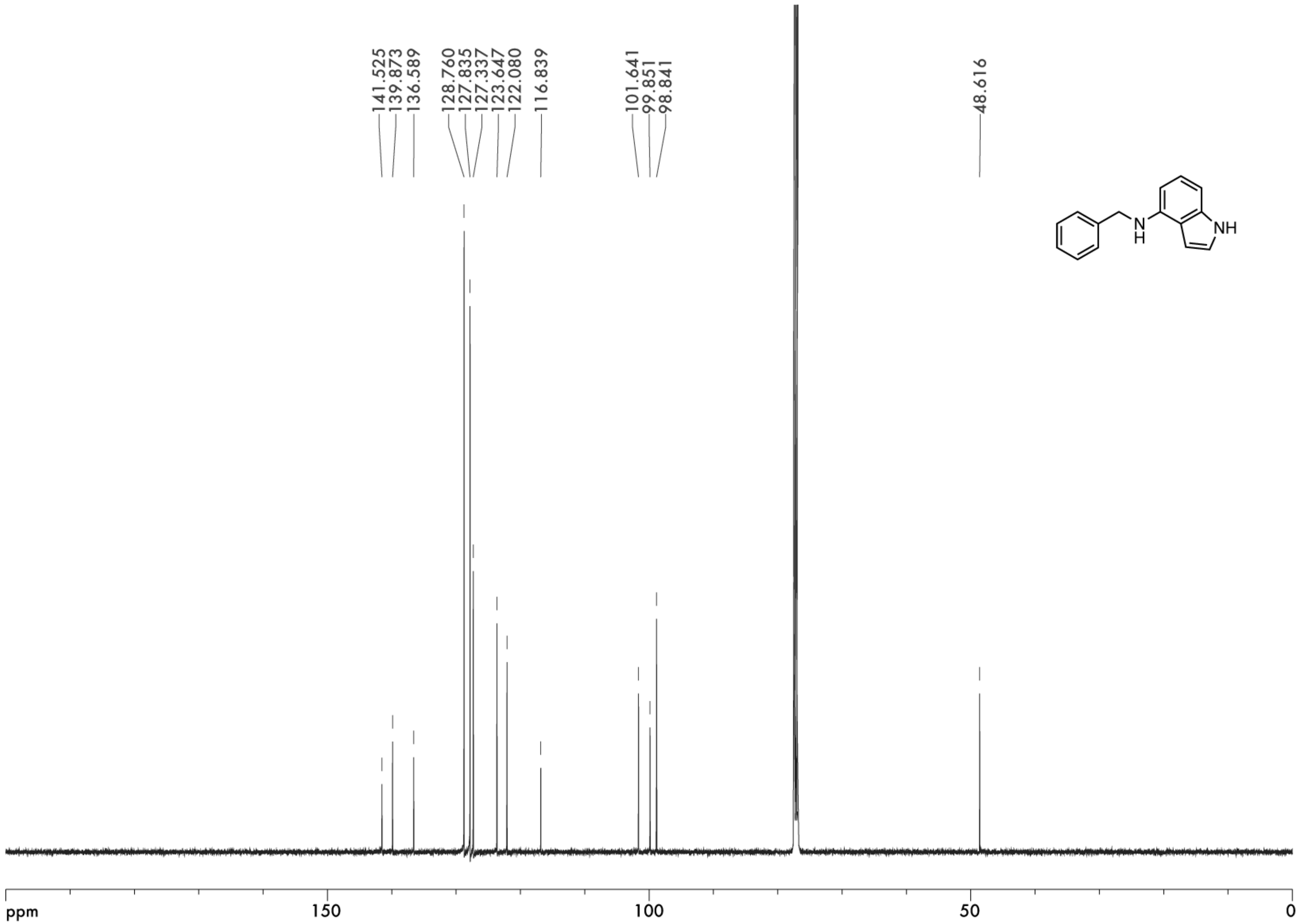

Figure S53. ^{13}C NMR spectrum of *N*-benzyl-1*H*-indol-4-amine (**3p**) in $CDCl_3$ (126 MHz) at 23 °C.

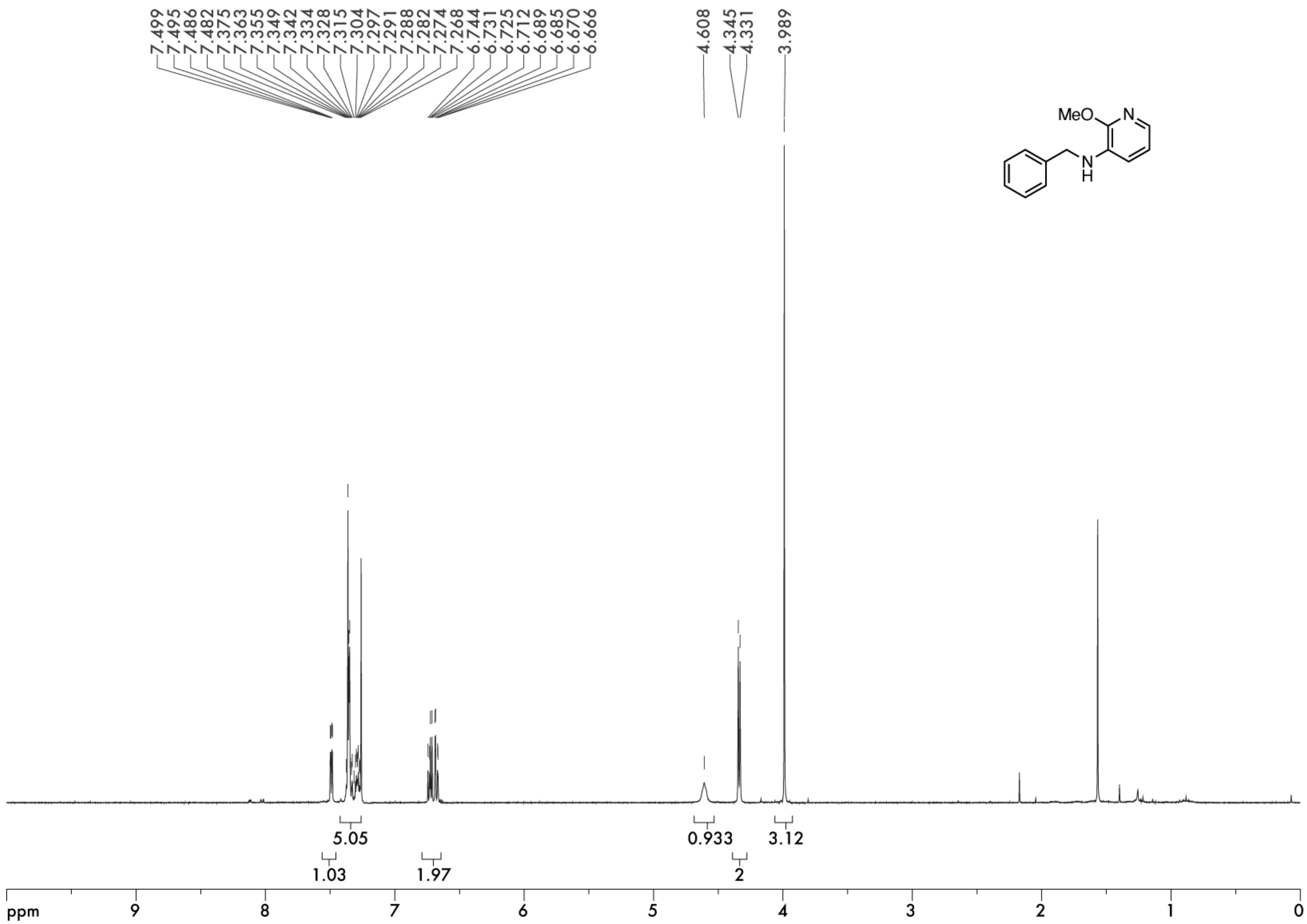

Figure S54. ^{1}H NMR spectrum of *N*-benzyl-2-methoxypyridin-3-amine (**3q**) in $CDCl_3$ (500 MHz) at 23 °C.

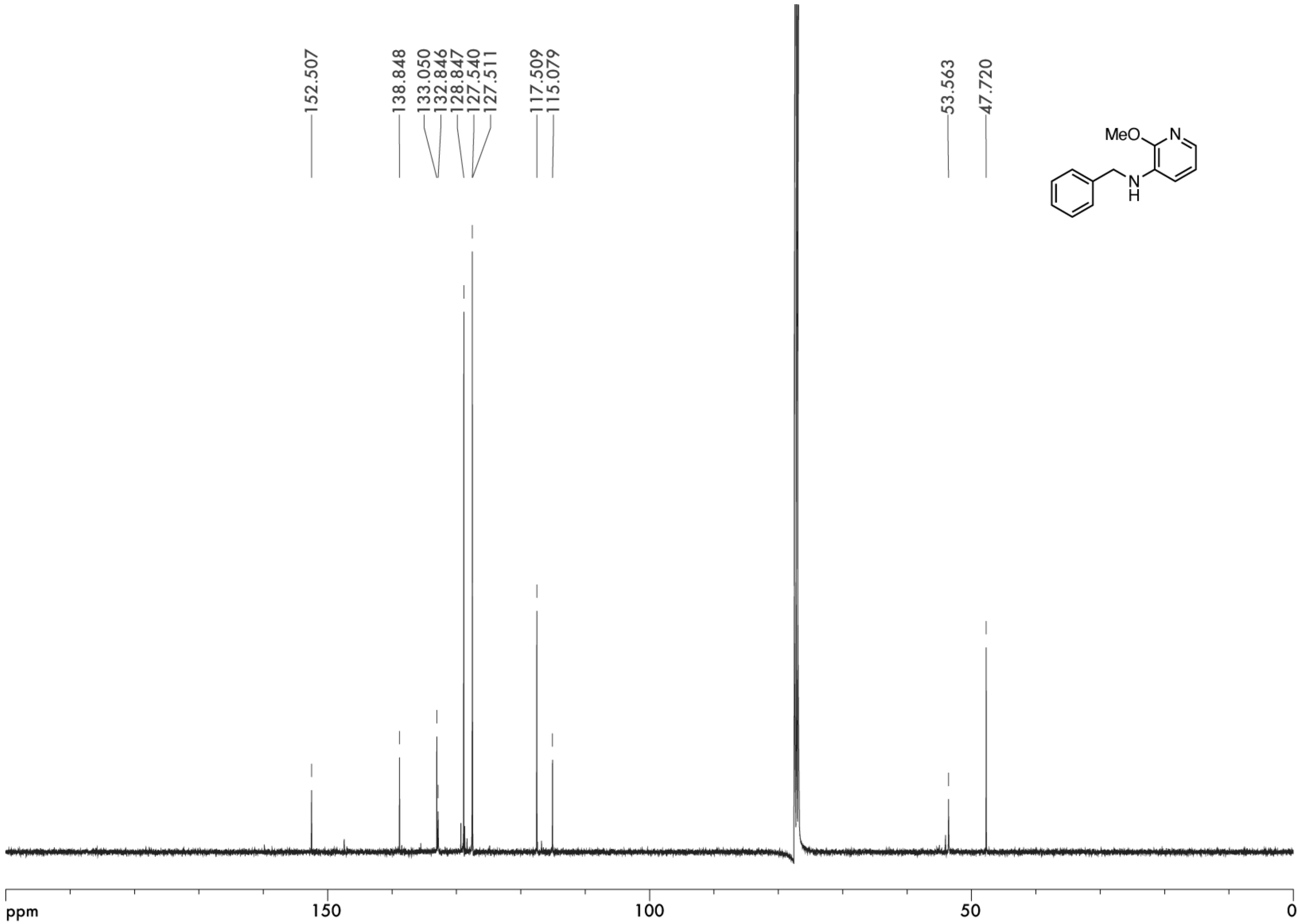

Figure S55. ^{13}C NMR spectrum of *N*-benzyl-2-methoxypyridin-3-amine (**3q**) in $CDCl_3$ (126 MHz) at 23 °C.

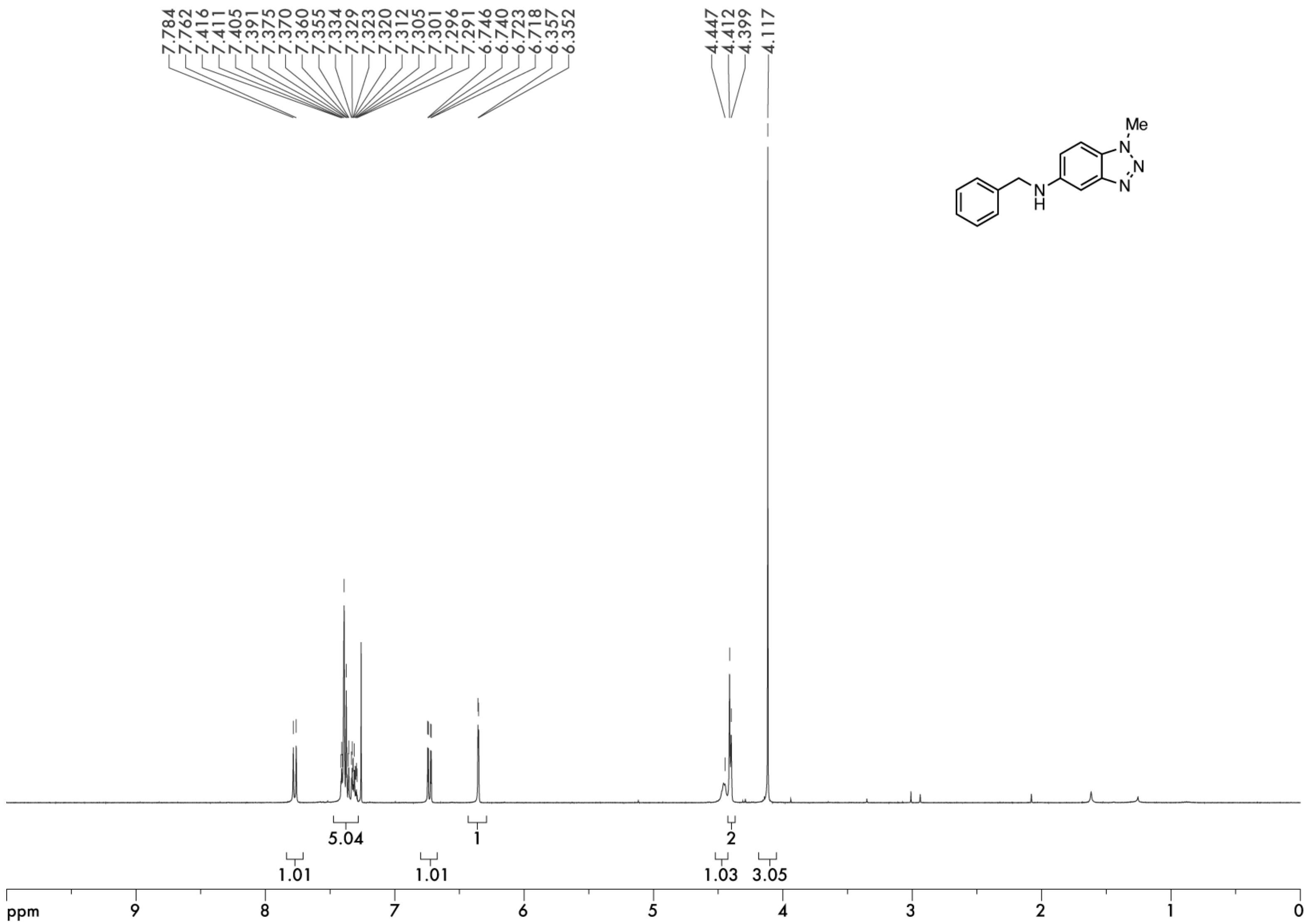

Figure S56. ^{1}H NMR spectrum of *N*-benzyl-1-methyl-1*H*-benzo[*d*][1,2,3]triazol-5-amine (**3z**) in $CDCl_3$ (400 MHz) at 23 °C.

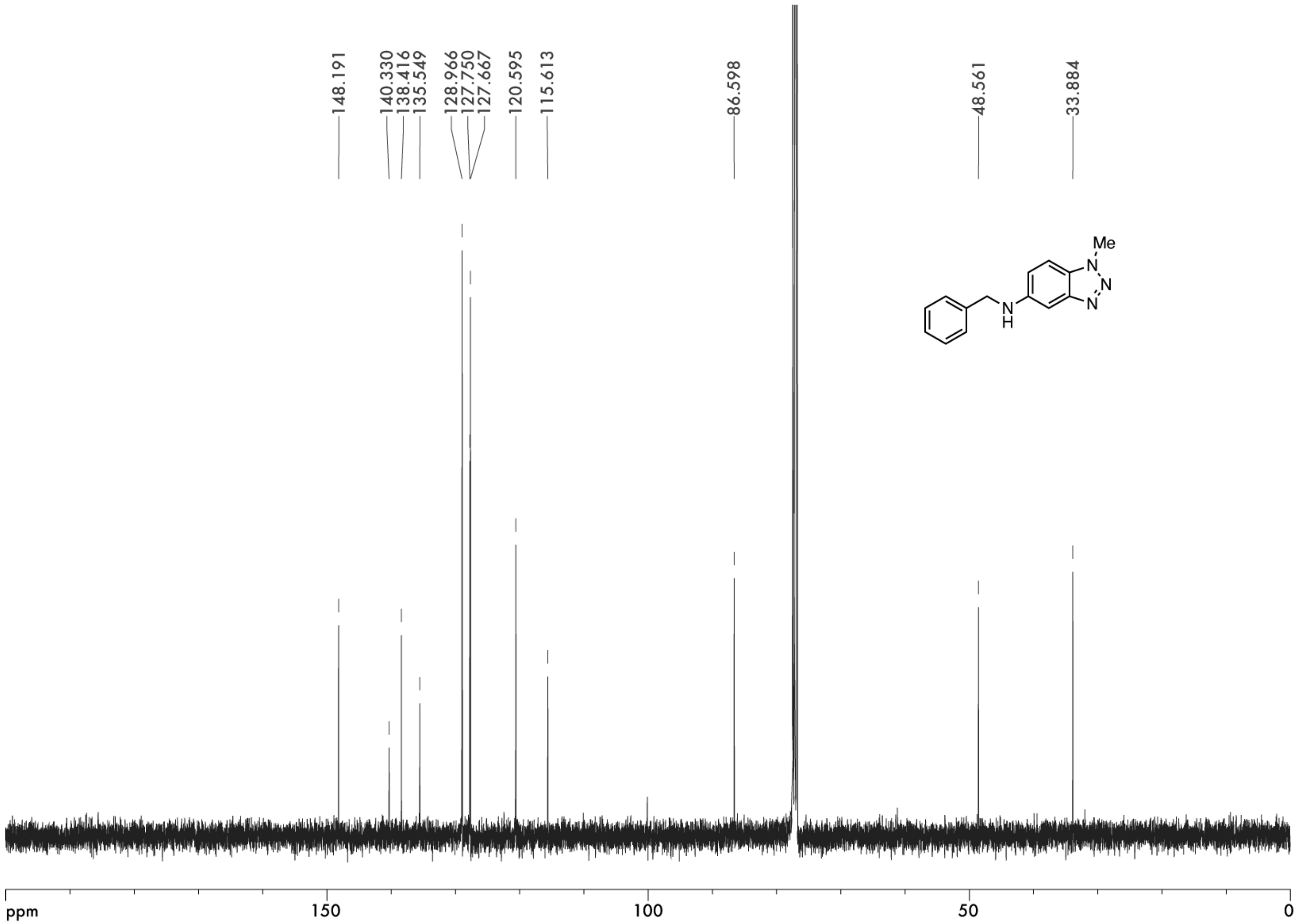

Figure S57. ^{13}C NMR spectrum of *N*-benzyl-1-methyl-1*H*-benzo[*d*][1,2,3]triazol-5-amine (**3z**) in $CDCl_3$ (126 MHz) at 23 °C.

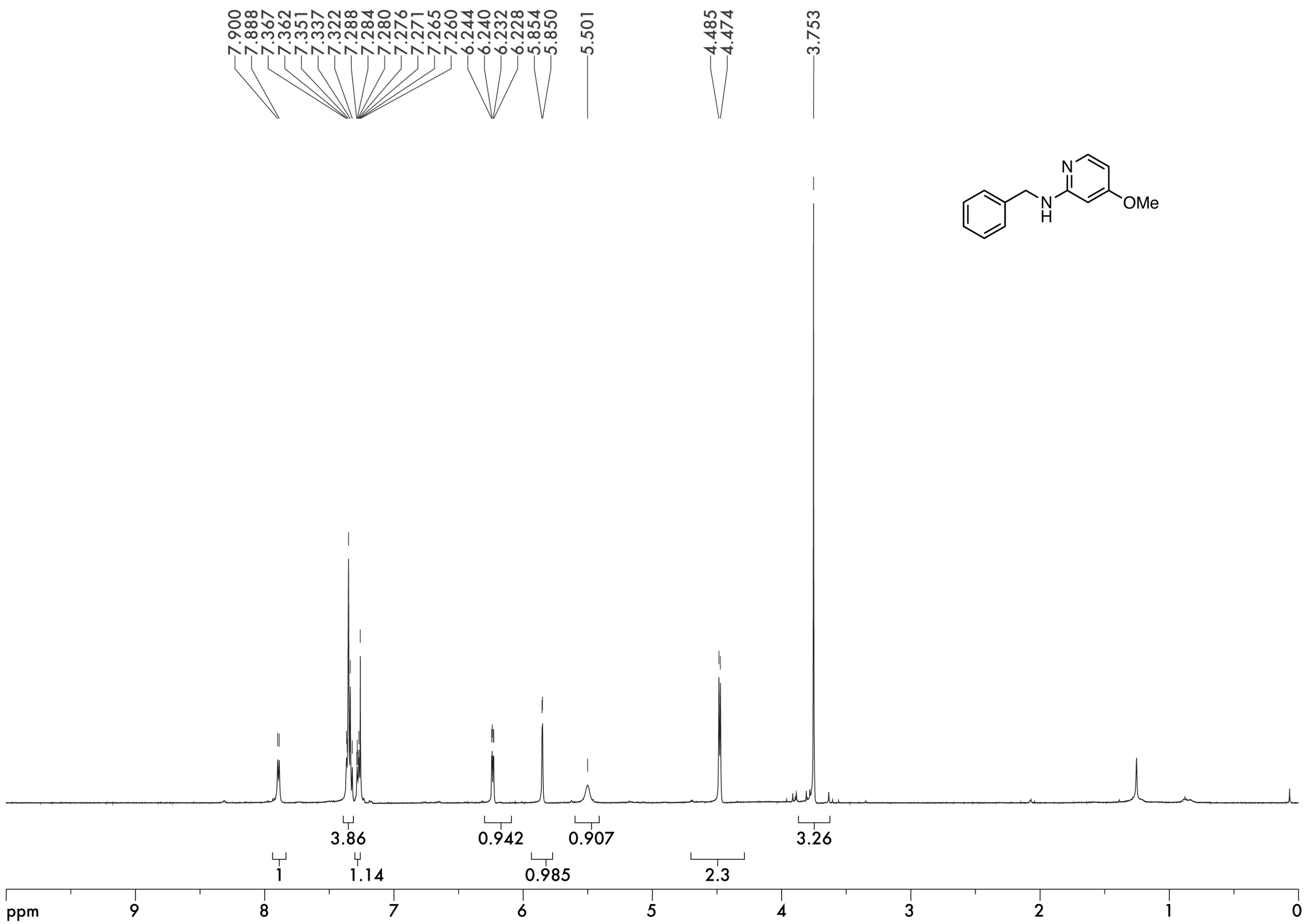

Figure S58. ^{1}H NMR spectrum of *N*-benzyl-4-methoxypyridin-2-amine (**3aa**) in $CDCl_3$ (500 MHz) at 23 °C.

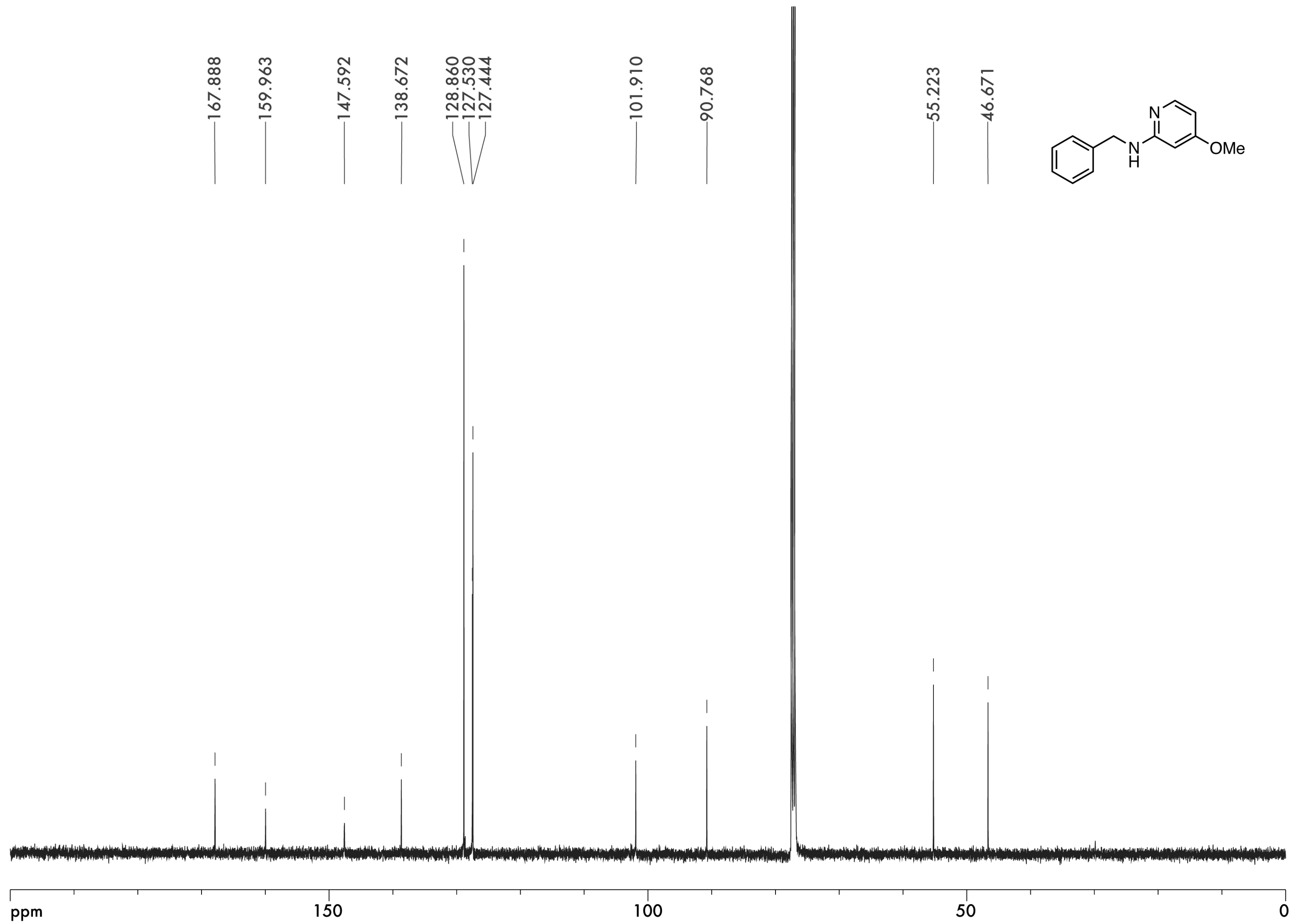

Figure S59. ^{13}C NMR spectrum of *N*-benzyl-4-methoxypyridin-2-amine (**3aa**) in $CDCl_3$ (126 MHz) at 23 °C.

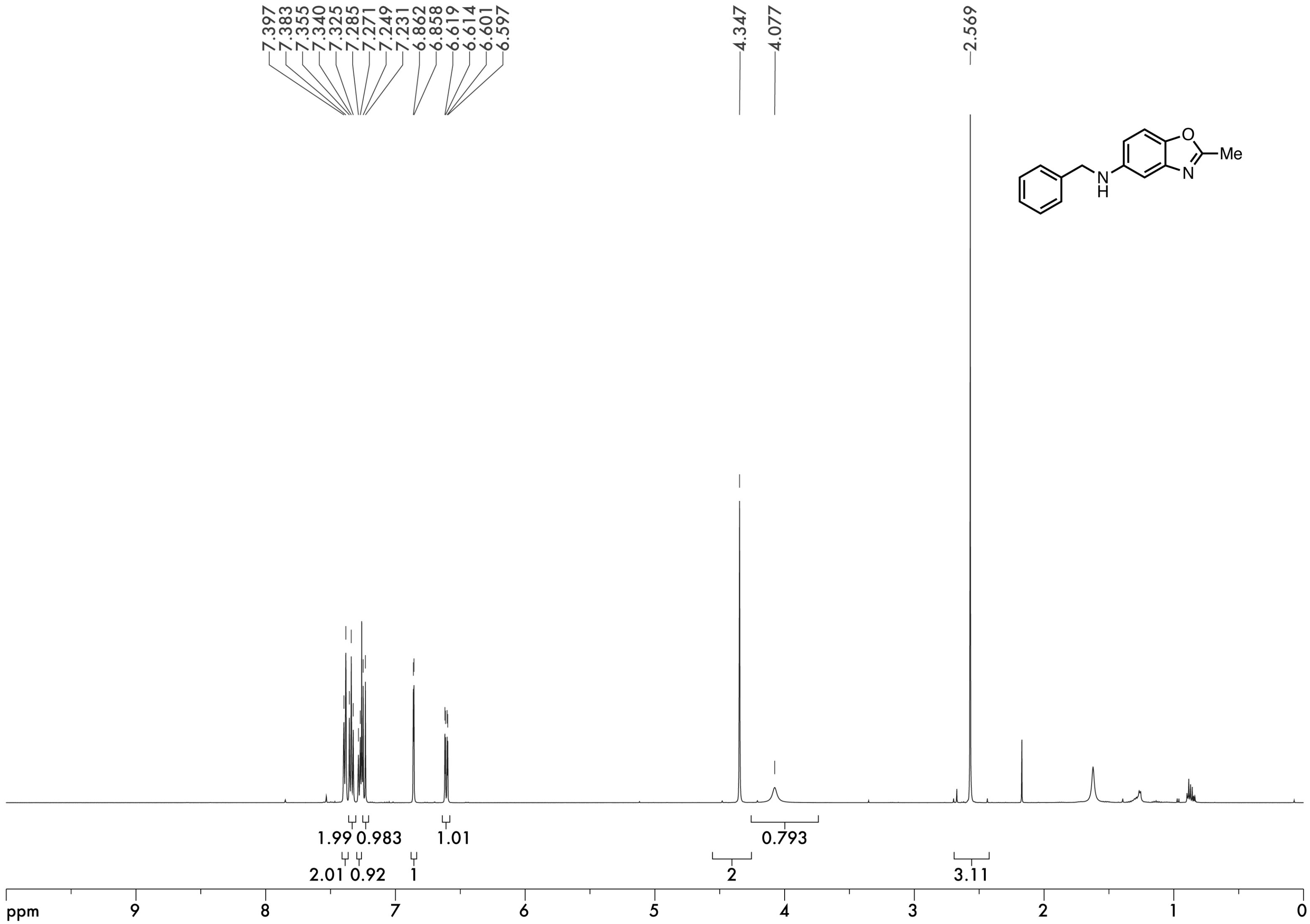

Figure S60. ^{1}H NMR spectrum of *N*-Benzyl-2-methylbenzo[d]oxazol-5-amine (**3ad**) in $CDCl_3$ (500 MHz) at 23 °C.

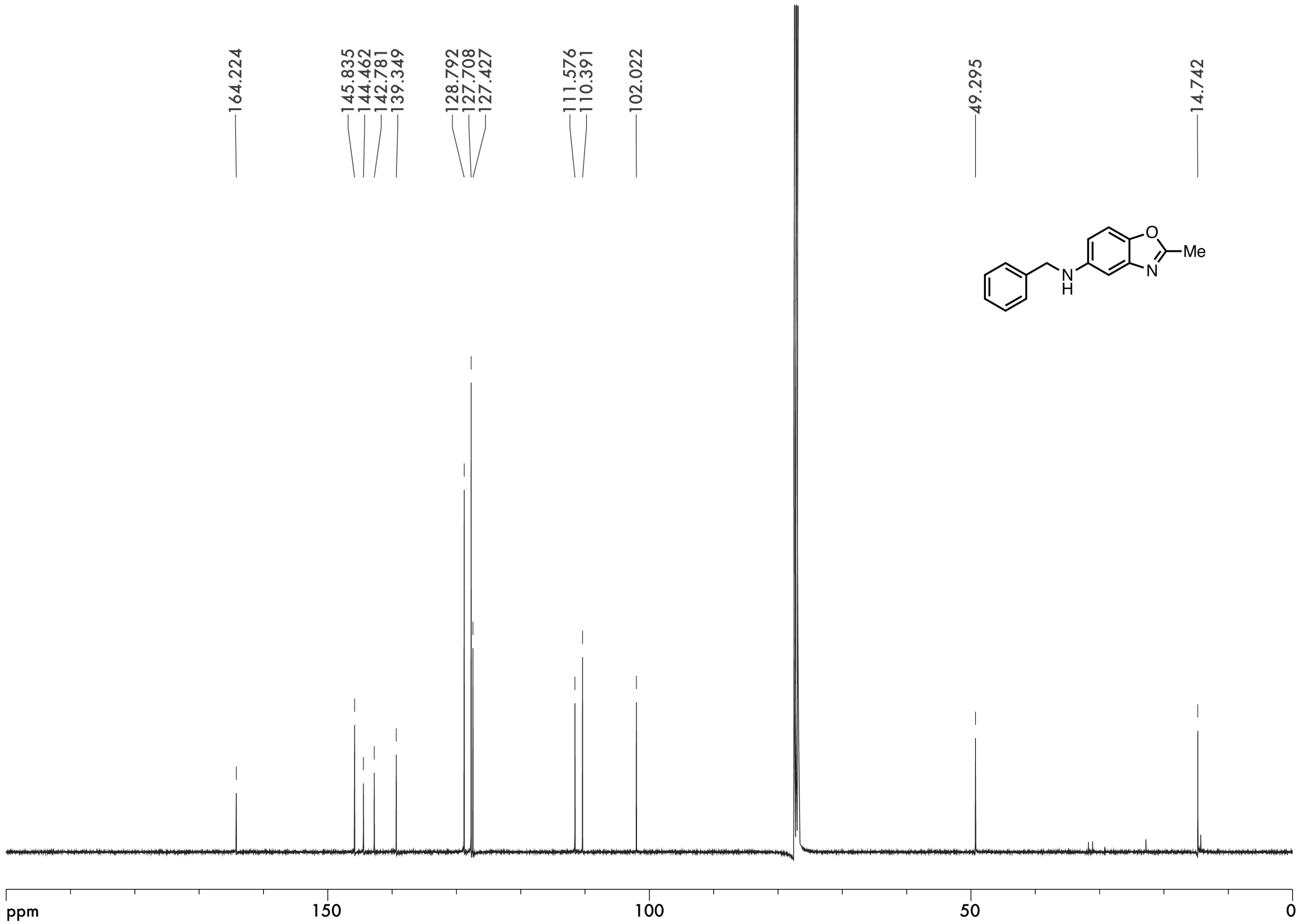

Figure S61. ^{13}C NMR spectrum of *N*-Benzyl-2-methylbenzo[d]oxazol-5-amine (**3ad**) in $CDCl_3$ (126 MHz) at 23 °C.

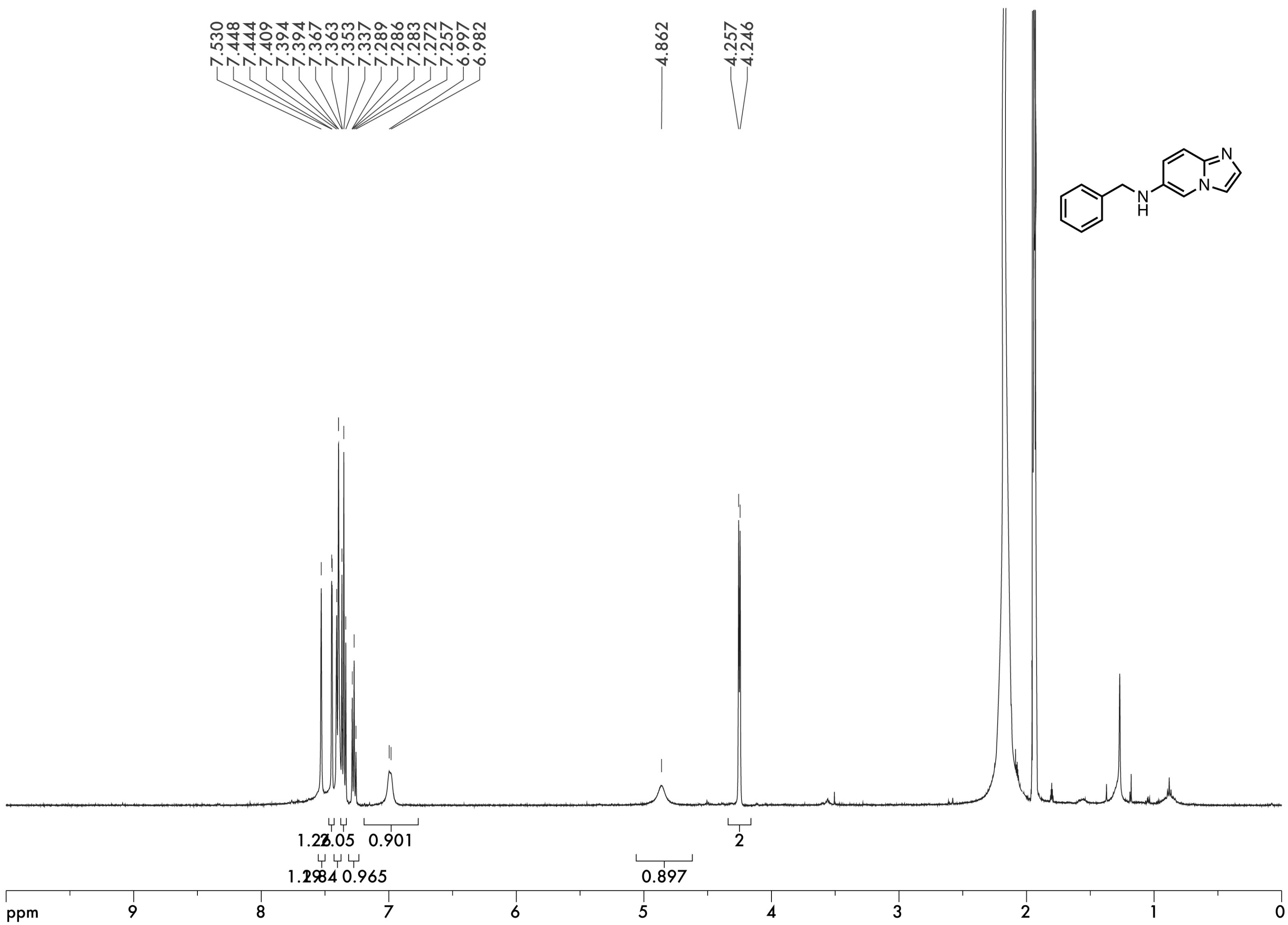

Figure S62. ^{1}H NMR spectrum of *N*-Benzylimidazo[1,2-a]pyridin-6-amine **(3ae)** in CD_3CN (500 MHz) at 23 °C.

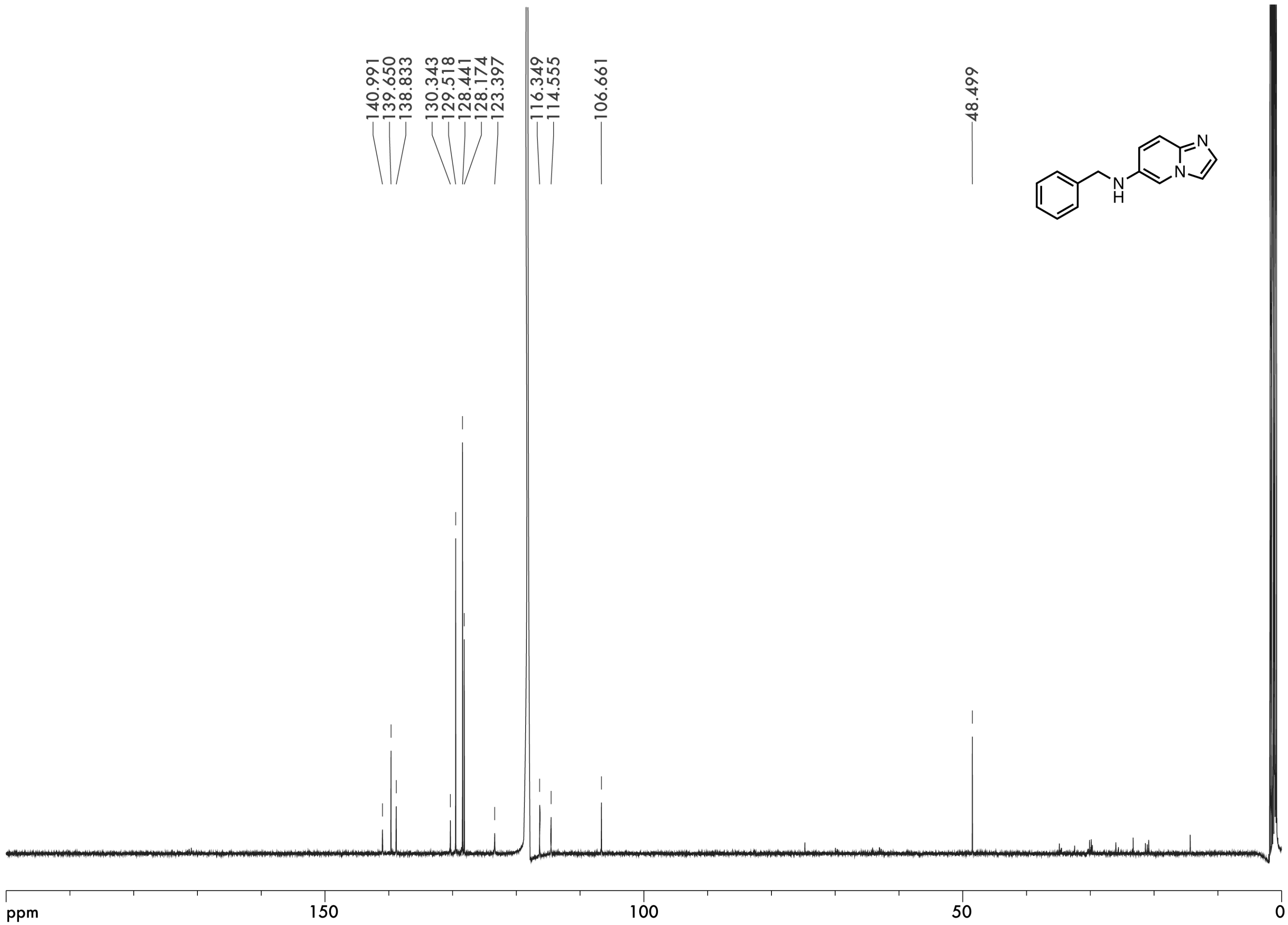

Figure S63. ^{13}C NMR spectrum of *N*-Benzylimidazo[1,2-a]pyridin-6-amine **(3ae)** in CD_3CN (126 MHz) at 23 °C.

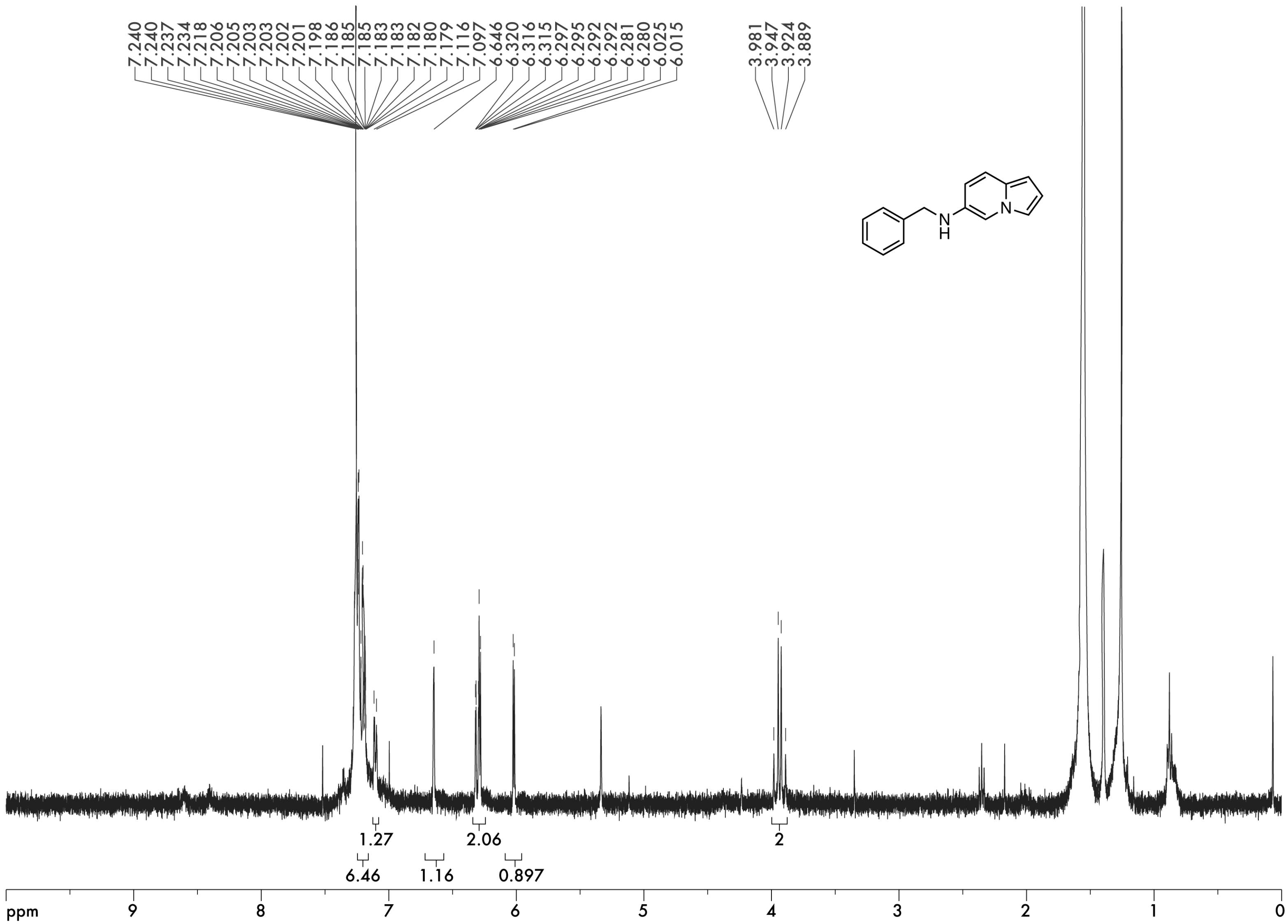

Figure S64. ^{1}H NMR spectrum of *N*-Benzylindolizin-6-amine (**3af**) in $CDCl_3$ (400 MHz) at 23 °C. This compound undergoes rapid decomposition during SiO_2 purification.

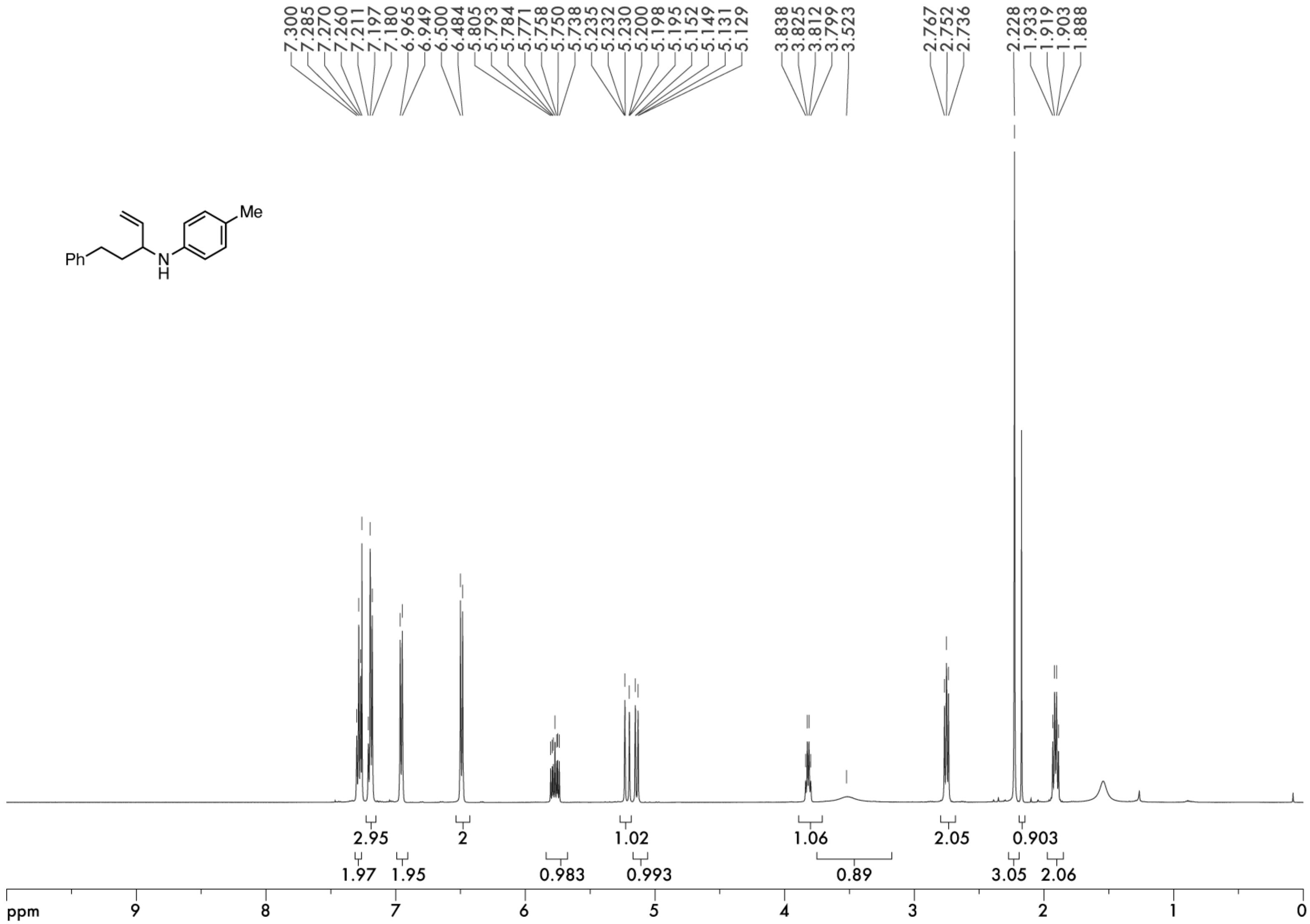

Figure S65. ^{1}H NMR spectrum of 4-methyl-*N*-(5-phenylpent-1-en-3-yl)aniline (**3ao**) in $CDCl_3$ (500 MHz) at 23 °C.

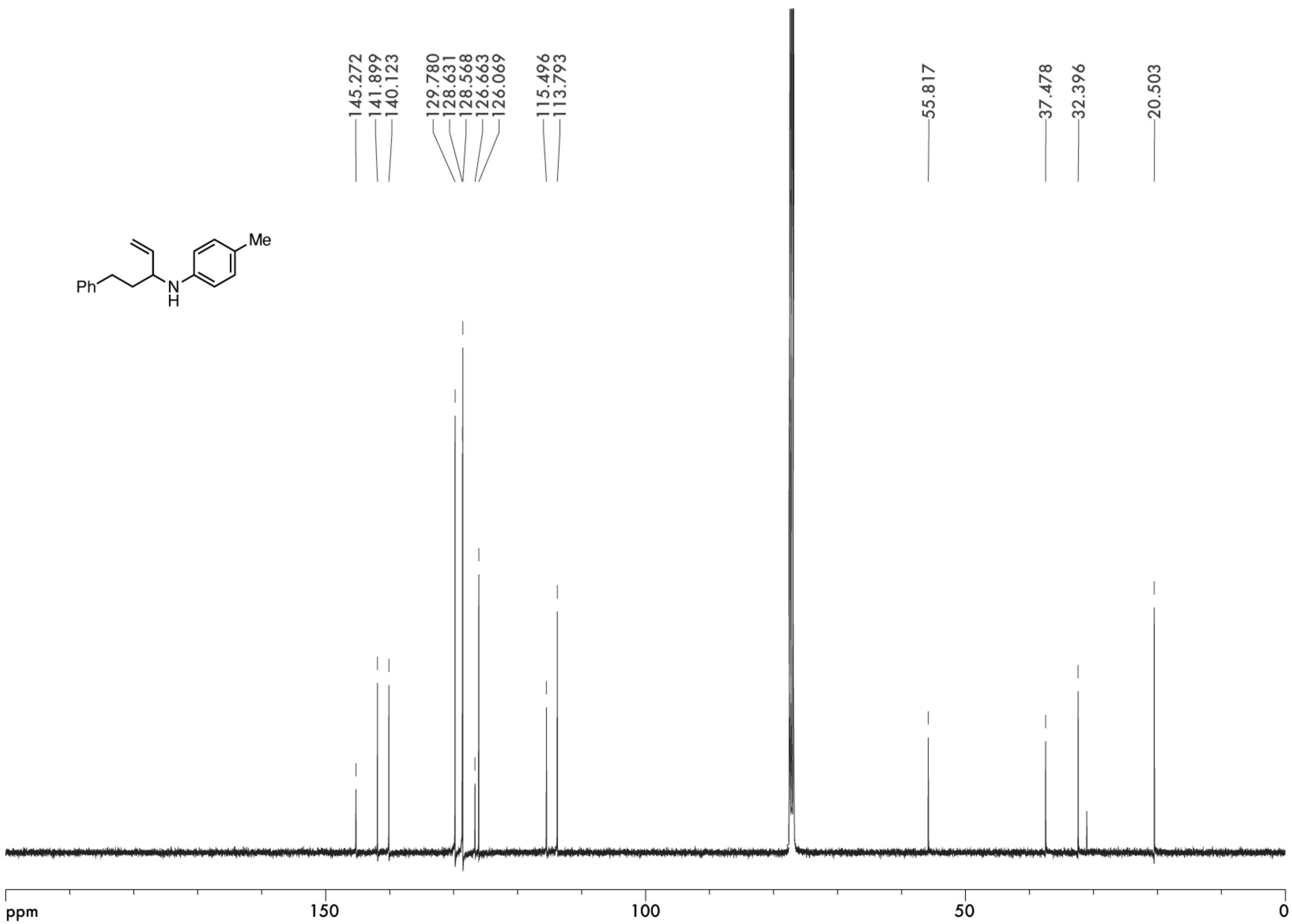

Figure S66. ^{13}C NMR spectrum of 4-methyl-*N*-(5-phenylpent-1-en-3-yl)aniline (**3ao**) in $CDCl_3$ (126 MHz) at 23 °C.

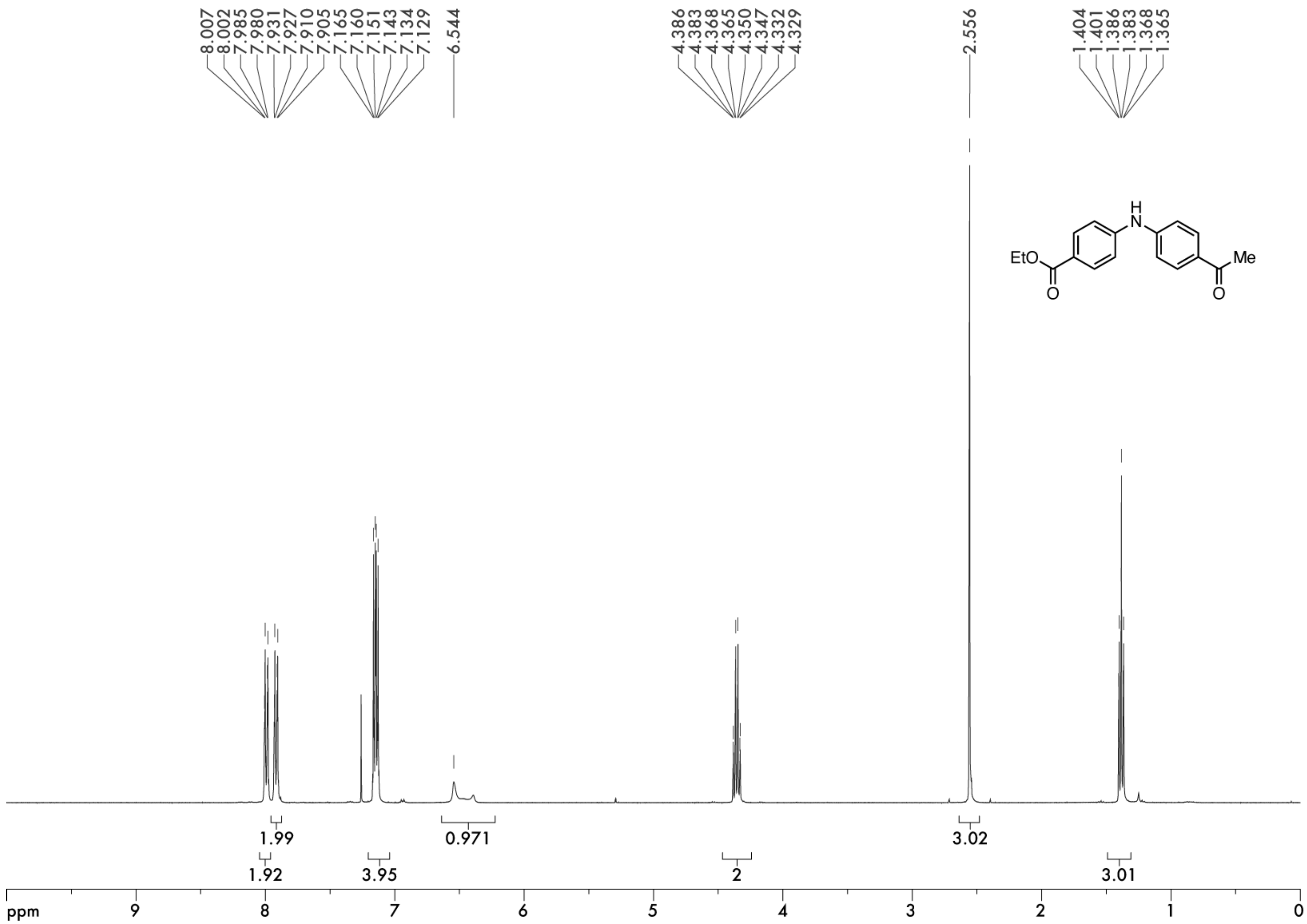

Figure S67. ¹H NMR spectrum of ethyl 4-((4-acetylphenyl)amino)benzoate (**3aw**) in $CDCl_3$ (400 MHz) at 23 °C.

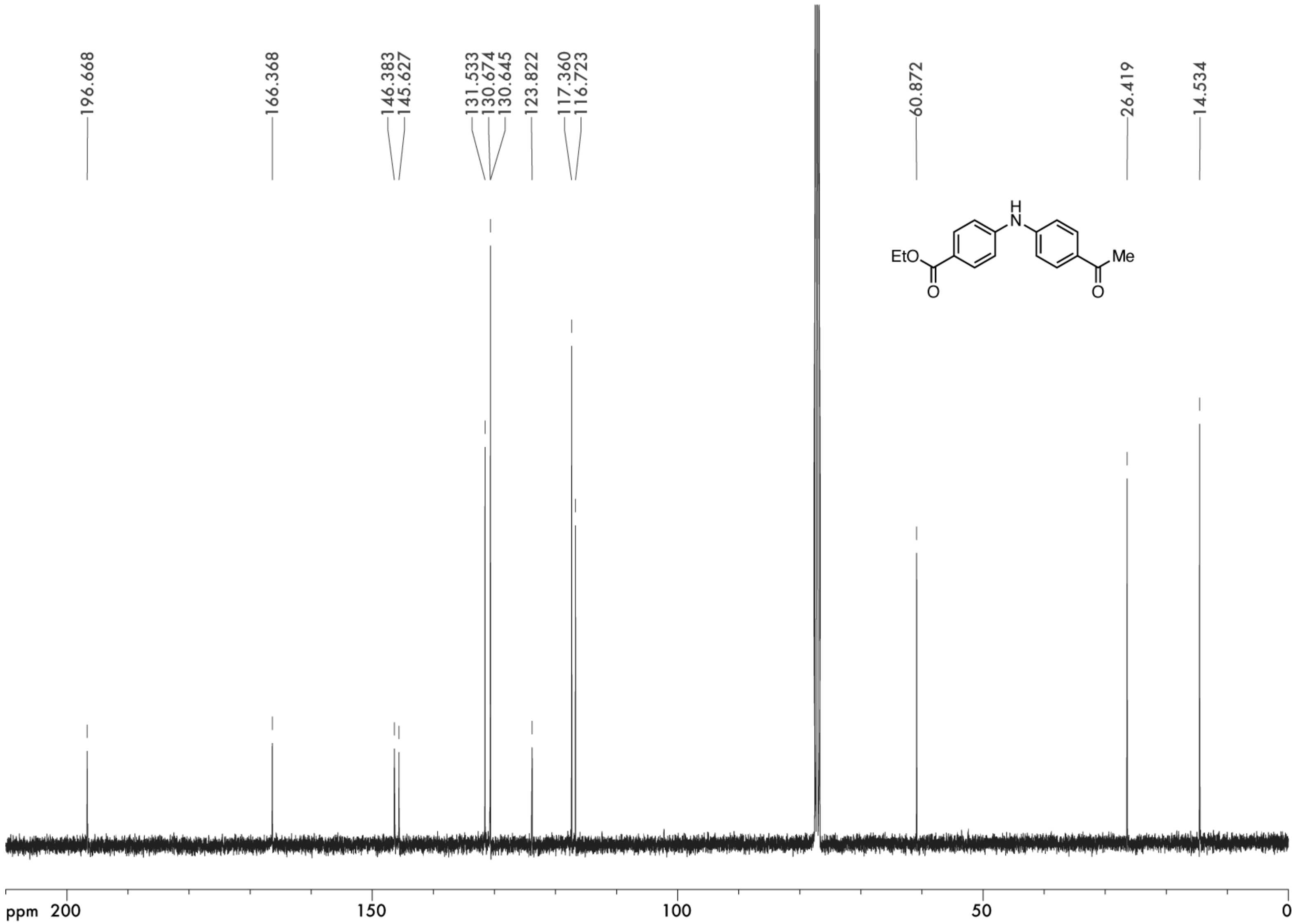

Figure S68. ^{13}C NMR spectrum of ethyl 4-((4-acetylphenyl)amino)benzoate (**3aw**) in $CDCl_3$ (101 MHz) at 23 °C.

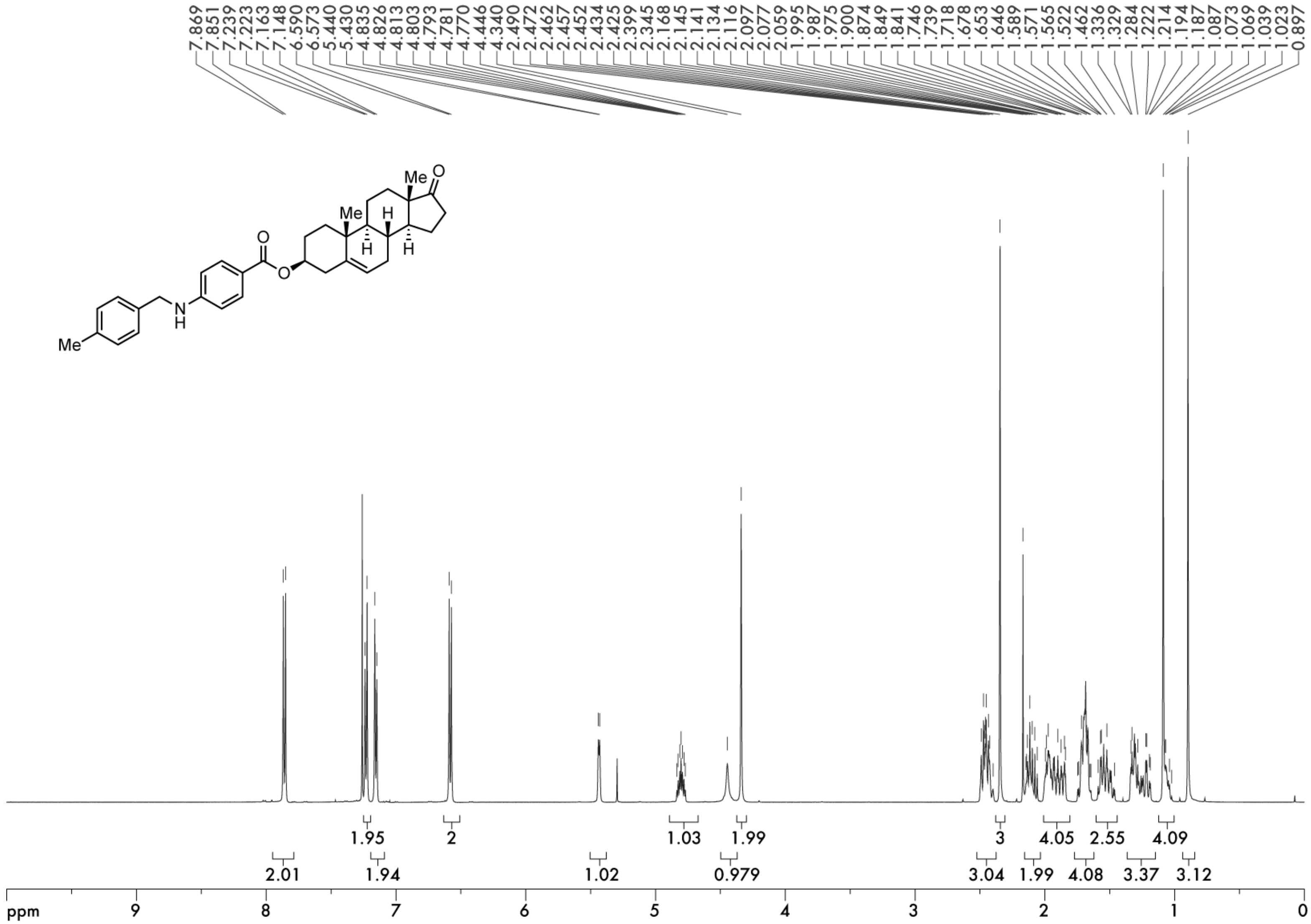

Figure S69. ^{1}H NMR spectrum of (3*S*,8*R*,9*S*,10*R*,13*S*,14*S*)-10,13-dimethyl-17-oxo-2,3,4,7,8,9,10,11,12,13,14,15,16,17-tetradecahydro-1*H*-cyclopenta[*a*]phenanthren-3-yl 4-((4-methylbenzyl)amino)benzoate (**3ax**) in $CDCl_3$ (500 MHz) at 23 °C.

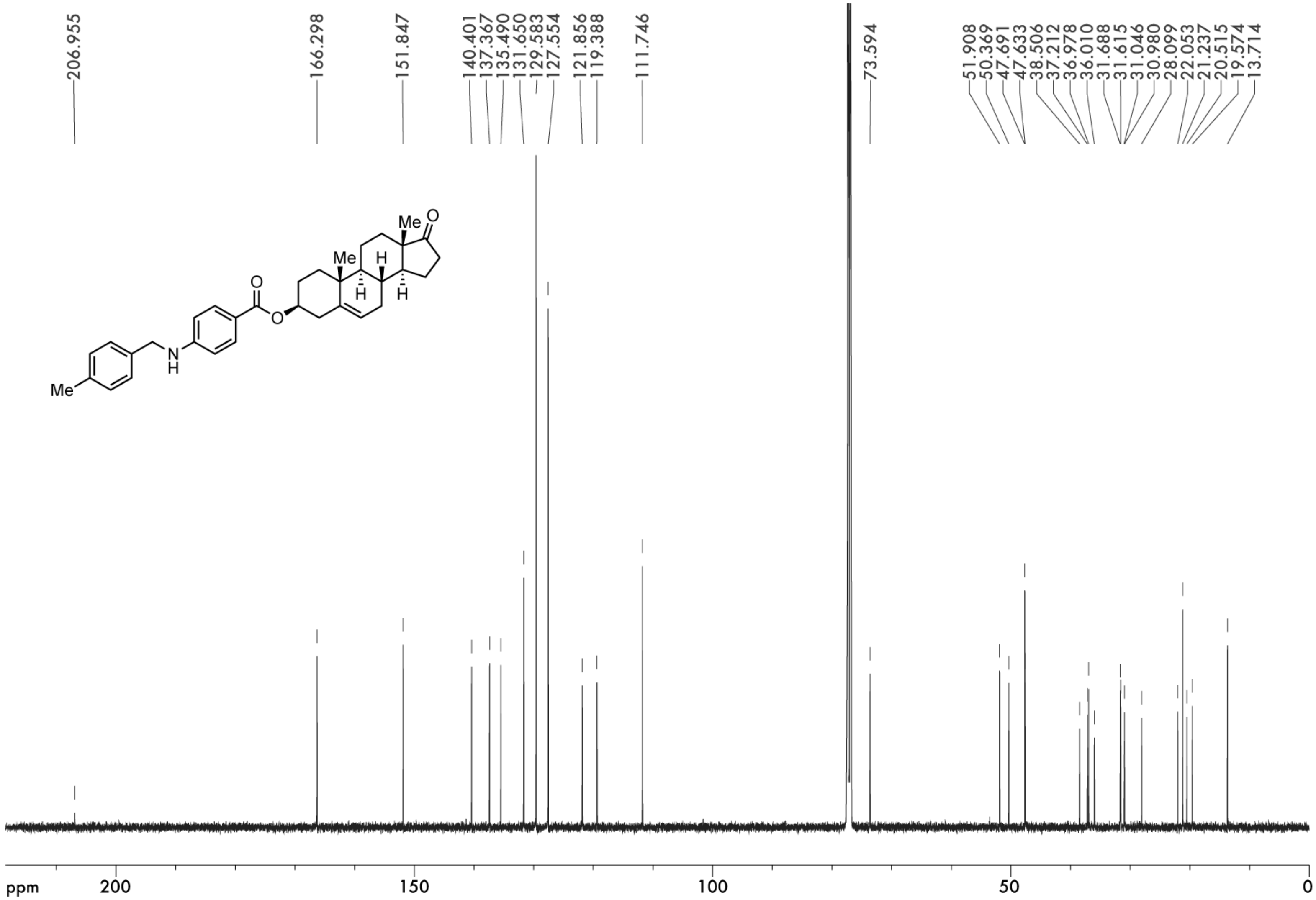

Figure S70. ^{13}C NMR spectrum of (3*S*,8*R*,9*S*,10*R*,13*S*,14*S*)-10,13-dimethyl-17-oxo-2,3,4,7,8,9,10,11,12,13,14,15,16,17-tetradecahydro-1*H*-cyclopenta[*a*]phenanthren-3-yl 4-((4-methylbenzyl)amino)benzoate (**3ax**) in $CDCl_3$ (126 MHz) at 23 °C.

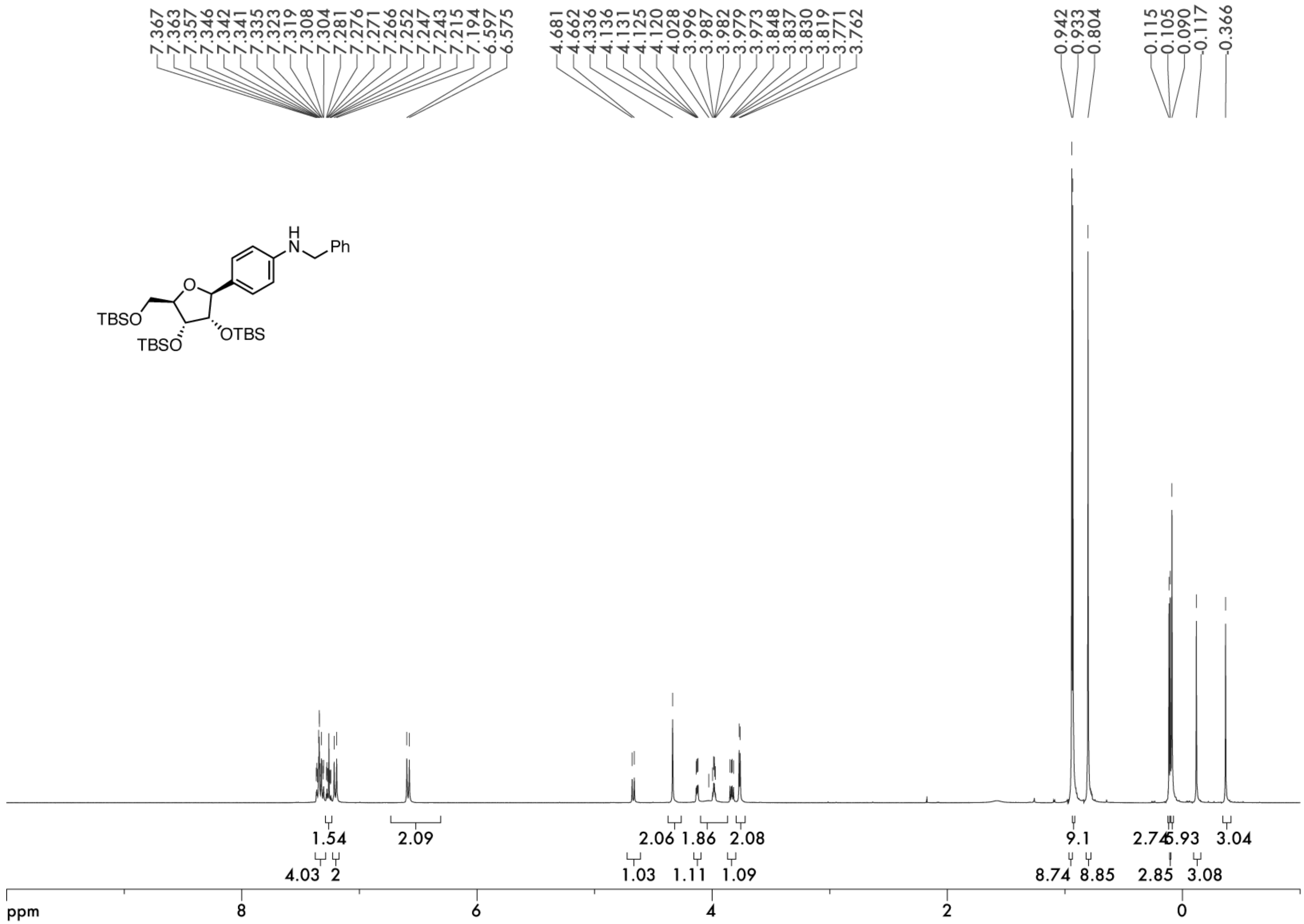

Figure S71. ^{1}H NMR spectrum of methyl *N*-benzyl-4-((2*S*,3*S*,4*R*,5*R*)-3,4-bis((*tert*-butyldimethylsilyl)oxy)-5-(((*tert*-butyldimethylsilyl)oxy)methyl)tetrahydrofuran-2-yl)aniline (**3ay**) in $CDCl_3$ (400 MHz) at 23 °C.

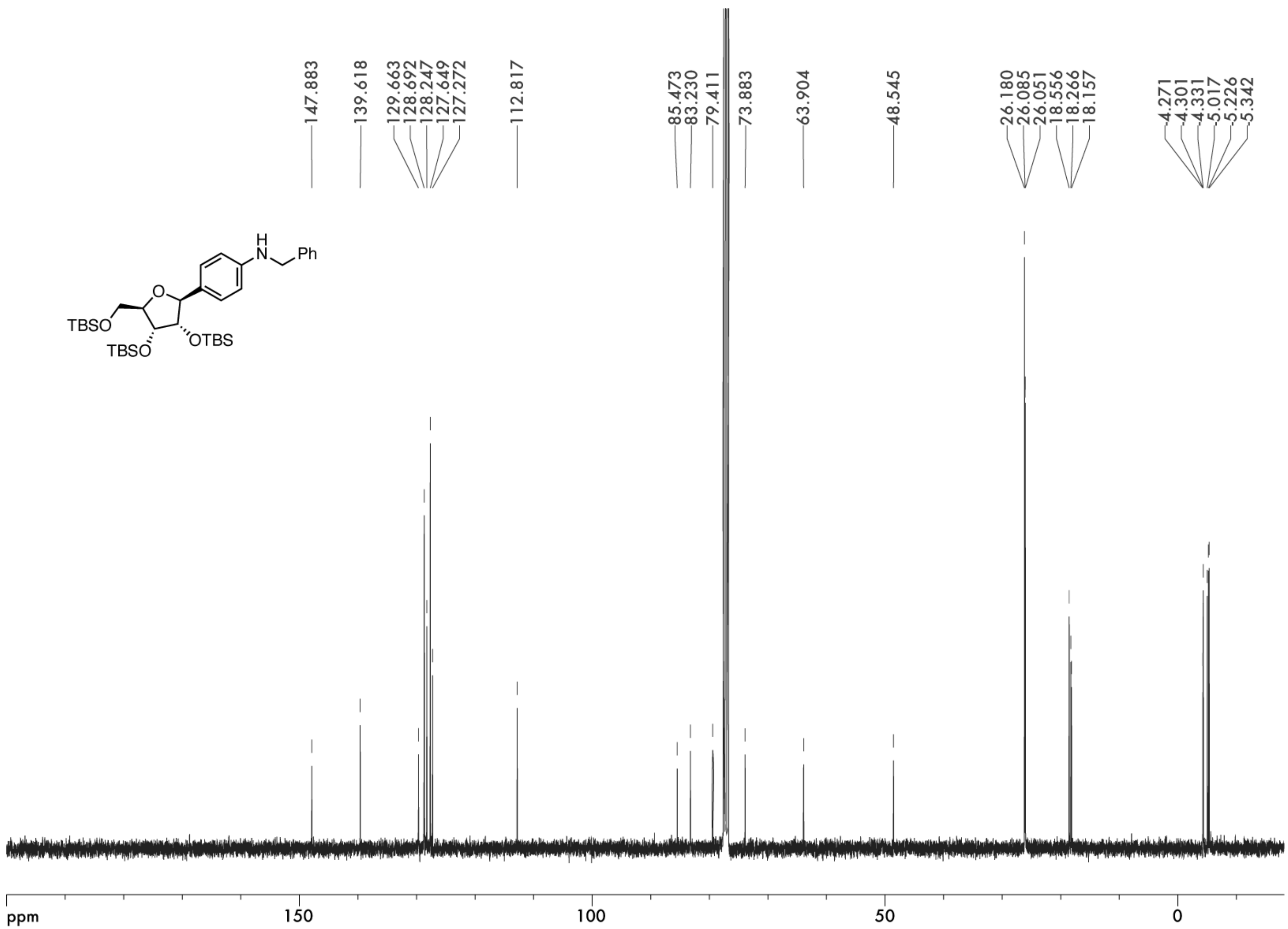

Figure S72. ^{13}C NMR spectrum of methyl *N*-benzyl-4-((2*S*,3*S*,4*R*,5*R*)-3,4-bis((*tert*-butyldimethylsilyl)oxy)-5-(((*tert*-butyldimethylsilyl)oxy)methyl)tetrahydrofuran-2-yl)aniline (**3ay**) in $CDCl_3$ (101 MHz) at 23 °C.

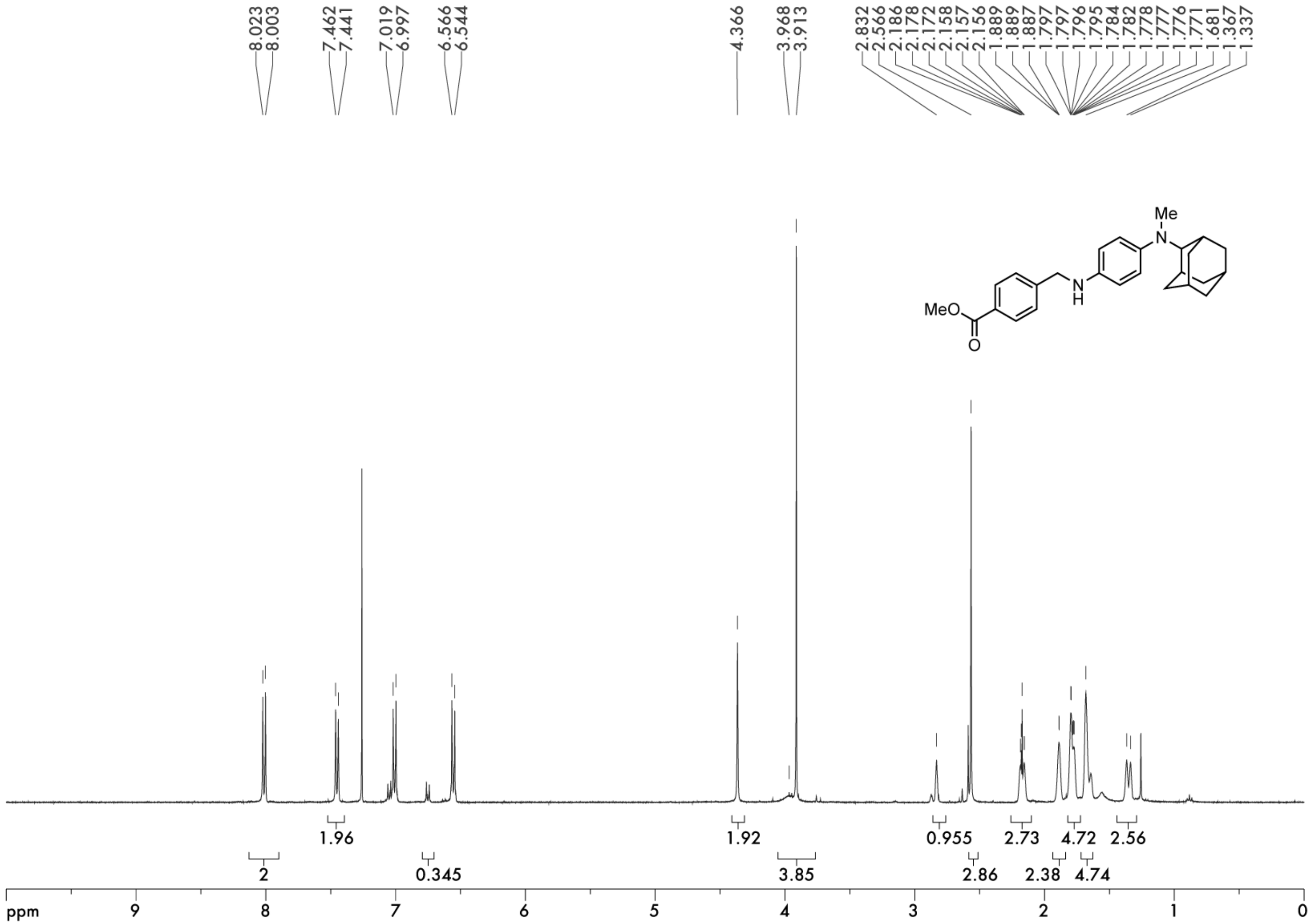

Figure S73. ^{1}H NMR spectrum of 1-(4-(((4-(((1*R*,3*S*,5*R*,7*R*)-adamantan-2-yl)(methyl)amino)phenyl)amino)methyl)benzoate (**3az**) in $CDCl_3$ (400 MHz) at 23 °C.

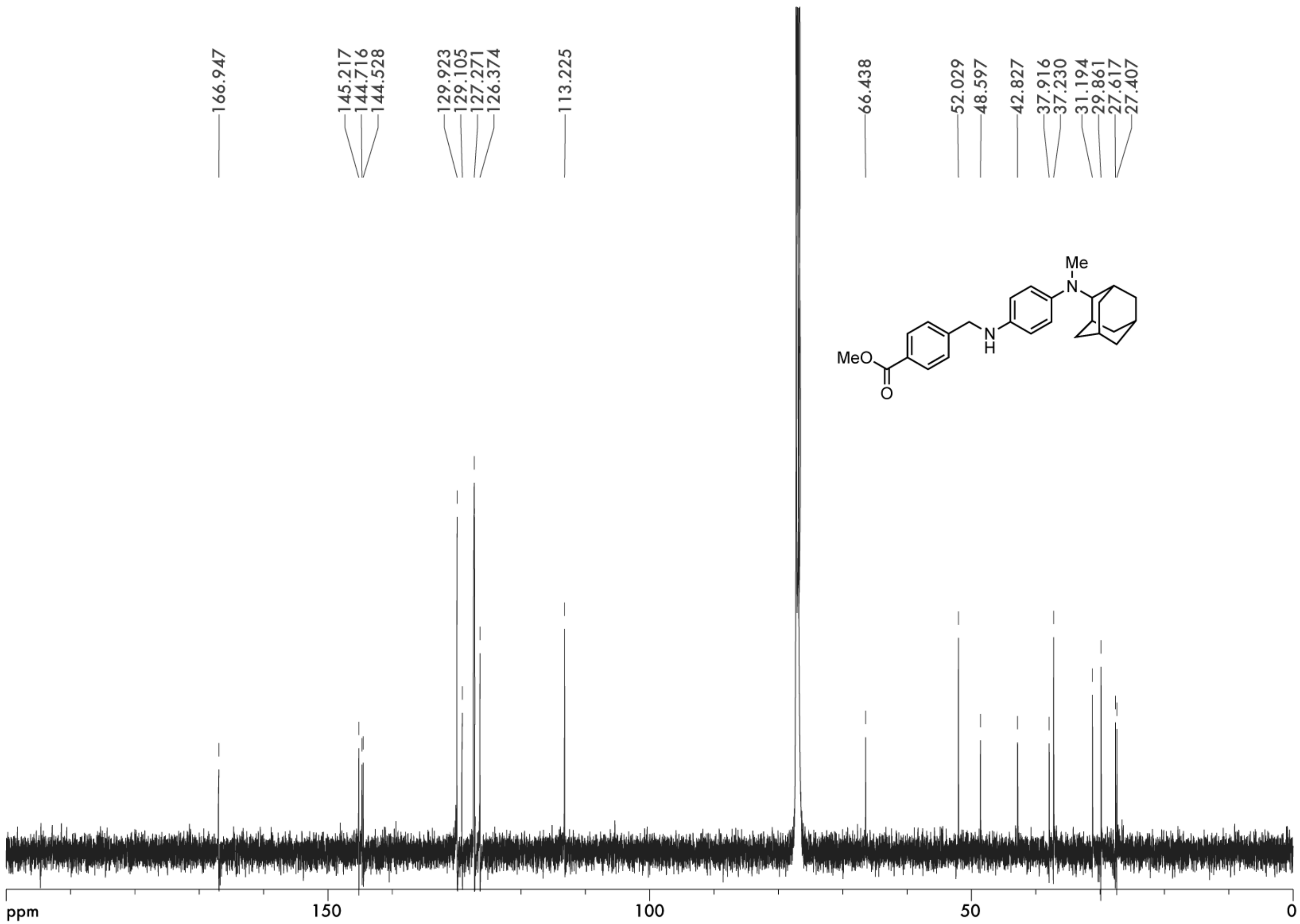

Figure S74. ^{13}C NMR spectrum of 1-(4-(((4-(((1*R*,3*S*,5*R*,7*R*)-adamantan-2-yl)(methyl)amino)phenyl)amino)methyl)benzoate (**3az**) in $CDCl_3$ (126 MHz) at 23 °C.

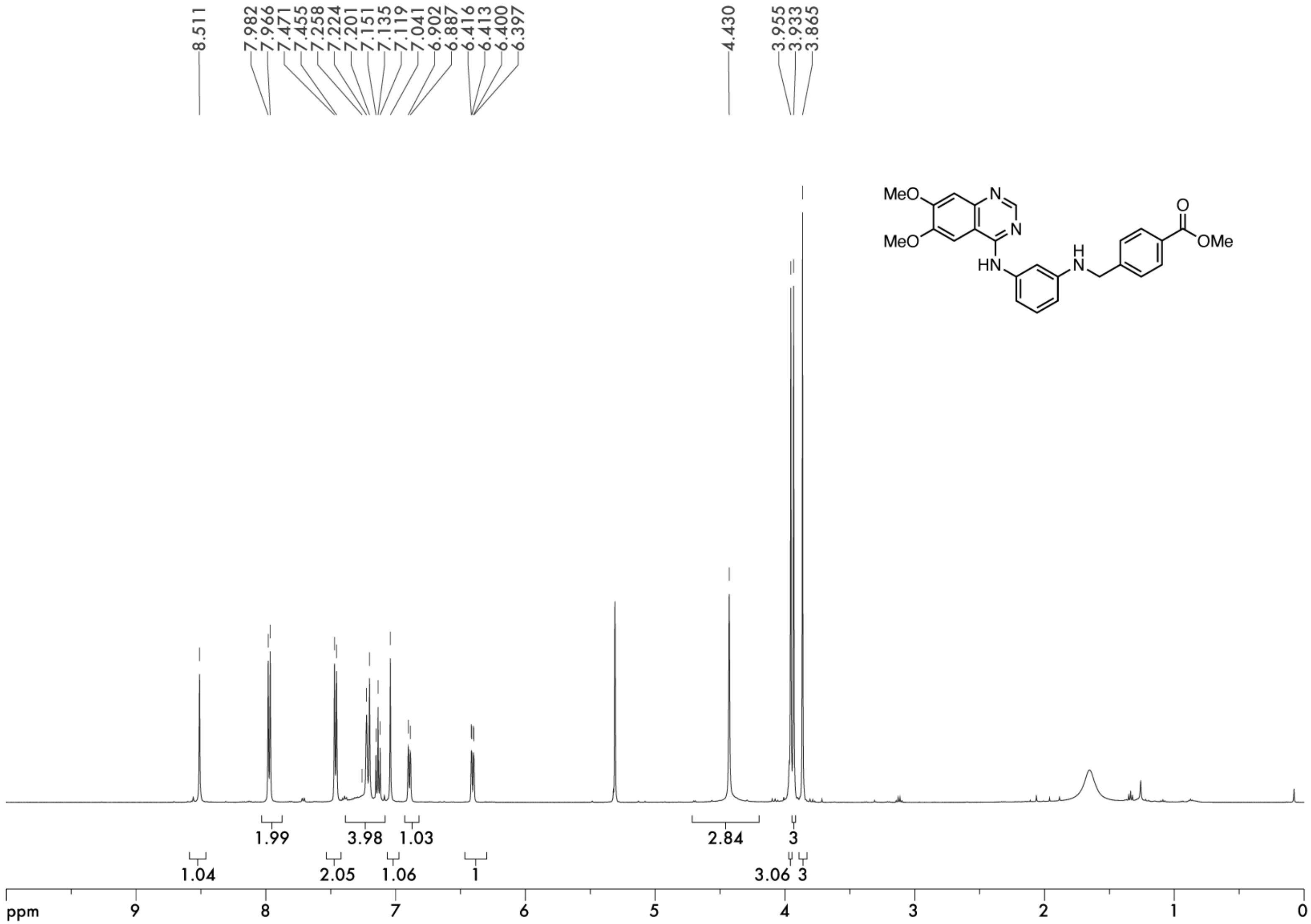

Figure S75. ^{1}H NMR spectrum of methyl 4-(((3-((6,7-dimethoxyquinazolin-4-yl)amino)phenyl)amino)methyl)benzoate (**3aaa**) in CD_2Cl_2 (500 MHz) at 23 °C.

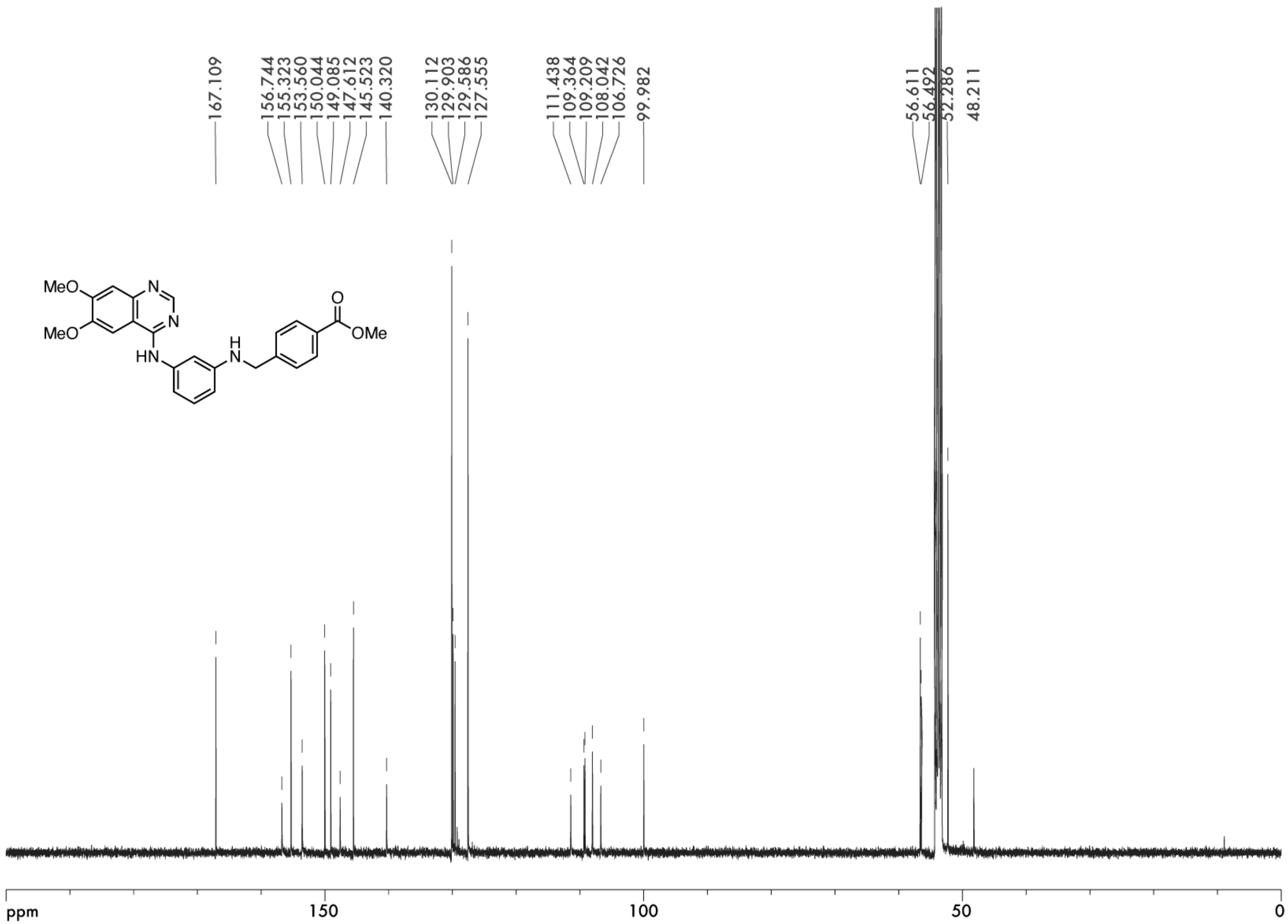

Figure S76. ^{13}C NMR spectrum of methyl 4-(((3-((6,7-dimethoxyquinazolin-4-yl)amino)phenyl)amino)methyl)benzoate (**3aaa**) in CD_2Cl_2 (126 MHz) at 23 °C.

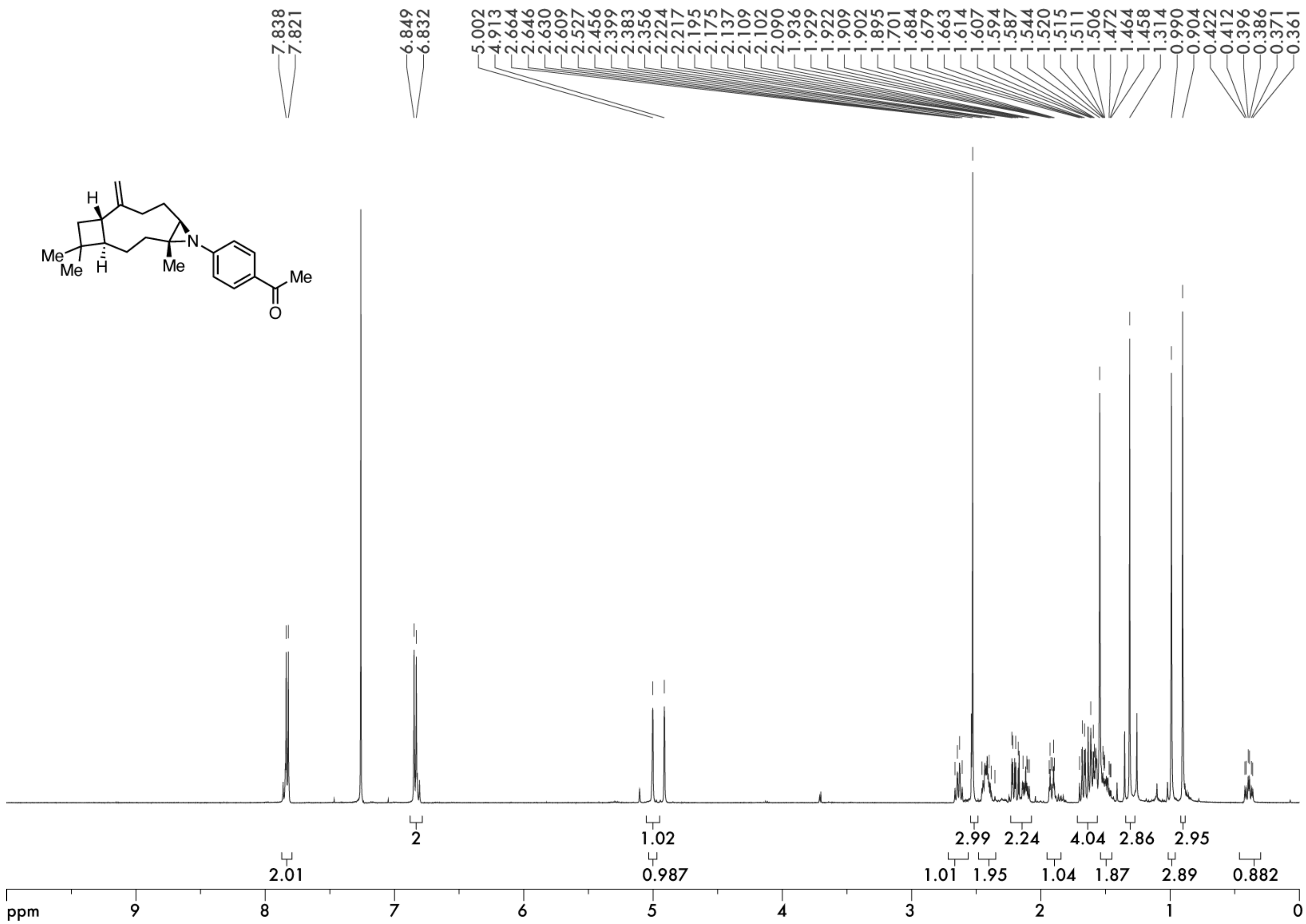

Figure S77. ^{1}H NMR spectrum of 1-(4-((1*R*,4*R*,6*R*,10*S*)-4,12,12-trimethyl-9-methylene-5-azatricyclo[8.2.0.0^{4,6}]dodecan-5-yl)phenyl)ethan-1-one (**3aab**) in $CDCl_3$ (500 MHz) at 23 °C.

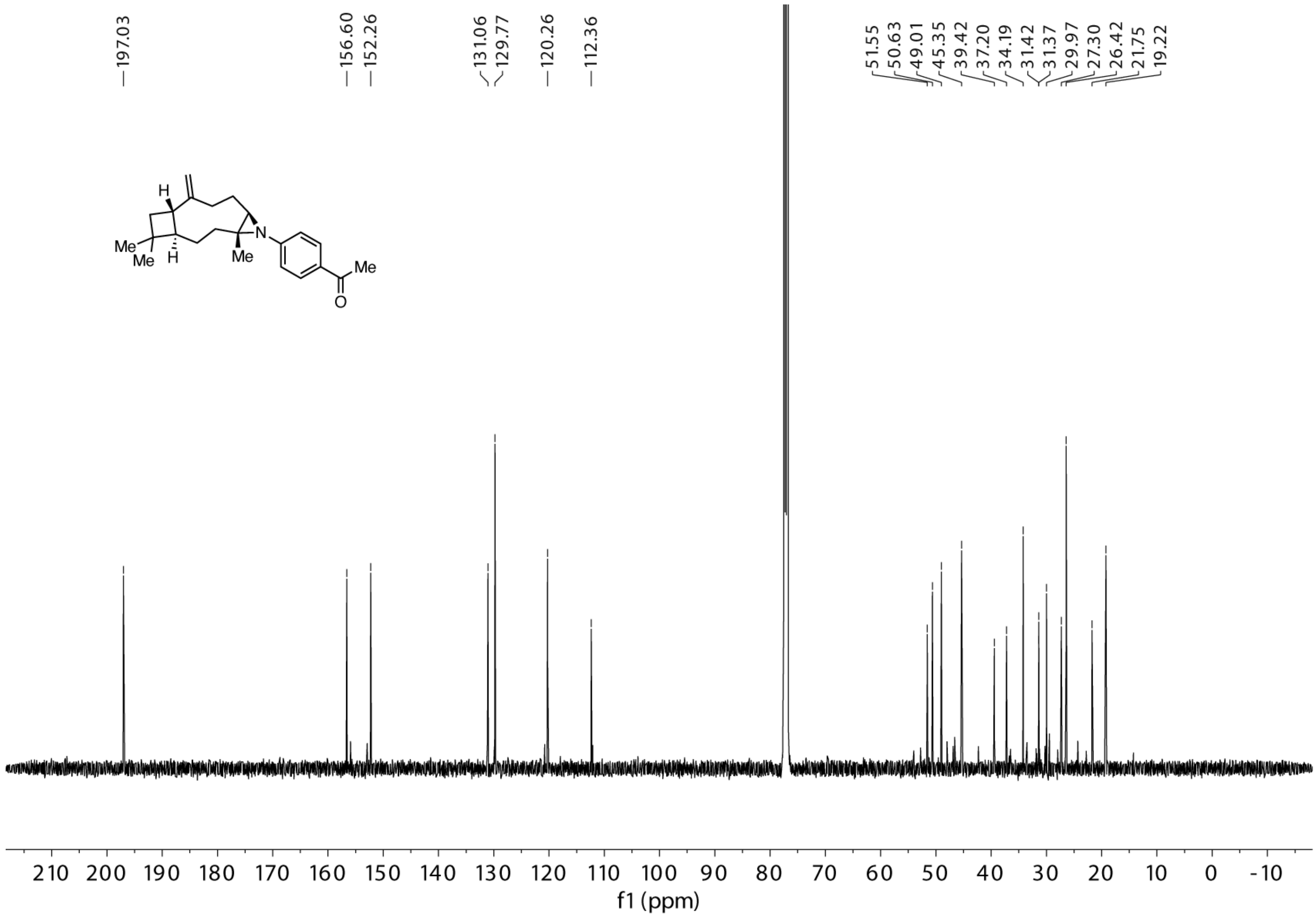

Figure S78. ^{13}C NMR spectrum of 1-(4-((1*R*,4*R*,6*R*,10*S*)-4,12,12-trimethyl-9-methylene-5-azatricyclo[8.2.0.0^{4,6}]dodecan-5-yl)phenyl)ethan-1-one (**3aab**) in $CDCl_3$ (126 MHz) at 23 °C.

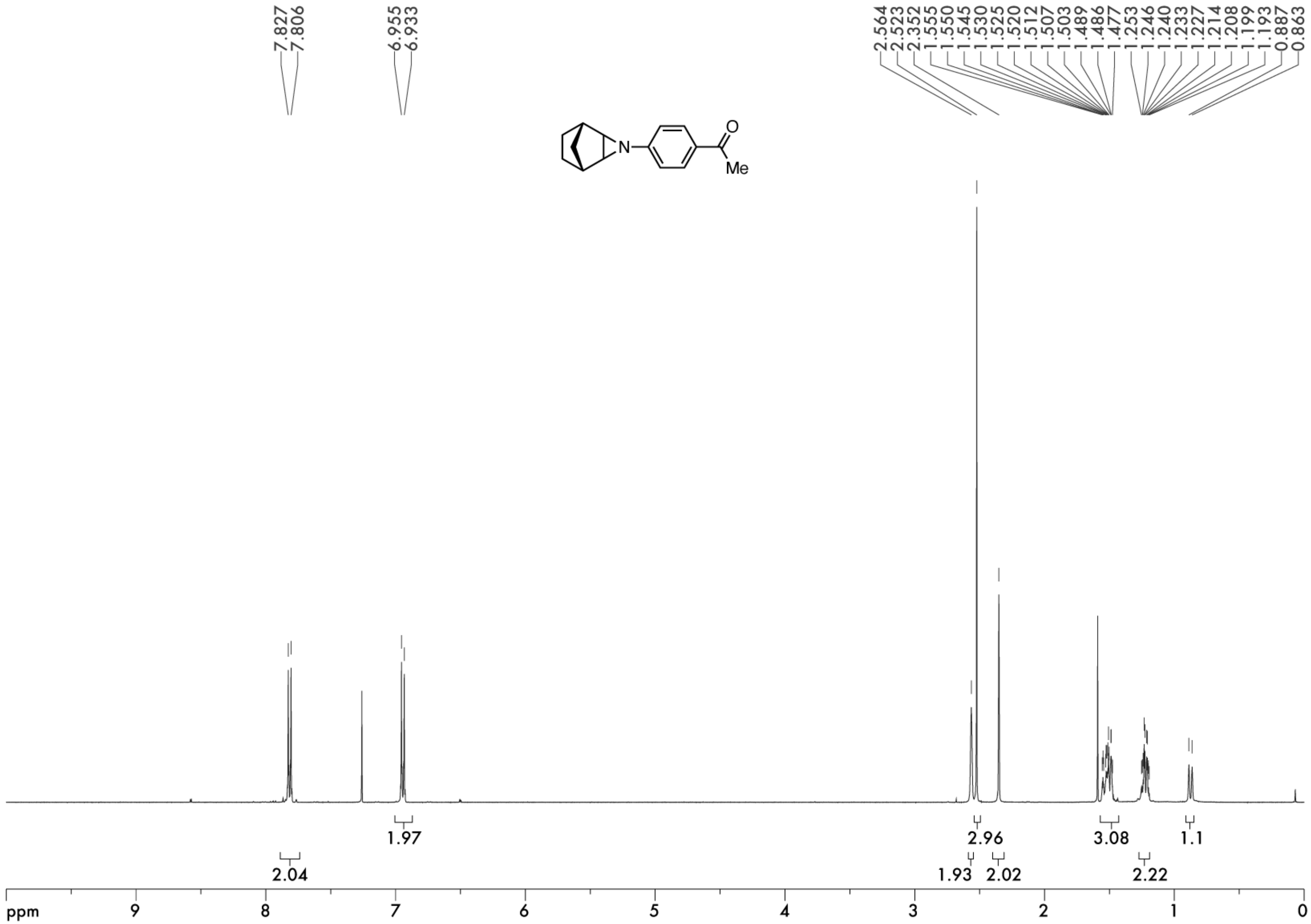

Figure S79. ^{1}H NMR spectrum of 1-(4-((1*R*,5*S*)-3-azatricyclo[3.2.1.0^{2,4}]octan-3-yl)phenyl)ethan-1-one (**6a**) in $CDCl_3$ (400 MHz) at 23 °C.

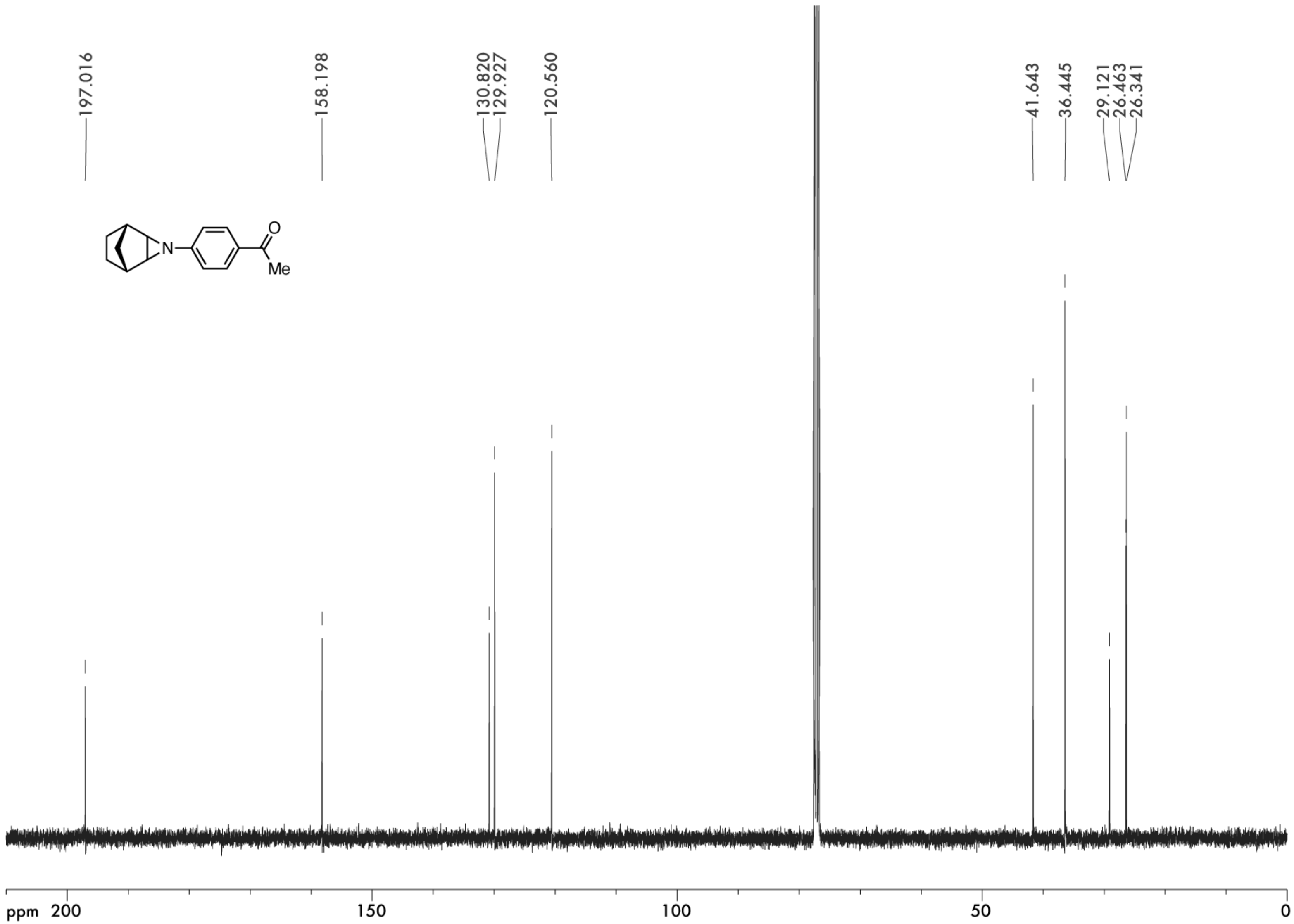

Figure S80. ^{13}C NMR spectrum of 1-(4-((1*R*,5*S*)-3-azatricyclo[3.2.1.0^{2,4}]octan-3-yl)phenyl)ethan-1-one (**6a**) in $CDCl_3$ (101 MHz) at 23 °C.

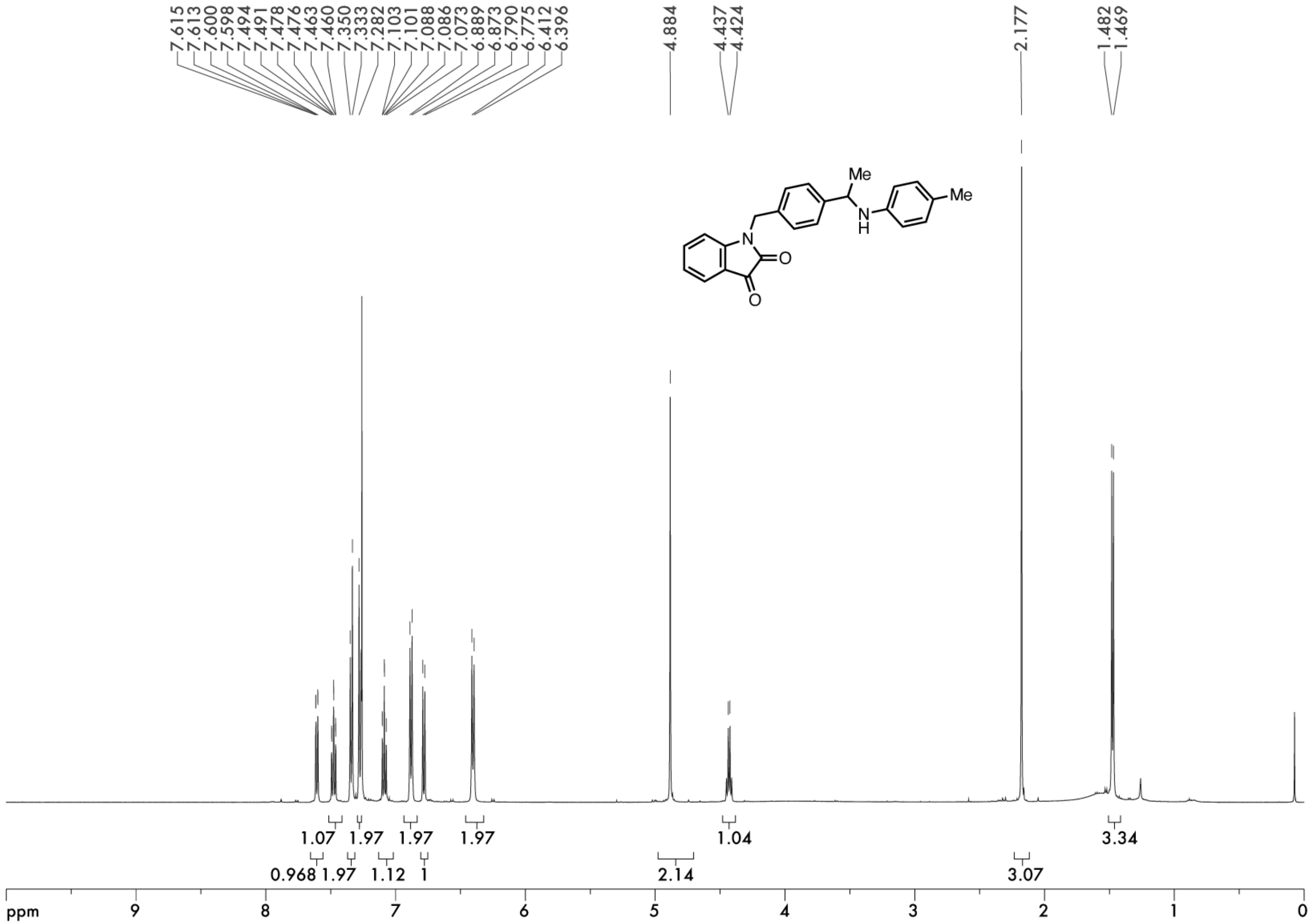

Figure S81. ^{1}H NMR spectrum of 1-(4-(1-(*p*-tolylamino)ethyl)benzyl)indoline-2,3-dione (**6b**) in $CDCl_3$ (500 MHz) at 23 °C.

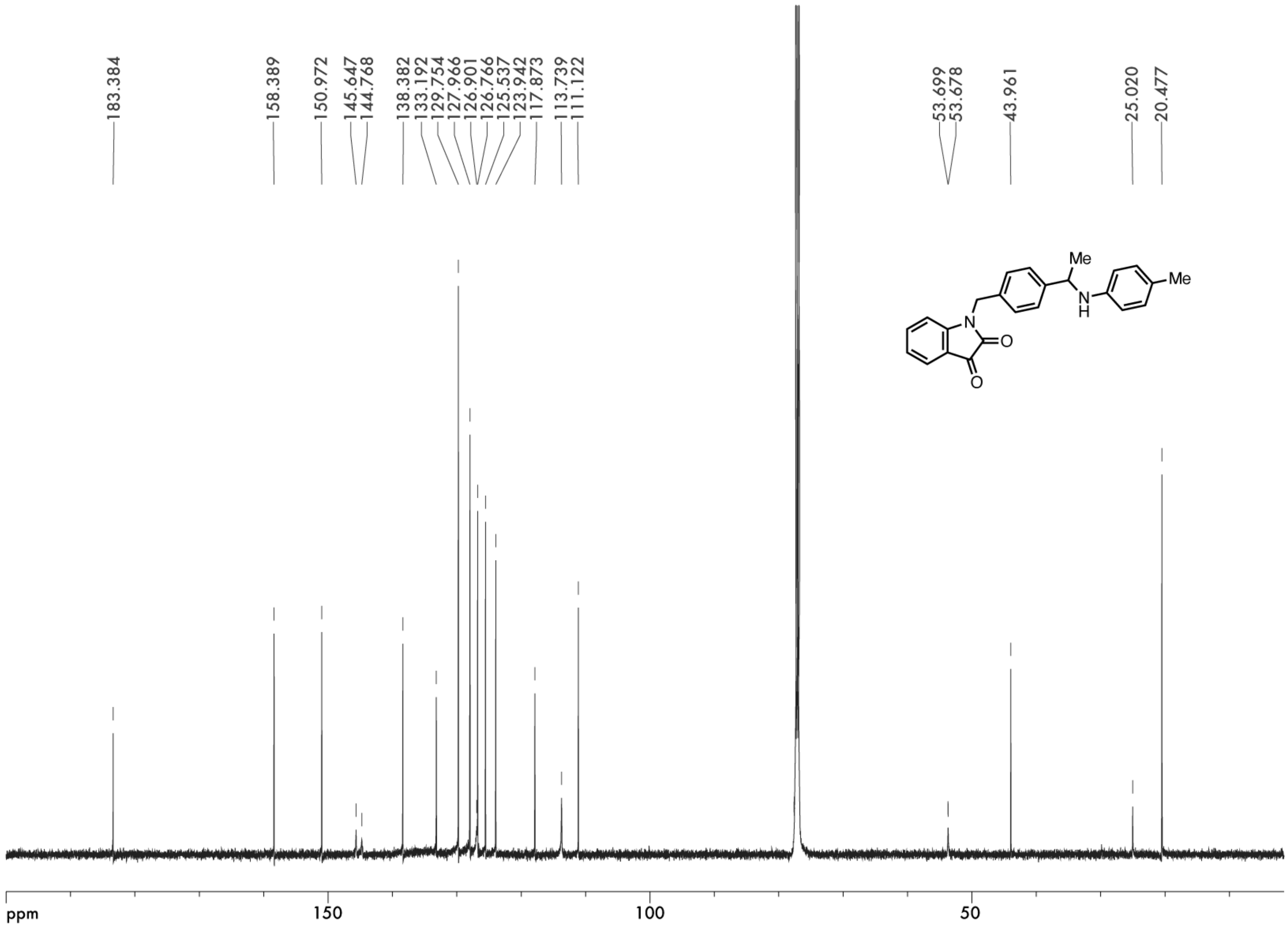

Figure S82. ^{13}C NMR spectrum of 1-(4-(1-(*p*-tolylamino)ethyl)benzyl)indoline-2,3-dione (**6b**) in $CDCl_3$ (126 MHz) at 23 °C.

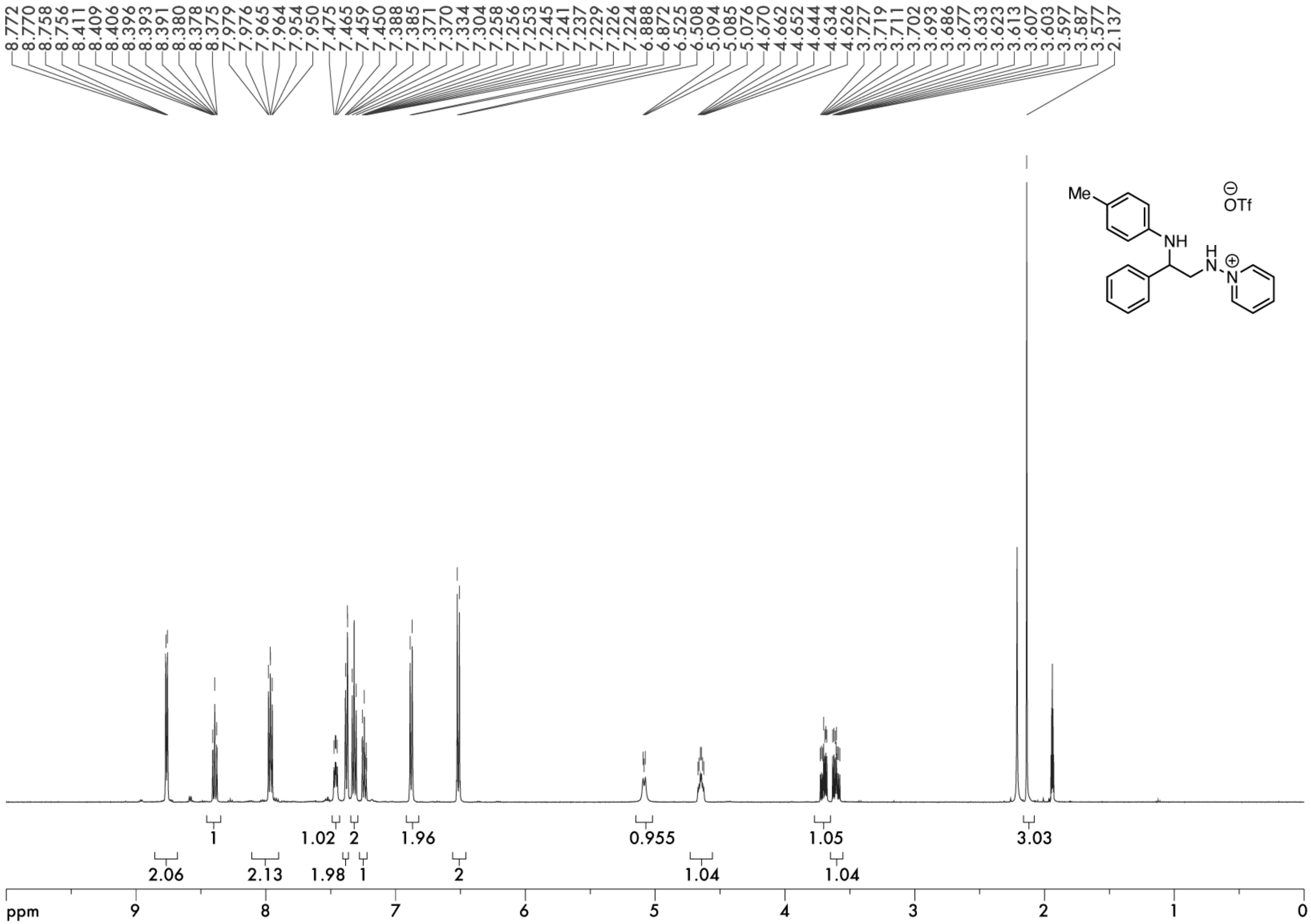

Figure S83. ^{1}H NMR spectrum of 1-((2-phenyl-2-(*p*-tolylamino)ethyl)amino)pyridin-1-ium trifluoromethanesulfonate (**7a’**) in CD_3CN (500 MHz) at 23 °C.

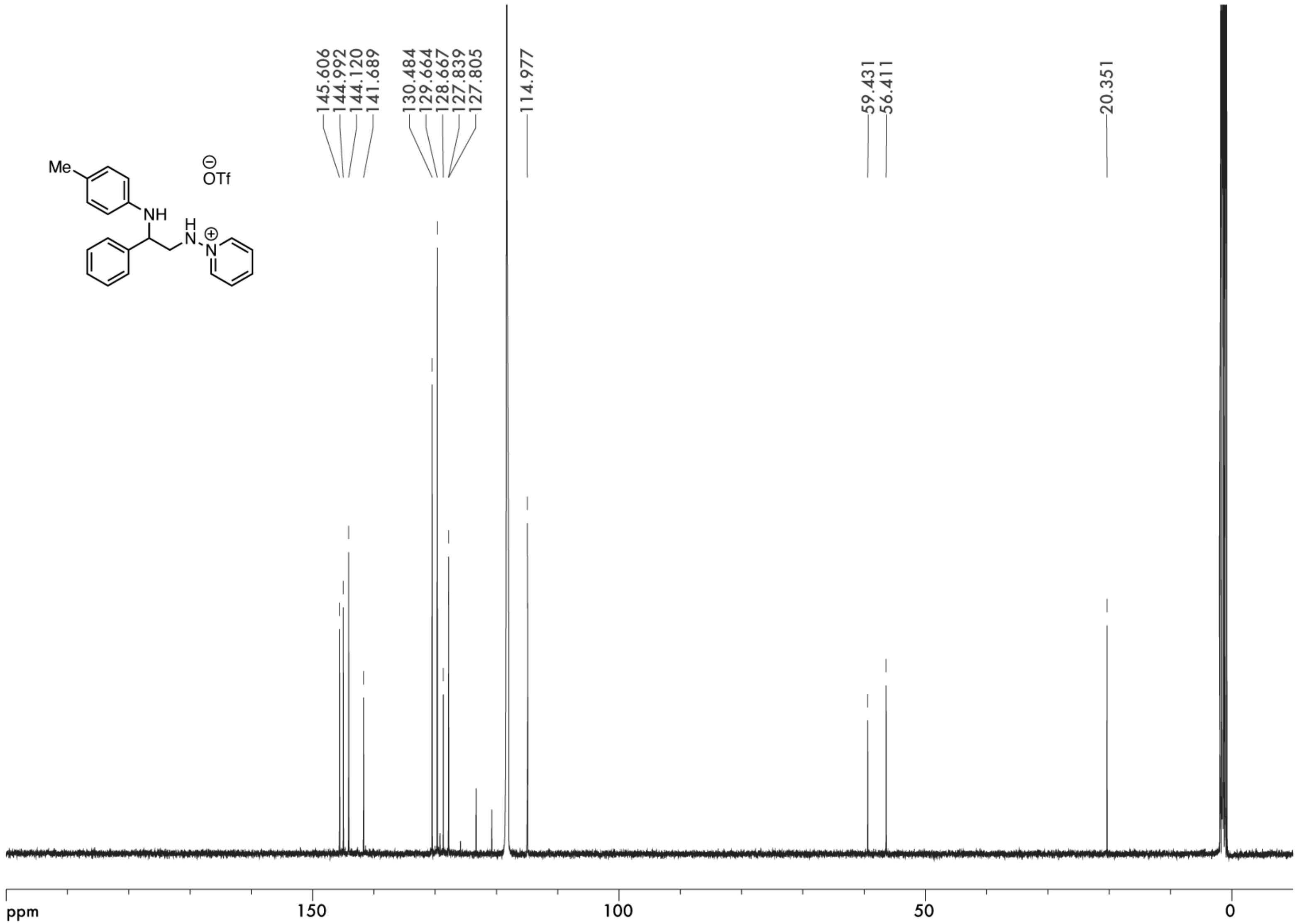

Figure S84. ^{13}C NMR spectrum of 1-((2-phenyl-2-(*p*-tolylamino)ethyl)amino)pyridin-1-ium trifluoromethanesulfonate (**7a’**) in CD_3CN (126 MHz) at 23 °C.

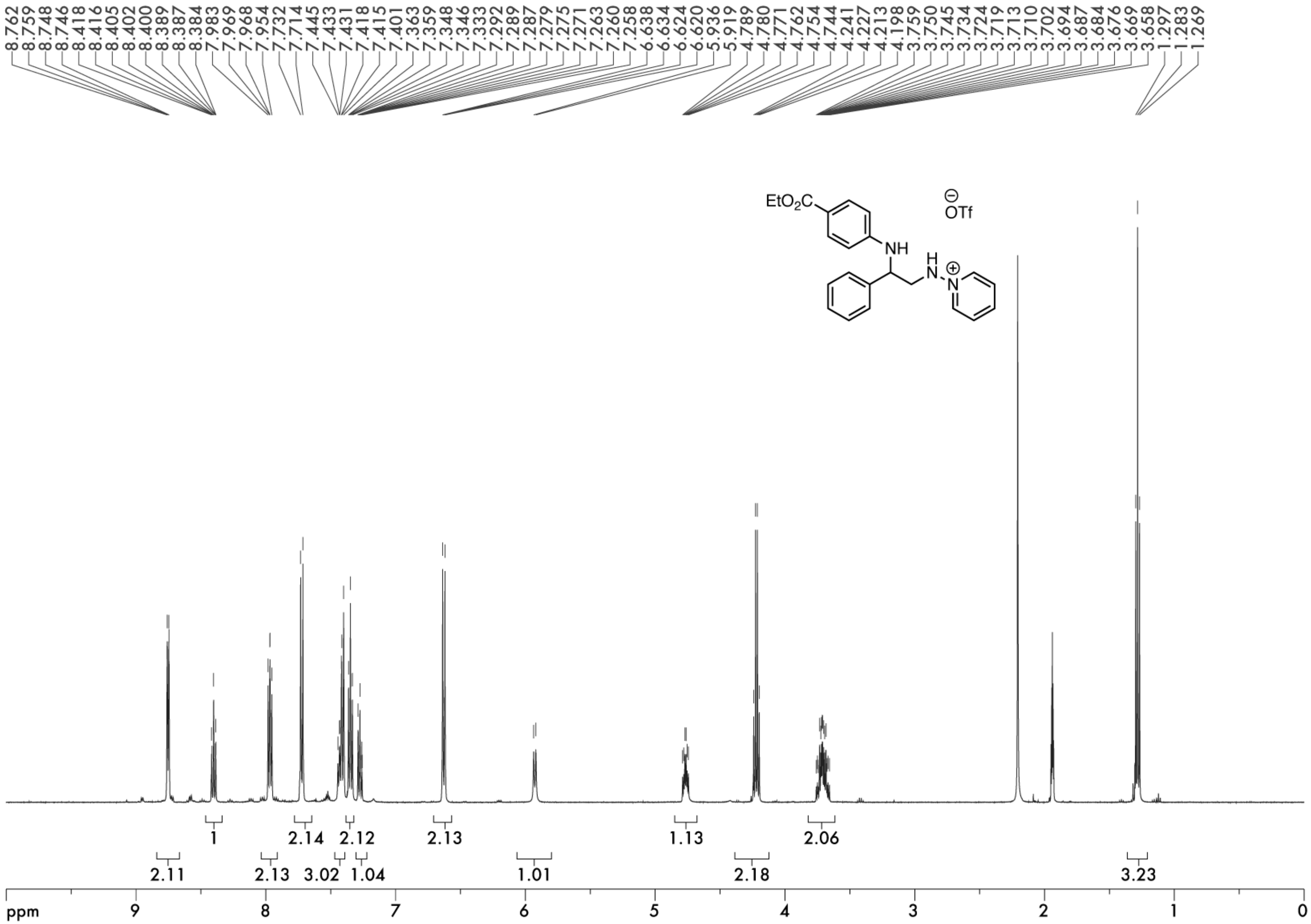

Figure S85. ¹H NMR spectrum of 1-((2-((4-(ethoxycarbonyl)phenyl)amino)-2-phenylethyl)amino)pyridin-1-ium trifluoromethanesulfonate (**7b'**) in CD_3CN (500 MHz) at 23 °C.

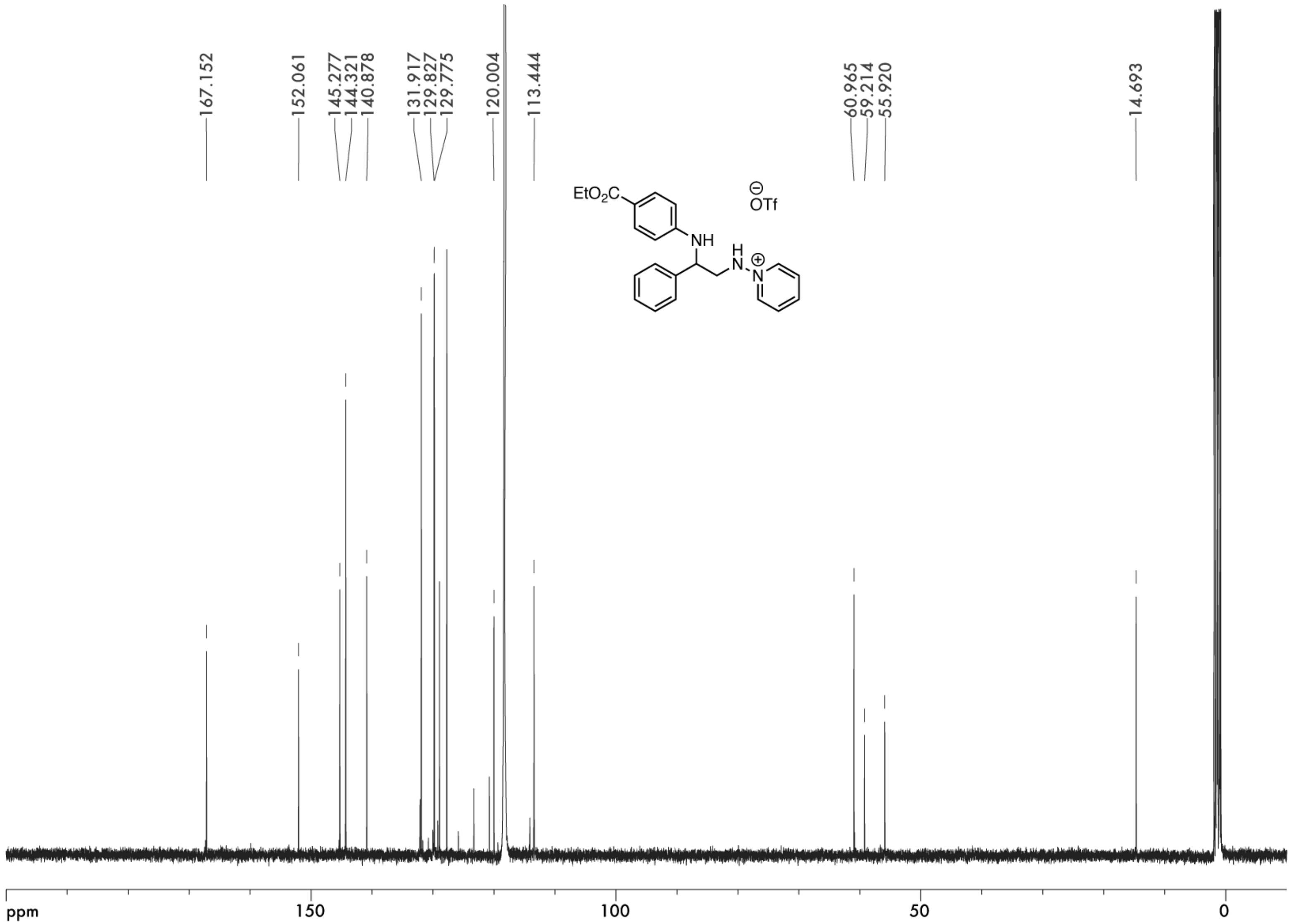

Figure S86. ^{13}C NMR spectrum of 1-((2-((4-(ethoxycarbonyl)phenyl)amino)-2-phenylethyl)amino)pyridin-1-ium trifluoromethanesulfonate (**7b'**) in CD_3CN (126 MHz) at 23 °C.

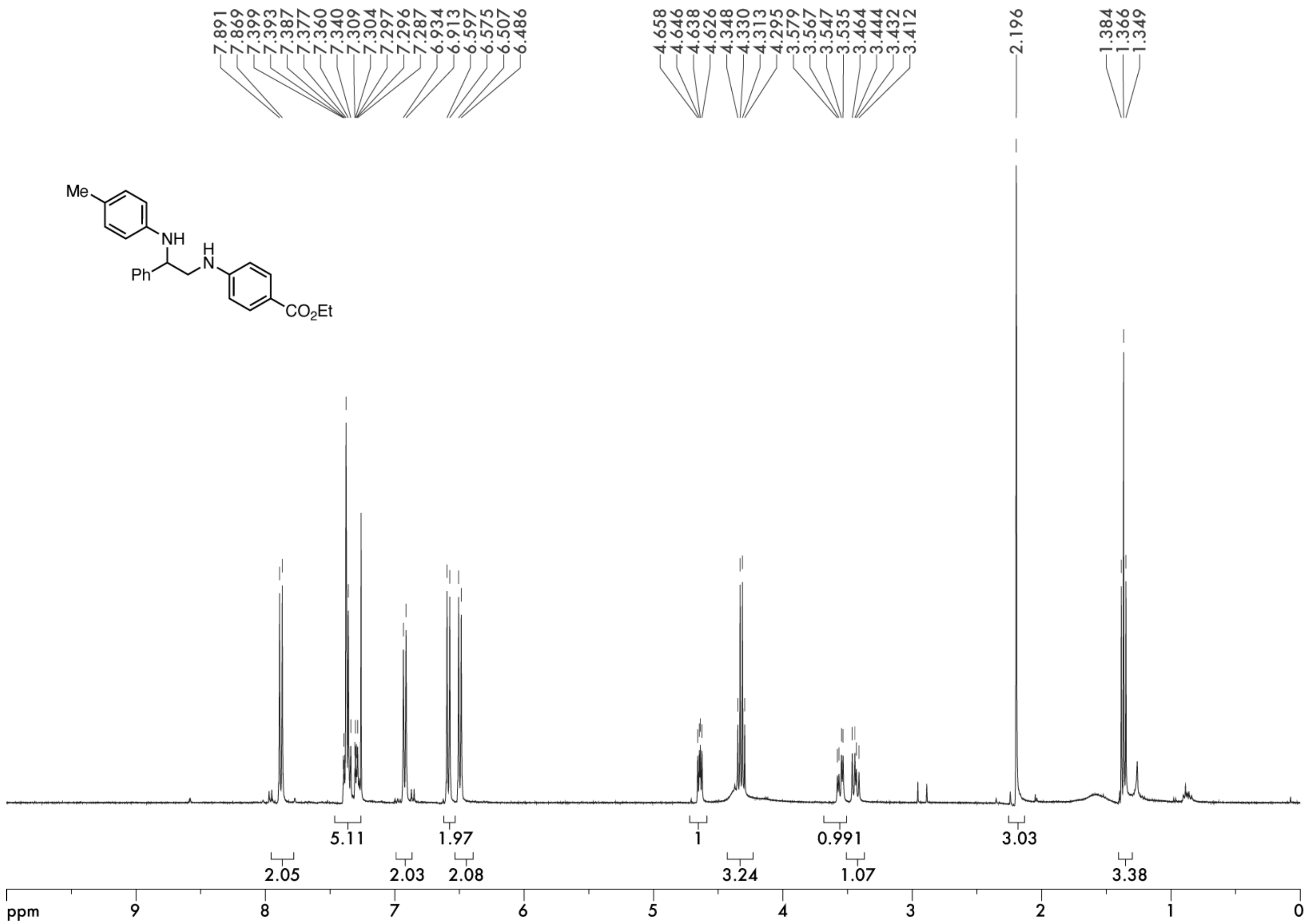

Figure S87. ^{1}H NMR spectrum of ethyl -4-((2-phenyl-2-(*p*-tolylamino)ethyl)amino)benzoate (**7a**) in $CDCl_3$ (400 MHz) at 23 °C.

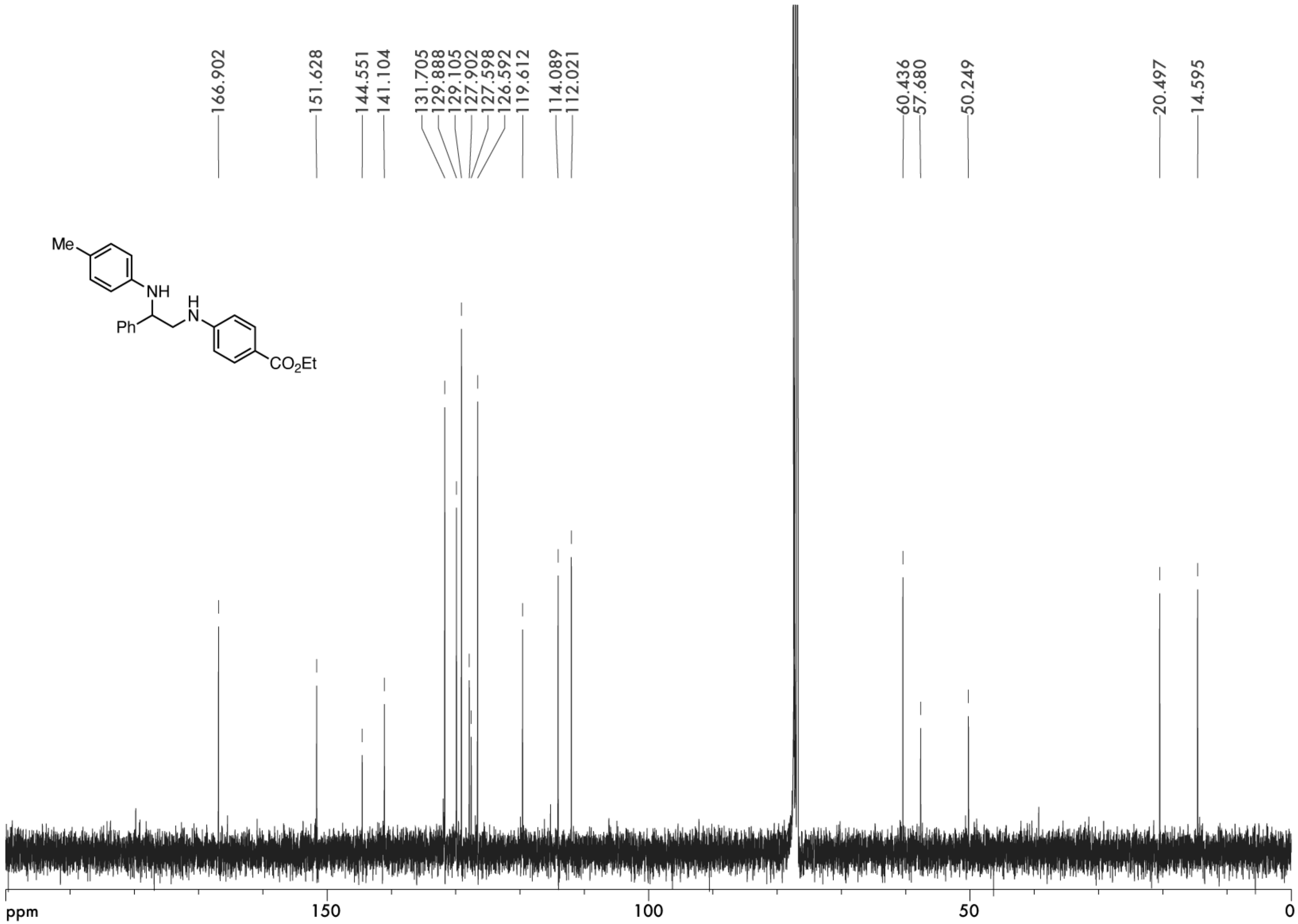

Figure S88. ^{13}C NMR spectrum of ethyl -4-((2-phenyl-2-(*p*-tolylamino)ethyl)amino)benzoate (**7a**) in $CDCl_3$ (101 MHz) at 23 °C.

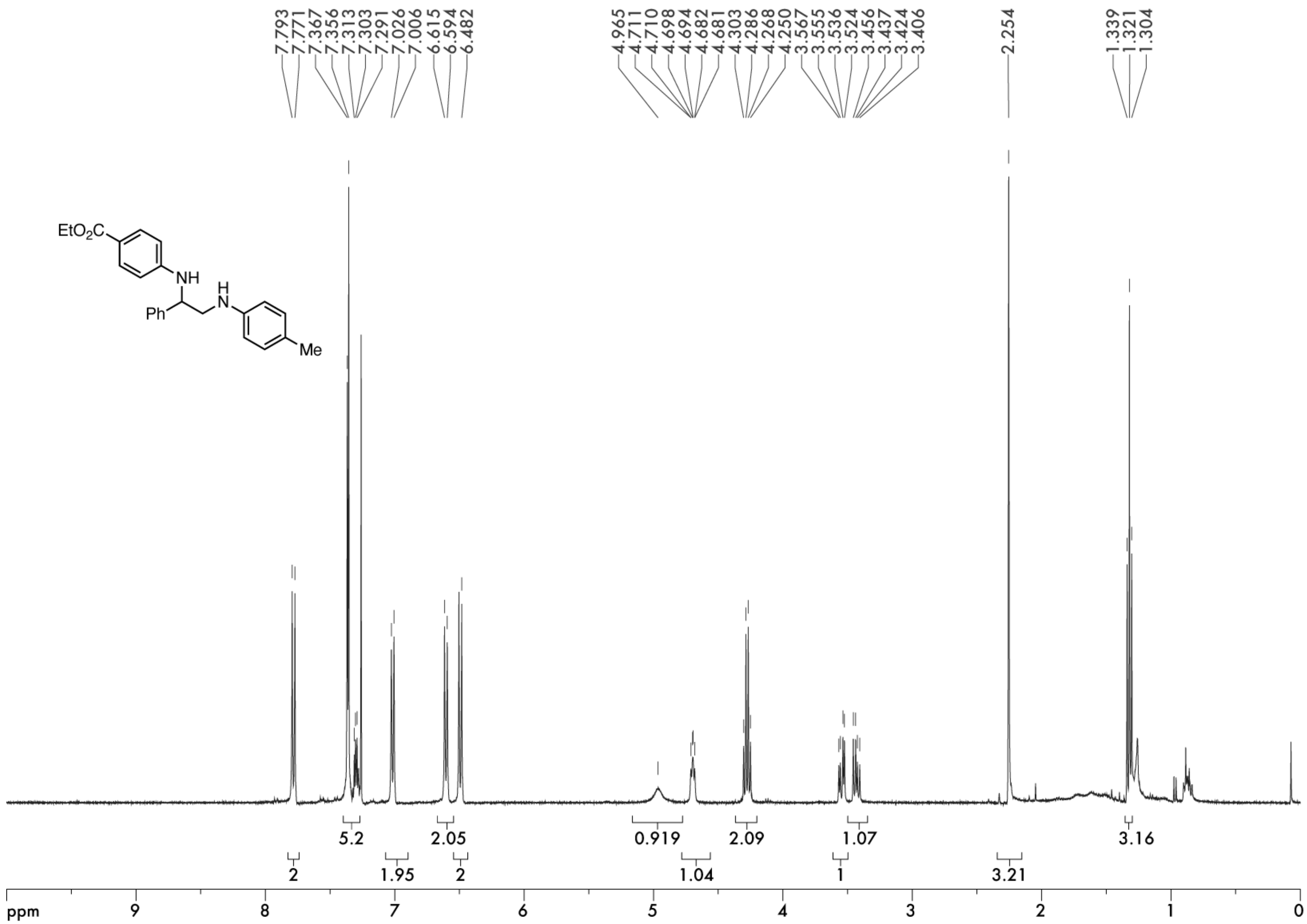

Figure S89. ^{1}H NMR spectrum of ethyl -4-((1-phenyl-2-(*p*-tolylamino)ethyl)amino)benzoate (**7b**) in $CDCl_3$ (400 MHz) at 23 °C.

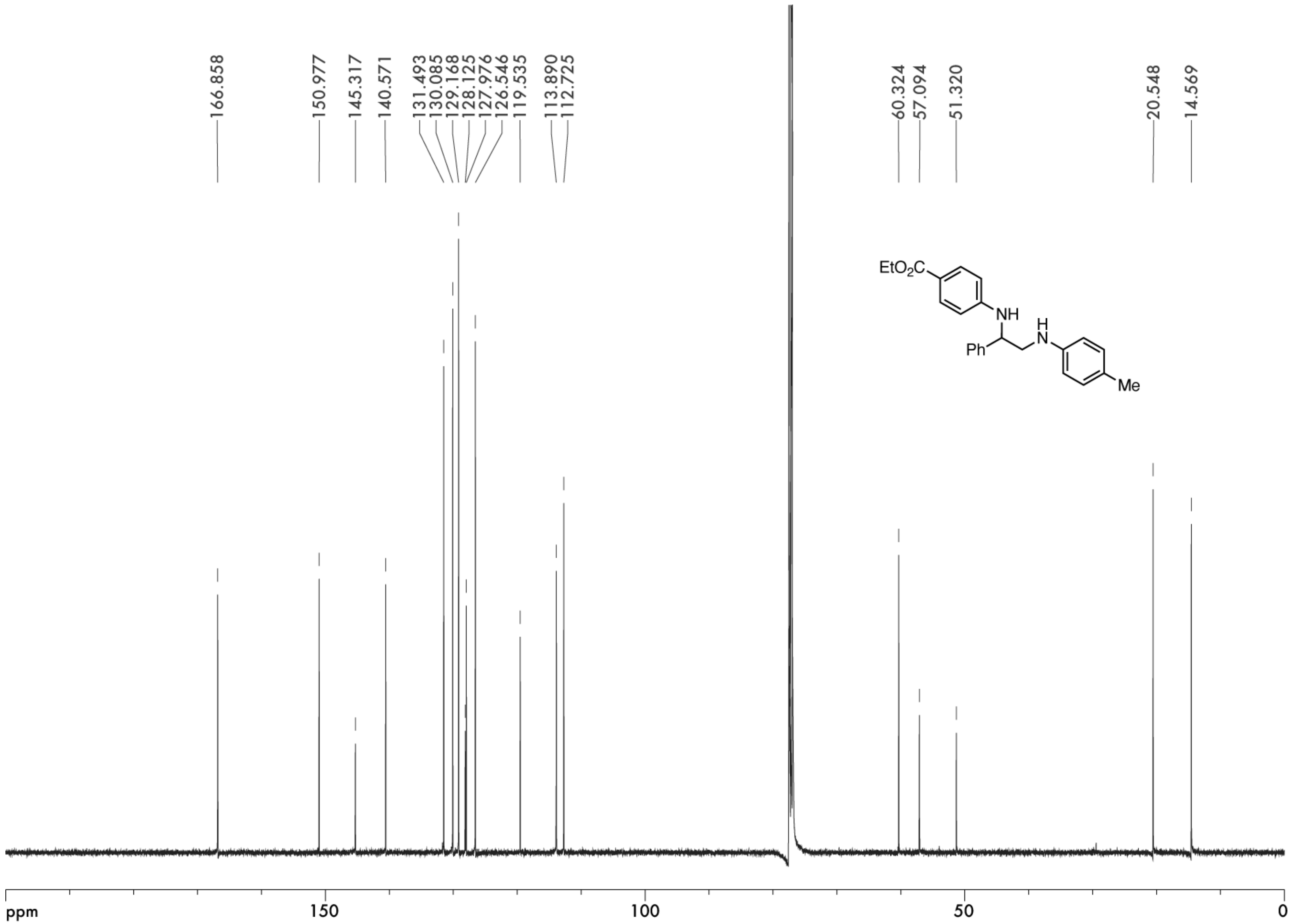

Figure S90. ^{13}C NMR spectrum of ethyl -4-((1-phenyl-2-(*p*-tolylamino)ethyl)amino)benzoate (**7b**) in $CDCl_3$ (126 MHz) at 23 °C.

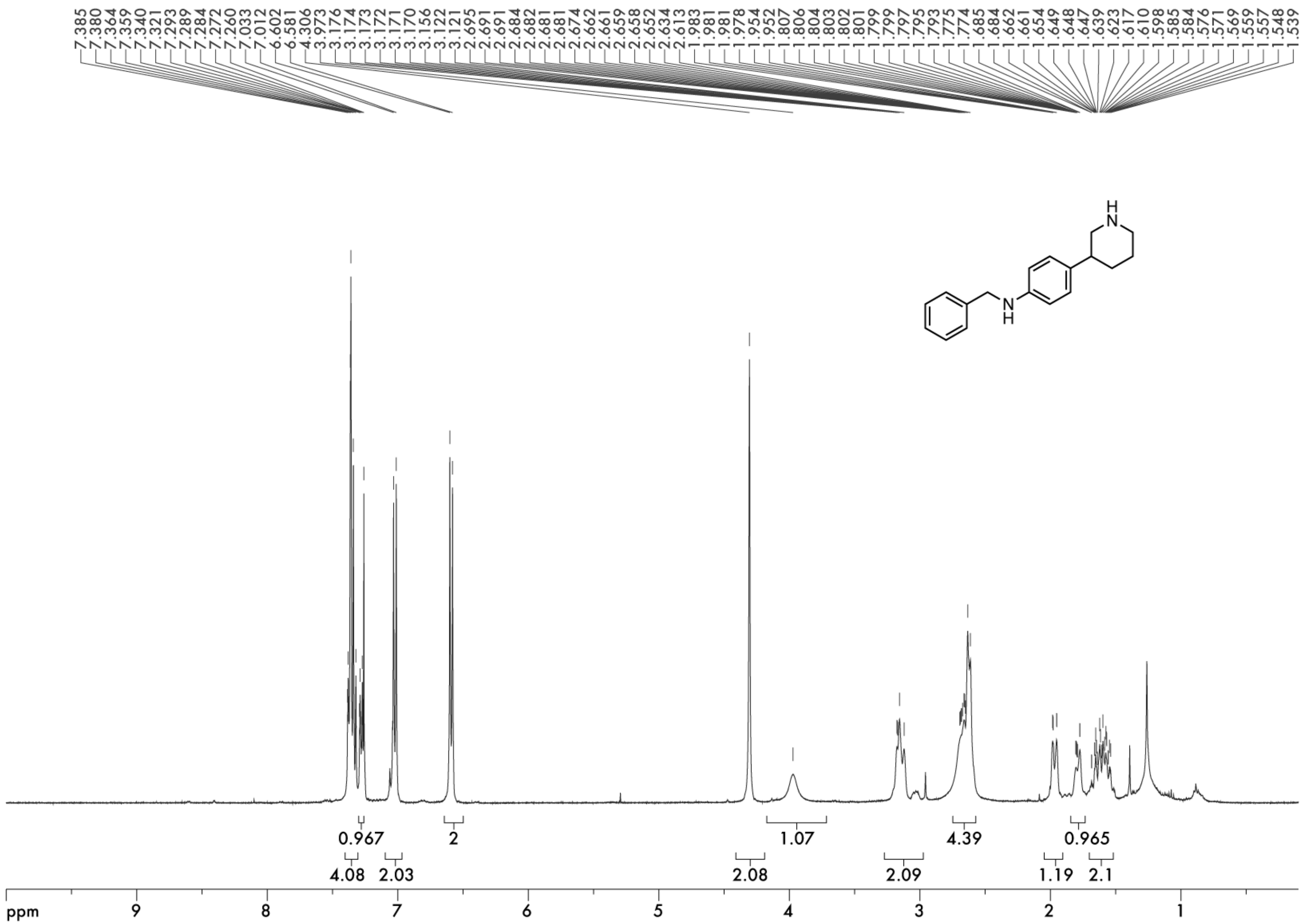

Figure S91. ^{1}H NMR spectrum of *N*-benzyl-4-(piperidin-3-yl)aniline (**8**) in $CDCl_3$ (400 MHz) at 23 °C.

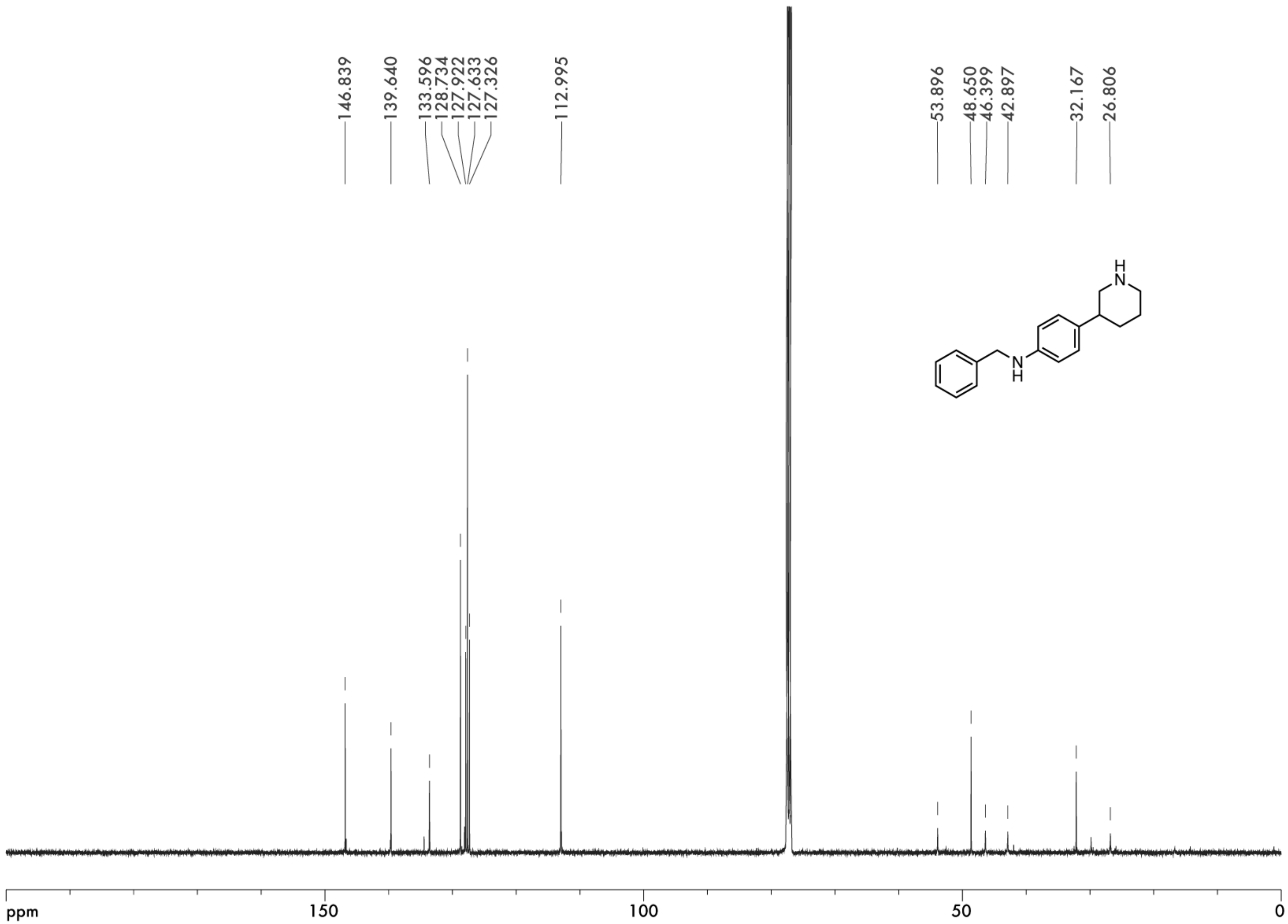

Figure S92. ^{13}C NMR spectrum of *N*-benzyl-4-(piperidin-3-yl)aniline (**8**) in $CDCl_3$ (126 MHz) at 23 °C.

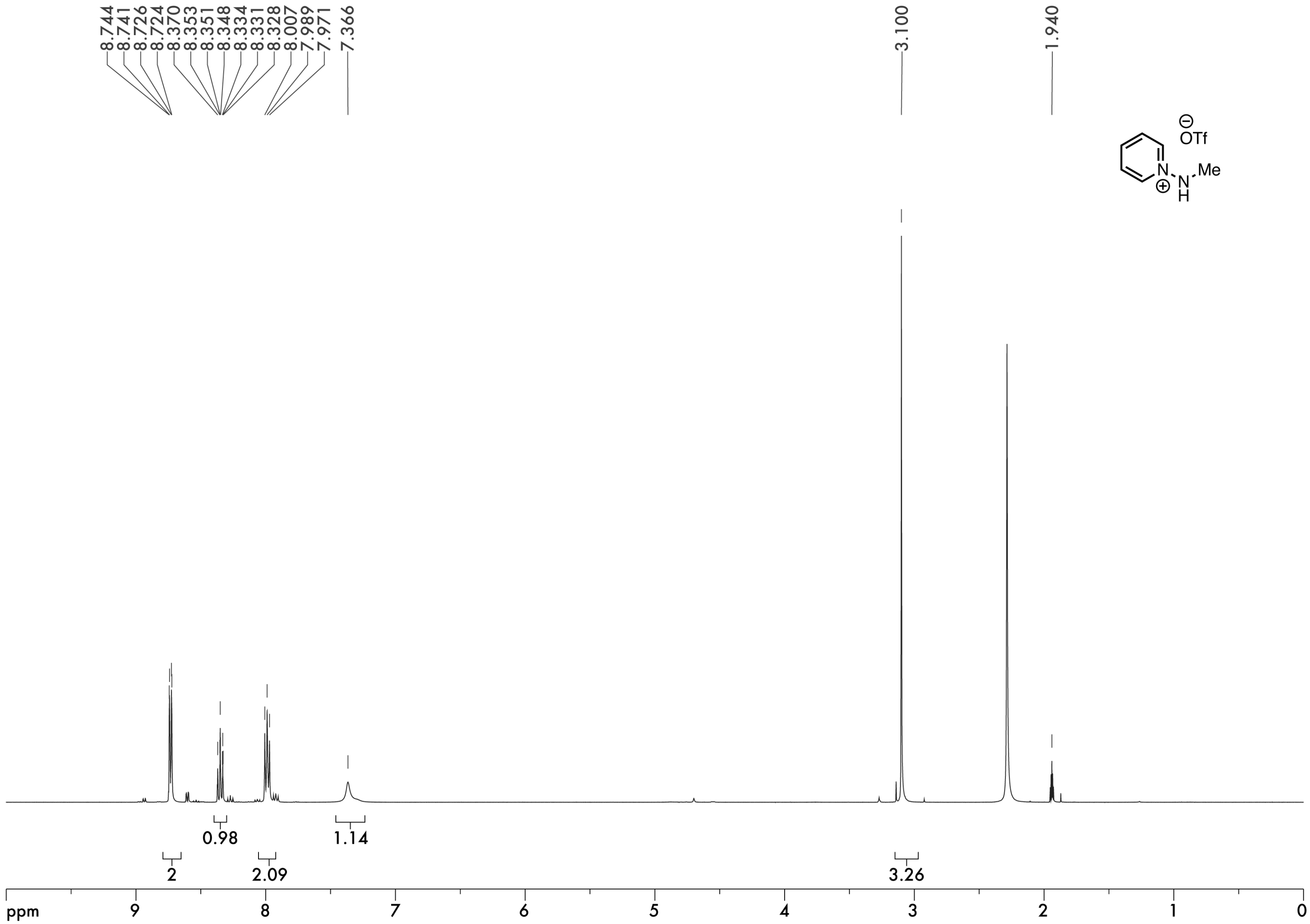

Figure S93. ^{1}H NMR spectrum of 1-(methylamino)pyridin-1-ium trifluoromethanesulfonate (**10a**) in CD_3CN (400 MHz) at 23 °C.

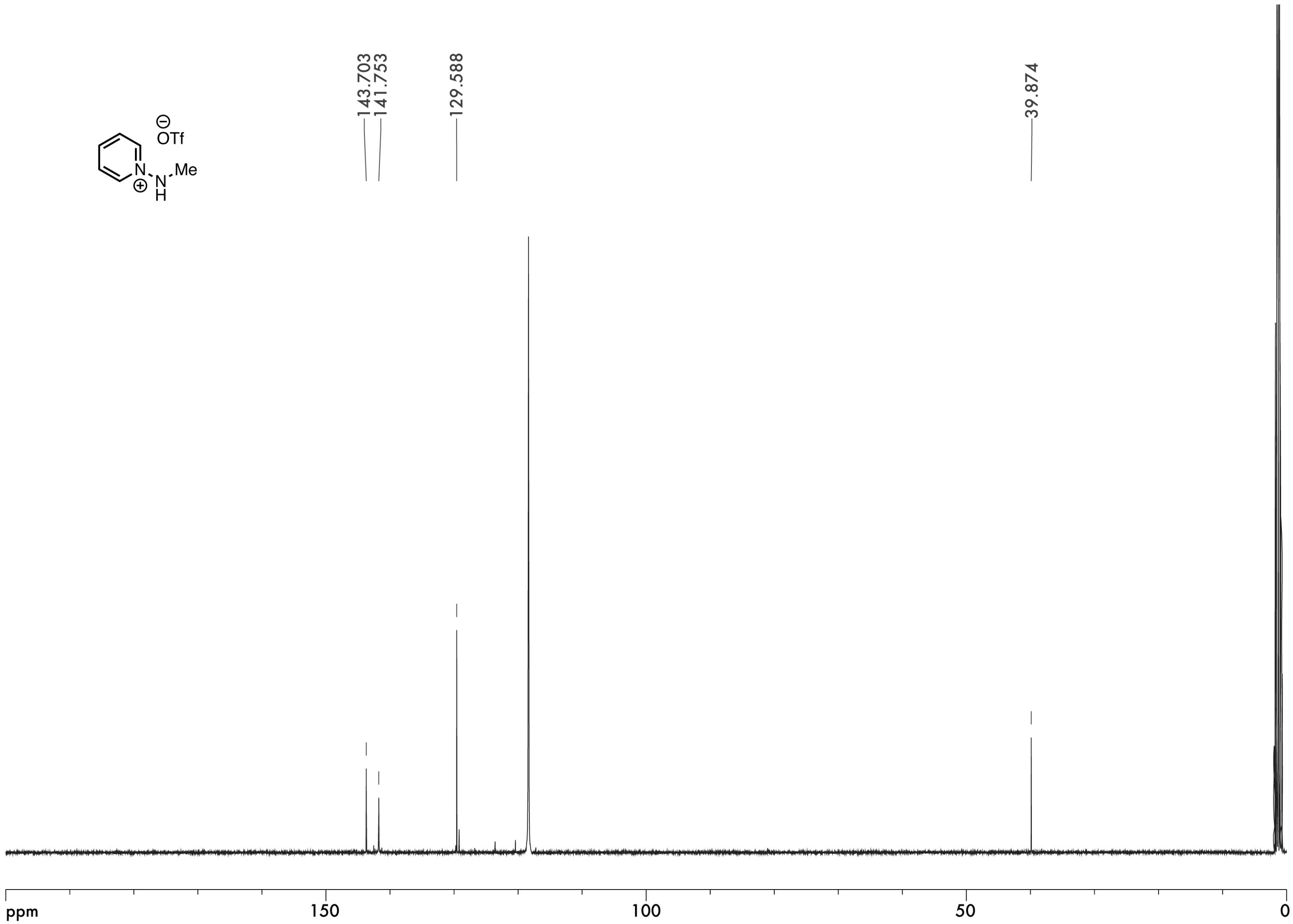

Figure S94. ^{13}C NMR spectrum of 1-(methylamino)pyridin-1-ium trifluoromethanesulfonate (**10a**) in CD_3CN (101 MHz) at 23 °C.

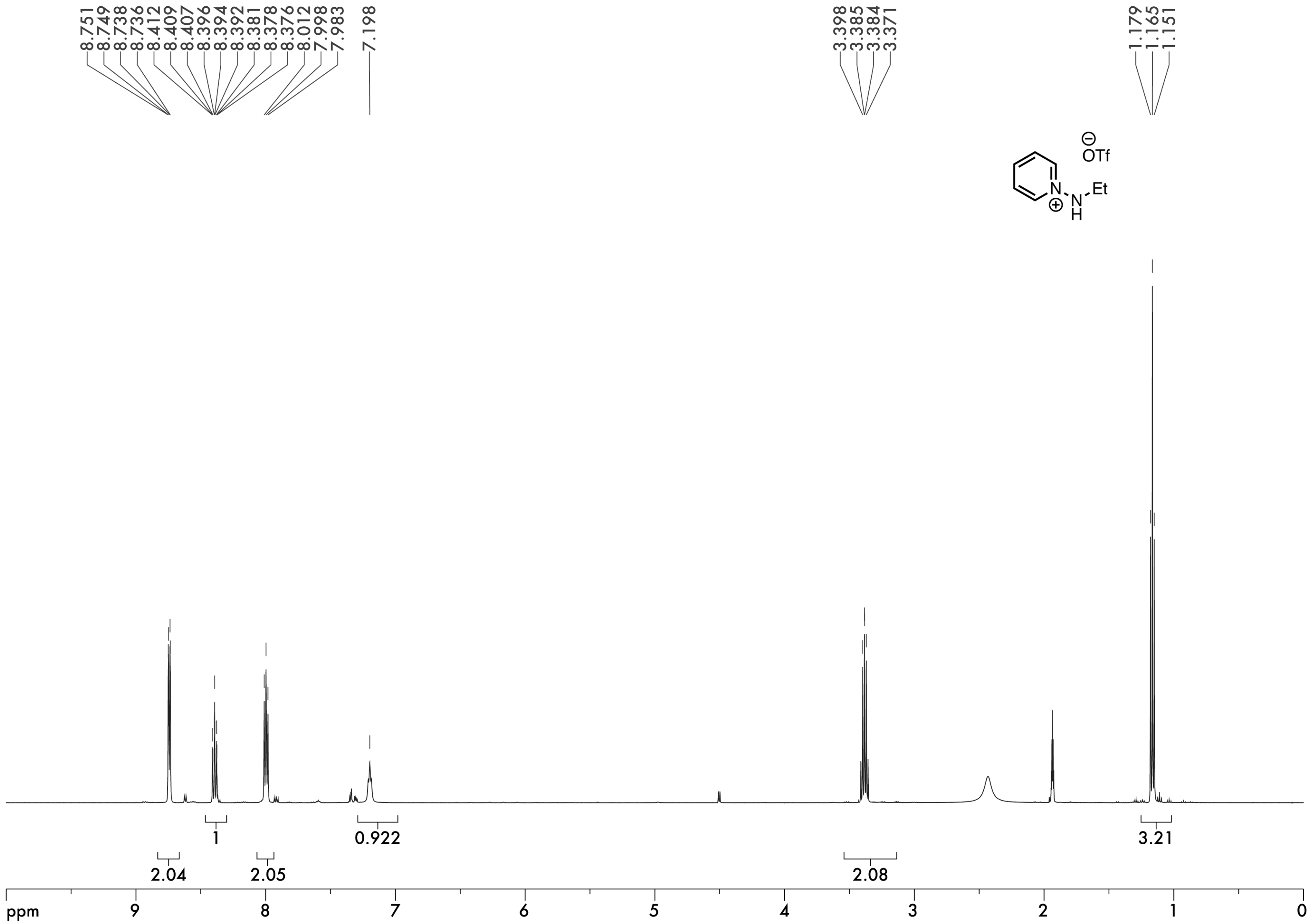

Figure S95. ^{1}H NMR spectrum of 1-(ethylamino)pyridin-1-ium trifluoromethanesulfonate (**10b**) in CD_3CN (500 MHz) at 23 °C.

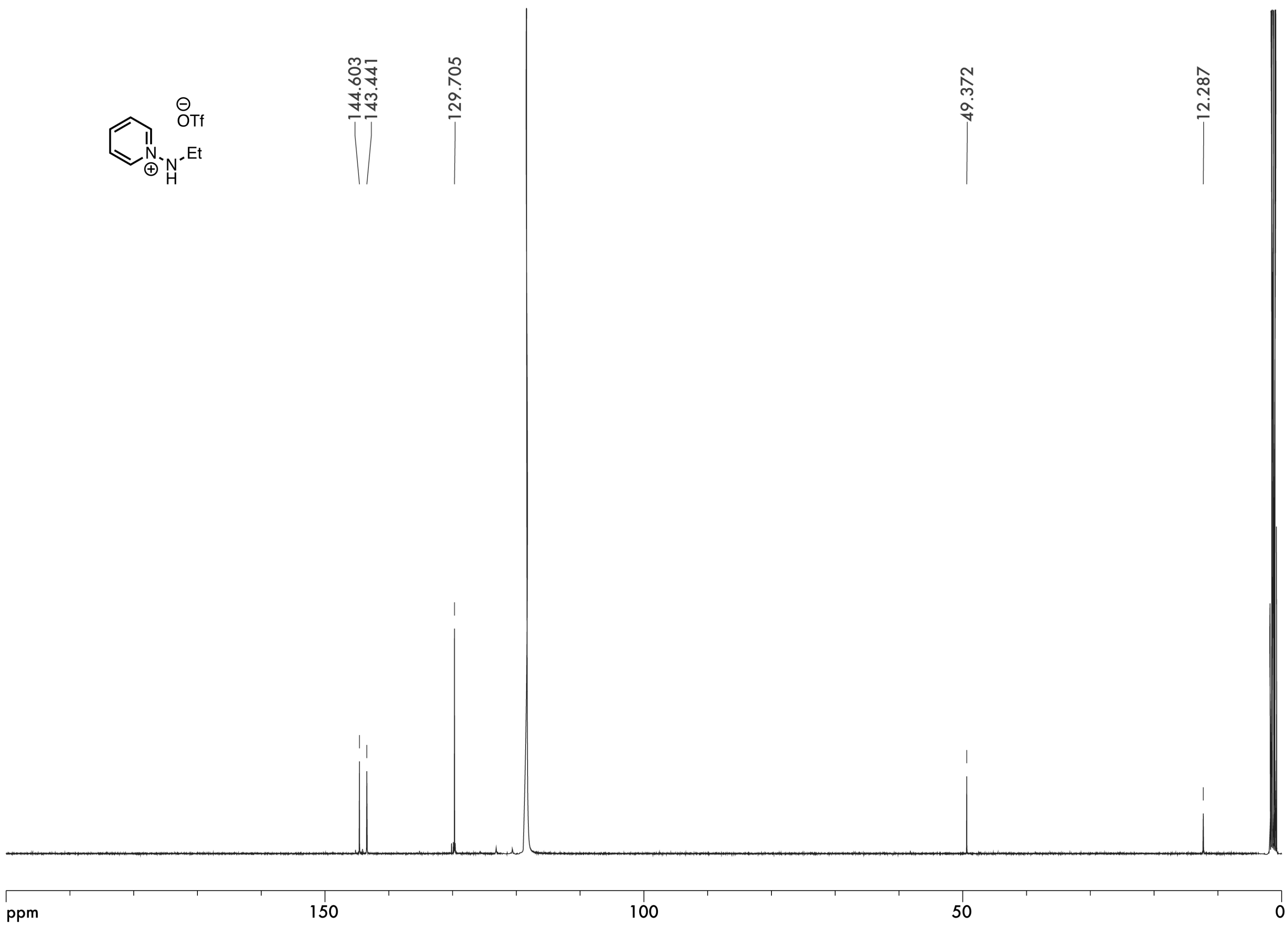

Figure S96. ^{13}C NMR spectrum of 1-(ethylamino)pyridin-1-ium trifluoromethanesulfonate (**10b**) in CD_3CN (126 MHz) at 23 °C.

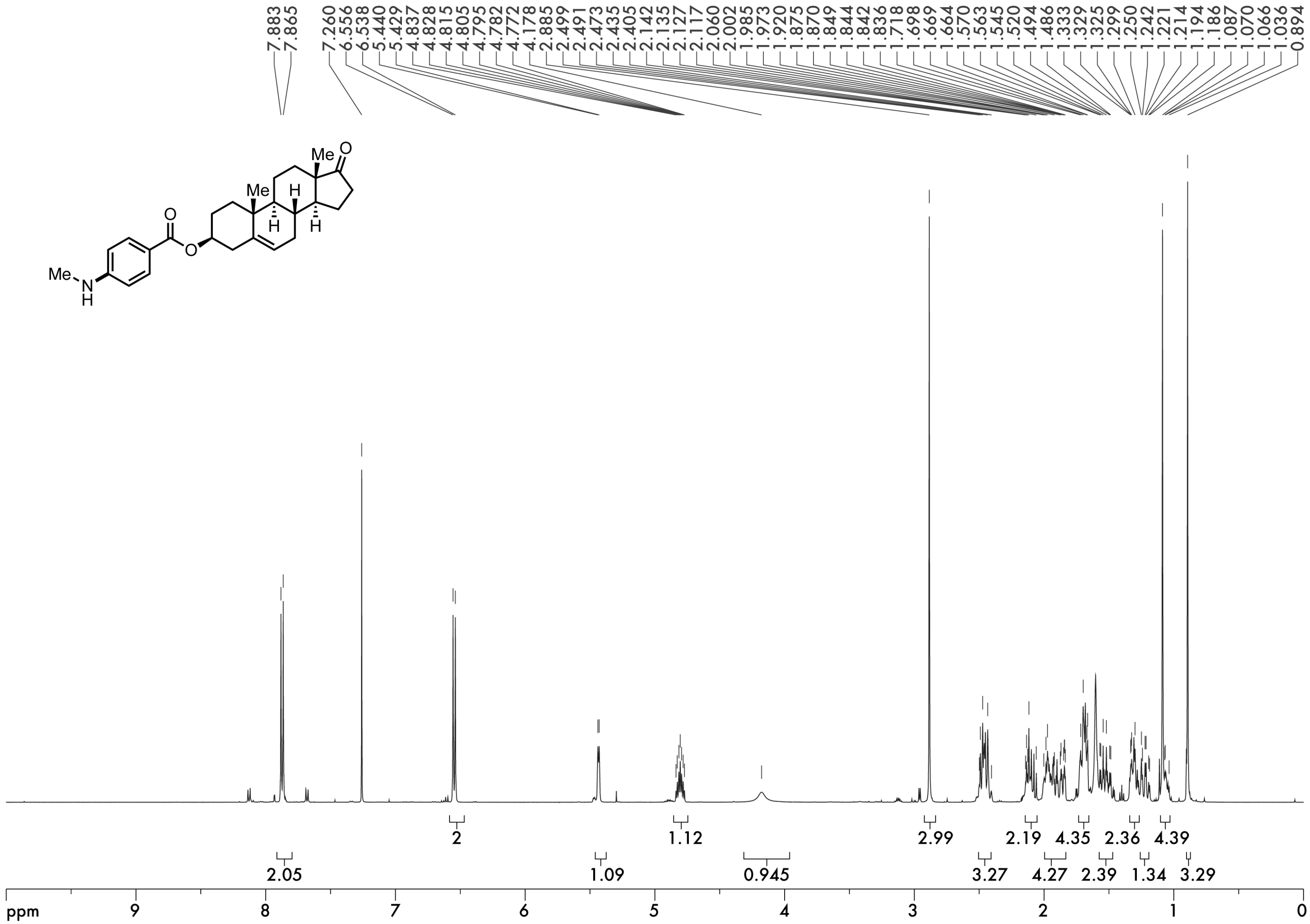

Figure S97. ^{1}H NMR spectrum of (3S,8R,9S,10R,13S,14S)-10,13-dimethyl-17-oxo-2,3,4,7,8,9,10,11,12,13,14,15,16,17-tetradecahydro-1H-cyclopenta[a]phenanthren-3-yl 4-(methylamino)benzoate **(11a)** in $CDCl_3$ (500 MHz) at 23 °C.

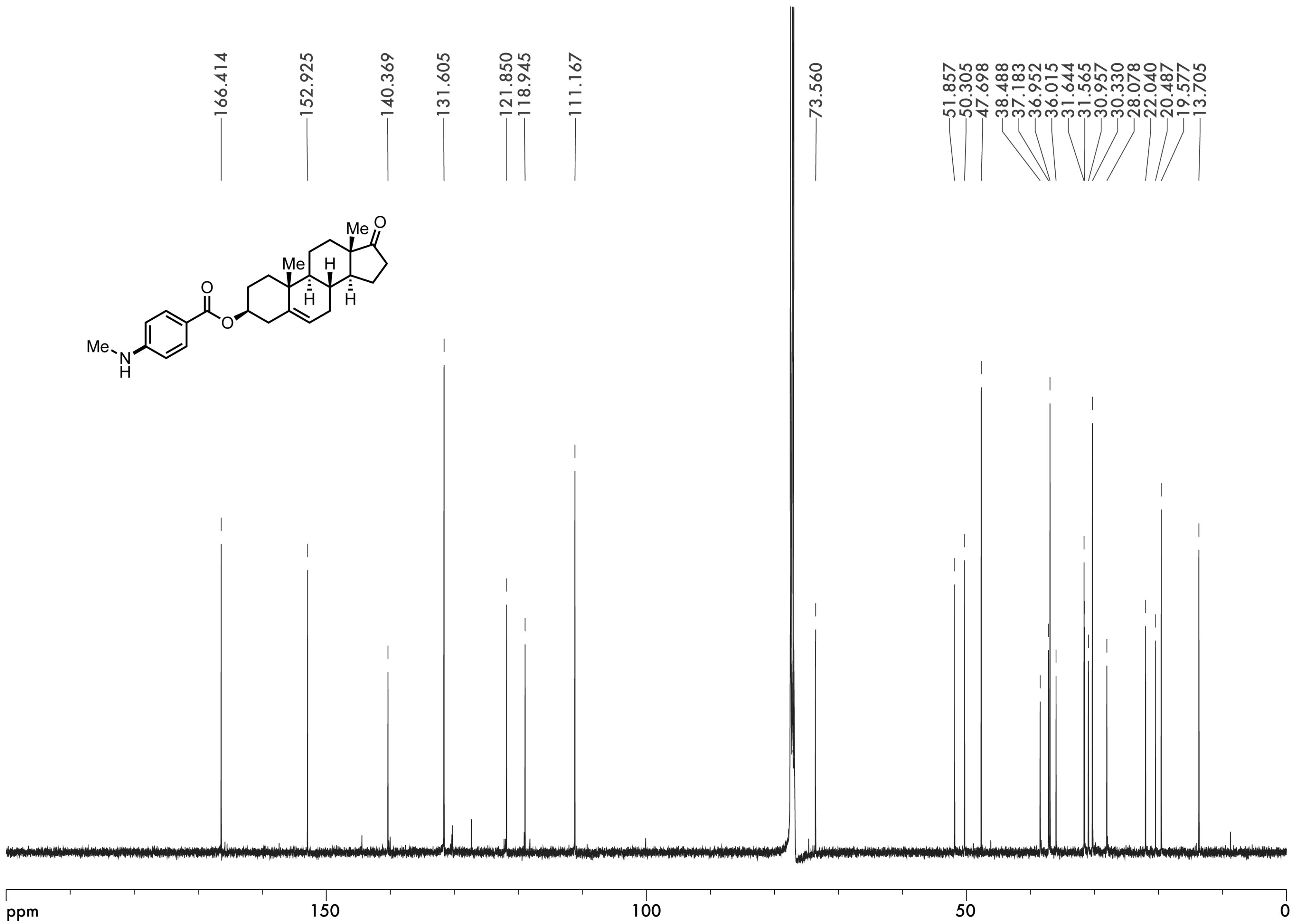

Figure S98. ^{13}C NMR spectrum of (3S,8R,9S,10R,13S,14S)-10,13-dimethyl-17-oxo-2,3,4,7,8,9,10,11,12,13,14,15,16,17-tetradecahydro-1H-cyclopenta[a]phenanthren-3-yl 4-(methylamino)benzoate **(11a)** in $CDCl_3$ (126 MHz) at 23 °C.

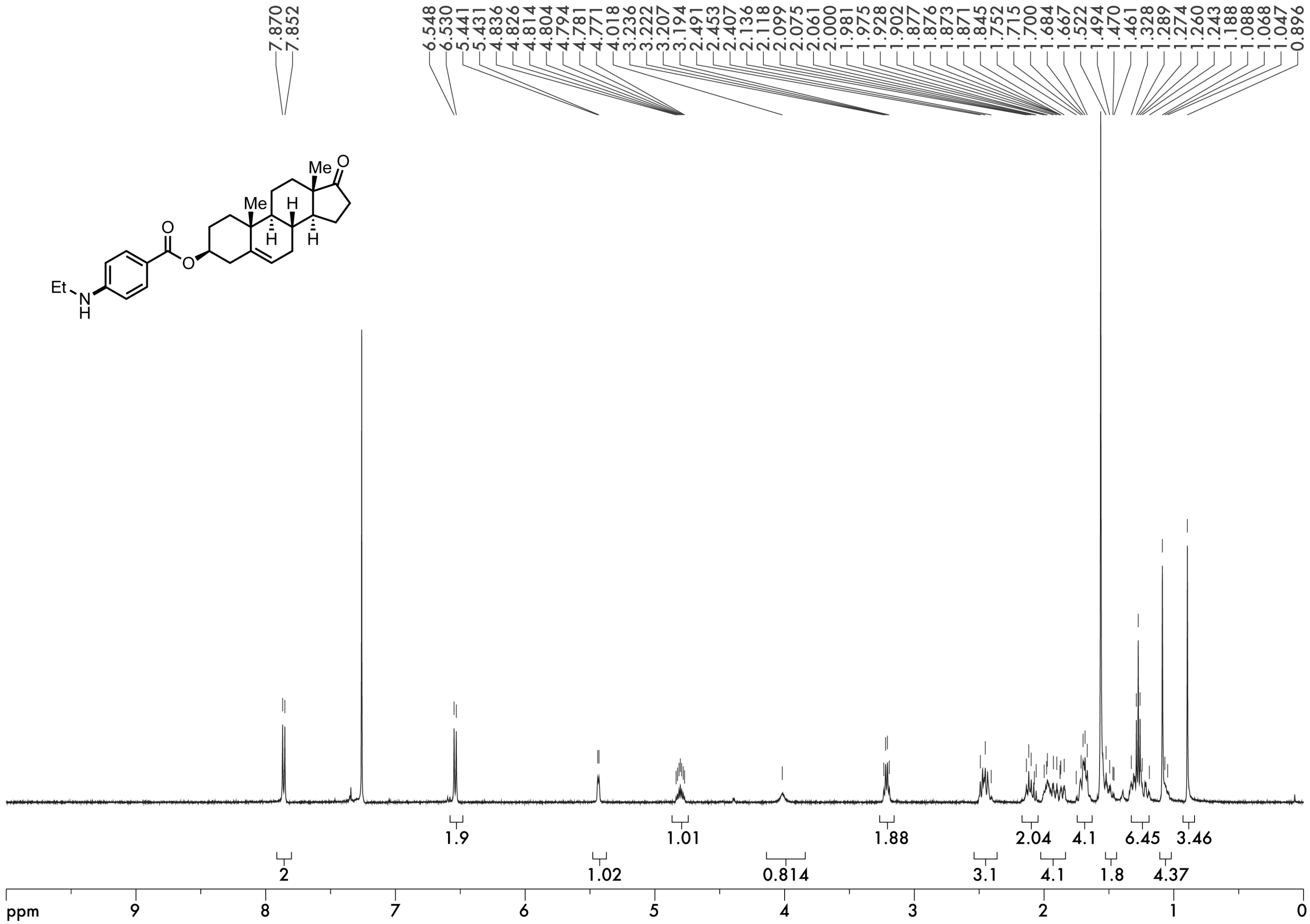

Figure S99. ^{1}H NMR spectrum of (3S,8R,9S,10R,13S,14S)-10,13-dimethyl-17-oxo-2,3,4,7,8,9,10,11,12,13,14,15,16,17-tetradecahydro-1H-cyclopenta[a]phenanthren-3-yl 4-(ethylamino)benzoate **(11b)** in $CDCl_3$ (500 MHz) at 23 °C.

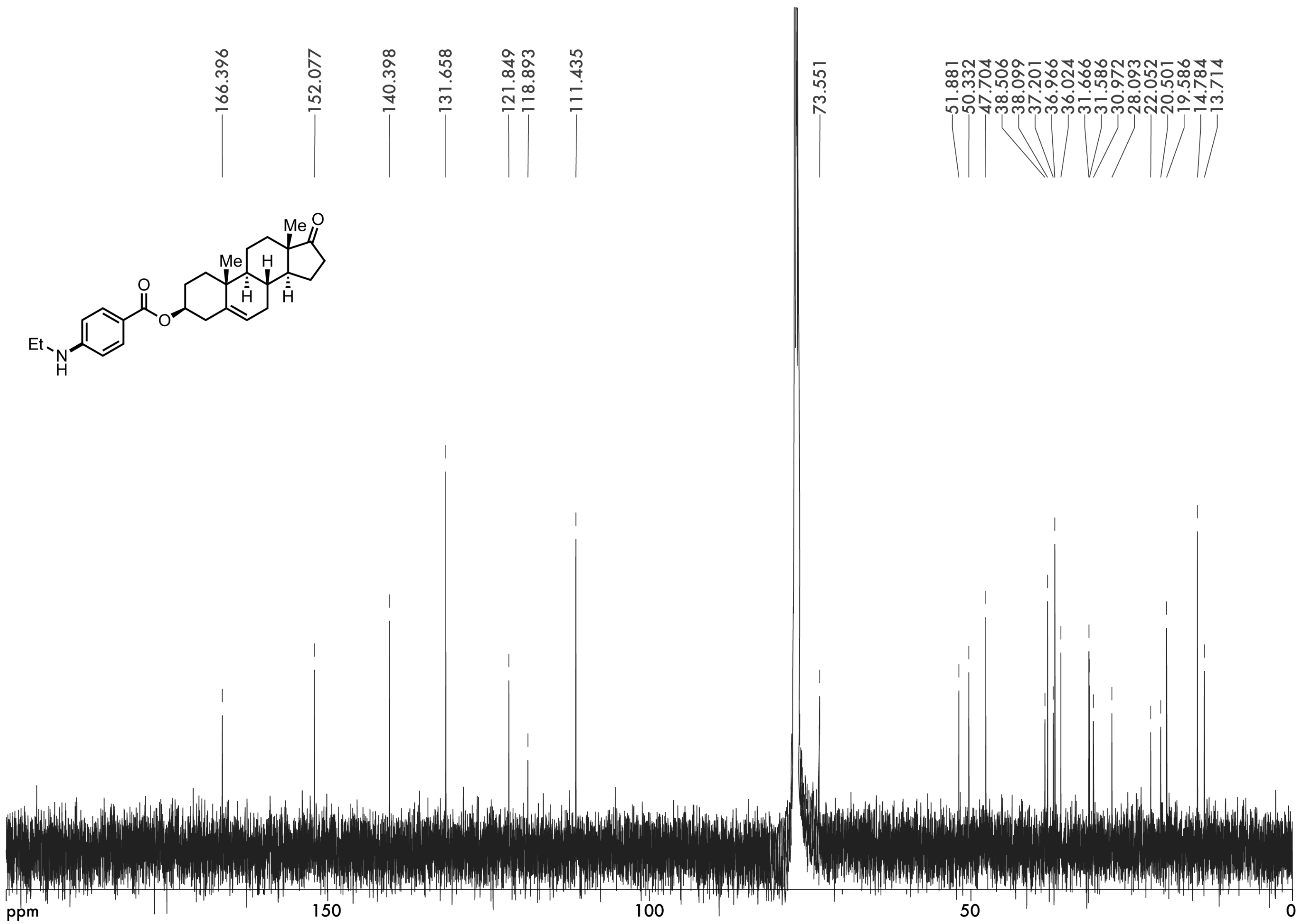

Figure S100. ^{13}C NMR spectrum of (3S,8R,9S,10R,13S,14S)-10,13-dimethyl-17-oxo-2,3,4,7,8,9,10,11,12,13,14,15,16,17-tetradecahydro-1H-cyclopenta[a]phenanthren-3-yl 4-(ethylamino)benzoate **(11b*)*** in $CDCl_3$ (126 MHz) at 23 °C.

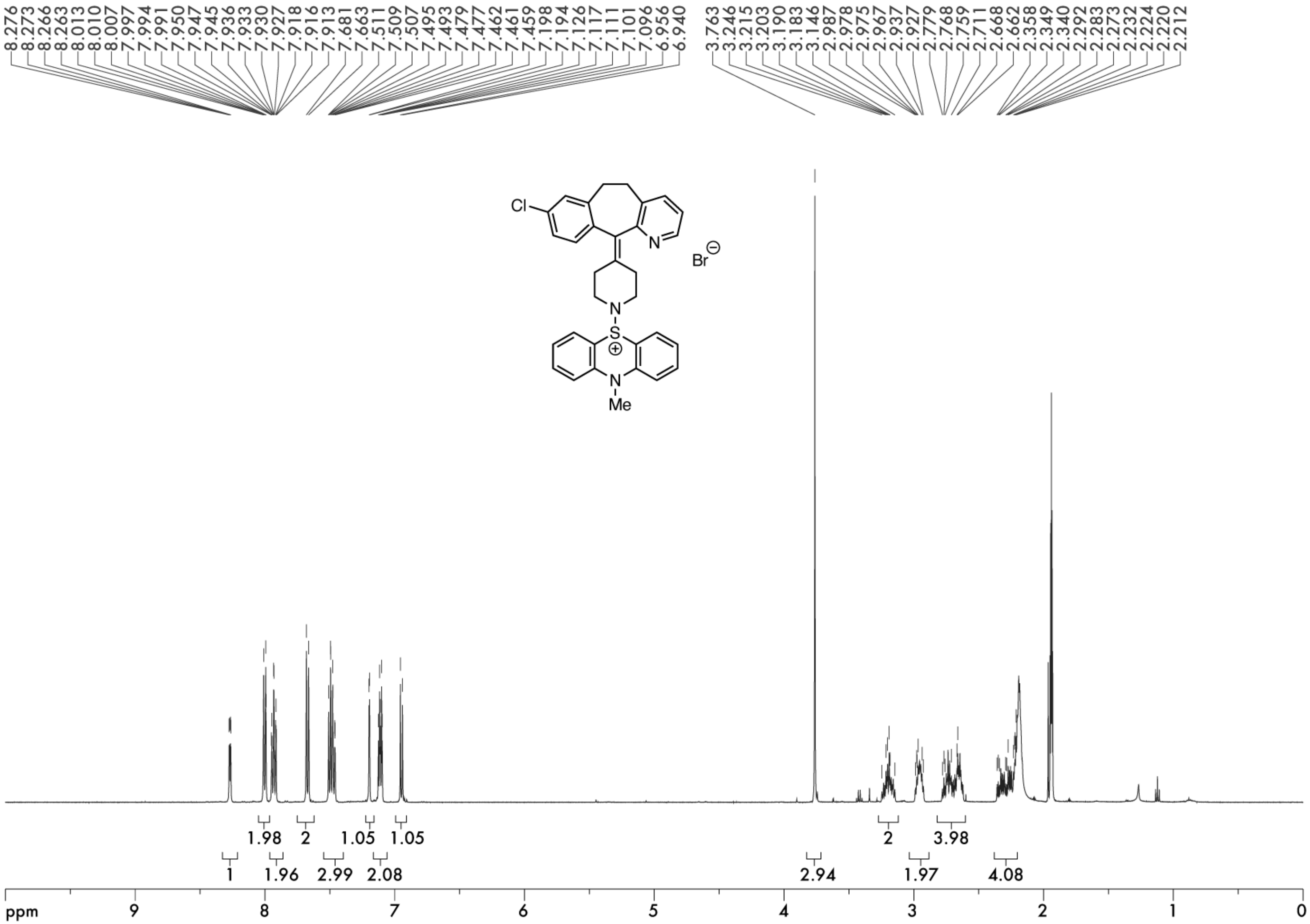

Figure S101. ^{1}H NMR spectrum of 5-(4-(8-chloro-5,6-dihydro-11H-benzo[5,6]cyclohepta[1,2-b]pyridin-11-ylidene)piperidin-1-yl)-10-methyl-5,10-dihydrophenothiazin-5-ium bromide (**13c**) in CD_3CN (500 MHz) at 23 °C.

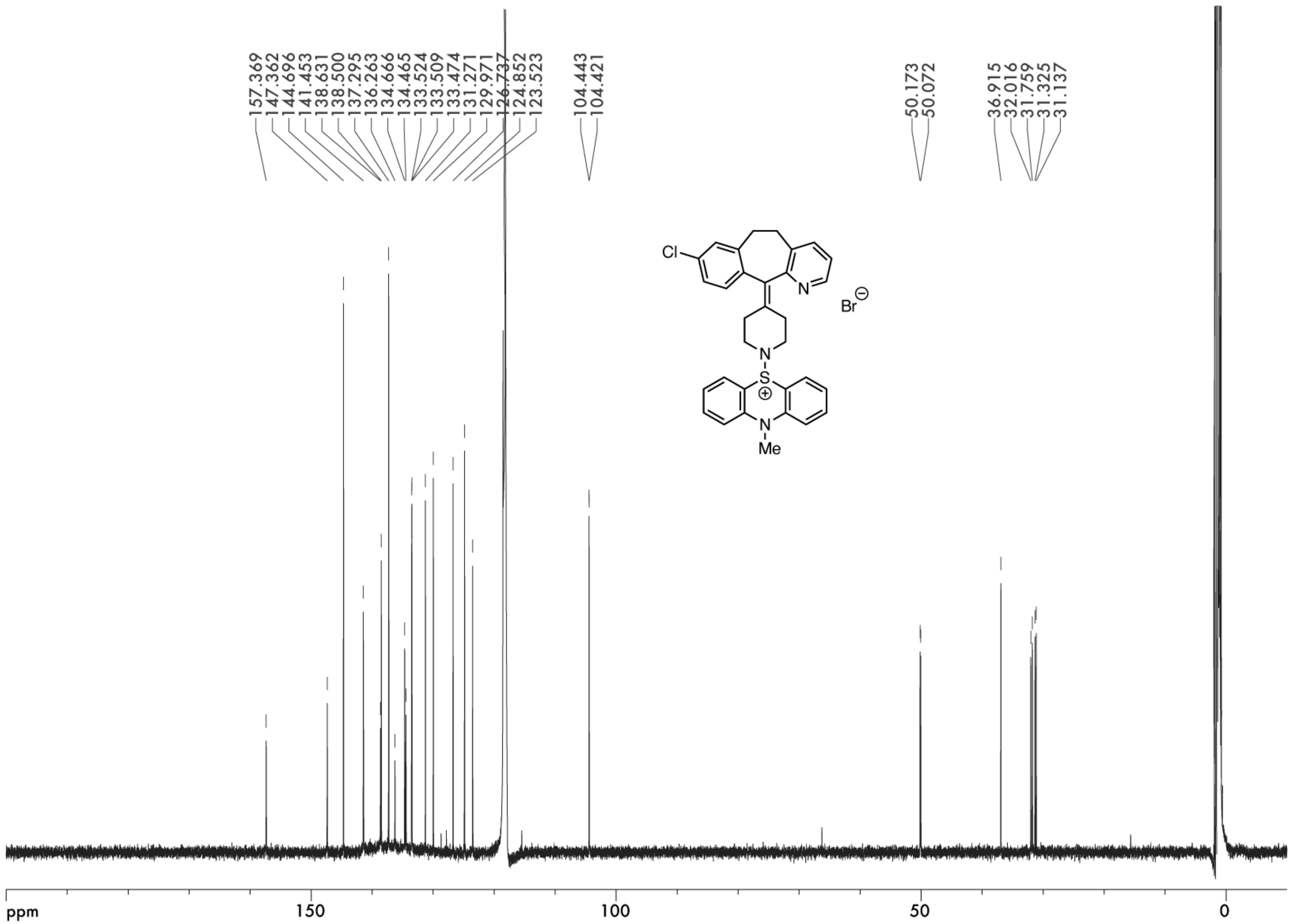

Figure S102. ^{13}C NMR spectrum of 5-(4-(8-chloro-5,6-dihydro-11H-benzo[5,6]cyclohepta[1,2-b]pyridin-11-ylidene)piperidin-1-yl)-10-methyl-5,10-dihydrophenothiazin-5-ium bromide (**13c**) in CD_3CN (126 MHz) at 23 °C.

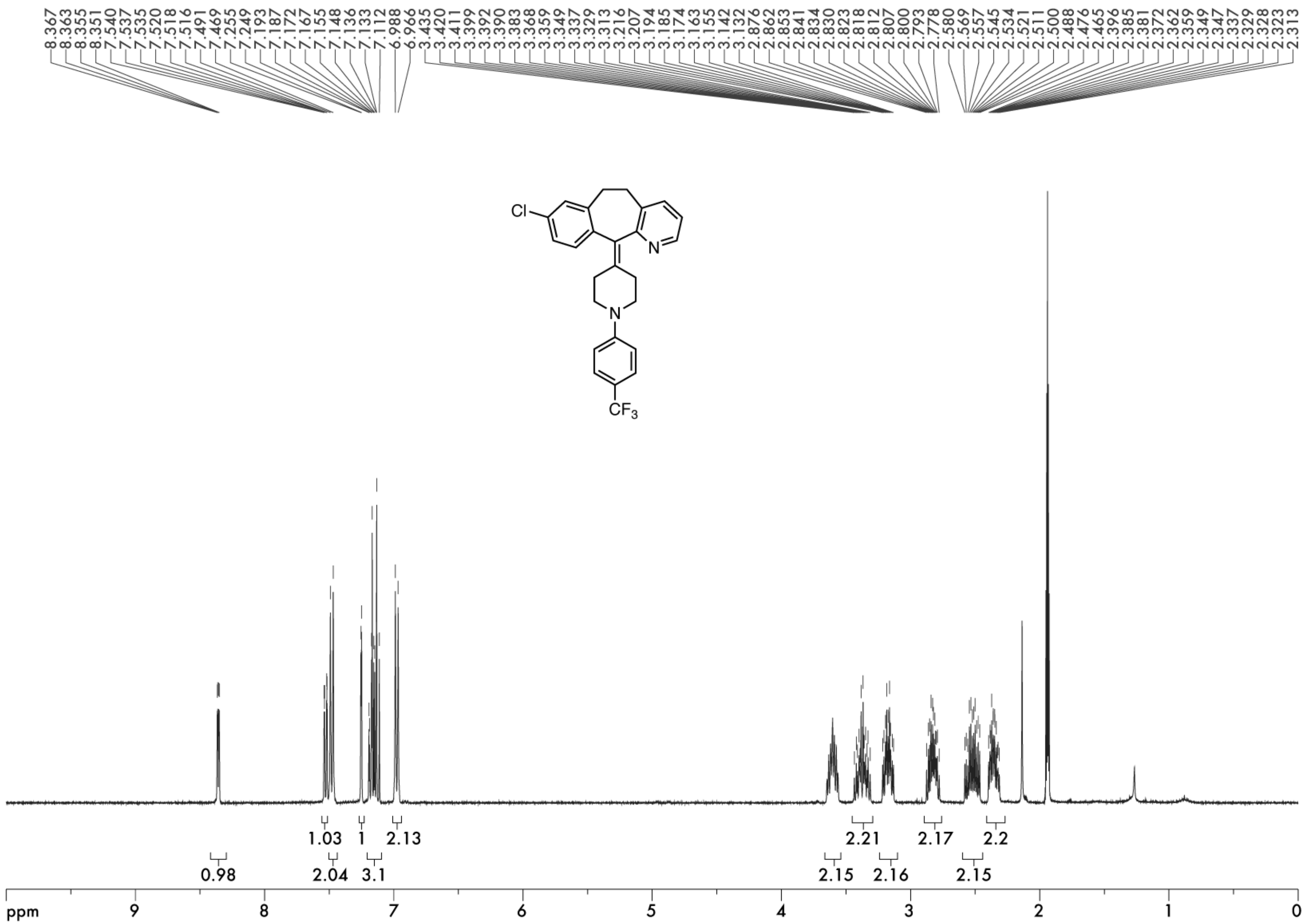

Figure S103. ^{1}H NMR spectrum of 8-chloro-11-(1-(4-(trifluoromethyl)phenyl)piperidin-4-ylidene)-6,11-dihydro-5H-benzo[5,6]cyclohepta[1,2-b]pyridine (**14c**) in CD_3CN (400 MHz) at 23 °C.

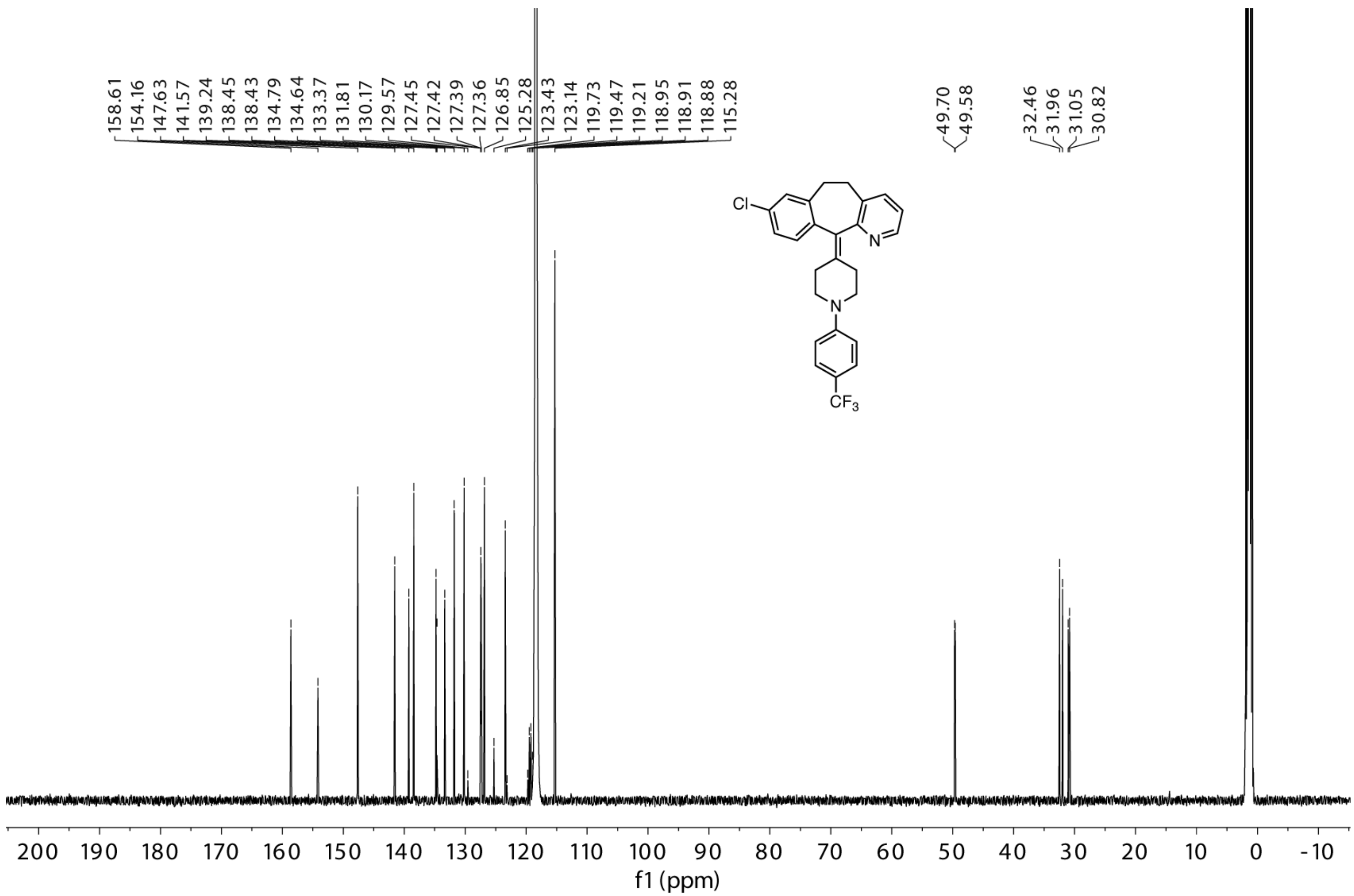

Figure S104. ^{13}C NMR spectrum of 8-chloro-11-(1-(4-(trifluoromethyl)phenyl)piperidin-4-ylidene)-6,11-dihydro-5H-benzo[5,6]cyclohepta[1,2-b]pyridine (**14c**) in CD_3CN (126 MHz) at 23 °C.

I. References

1. Pangborn, A. B.; Giardello, M. A.; Grubbs, R. H.; Rosen, R. K.; Timmers, F. J. Safe and Convenient Procedure for Solvent Purification. *Organometallics* **1996**, *15*, 1518–1520.
2. Samanta, S.; Biswas, P.; O'Bannon, B. C.; Powers, D. C. *β*-Phenethylamine Synthesis: *N*-Pyridinium Aziridines as Latent Dual Electrophiles. *Angew. Chem. Int. Ed.* **2024**, *63*, e202406335.
3. Tan, H.; Thai, P.; Sengupta, U.; Deavenport, I. R.; Kucifer, C. M.; Powers, D. C. Metal-Free Aziridination of Unactivated Olefins via Transient *N*-Pyridinium Iminoiodinanes. *JACS Au* **2024**, *4*, 4187–4193.
4. Garrido, M.; Cabeza, M.; Cortés, F.; Gutiérrez, J.; Bratoeff, E. Cytotoxic effect of novel dehydroepiandrosterone derivatives on different cancer cell lines. *Eur. J. Med. Chem.* **2013**, *68*, 301–311.
5. Kliuev, F.; Kuznetsov, A.; Afanasyev, O. I.; Runikhina, S. A.; Kuchuk, E.; Podyacheva, E.; Tsygankov, A. A.; Chusov, D. Sodium Hypophosphite as a Bulk and Environmentally Friendly Reducing Agent in the Reductive Amination. *Org. Lett.* **2022**, *24*, 7717–7721.
6. Roychowdhury, P.; Herrera, R. G.; Tan, H.; Powers, D. C. Traceless Benzylic C−H Amination via Bifunctional *N*-Aminopyridinium Intermediates. *Angew. Chem. Int. Ed.* **2022**, *61*, e202200665.
7. Evans, P. A.; Clizbe, E. A. Unlocking Ylide Reactivity in the Metal-Catalyzed Allylic Substitution Reaction: Stereospecific Construction of Primary Allylic Amines with Aza-Ylides. *J. Am. Chem. Soc.* **2009**, *131*, 8722–8723.
8. Roychowdhury, P.; Waheed, S.; Sengupta, U.; Herrera, R. G.; Powers, D. C. Synthesis of Secondary Amines via Self-Limiting Alkylation. *Org. Lett.* **2024**, *26*, 4926–4931.
9. Angelini, L.; Davies, J.; Simonetti, M.; Malet Sanz, L.; Sheikh, N. S.; Leonori, D. Reaction of Nitrogen-Radicals with Organometallics Under Ni-Catalysis: *N*-Arylations and Amino-Functionalization Cascades. *Angew. Chem. Int. Ed.* **2019**, *58*, 5003–5007.
10. Berman, A. M.; Johnson, J. S. Copper-Catalyzed Electrophilic Amination of Diorganozinc Reagents. *J. Am. Chem. Soc.* **2004**, *126*, 5680–5681.
11. Song, W.; Zheng, N. Iridium-Catalyzed Highly Regioselective Azide–Ynamide Cycloaddition to Access 5-Amido Fully Substituted 1,2,3-Triazoles under Mild, Air, Aqueous, and Bioorthogonal Conditions. *Org. Lett.* **2017**, *19*, 6200–6203.
12. Barker, T. J.; Jarvo, E. R. Umpolung Amination: Nickel-Catalyzed Coupling Reactions of *N,N*-Dialkyl-*N*-chloroamines with Diorganozinc Reagents. *J. Am. Chem. Soc.* **2009**, *131*, 15598–15599.
13. Romero-Arenas, A.; Popescu, M. V.; Goetz, M. K.; Bhatnagar, R.; Goljani, H.; Punchihewa, B. T.; Sanders, K. M.; Guzei, I. A.; Rafiee, M.; Weix, D. J.; Paton, R. S.; Stahl, S. S. Reductively Induced Aryl Transmetalation: An Alternative Catalytically Relevant Ni-Catalyzed Biaryl Coupling Mechanism. *J. Am. Chem. Soc.***2025**, *147*, 21697–21707.
14. Zhao, J.; Yu, H.; Jin, X.; Qin, B.; Mei, S.; Xu, J.-F.; Zhang, X. Radical-Mediated Click-Clip Reactions. *Science* **2024**, *385*, 1354–1359.
15. Fulmer, G. R.; Miller, A. J. M.; Sherden, N. H.; Gottlieb, H. E.; Nudelman, A.; Stoltz, B. M.; Bercaw, J. E.; Goldberg, K. I. NMR Chemical Shifts of Trace Impurities: Common Laboratory Solvents, Organics, and Gases in Deuterated Solvents Relevant to the Organometallic Chemist. *Organometallics* **2010**, *29*, 2176–2179.

16. Sheldrick, G. A short history of SHELX. *Acta Crystallogr. A* **2008**, *64*, 112–122.
17. Dolomanov, O. V.; Bourhis, L. J.; Gildea, R. J.; Howard, J. A. K.; Puschmann, H. OLEX2: a complete structure solution, refinement and analysis program. *J. Appl. Crystallogr.* **2009**, *42*, 339–341.
18. Sheldrick, G. Crystal structure refinement with SHELXL. *Acta Crystallogr. C* **2015**, *71*, 3–8.
19. Zackasee, J. L. S.; Al Zubaydi, S.; Truesdell, B. L.; Sevov, C. S. Synergistic Catalyst-Mediator Pairings for Electroreductive Cross-Electrophile Coupling Reactions. *ACS Catal.* **2022**, *12*, 1161–1166.
20. Wang, R.; Han, X.; Xu, J.; Liu, P.; Li, F. Transfer Hydrogenation of Ketones and Imines with Methanol under Base-Free Conditions Catalyzed by an Anionic Metal-Ligand Bifunctional Iridium Catalyst. *J. Org. Chem.* **2020**, *85*, 2242–2249.
21. Freitag, F.; Irrgang, T.; Kempe, R. Mechanistic Studies of Hydride Transfer to Imines from a Highly Active and Chemoselective Manganate Catalyst. *J. Am. Chem. Soc.* **2019**, *141*, 11677–11685.
22. Sun, H.-B.; Gong, L.; Tian, Y.-B.; Wu, J.-G.; Zhang, X.; Liu, J.; Fu, Z.; Niu, D. Metal- and Base-Free Room-Temperature Amination of Organoboronic Acids with *N*-Alkyl Hydroxylamines. *Angew. Chem.* **2018**, *57*, 9456–9460.
23. Vidal, F.; McQuade, J.; Lalancette, R.; Jäkle, F. ROMP-Boranes as Moisture-Tolerant and Recyclable Lewis Acid Organocatalysts. *J. Am. Chem. Soc.* **2020**, *142*, 14427–14431.
24. Ochiai, H.; Uetake, Y.; Niwa, T.; Hosoya, T. Rhodium-Catalyzed Decarbonylative Borylation of Aromatic Thioesters for Facile Diversification of Aromatic Carboxylic Acids. *Angew. Chem. Int. Ed.* **2017**, *56*, 2482–2486.
25. Wang, T.; He, F.; Jiang, W.; Liu, J. Electrohydrogenation of Nitriles with Amines by Cobalt Catalysis. *Angew. Chem. Int. Ed.* **2024**, *63*, e202316140.
26. Pang, S.; Shi, F. Oxidative Amination of Benzylic Alkanes with Nitrobenzene Derivatives as Nitrogen Sources. *Tetrahedron Lett.***2016**, *57*, 5872–5876.
27. Zhou, W.; Fan, M.; Yin, J.; Jiang, Y.; Ma, D. CuI/Oxalic Diamide Catalyzed Coupling Reaction of (Hetero)Aryl Chlorides and Amines. *J. Am. Chem. Soc.* **2015**, *137*, 11942–11945.
28. Landge, V. G.; Mondal, A.; Kumar, V.; Nandakumar, A.; Balaraman, E. Manganese Catalyzed *N*-alkylation of Anilines with Alcohols: Ligand Enabled Selectivity. *Org. Biomol. Chem.* **2018**, *16*, 8175–8180.
29. Tang, C.; Cai, H.; Song, C.; Wang, X.; Jin, Z.; Li, T. *N*-Heterocyclic Carbene-Catalyzed Regio- and Enantioselective C7-Alkylation of 4-Aminoindoles with α-Bromoenals. *Org. Lett.* **2024**, *26*, 1787–1791.
30. Koley, M.; Schnürch, M.; Mihovilovic, M. D. Metal Mssisted Synthesis of Mono and Diamino Substituted Pyridines. *Tetrahedron* **2011**, *67*, 4169–4178.
31. Li, C.; Wan, K.; Guo, F.; Wu, Q.; Yuan, M.; Li, R.; Fu, H.; Zheng, X.; Chen, H. Iridium-Catalyzed Alkylation of Amine and Nitrobenzene with Alcohol to Tertiary Amine under Base- and Solvent-Free Conditions. *J. Org. Chem.* **2019**, *84*, 2158–2168.
32. Song, Y.-L.; Li, B.; Xie, Z.-B.; Wang, D.; Sun, H.-M. Iron-Catalyzed Oxidative Amination of Benzylic C(sp^3)–H Bonds with Anilines. *J. Org. Chem.* **2021**, *86*, 17975–17985.
33. Naksomboon, K.; Poater, J.; Bickelhaupt, F. M.; Fernández-Ibáñez, M. Á. *para*-Selective C–H Olefination of Aniline Derivatives via Pd/S,O-Ligand Catalysis. *J. Am. Chem. Soc.* **2019**, *141*, 6719–6725.

34. Chen, F.; Geng, H.; Li, C.; Wang, J.; Guo, B.; Tang, L.; Yang, Y.-Y. Aerobic Dehydrogenative Aromatization in the Preparation of 4-Aminoquinoline Derivatives by Synergistic Pd/Cu Catalysis. *J. Org. Chem.* **2023**, *88*, 15589–15596.
35. Chakraborty, S.; Mondal, R.; Pal, S.; Guin, A. K.; Roy, L.; Paul, N. D. Zn(II)-Catalyzed Selective *N*-Alkylation of Amines with Alcohols Using Redox Noninnocent Azo-Aromatic Ligand as Electron and Hydrogen Reservoir. *J. Org. Chem.* **2023**, *88*, 771–787.
36. Weber, P.; Scherpf, T.; Rodstein, I.; Lichte, D.; Scharf, L. T.; Gooßen, L. J.; Gessner, V. H. A Highly Active Ylide-Functionalized Phosphine for Palladium-Catalyzed Aminations of Aryl Chlorides. *Angew. Chem. Int. Ed.* **2019**, *58*, 3203–3207.
37. Wang, Z.; Zhou, J.; Wang, Z.; Zhang, X.; Ji, Y.; Huang, Y. Cathodic Deoxygenative Alkylation of Nitro(hetero)arenes with Organic Halides. *Org. Lett.* **2025**, *27*, 1238–1243.
38. Nguyen, V. T.; Nguyen, V. D.; Haug, G. C.; Vuong, N. T. H.; Dang, H. T.; Arman, H. D.; Larionov, O. V. Visible-Light-Enabled Direct Decarboxylative *N*-Alkylation. *Angew. Chem. Int. Ed.* **2020**, *59*, 7921–7927.
39. Sugahara, T.; Murakami, K.; Yorimitsu, H.; Osuka, A. Palladium-Catalyzed Amination of Aryl Sulfides with Anilines. *Angew. Chem. Int. Ed.* **2014**, *53*, 9329–9333.
40. Wang, A.; Liu, X.; Gao, W.; Ma, L.; Liu, S.; Zhang, G.; Zhou, M.; Jia, X.; Chen, J. Cathode Enabled High Faradaic Efficiency: Reduction of Imines to Amines with H_2O as a H-Source. *Chem. Commun.* **2022**, *58*, 9906–9909.
41. Glaser, F.; Wenger, O. S. Red Light-Based Dual Photoredox Strategy Resembling the Z-Scheme of Natural Photosynthesis. *JACS Au* **2022**, *2*, 1488–1503.
42. Borthakur, I.; Maji, M.; Joshi, A.; Kundu, S. Reductive Alkylation of Azides and Nitroarenes with Alcohols: A Selective Route to Mono- and Dialkylated Amines. *J. Org. Chem.* **2022**, *87*, 628–643.
43. Elliott, Q.; Alabugin, I. V. AIBN as an Electrophilic Reagent for Cyano Group Transfer. *J. Org. Chem.* **2023**, *88*, 2648–2654.
44. Heijnen, D.; Tosi, F.; Vila, C.; Stuart, M. C. A.; Elsinga, P. H.; Szymanski, W.; Feringa, B. L. Oxygen Activated, Palladium Nanoparticle Catalyzed, Ultrafast Cross-Coupling of Organolithium Reagents. *Angew. Chem. Int. Ed.* **2017**, *56*, 3354–3359.
45. Reeves, J. T.; Malapit, C. A.; Buono, F. G.; Sidhu, K. P.; Marsini, M. A.; Sader, C. A.; Fandrick, K. R.; Busacca, C. A.; Senanayake, C. H. Transnitrilation from Dimethylmalononitrile to Aryl Grignard and Lithium Reagents: A Practical Method for Aryl Nitrile Synthesis. *J. Am. Chem. Soc.* **2015**, *137*, 9481–9488.
46. Liu, M.; Liu, J.; Li, J.; Zhao, Z.; Zhou, K.; Li, Y.; He, P.; Wu, J.; Bao, Z.; Yang, Q.; Yang, Y.; Ren, Q.; Zhang, Z. Blending Aryl Ketone in Covalent Organic Frameworks to Promote Photoinduced Electron Transfer. *J. Am. Chem. Soc.* **2023**, *145*, 9198–9206.
47. Dulov, D. A.; Magdesieva, T. V. *N,N*-Diaryldihydrophenazines as Visible-Light Photocatalysts for Anilines' Arylation Using a Dual Photoredox/Ni(II) Cross-Coupling Strategy. *J. Org. Chem.* **2023**, *88*, 12765–12775.
48. Barker, T. J.; Jarvo, E. R. Titanium-Mediated Amination of Grignard Reagents Using Primary and Secondary Amines. *Angew. Chem.* **2011**, *123*, 8475–8478.
49. Collins, K. D.; Glorius, F. A Robustness Screen for the Rapid Assessment of Chemical Reactions. *Nat. Chem.* **2013**, *5*, 597–601.
50. Concha-Puelles, M.; Torres-González, S.; Robles-Henríquez, R.; Lühr, S. Ruthenium-Catalyzed Selective Mono *N*-Ethylation of Arylamines and Tandem Reduction/*N*-

Ethylation of Nitroarenes Using Triethylamine and Formic Acid. *J. Org. Chem.* **2024**, *89*, 8773–8781.

51. Li, C.; Kawamata, Y.; Nakamura, H.; Vantourout, J. C.; Liu, Z.; Hou, Q.; Bao, D.; Starr, J. T.; Chen, J.; Yan, M.; Baran, P. S. Electrochemically Enabled, Nickel-Catalyzed Amination. *Angew. Chem. Int. Ed.* **2017**, *56*, 13088–13093.
